# Effects of opioids administered via intravenous or epidural patient-controlled analgesia after caesarean section: A network meta-analysis of randomised controlled trials

**DOI:** 10.1016/j.eclinm.2022.101787

**Published:** 2022-12-24

**Authors:** Chun-Yu Chang, Yu-Kang Tu, Ming-Chang Kao, Ping-Cheng Shih, I-Min Su, Han-Yu Lin, Yung-Jiun Chien, Meng-Yu Wu, Chih-Hao Chen, Chu-Ting Chen

**Affiliations:** a Department of Anesthesiology, Taipei Tzu Chi Hospital, Buddhist Tzu Chi Medical Foundation, New Taipei City, Taiwan; b Institute of Epidemiology & Preventive Medicine, College of Public Health, National Taiwan University, Taipei, Taiwan; c Department of Dentistry, National Taiwan University Hospital, Taipei, Taiwan; d Department of Anesthesiology, New Taipei Municipal TuCheng Hospital (Built and Operated by Chang Gung Medical Foundation), New Taipei City, Taiwan; e Department of Anesthesiology, Buddhist Tzu Chi General Hospital, Hualien, Taiwan; f Department of Physical Medicine and Rehabilitation, Taipei Tzu Chi Hospital, Buddhist Tzu Chi Medical Foundation, New Taipei City, Taiwan; g Department of Emergency Medicine, Taipei Tzu Chi Hospital, Buddhist Tzu Chi Medical Foundation, New Taipei City, Taiwan; h Department of Otolaryngology-Head and Neck Surgery, Taipei Veterans General Hospital, Taipei, Taiwan; i School of Medicine, Tzu Chi University, Hualien, Taiwan

**Keywords:** Caesarean section, Epidural, Network meta-analysis, Opioids, Patient-controlled analgesia

## Abstract

**Background:**

Post-caesarean section analgesia is important physiologically and psychologically for both mothers and infants. Patient-controlled analgesia is a well-established method of administering opioids for postoperative pain. However, to date, no study has systematically investigated the effects of opioids administered through intravenous patient-controlled analgesia (IVPCA) or patient-controlled epidural analgesia (PCEA) in parturients who have undergone caesarean section.

**Methods:**

This systematic review and network meta-analysis aimed to evaluate the analgesic and adverse effects of opioids administered via IVPCA or PCEA in parturients who have undergone a caesarean section. PubMed, Embase, Scopus, Web of Science, and Cochrane Library were searched from inception through 02 10, 2022 for relevant records. Randomised controlled trials (RCTs) that compared opioids administered via IVPCA or PCEA and reported outcomes of interest were included. Studies were excluded if the solution for patient-controlled analgesia contained antiemetics and/or other analgesics in addition to opioids. The methodological quality of RCTs was assessed using the revised Cochrane Risk of Bias Tool. Summary data were extracted from each eligible study. The primary outcome was pain intensity, and the secondary outcomes were opioid-related adverse effects. Frequentist network meta-analyses were performed using a contrast-based random-effects model. This study is registered with PROSPERO, CRD42021254040.

**Findings:**

Twenty-three studies with 2589 parturients were included. Compared with IVPCA morphine as a reference treatment, PCEA fentanyl had better analgesic effects at 4 h (mean difference [MD] in the visual analogue scale score, −0.75; 95% confidence interval [CI] [-1.16, −0.34]) and 8 h (MD, −0.93; 95% CI [-1.57, −0.28]) and yielded lower odds of developing nausea/vomiting (odds ratio [OR], 0.27; 95% CI [0.09, 0.80]) and sedation/drowsiness (OR, 0.22; 95% CI [0.11, 0.45]). However, PCEA fentanyl may be more likely to cause pruritus than IVPCA treatments.

**Interpretation:**

Considering the analgesic efficacy; opioid-induced nausea, vomiting, and sedation; and the well-being of breastfed infants, PCEA fentanyl may be the treatment of choice for post-caesarean section analgesia.

**Funding:**

The 10.13039/501100008108Taipei Tzu Chi Hospital, 10.13039/501100005925Buddhist Tzu Chi Medical Foundation (TCRD-TPE-111-27)

**Research in context**

**Evidence before this study**

Existing literature assessing the efficacy and safety of different opioids delivered via intravenous patient-controlled analgesia (IVPCA) or patient-controlled epidural analgesia (PCEA) for post-caesarean pain relief is limited. The head-to-head comparison of the same opioid agents delivered via IVPCA and PCEA is scarce. PubMed, Embase, Scopus, Web of Science, and Cochrane Library were systematically searched. Randomised controlled trials (RCTs) that enrolled patients undergoing caesarean section, compared opioids administered via IVPCA or PCEA, and reported outcomes of interest were included. Studies were excluded if the solution for patient-controlled analgesia contained antiemetics and/or other analgesics in addition to opioids. Twenty-three RCTs were considered eligible and were included in the present network meta-analysis.

**Added value of this study**

Opioids delivered via PCEA generally provide better pain relief than IVPCA. PCEA treatments yield lower odds of developing nausea, vomiting, sedation or drowsiness than IVPCA treatments. However, PCEA treatments, particularly PCEA fentanyl and PCEA sufentanil, tend to cause pruritus.

**Implications of all the available evidence**

Considering the analgesic efficacy; opioid-induced nausea, vomiting, and sedation; and the well-being of breastfed infants, PCEA fentanyl may be the treatment of choice for post-caesarean section analgesia.

## Introduction

Caesarean section is a common surgical procedure and accounts for more than 20% of childbirths worldwide.^1^ Postoperative pain control after caesarean section is of great importance and is ranked the highest priority in parturients who undergo caesarean section.^2^ Acute postoperative pain due to inadequate analgesia after caesarean section has been associated with postpartum depression.^3^ Moreover, early skin-to-skin contact has been demonstrated to promote breastfeeding and is associated with physiological and psychological benefits in both mothers and infants.^4^^,^^5^ Inadequate analgesia after delivery may affect the mothers’ willingness for early skin-to-skin contact.^6^, ^7^, ^8^ It is therefore crucial to provide effective analgesia and facilitate safe breastfeeding and bonding between the mother and infant.

Patient-controlled analgesia (PCA) is a well-established method of administering opioids for moderate-to-severe postoperative pain. Compared with conventional “as-needed” parenteral analgesia that is administered intravenously by medical staff upon demand, PCA allows timely access to pain medication with better pain control and greater patient satisfaction.^9^ PCA can be administered via an intravenous or epidural route. Patient-controlled epidural analgesia (PCEA) is generally believed to provide pain relief equal to or better than intravenous patient-controlled analgesia (IVPCA) with similar or fewer unwanted opioid-related adverse effects due to local spinal mechanisms of action. In patients undergoing intra-abdominal surgery, PCEA provides significantly better pain control than IVPCA without increased risks of opioid-related adverse effects except for pruritus.^10^ In parturients who have undergone caesarean section, PCEA fentanyl provides better pain relief and less nausea or vomiting than IVPCA fentanyl. However, PCEA fentanyl more frequently results in pruritus.^11^ Similarly, Cohen and colleagues observed that PCEA fentanyl, compared with IVPCA fentanyl, conferred better pain relief with less nausea, vomiting, or sedation. Although PCEA fentanyl resulted in more pruritus than IVPCA fentanyl, the difference was not statistically significant.^12^ In contrast, Grass and colleagues concluded that PCEA sufentanil offers no clear advantage over IVPCA morphine.^13^

To date, no study has systematically investigated the effects of opioids administered through IVPCA or PCEA in parturients who have undergone caesarean section. The aim of the present study was hence to simultaneously assess the analgesic efficacy and adverse effects of opioids administered via IVPCA or PCEA in parturients who have undergone caesarean section and to determine which treatment most effectively achieves pain relief with the least unwanted adverse effects.

## Methods

### Search strategy and selection criteria

This systematic review and network meta-analysis aimed to evaluate the analgesic effects of postoperative opioids administered via IVPCA or PCEA in parturients who have undergone a caesarean section. The primary outcome was pain intensity. The secondary outcomes were opioid-related adverse effects (i.e., nausea/vomiting, pruritus, sedation/drowsiness, respiratory depression). The present review has been registered with The International Prospective Register of Systematic Reviews (PROSPERO registration number CRD42021254040) and complies with the Preferred Reporting Items for Systematic Review and Meta-analyses (PRISMA) extension statement for network meta-analyses.^14^

Two authors (C.-Y.C. and Y.-J.C.) searched PubMed, Embase, Scopus, Web of Science, and Cochrane Library from inception through 10 May 2021. An updated search was conducted on 10 February 2022 to identify any eligible study that was published after 10 May 2021. Subject headings (i.e., MeSH terms in PubMed and Cochrane Library and Emtree terms in Embase) and search field tags of title, abstracts and keywords were used to facilitate searching. The following terms were used to search for relevant records: “patient-controlled analgesia”, “patient controlled analgesia”, “intravenous patient-controlled analgesia”, “intravenous patient controlled analgesia”, “patient-controlled intravenous analgesia”, “patient controlled intravenous analgesia”, “patient-controlled epidural analgesia”, “patient controlled epidural analgesia”, “epidural patient-controlled analgesia”, “epidural patient controlled analgesia”, “cesarean section”, “cesarean sections”, “abdominal delivery”, “abdominal deliveries”, “caesarean section”, “caesarean sections”, “c-section”, “c-sections”, “c section”, “c sections”, “postcesarean section”, and “postcaesarean section”. The search queries were constructed by using the Boolean operators “OR” and “AND” to cover similar and intersect different concepts, respectively. The identified records were screened by titles, abstracts, and keywords, and those with potential eligibility were then subject to full-text review. No language restrictions were imposed on the search strategy. Eligible studies that were published in a non-English language were translated to English using Google Translate.^15^ The reference lists of the included studies were manually searched to identify additional studies. The detailed search queries are available in Supplementary Table S1.

All studies were assessed for eligibility by two authors (C.-Y.C. and Y.-J.C.) According to the following criteria, all conditions were met: (a) the study consisted of a randomised controlled trial that compared opioids administered via IVPCA or PCEA in parturients who had undergone a caesarean delivery, (b) the study reported one of the clinical outcomes of interest, including pain intensity, nausea/vomiting, pruritus, sedation/drowsiness, and respiratory depression, and (c) the full paper of the study could be obtained. We excluded studies in which the solution for patient-controlled analgesia contained antiemetics and/or other analgesics in addition to opioids. Studies were also excluded if they were disconnected from the network map. A third author (C.-T.C.) provided a consensus or discussion if there was any discrepancy in the study selection. The methodological quality of randomised controlled trials was assessed using the revised Cochrane Risk of Bias Tool.^16^ Disagreements in the assessment were resolved through consensus or discussion.

### Data analysis

Data sets were extracted by two authors (C.-Y.C. and Y.-J.C.) from each eligible study. The required information included the author's name, publication year, study design, number of patients, anaesthetic regimen for caesarean delivery, protocol for patient-controlled analgesia, and effect estimates for clinical outcomes of interest. In studies in which the outcomes of interest were reported as graphical results, the numerical data were extracted with WebPlotDigitizer Software.^17^ The reliability of WebPlotDigitizer has been previously validated and cited in peer-reviewed articles.^18^^,^^19^ In a crossover randomised controlled trial, data that were reported before, but not after, the crossover took place were extracted.

The effect estimate for the primary outcome (i.e., pain intensity) was reported as the mean difference (MD). In studies in which the pain intensity was presented as medians and interquartile ranges, means and standard deviations were estimated using the method reported by Wan.^20^^,^^21^ The effect estimates for the secondary outcomes (i.e., nausea/vomiting, pruritus, and sedation/drowsiness) were reported as odds ratios (ORs). Sedation/drowsiness was reported as continuous data in some studies and as dichotomous data in others. Instead of analysing the continuous and dichotomous outcomes separately, which may lead to a loss of information and misleading results, in studies in which sedation/drowsiness was reported as continuous data, we calculated the standardised mean difference followed by reporting it as log odds ratios using the formula developed by Chinn,^21^^,^^22^ and thereby analysed the results together with the dichotomous data.

Pairwise meta-analyses were performed to compare different treatment arms directly. Based on the assumption of consistency and transitivity, frequentist network meta-analyses were performed for each outcome using a contrast-based random-effects model to combine the direct and indirect evidence.^23^ We estimated the probabilities of each treatment being assigned to each rank and obtained a treatment ranking from the surface under the cumulative ranking (SUCRA) curve.^24^ SUCRA, ranging from 0 to 100%, is a numeric summary of the ranking probabilities for each treatment. It can be interpreted as an index for the relative efficacy of a treatment compared to a hypothetical perfect treatment that is always the best. The higher the SUCRA value, the higher the likelihood that a treatment is better than other treatments in the network; a SUCRA value closer to 0 indicates that a treatment is more likely to be less effective than other treatments. The normalised entropy (NE) was then calculated to measure the uncertainty of the treatment ranking for each treatment.^25^ NE evaluates the distribution of ranking probabilities to measure the uncertainty of the ranking for each treatment. NE ranges from 0 to 1, with 0 indicating the greatest certainty and 1 the most uncertainty. A low NE suggests that the ranking of this treatment is less likely to change when some studies are excluded from or new studies are included in the network meta-analysis. Although no definite threshold was defined to classify the certainty of the treatment ranking, some researchers suggested dividing the NE into 4 groups, i.e., perfect (0–0.2), high (0.2–0.4), medium (0.4–0.6), and low (more than 0.6) certainty. For primary outcomes except for pain at 24 h, a sensitivity analysis was conducted to evaluate if the addition of local anaesthetics significantly alters the analgesic effects of PCEA treatments by excluding studies in which PCEA treatments contain local anaesthetics. For pain at 24 h, a meta-regression analysis was performed by including local anaesthetics as an explanatory variable because the exclusion of those studies in which PCEA treatments contained local anaesthetics would result in a disconnected network in which not all treatments could be compared in the same model. Clustered ranking plots were constructed to group the treatments into meaningful clusters according to their similarity with regard to both the analgesic efficacy and adverse effects.^26^ We evaluated the potential inconsistency using the design-by-treatment interaction model,^27^ loop inconsistency model,^27^ and node-splitting model.^28^ A comparison-adjusted funnel plot and Egger's test were used to assess publication bias.^26^ All statistical analyses were performed using the “network” suite in the statistical software package Stata, version 15 (StataCorp, College Station, TX).^23^ The quality of the direct, indirect and network evidences was evaluated using the GRADE approach (Grading of Recommendations Assessment, Development, and Evaluation).^29^^,^^30^

### Role of the funding source

The funder of the study had no role in study design, data collection, data analysis, data interpretation, or writing of the report. All authors have access to the data sets, and have agreed to submit the present study for publication.

## Results

### Study selection

**Fig. 1:**
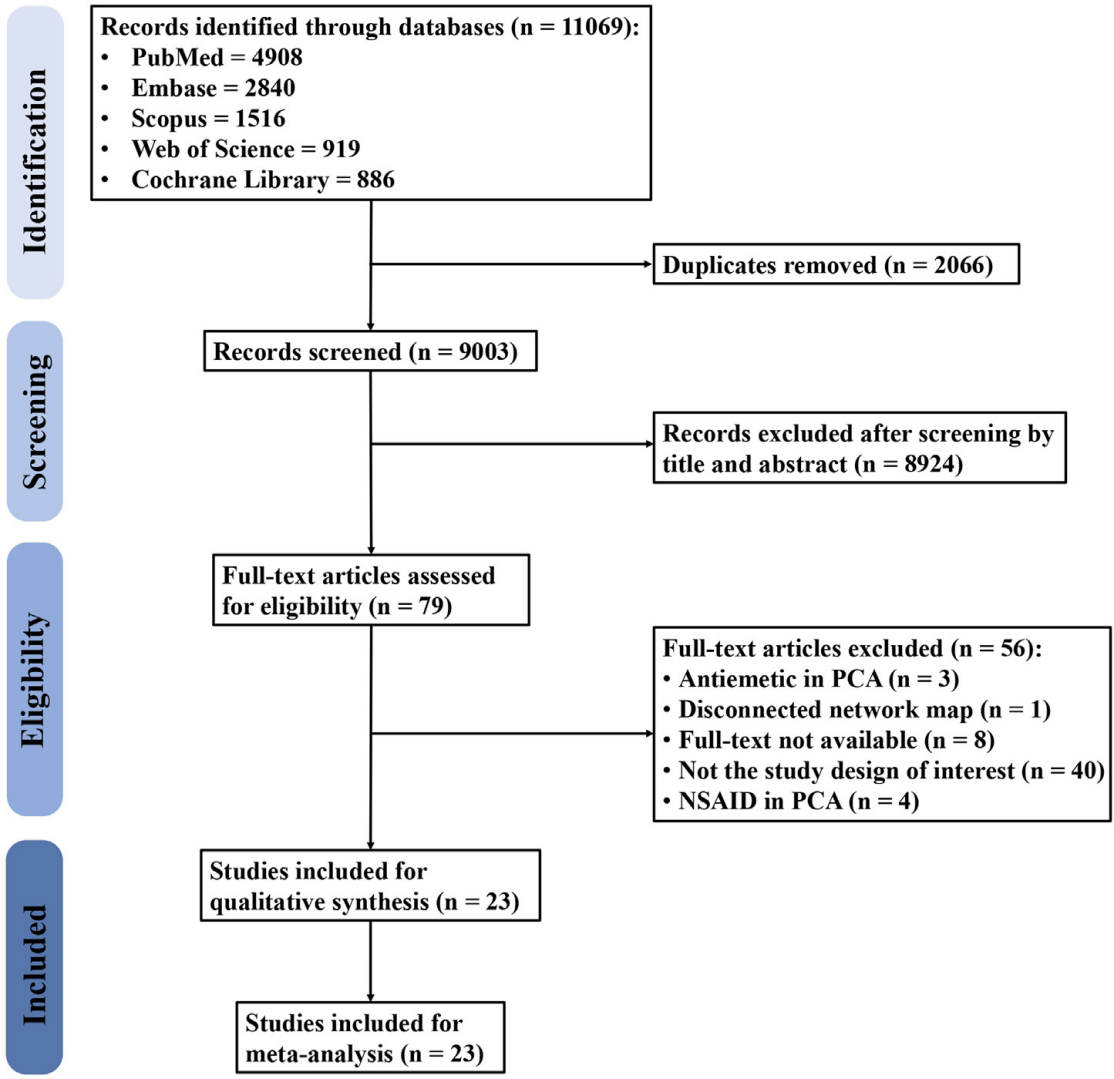
**Flow diagram of study selection**. This figure depicts the process of study selection. Reasons for excluding studies after a full-text review are provided. PCA, patient-controlled analgesia; NSAID, non-steroidal anti-inflammatory drugs.

The flow diagram of the study selection process is presented in Fig. 1. A total of 11,069 records were retrieved from five databases, including PubMed (n = 4908), Embase (n = 2840), Scopus (n = 1516), Web of Science (n = 919), and Cochrane Library (n = 886). After removing duplicates, 9003 records were screened for eligibility, 79 of which were then assessed with a full-text review, while the rest were excluded due to irrelevance. Fifty-six studies were thereafter excluded for containing antiemetics (n = 3) or analgesics (n = 4) in addition to opioids in the PCA solution, for yielding a disconnected network map (n = 1), for not having the study design of interest (n = 40), and for being unavailable for full-text review (n = 8). Finally, a total of 23 studies were included in the present study.

### Study characteristics and risk of bias

All the included studies were randomised controlled trials, and most of them had a parallel design except for 3 studies that had a crossover design.^31^, ^32^, ^33^ The anaesthetic techniques for caesarean section included general anaesthesia in 7 studies,^34^, ^35^, ^36^, ^37^, ^38^, ^39^, ^40^ epidural anaesthesia in 11 studies,^11^, ^12^, ^13^^,^^32^^,^^33^^,^^41^, ^42^, ^43^, ^44^, ^45^, ^46^ combined spinal epidural anaesthesia in 3 studies,^47^, ^48^, ^49^ spinal anaesthesia in 1 study,^50^ and epidural anaesthesia or combined spinal epidural anaesthesia in 1 study.^31^ Eight studies compared IVPCA with PCEA,^11^, ^12^, ^13^^,^^32^^,^^33^^,^^38^^,^^44^^,^^47^ whereas the other studies compared different opioids administered through the same route. Continuous infusion of opioids in addition to the demand dose was given via a patient-controlled analgesia approach in 9 studies.^12^^,^^13^^,^^34^^,^^35^^,^^38^^,^^41^, ^42^, ^43^^,^^49^ Of the 23 included studies, 11 stated that the visual analogue scale (VAS) scores were reported by mothers,^13^^,^^31^, ^32^, ^33^, ^34^^,^^36^^,^^40^^,^^44^, ^45^, ^46^^,^^50^ whereas 12 did not specify whether the reported VAS scores were subjective or objective assessments.^11^^,^^12^^,^^35^^,^^37^, ^38^, ^39^^,^^41^, ^42^, ^43^^,^^47^, ^48^, ^49^ The study characteristics are presented in detail in Table 1. Opioid consumption reported in each included study is presented in Supplementary Table S2. The assessment of the risk of bias for each included study is presented in Supplementary Fig. S1.

**Table 1:** Study characteristics.

Study	RCT type	Anaesthesia	Comparisons	Number of patients	PCA regimen					Outcomes				
					Loading	Demand	Lock-out interval (min)	Limit^a^	Continuous infusion	Pain	Nausea/vomiting	Pruritus	Sedation/drowsiness	Respiratory depression
Wu et al., 2021^49^	Parallel	CSE	• IVPCA tramadol (4 mg ml^−1^)	410	NA	4 mg	15	NA	16 mg h^−1^	V	V	V	V	
			• IVPCA hydromorphone (40 μg ml^−1^)	410	NA	40 μg	15	NA	160 μg h^−1^					
Chi et al., 2017^34^	Parallel	GA	• IVPCA sufentanil (1.5 μg ml^−1^)	73	NA	3 μg	25	10.5 μg	1.5 μg h^−1^	V	V			
			• IVPCA tramadol (10 mg ml^−1^)	73	NA	20 mg	15	70 mg	10 mg h^−1^					
Ebneshahidi et al., 2012^35^	Parallel	GA	• IVPCA morphine (0.1 mg ml^−1^)	200	NA	0.1 mg	15	NA	0.4 mg h^−1^	V	V	V	V	
			• IVPCA methadone (0.05 mg ml^−1^)	100	NA	0.05 mg	15	NA	0.2 mg h^−1^					
Saracoglu et al., 2010^40^	Parallel	GA	• IVPCA fentanyl (10 μg ml^−1^)	30	1 μg kg^−1^	20 μg	8	NA	NA	V	V			
			• IVPCA tramadol (10 mg ml^−1^)	30	1 mg kg^−1^	20 mg	8	NA	NA					
Cohen et al., 2002^12^	Parallel	EA	• IVPCA fentanyl (20 μg ml^−1^)	23	NA	20 μg	10	120 μg	40 μg h^−1^	V	V	V	V	V
			• PCEA fentanyl (20 μg ml^−1^)	21	NA	20 μg	10	120 μg	40 μg h^−1^					
Cooper et al., 1999^47^	Parallel	CSE	• IVPCA morphine (2 mg ml^−1^)	42	NA	1 mg	5	NA	NA	V	V	V	V	V
			• PCEA fentanyl (4 μg ml^−1^)	42	NA	20 μg	10	NA	NA					
Kim et al., 1999^37^	Parallel	GA	• IVPCA morphine	20	NA	1 mg	8	NA	NA		V	V		V
			• IVPCA meperidine	20	NA	10 mg	8	NA	NA					
Vercauteren et al., 1999^48^	Parallel	CSE	• PCEA sufentanil (2 μg ml^−1^)	22	20 μg	5 μg	10	20 μg	NA	V	V	V	V	V
			• PCEA tramadol (10 mg ml^−1^)	22	100 mg	25 mg	10	100 mg	NA					
James et al., 1997^50^	Parallel	SA	• IVPCA meptazinol (10 mg ml^−1^)	24	50 mg	10 mg	3	NA	NA		V		V	
			• IVPCA morphine (1 mg ml^−1^)	23	5 mg	1 mg	3	NA	NA					
Lee et al., 1997^39^	Parallel	GA	• IVPCA morphine	45	NA	1 mg	8	NA	NA		V	V	V	V
			• IVPCA meperidine	45	NA	10 mg	8	NA	NA					
Ngan Kee et al., 1997^32^	Crossover	EA	• IVPCA meperidine (10 mg ml^−1^)	20	40 mg	20 mg	6	400 mg^b^	NA	V				V
			• PCEA meperidine (10 mg ml^−1^)	20	40 mg	20 mg	6	400 mg^b^	NA					
			• IVPCA fentanyl (20 μg ml^−1^)	20	80 μg	40 μg	6	800 μg^b^	NA					
			• PCEA fentanyl (20 μg ml^−1^)	20	80 μg	40 μg	6	800 μg^b^	NA					
Goh et al., 1996^31^	Crossover	EA/CSE	• PCEA fentanyl (10 μg ml^−1^)	21	NA	50 μg	20	NA	NA	V	V	V	V	
			• PCEA meperidine (5 mg ml^−1^)	25	NA	25 mg	20	NA	NA					
Cooper et al., 1995^11^	Parallel	EA	• IVPCA fentanyl (4 μg ml^−1^)	20	NA	20 μg	10	NA	NA	V	V	V	V	V
			• PCEA fentanyl (4 μg ml^−1^)	20	NA	20 μg	10	NA	NA					
Howell et al., 1995^36^	Parallel	GA	• IVPCA morphine (1 mg ml^−1^)	19	NA	1 mg	10	6 mg	NA		V	V		
			• IVPCA fentanyl (25 μg ml^−1^)	18	NA	25 μg	10	150 μg	NA					
Lee et al., 1995^38^	Parallel	GA	• IVPCA meperidine	20	NA	10 mg	8	NA	4 mg h^−1^	V	V	V	V	V
			• PCEA meperidine^d^	20	NA	2 mg	8	NA	4 mg h^−1^					
Grass et al., 1994^13^	Parallel	EA	• IVPCA morphine	25	5 mg	5 mg → 1 mg (after 2 h)	5 → 10 (after 2 h)	NA	1 mg h^−1^	V		V	V	V
			• PCEA sufentanil	25	30 μg	8 μg → 4 μg (after 2 h)	10	NA	6 μg h^−1^					
Paech et al., 1994^33^	Crossover	EA	• PCEA meperidine (4 mg ml^−1^)	24	25 mg	20 mg	5	200 mg^c^	NA	V		V	V	
			• IVPCA meperidine (4 mg ml^−1^)	21	25 mg	20 mg	5	200 mg^c^	NA					
Cohen et al., 1993^41^	Parallel	EA	• PCEA fentanyl (2 μg ml^−1^)^e^	125	NA	6 μg	15	NA	32 μg h^−1^	V	V	V	V	V
			• PCEA sufentanil (0.8 μg ml^−1^)^e^	125	NA	2.4 μg	15	NA	12.8 μg h^−1^					
Cohen et al., 1992-A^43^	Parallel	EA	• PCEA buprenorphine (3 μg ml^−1^)^f^	26	NA	30 μg	120	NA	30 μg h^−1^	V	V	V	V	V
			• PCEA fentanyl (3 μg ml^−1^)^f^	26	NA	30 μg	120	NA	30 μg h^−1^					
Cohen et al., 1992-B^42^	Parallel	EA	• PCEA buprenorphine (3 μg ml^−1^)^g^	12	NA	30 μg	120	NA	30 μg h^−1^	V	V	V	V	V
			• PCEA fentanyl (2 μg ml^−1^)^g^	11	NA	20 μg	120	NA	20 μg h^−1^					
Parker et al., 1992^44^	Parallel	EA	• IVPCA hydromorphone (150 μg ml^−1^)	49	NA	150 μg	10	NA	NA	V	V	V	V	V
			• PCEA hydromorphone (75 μg ml^−1^)	41	225 μg	150 μg	30	NA	NA					
Sinatra et al. 1989-A^46^	Parallel	EA	• IVPCA morphine (1.5 mg ml^−1^)	24	6.0–7.5 mg (1.5 mg increments 5 min apart)	1.8 mg	8	NA	NA	V	V	V	V	
			• IVPCA meperidine (15 mg ml^−1^)	25	60–75 mg (15 mg increments 5 min apart)	18 mg	8	NA	NA					
			• IVPCA oxymorphone (0.25 mg ml^−1^)	26	1.00–1.25 mg (0.25 mg increments 5 min apart)	0.3 mg	8	NA	NA					
Sinatra et al. 1989-B^45^	Parallel	EA	• IVPCA morphine (1.5 mg ml^−1^)	16	NA	1.8 mg	8	10.8 mg	NA	V	V	V	V	
			• IVPCA oxymorphone (0.25 mg ml^−1^)	16	NA	0.3 mg	8	1.8 mg	NA					

CSE, combined spinal-epidural anaesthesia; EA, epidural anaesthesia; PCEA, epidural patient-controlled analgesia; GA, general anaesthesia; IVPCA, intravenous patient-controlled analgesia; NA, not applicable; PCA, patient-controlled analgesia; RCT, randomised controlled trial; SA, spinal anaesthesia.

a One-hour limit unless specified otherwise.

b Four-hour limit.

c Two-hour limit.

d The solution contains 0.07% bupivacaine in addition to opioids.

e The solution contains 0.01% bupivacaine and epinephrine (0.5 μg ml^−1^) in addition to opioids.

f The solution contains 0.015% bupivacaine and epinephrine (1 μg ml^−1^) in addition to opioids.

g The solution contains 0.03% bupivacaine in addition to opioids.

### Primary outcome

#### Pain intensity at 4 h after surgery

**Fig. 2:**
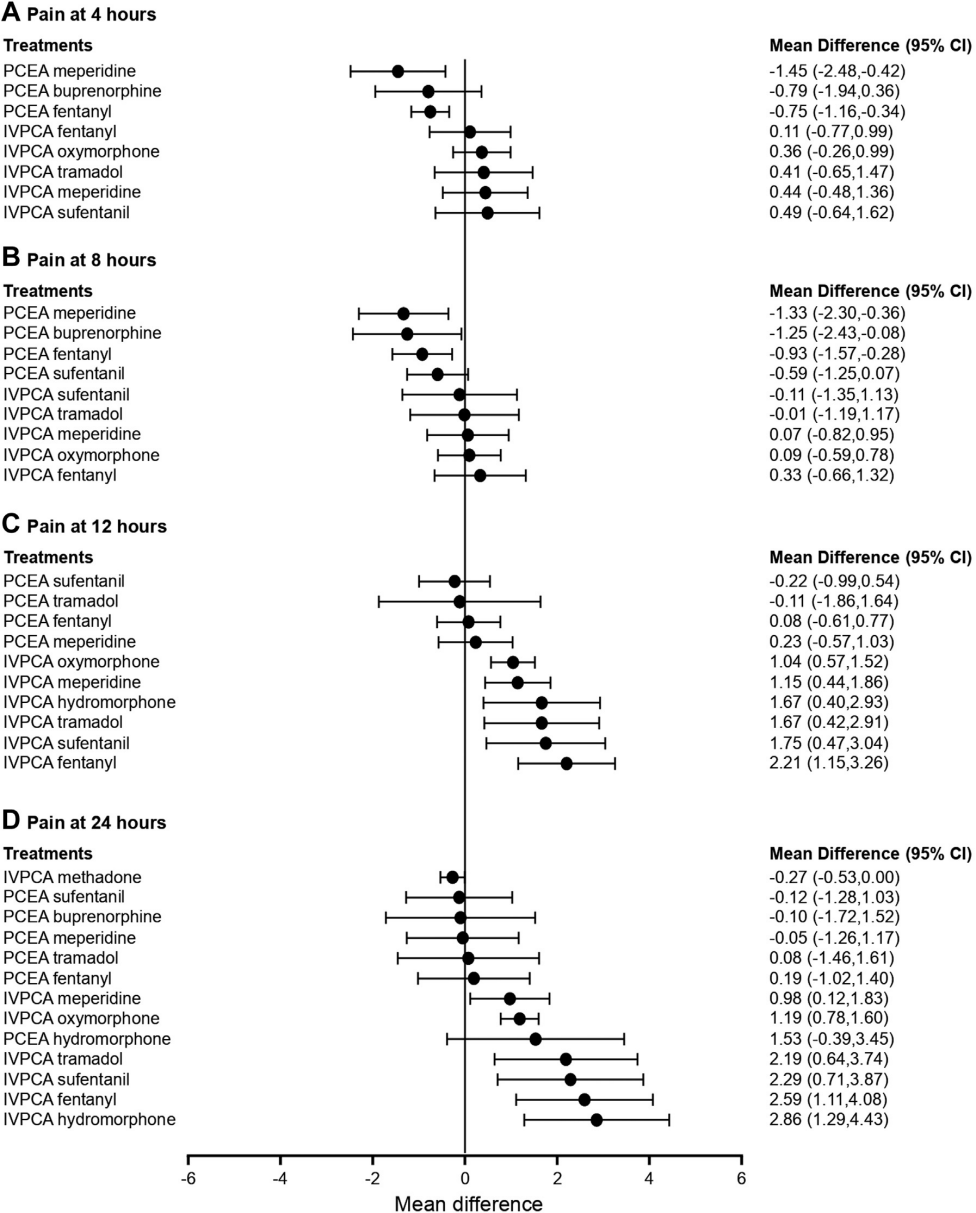
**Forest plot of pain intensity**. The forest plot depicts the effects of each treatment relative to that of IVPCA morphine on (A) pain intensity at 4 h after surgery (B) pain intensity at 8 h after surgery (C) pain intensity at 12 h after surgery, and (D) pain intensity at 24 h after surgery. The black solid circles represent the point estimates, and the error bars represent 95% confidence interval. CI, confidence interval; IVPCA, intravenous patient-controlled analgesia; PCEA, patient-controlled epidural analgesia.

Eleven studies reported the pain intensity at 4 h after surgery and were included in the pairwise meta-analysis. Pain intensity was measured using a 10-cm or 100-mm visual analogue scale (VAS) with a score of 0 cm or 0 mm indicating no pain and 10 cm or 100 mm indicating the worst pain imaginable. The 100-mm VAS scale was converted to a 10-cm VAS scale by dividing the scores by 10. Of 13 different comparisons that were conducted, 9 were performed in a single study, and 4 involved at least two studies. No statistically significant heterogeneity was observed in the 4 comparisons that involved multiple studies (Supplementary Fig. S2). A network meta-analysis was conducted and consisted of 9 treatments (Supplementary Fig. S3A). The effects of each treatment on pain intensity relative to that of IVPCA morphine are shown in Fig. 2A, and the relative effects of all the competing treatments are summarised in Supplementary Table S3. Direct comparisons are displayed along with the pooled overall treatment effects in the network meta-analysis forest plot (Supplementary Fig. S4A). The cumulative ranking probability of each treatment is shown in Supplementary Fig. S5A. The SUCRA and NE values of each treatment are presented in Table 2. The MD in pain intensity at 4 h obtained from the network evidence ranged from −1.45 (95% confidence interval [CI] [−2.48, −0.42]) for the highest ranked treatment (PCEA meperidine) to 0.49 (95% CI [−0.64, 1.62]) for the lowest ranked treatment (IVPCA sufentanil) compared with IVPCA morphine. PCEA fentanyl and PCEA meperidine resulted in significantly lower pain intensities than IVPCA fentanyl (MD, −0.86; 95% CI [−1.66, −0.07]) and IVPCA meperidine (MD, −1.89; 95% CI [−2.72, −1.07]). The three PCEA treatments ranked highly, followed by the IVPCA treatments. The NE was relatively low for PCEA meperidine (0.31) and PCEA fentanyl (0.44), suggesting a robust treatment ranking.

**Table 2:** The surface under the cumulative ranking curve (SUCRA) and normalised entropy (NE) of each treatment for pain intensity.

Treatments	SUCRA	NE
**Pain intensity at 4 h after surgery**		
PCEA meperidine	0.97	0.31
PCEA fentanyl	0.81	0.44
PCEA buprenorphine	0.79	0.71
IVPCA morphine	0.49	0.72
IVPCA fentanyl	0.46	0.79
IVPCA oxymorphone	0.27	0.83
IVPCA tramadol	0.26	0.79
IVPCA meperidine	0.24	0.80
IVPCA sufentanil	0.21	0.79
**Pain intensity at 8 h after surgery**		
PCEA meperidine	0.92	0.51
PCEA buprenorphine	0.88	0.61
PCEA fentanyl	0.81	0.46
PCEA sufentanil	0.63	0.51
IVPCA sufentanil	0.42	0.90
IVPCA tramadol	0.34	0.85
IVPCA morphine	0.32	0.81
IVPCA meperidine	0.27	0.81
IVPCA oxymorphone	0.27	0.84
IVPCA fentanyl	0.14	0.64
**Pain intensity at 12 h after surgery**		
PCEA sufentanil	0.91	0.52
PCEA tramadol	0.80	0.74
IVPCA morphine	0.79	0.67
PCEA fentanyl	0.76	0.59
PCEA meperidine	0.70	0.66
IVPCA oxymorphone	0.42	0.64
IVPCA meperidine	0.39	0.60
IVPCA tramadol	0.26	0.61
IVPCA hydromorphone	0.25	0.67
IVPCA sufentanil	0.19	0.65
IVPCA fentanyl	0.03	0.26
**Pain intensity at 24 h after surgery**		
IVPCA methadone	0.86	0.67
PCEA sufentanil	0.81	0.71
PCEA buprenorphine	0.77	0.80
PCEA meperidine	0.77	0.78
IVPCA morphine	0.73	0.72
PCEA tramadol	0.71	0.84
PCEA fentanyl	0.66	0.73
IVPCA meperidine	0.43	0.70
IVPCA oxymorphone	0.38	0.67
PCEA hydromorphone	0.37	0.69
IVPCA tramadol	0.23	0.44
IVPCA sufentanil	0.19	0.51
IVPCA fentanyl	0.09	0.41
IVPCA hydromorphone	0.02	0.21

IVPCA, intravenous patient-controlled analgesia; NE, normalised entropy; PCEA, patient-controlled epidural analgesia; SUCRA, the surface under the cumulative ranking curve.

#### Pain intensity at 8 h after surgery

Twelve studies reported the pain intensity at 8 h after surgery and were included in the pairwise meta-analysis. Of 14 different comparisons that were conducted, 10 were performed in a single study, and 4 involved at least two studies. Statistically significant heterogeneity was not observed in the 4 comparisons that involved multiple studies (Supplementary Fig. S6). A network meta-analysis was conducted and consisted of 10 treatment agents (Supplementary Fig. S3B). The effects of each treatment on pain intensity relative to that of IVPCA morphine are shown in Fig. 2B, and the relative effects of all the competing treatments are summarised in Supplementary Table S4. Direct comparisons are displayed along with the pooled overall treatment effects in the network meta-analysis forest plot (Supplementary Fig. S4B). The cumulative ranking probability of each treatment is shown in Supplementary Fig. S5B. The SUCRA and NE values of each treatment are presented in Table 2. The MD in pain intensity at 8 h obtained from the network evidence ranged from −1.33 (95% CI [−2.30, −0.36]) for the highest ranked treatment (PCEA meperidine) to 0.33 (95% CI [−0.66, 1.32]) for the lowest ranked treatment (IVPCA fentanyl) compared with IVPCA morphine. PCEA fentanyl and PCEA meperidine were associated with significantly lower pain intensities than IVPCA fentanyl (MD, −1.26; 95% CI [−2.03, −0.48]) and IVPCA meperidine (MD, −1.40; 95% CI [−1.97, −0.82]). The four PCEA treatments ranked highly, followed by the IVPCA treatments. The NE was relatively low for PCEA meperidine (0.51), PCEA fentanyl (0.46), and PCEA sufentanil (0.51).

#### Pain intensity at 12 h after surgery

Fifteen studies reported the pain intensity at 12 h after surgery and were included in the pairwise meta-analysis. Of 16 different comparisons that were conducted, 12 were performed in a single study, and 4 involved at least two studies. Statistically significant heterogeneity was not observed in the 4 comparisons that involved multiple studies (Supplementary Fig. S7). A network meta-analysis was conducted and consisted of 11 treatment agents (Supplementary Fig. S3C). The effects of each treatment on pain intensity relative to that of IVPCA morphine are shown in Fig. 2C, and the relative effects of all the competing treatments are summarised in Supplementary Table S5. Direct comparisons are displayed along with the pooled overall treatment effects in the network meta-analysis forest plot (Supplementary Fig. S4C). The cumulative ranking probability of each treatment is shown in Supplementary Fig. S5C. The SUCRA and NE values of each treatment are presented in Table 2. The MD in pain intensity at 12 h based on the network evidence ranged from −0.22 (95% CI [−0.99, 0.54]) for the highest ranked treatment (PCEA sufentanil) to 2.21 (95% CI [1.15, 3.26]) for the lowest ranked treatment (IVPCA fentanyl) compared with IVPCA morphine. PCEA fentanyl, PCEA meperidine, and PCEA sufentanil resulted in significantly lower pain intensities than IVPCA fentanyl (MD, −2.12; 95% CI [−2.96, −1.29]), IVPCA meperidine (MD, −0.91; 95% CI [−1.54, −0.28]), and IVPCA sufentanil (MD, −1.98; 95% CI [−3.19, −0.77]). In general, PCEA treatments ranked higher than IVPCA treatments, with the exception that IVPCA morphine ranked third. The NE was relatively low for PCEA sufentanil (0.52), PCEA fentanyl (0.59), IVPCA meperidine (0.60), and IVPCA fentanyl (0.26).

#### Pain intensity at 24 h after surgery

Sixteen studies reported the pain intensity at 24 h after surgery and were included in the pairwise meta-analysis. Of 15 different comparisons that were conducted, 12 were performed in a single study, and 3 involved at least two studies. Statistically significant heterogeneity was not observed in the 3 comparisons that involved multiple studies (Supplementary Fig. S8). A network meta-analysis was conducted and consisted of 14 treatment agents (Supplementary Fig. S3D). The effects of each treatment on pain intensity relative to that of IVPCA morphine are shown in Fig. 2D, and the relative effects of all the competing treatments are summarised in Supplementary Table S6. Direct comparisons are displayed along with the pooled overall treatment effects in the network meta-analysis forest plot (Supplementary Fig. S4D). The cumulative ranking probability of each treatment is shown in Supplementary Fig. S5D. The SUCRA and NE values of each treatment are presented in Table 2. The MD in pain intensity at 24 h obtained from the network evidence ranged from −0.27 (95% CI [−0.53, 0.00]) for the highest ranked treatment (IVPCA methadone) to 2.86 (95% CI [1.29, 4.43]) for the lowest ranked treatment (IVPCA hydromorphone) compared with IVPCA morphine. PCEA fentanyl, PCEA meperidine, PCEA sufentanil, PCEA tramadol, and PCEA hydromorphone resulted in significantly lower pain intensity than IVPCA fentanyl (MD, −2.40; 95% CI [−3.26, −1.54]), IVPCA meperidine (MD, −1.02; 95% CI [−2.00, −0.04]), IVPCA sufentanil (MD, −2.41; 95% CI [−3.54, −1.29]), IVPCA tramadol (MD, −2.12; 95% CI [−3.60, −0.63]), and IVPCA hydromorphone (MD, −1.33; 95% CI [−2.43, −0.23]). In general, PCEA treatments ranked higher than IVPCA treatments, with the exceptions that IVPCA methadone ranked first and IVPCA morphine ranked fifth. The NE was relatively low for IVPCA tramadol (0.44), IVPCA sufentanil (0.51), IVPCA fentanyl (0.41), and IVPCA hydromorphone (0.21).

### Secondary outcomes

#### Nausea/vomiting

**Fig. 3:**
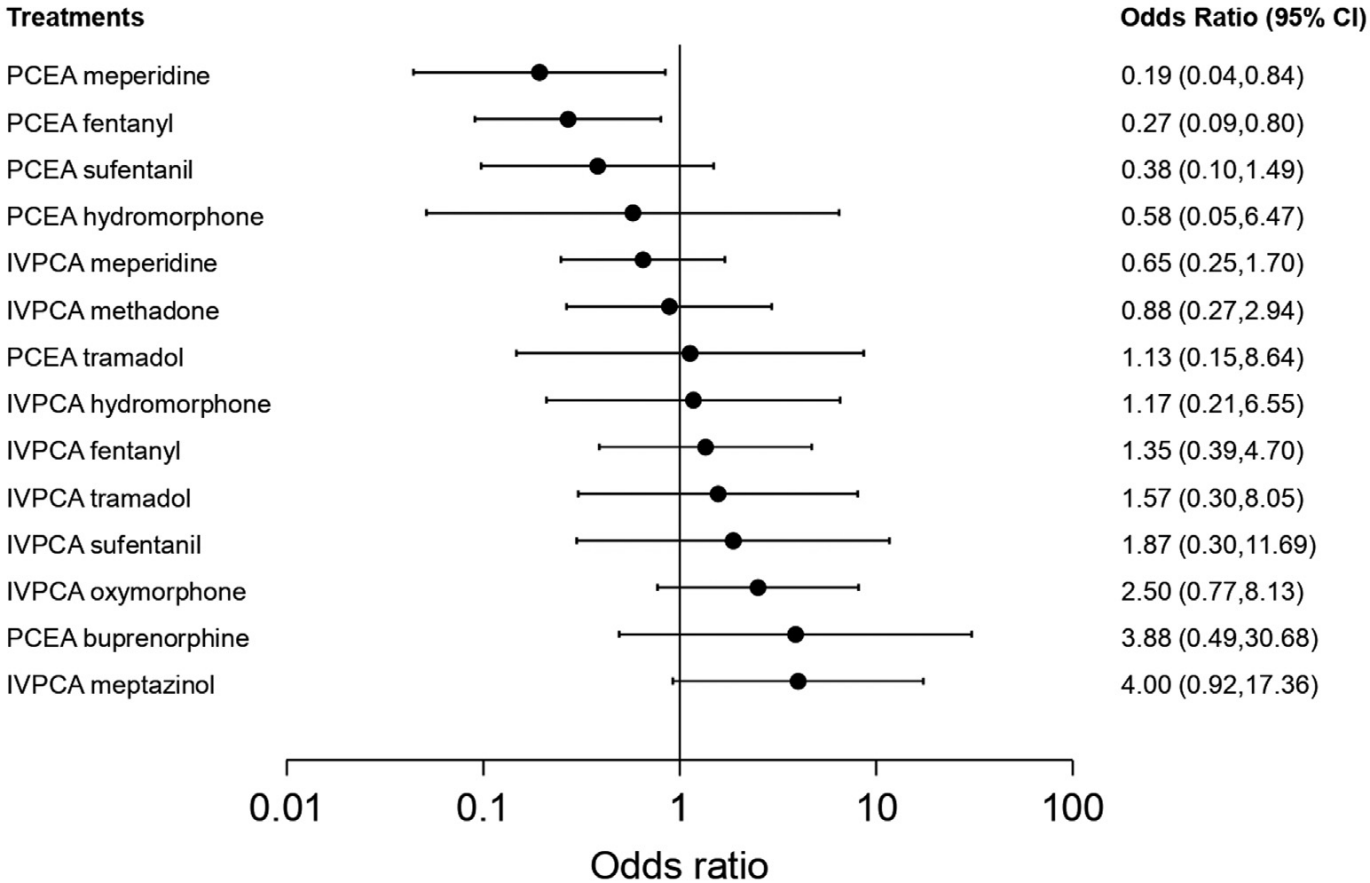
**Forest plot of nausea/vomiting**. The forest plot depicts the effects of each treatment relative to that of IVPCA morphine on nausea/vomiting. The black solid circles represent the point estimates, and the error bars represent 95% confidence interval. CI, confidence interval; IVPCA, intravenous patient-controlled analgesia; PCEA, patient-controlled epidural analgesia.

Twenty studies reported nausea/vomiting and were included in the pairwise meta-analysis. Of 17 different comparisons that were conducted, 13 were performed in a single study, and 4 involved at least two studies. Statistically significant heterogeneity was not observed in the 4 comparisons that involved multiple studies (Supplementary Fig. S9). A network meta-analysis was conducted and consisted of 15 treatment agents (Supplementary Fig. S10A). The effects of each treatment on nausea/vomiting relative to that of IVPCA morphine are shown in Fig. 3, and the relative effects of all the competing treatments are summarised in Supplementary Table S7. Direct comparisons are displayed along with the pooled overall treatment effects in the network meta-analysis forest plot (Supplementary Fig. S11A). The cumulative ranking probability of each treatment is shown in Supplementary Fig. S12A. The SUCRA and NE values of each treatment are presented in Table 3. The OR of nausea/vomiting obtained from the network evidence ranged from 0.19 (95% CI [0.04, 0.84]) for the highest ranked treatment (PCEA meperidine) to 4.00 (95% CI [0.92, 17.36]) for the lowest ranked treatment (IVPCA meptazinol) compared with IVPCA morphine. IVPCA fentanyl was associated with a significantly higher odds of developing nausea/vomiting than PCEA fentanyl (OR, 5.01; 95% CI [1.83, 13.75]). In general, PCEA treatments ranked higher than IVPCA treatments, with the exception of PCEA tramadol and PCEA buprenorphine. The NE was relatively low for PCEA meperidine (0.48) and PCEA fentanyl (0.51).

**Table 3:** The surface under the cumulative ranking curve (SUCRA) and normalised entropy (NE) of each treatment for adverse effects.

Treatments	SUCRA	NE
**Nausea/vomiting**		
PCEA meperidine	0.94	0.48
PCEA fentanyl	0.89	0.51
PCEA sufentanil	0.80	0.69
PCEA hydromorphone	0.66	0.94
IVPCA meperidine	0.65	0.86
IVPCA methadone	0.55	0.94
IVPCA morphine	0.51	0.80
IVPCA hydromorphone	0.48	0.89
PCEA tramadol	0.46	0.97
IVPCA fentanyl	0.41	0.81
IVPCA tramadol	0.35	0.83
IVPCA sufentanil	0.30	0.88
IVPCA oxymorphone	0.22	0.79
PCEA buprenorphine	0.16	0.73
IVPCA meptazinol	0.13	0.68
**Pruritus**		
IVPCA methadone	0.84	0.71
PCEA tramadol	0.80	0.76
IVPCA meperidine	0.74	0.74
IVPCA oxymorphone	0.61	0.93
PCEA buprenorphine	0.58	0.88
IVPCA fentanyl	0.55	0.86
PCEA meperidine	0.35	0.86
IVPCA morphine	0.32	0.73
PCEA fentanyl	0.12	0.55
PCEA sufentanil	0.10	0.57
**Sedation/drowsiness**		
PCEA meperidine	0.88	0.61
PCEA fentanyl	0.78	0.60
IVPCA meptazinol	0.70	0.86
PCEA buprenorphine	0.69	0.82
PCEA sufentanil	0.59	0.64
PCEA tramadol	0.55	0.96
IVPCA oxymorphone	0.52	0.78
IVPCA meperidine	0.28	0.71
IVPCA morphine	0.23	0.60
IVPCA fentanyl	0.15	0.66
IVPCA methadone	0.14	0.59

IVPCA, intravenous patient-controlled analgesia; NE, normalised entropy; PCEA, patient-controlled epidural analgesia; SUCRA, the surface under the cumulative ranking curve.

#### Pruritus

**Fig. 4:**
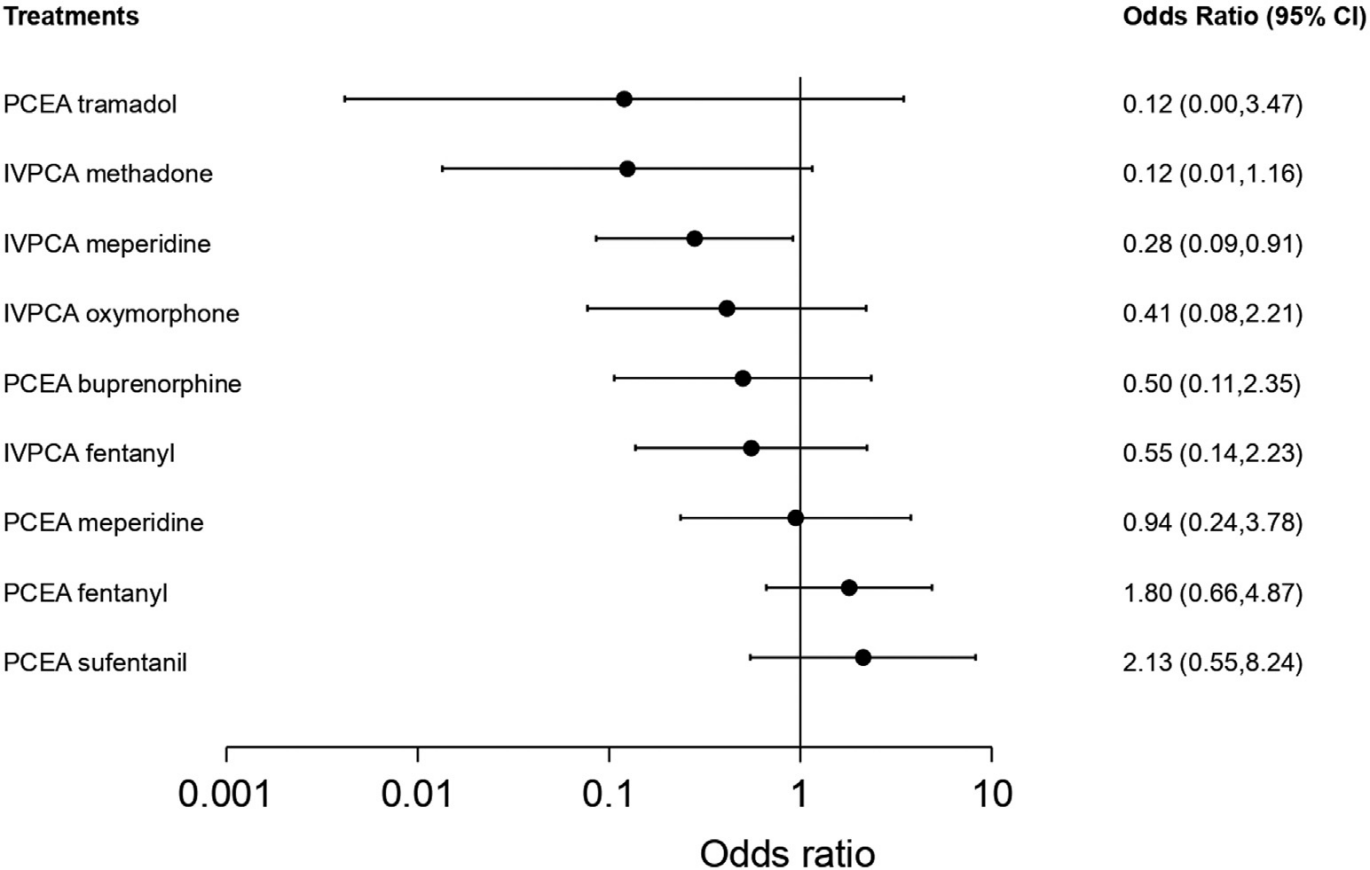
**Forest plot of pruritus**. The forest plot depicts the effects of each treatment relative to that of IVPCA morphine on pruritus. The black solid circles represent the point estimates, and the error bars represent 95% confidence interval. CI, confidence interval; IVPCA, intravenous patient-controlled analgesia; PCEA, patient-controlled epidural analgesia.

Seventeen studies reported pruritus and were included in the pairwise meta-analysis. Of 13 different comparisons that were conducted, 8 were performed in a single study, and 5 involved at least two studies. Statistically significant heterogeneity was not observed in the 5 comparisons that involved multiple studies (Supplementary Fig. S13). A network meta-analysis was conducted and consisted of 10 treatment agents (Supplementary Fig. S10B). The effects of each treatment on pruritus relative to that of IVPCA morphine are shown in Fig. 4, and the relative effects of all the competing treatments are summarised in Supplementary Table S8. Direct comparisons are displayed along with the pooled overall treatment effects in the network meta-analysis forest plot (Supplementary Fig. S11B). The cumulative ranking probability of each treatment is shown in Supplementary Fig. S12B. The SUCRA and NE values of each treatment are presented in Table 3. The OR of pruritus based on the network evidence ranged from 0.12 (95% CI [0.00, 3.47]) for the highest ranked treatment (PCEA tramadol) to 2.13 (95% CI [0.55, 8.24]) for the lowest ranked treatment (PCEA sufentanil) compared with IVPCA morphine. IVPCA fentanyl was associated with a significantly lower odds of developing pruritus than PCEA fentanyl (OR, 0.31; 95% CI [0.10, 0.92]). The NE was relatively low for PCEA fentanyl (0.55) and PCEA sufentanil (0.57).

#### Sedation/drowsiness

**Fig. 5:**
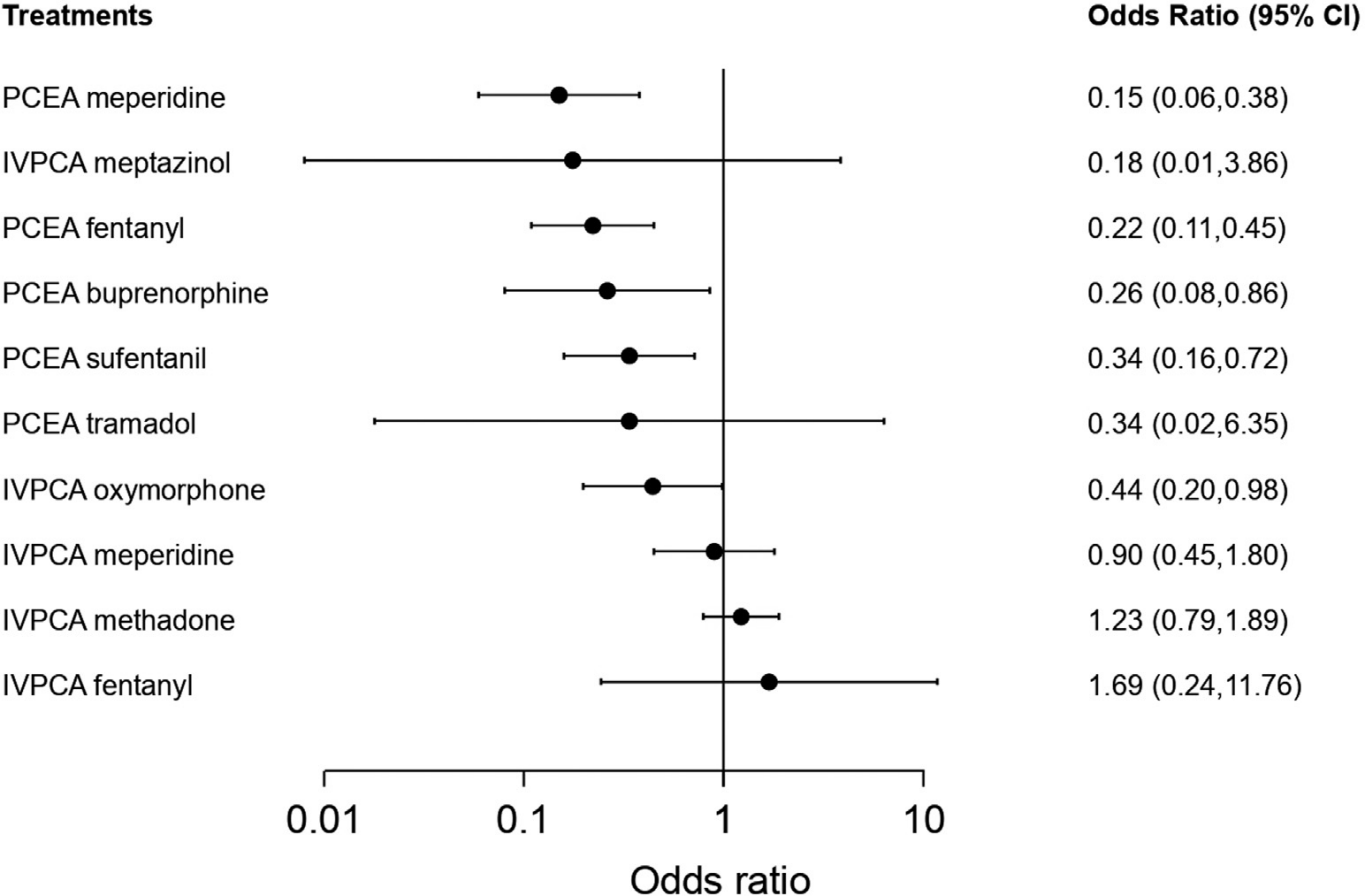
**Forest plot of sedation/drowsiness**. The forest plot depicts the effects of each treatment relative to that of IVPCA morphine on sedation/drowsiness. The black solid circles represent the point estimates, and the error bars represent 95% confidence interval. CI, confidence interval; IVPCA, intravenous patient-controlled analgesia; PCEA, patient-controlled epidural analgesia.

Sixteen studies reported sedation/drowsiness and were included in the pairwise meta-analysis. Of the 8 different comparisons that were conducted, 7 were performed in a single study, and 1 involved at least two studies. Statistically significant heterogeneity was not observed in the comparison that involved multiple studies (Supplementary Fig. S14). A network meta-analysis was conducted and consisted of 11 treatment agents (Supplementary Fig. S10C). The effects of each treatment on sedation/drowsiness relative to that of IVPCA morphine are shown in Fig. 5, and the relative effects of all the competing treatments are summarised in Supplementary Table S9. Direct comparisons are displayed along with the pooled overall treatment effects in the network meta-analysis forest plot (Supplementary Fig. S11C). The cumulative ranking probability of each treatment is shown in Supplementary Fig. S12C. The SUCRA and NE values of each treatment are presented in Table 3. The OR of sedation/drowsiness obtained from the network evidence ranged from 0.15 (95% CI [0.06, 0.38]) for the highest ranked treatment (PCEA meperidine) to 1.69 (95% CI [0.24, 11.76]) for the lowest ranked treatment (IVPCA fentanyl) compared with IVPCA morphine. IVPCA fentanyl and IVPCA meperidine were associated with significantly higher odds of developing sedation/drowsiness than PCEA fentanyl (OR, 7.63; 95% CI [1.25, 46.48]) and PCEA meperidine (OR, 5.98; 95% CI [2.51, 14.29]). In general, the PCEA treatments ranked higher than the IVPCA treatments. The NE was relatively low for PCEA meperidine (0.61), PCEA fentanyl (0.60), IVPC morphine (0.60), and IVPCA methadone (0.59).

#### Respiratory depression

Of the 23 included studies, respiratory depression or ventilatory frequency was assessed and reported in 13 studies. Respiratory depression was defined as a respiratory rate less than 10 to 12 breaths per minute in most of the studies. No respiratory depression was observed. In the study by Cooper et al., the respiratory rate ranged from 14 to 18 breaths per minute in the PCEA fentanyl group and 12 to 20 breaths per minute in the IVPCA fentanyl group.^11^

### Sensitivity analysis

The sensitivity analysis is presented in Supplementary Fig. S15. The MD in pain intensity at 4 h obtained from the network evidence ranged from −1.45 (95% CI [−2.48, −0.42]) for PCEA meperidine to 0.49 (95% CI [−0.64, 1.62]) for IVPCA sufentanil compared with IVPCA morphine. The MD in pain intensity at 8 h based on the network evidence ranged from −1.33 (95% CI [−2.30, −0.36]) for PCEA meperidine to 0.33 (95% CI [−0.66, 1.32]) for IVPCA fentanyl compared with IVPCA morphine. The MD in pain intensity at 12 h obtained from the network evidence ranged from −0.07 (95% CI [−0.86, 0.71]) for PCEA fentanyl to 2.05 (95% CI [0.94, 3.17]) for IVPCA fentanyl compared with IVPCA morphine. In terms of pain intensity at 24 h, the meta-regression model revealed no statistically significant analgesic effects of the addition of local anaesthetics (coefficient, −0.56; *p*-value, 0.723).

### Simultaneous ranking of the treatments in terms of analgesic efficacy and adverse effects

The efficacy and adverse effects of the treatments are summarised in Supplementary Fig. S16. In terms of analgesic efficacy and nausea/vomiting, PCEA treatments (e.g., PCEA meperidine, PCEA fentanyl, and PCEA sufentanil) lie at the upper right corner and are more effective in pain relief with lower odds of nausea/vomiting. In terms of analgesic efficacy and sedation/drowsiness, PCEA treatments (PCEA meperidine, PCEA fentanyl, PCEA sufentanil, PCEA buprenorphine, and PCEA tramadol) lie at the upper right corner and are more effective in pain relief with lower odds of sedation/drowsiness. In terms of analgesic efficacy and pruritus, PCEA treatments generally have better analgesic efficacy. The likelihood of developing pruritus with PCEA treatments appears to be similar to that with IVPCA treatments, except for PCEA fentanyl and PCEA sufentanil, which yield higher odds of developing pruritus.

### Inconsistency

The assessment of the network inconsistency is presented in Supplementary Table S10. Statistically significant inconsistency was not observed for most of the outcomes. However, for pruritus, side-splitting models revealed statistically significant inconsistency between the direct and indirect evidence for PCEA sufentanil versus IVPCA morphine (P = 0.016) and for PCEA sufentanil versus PCEA fentanyl (P = 0.016). A deeper analyses indicated that this inconsistency was attributed to the heterogeneity in the effects on pruritus between IVPCA morphine, PCEA fentanyl, and PCEA sufentanil reported by Cooper et al.,^47^ Grass et al.,^13^ and Cohen et al.^41^ The incidence of pruritus was significantly higher in the PCEA sufentanil group than in the IVPCA morphine group in the study by Grass et al. (57% versus 12%). However, the incidence of pruritus in the PCEA sufentanil group was much lower (9.6%) in the study by Cohen et al., and the PCEA fentanyl group also showed a similar level of pruritus incidence (13.6%). In the study by Cooper et al., the overall incidences of pruritus were very similar in the PCEA fentanyl and IVPCA morphine groups (63% versus 62%). Consequently, these 3 studies reported inconsistent incidences of pruritus among the 3 treatment groups. However, Cooper et al. found that pruritus occurred sooner in the PCEA fentanyl group than in the IVPCA morphine group (54% versus 24% at 4–8 h and 55% versus 26% at 8–12 h). The overall incidence of pruritus in the study by Cooper et al. was extracted and analysed instead of the values reported at the specific time points. Most studies did not clearly describe when pruritus was assessed, and these discrepancies in the timing of the assessment may be the cause of heterogeneity.

### Publication bias and GRADE results

No statistically significant publication bias was detected for any of the outcomes using comparison-adjusted funnel plots and Egger's tests (Supplementary Fig. S17 and Supplementary Fig. S18). The GRADE results of all the outcomes are provided in Supplementary Table S11.

## Discussion

The main finding of the present study is that opioids delivered via PCEA generally provide better pain relief than IVPCA. In addition, PCEA treatments yield lower odds of developing nausea, vomiting, sedation or drowsiness than IVPCA treatments. However, PCEA treatments, especially PCEA fentanyl and PCEA sufentanil, are prone to causing pruritus. The same agents, such as fentanyl, sufentanil, meperidine, tramadol and hydromorphone, resulted in significantly better pain relief when delivered via PCEA than when delivered via IVPCA. Fentanyl delivered via PCEA had significantly lower odds of nausea or vomiting than when delivered via IVPCA. In contrast, there was no statistically significant difference in nausea or vomiting when sufentanil, meperidine, tramadol and hydromorphone were delivered via IVPCA or PCEA. Fentanyl and meperidine delivered via PCEA resulted in significantly lower odds of sedation or drowsiness than when delivered via IVPCA. Fentanyl delivered via PCEA had significantly higher odds of pruritus than when delivered via IVPCA. In contrast, there was no statistically significant difference in pruritus whether meperidine was delivered via IVPCA or PCEA. The sensitivity analyses showed that the better analgesic efficacy of PCEA treatments was not significantly driven by the addition of local anaesthetics.

Opioid receptors are abundant in the central nervous system, including the periaqueductal grey, rostral ventral medulla, and the substantia gelatinosa of the dorsal horn. Activation of the opioid receptors at the periaqueductal grey increases the neuronal signal through the nucleus raphe magnus, which in turn stimulates 5-hydroxytryptamine and enkephalin-containing neurons that connect with the substantia gelatinosa of the dorsal horn. This leads to the activation of the descending inhibition pathway, modulates nociceptive transmission, and reduces pain sensation.^51^ In addition, local spinal mechanisms also contribute to the analgesic effects of opioids. Fentanyl administered epidurally as a bolus acts through a local spinal mechanism.^52^ Activation of the opioid receptors at the substantia gelatinosa inhibits the release of glutamate and substance P from the primary afferent neuron and therefore reduces pain transmission.^53^ Common opioid-induced adverse effects include but are not limited to nausea and vomiting, pruritus, sedation, and respiratory depression. The mechanisms underlying opioid-induced nausea and vomiting include direct stimulation of the chemoreceptor trigger zone, increased vestibular sensitivity, and delayed gastric emptying.^54^ Systemic and neuraxial opioid-induced pruritus may be mediated by several complex mechanisms involving both peripheral and central pathways. Compelling evidence suggests that neuraxial opioid-induced pruritus may be mediated by a spinal mechanism that involves neuronal disinhibition.^55^ Opioids cause respiratory depression by direct action on brainstem respiratory centres. Nociceptive pathways also modulate arousal, and opioid-induced sedation may be due to the inhibition of periaqueductal grey GABAergic neurons, promoting nonrapid-eye-movement sleep and reduced electrocortical activity. In addition, inhibition of brainstem cholinergic neurons also contributes to opioid-induced sedation.^56^ Taken together, these findings show that opioids administered epidurally elicit their analgesic effects primarily by the local spinal mechanism and, to a lesser extent, the central descending inhibition pathway due to systemic redistribution secondary to limited vascular uptake. The local spinal mechanism inhibits pain transmission at the level of primary afferent nerve fibres, together with the central descending inhibition pathway, which may explain the observations in the present study that PCEA treatments generally provided better pain relief than IVPCA treatments. Moreover, compared with IVPCA treatments, PCEA treatments result in little, if any, systemic distribution of opioids that circulate to the effect sites of the brain responsible for opioid-induced adverse effects. This supports the observations in the present study that PCEA treatments pose lower odds of developing nausea, vomiting, sedation or drowsiness.

Meperidine is a unique opioid with well-known local anaesthetic properties. It has been shown to block the voltage-gated sodium and potassium channels, and therefore inhibits the generation of action potentials in spinal dorsal horn neurons.^57^ This unique characteristic, in addition to its agonistic effects on opioid receptors, may underlie the observation that PCEA meperidine elicits excellent pain relief. However, meperidine remains potentially harmful to infants. Meperidine carries a high risk of neonatal respiratory depression due to the long half-lives of 13 and 65 h for meperidine and its active metabolite normeperidine, respectively.^58^^,^^59^ In addition, normeperidine is known to lower seizure thresholds, especially in susceptible patients. The included studies of the present study that investigated the effects of meperidine either administered intravenously or epidurally were all published before 2000. In fact, The American Pain Society and Institute for Safe Medication Practices do not recommend the routine use of meperidine for acute pain management.^60^^,^^61^ Consequently, although the drug ranked highly in terms of analgesia, with relatively low odds of nausea/vomiting and sedation/drowsiness, we do not recommend the routine use of PCEA meperidine for post-caesarean section analgesia. Further research is warranted to investigate the efficacy and safety of PCEA meperidine.

The Enhanced Recovery After Surgery (ERAS) Society recommends that a multimodal analgesic approach be used for post-caesarean delivery, including intrathecal or epidural morphine, transversus abdominis plane block, local analgesia infiltration, and oral nonsteroidal anti-inflammatory drugs (NSAIDs).^62^ In contrast, a recent procedure-specific postoperative pain management (PROSPECT) guideline recommends against the use of PCEA for post-caesarean delivery analgesia due to limited procedure-specific evidence and concerns of side-effects.^63^ The duration of the effect of intrathecal morphine and epidural morphine was reported to be 10–40 h^64^ and 5–30 h,^65^ respectively. The incidence of common adverse effects was similar.^66^ Although recommended by ERAS and PROSPECT guidelines, the use of NSAIDs for post-caesarean delivery analgesia remain controversial due to their effects of causing a low level of amniotic fluid, premature closure of ductus arteriosus, platelet dysfunction, uterine atony, and increased cardiovascular and thrombotic events.^67^^,^^68^ In contrast, PCEA has several advantages that are worth highlighting. PCEA allows mothers to fine-tune the amount of pain relief by simply pushing a button, and provides analgesia for postoperative pain that may last for several days, at which time the effects of single-shot intrathecal or epidural opioid have worn off. In addition, local anaesthetic, if used, is present at a low concentration in PCEA, which does not cause motor blockade and allows ambulation by mothers. PCEA may not be the mainstay treatment for post-caesarean delivery pain relief, and its use remains feasible.

The present study has some limitations. First, although the demand doses of PCA are clinically relevant, it is difficult to ensure the equianalgesic effects among all treatments. Second, background continuous infusion of opioids was implemented in some included studies and may have influenced the outcomes of interest. Third, data extraction is a great challenge when the results are presented graphically without useful data. However, the exclusion of studies that present results only graphically may lead to a biased conclusion. In the present study, we utilised WebPlotDigitizer to overcome this challenge. Although having been validated, the data extracted from graphs may still have been slightly different from the real data should the latter have been reported.

In conclusion, considering the analgesic efficacy, opioid-induced nausea, vomiting, and sedation, and the well-being of breastfed infants, PCEA fentanyl and PCEA sufentanil may be the treatments of choice for post-caesarean section analgesia. However, these treatments confer higher odds of developing pruritus. Therefore, shared decision making with parturients is strongly encouraged in order to achieve pain relief while minimising any discomfort.

## Contributors

CYC: Conceptualization and study design, acquisition of data, data analysis and interpretation, writing of the first draft of the paper; YKT: Data analysis and interpretation, editing and revising the draft for important intellectual content; MCK: Data analysis and interpretation, editing and revising the draft for important intellectual content; PCS: Conceptualization and study design, data analysis and interpretation; IMS: Conceptualization and study design, data analysis and interpretation; HYL: Editing and revising the draft for important intellectual content; YJC: Acquisition of data, data analysis and interpretation; MYU: Acquisition of data, data analysis and interpretation, visualization; CHC: Acquisition of data, data analysis and interpretation; CTC: Conceptualization and study design, editing and revising the draft for important intellectual content. All authors have accessed and verified the underlying data, and have read and agreed to the published version of the manuscript.

## Data sharing statement

The data that support the findings of this study are available from the corresponding author upon reasonable request.

## Declaration of interests

All authors declare no competing interests.
