# Supplementary material for: Effects of opioids administered via intravenous or epidural patient-controlled analgesia after caesarean section: A network meta-analysis of randomised controlled trials
Source: eClinicalMedicine. 2022 Dec 24;56:101787. doi: 10.1016/j.eclinm.2022.101787 (PMC9800204; doi:10.1016/j.eclinm.2022.101787)
Supplement: Supplementary materials [file mmc1.pdf]

Supplementary Materials

Effects of opioids administered via intravenous or epidural patient-controlled analgesia after caesarean section: a network meta-analysis of randomised controlled trials

Chun-Yu Chang, Yu-Kang Tu, Ming-Chang Kao, Ping-Cheng Shih, I-Min Su, Han-Yu Lin, Yung-Jiun Chien, Meng-Yu Wu, Chih-Hao Chen, Chu-Ting Chen

Contents

Supplementary Materials ... 1

Contents ... 2

Supplementary Table S1. Detailed search queries ... 4

Supplementary Table S2. Opioid consumption ... 7

Supplementary Figure S1. Risk of bias summary and graph ... 11

Supplementary Figure S2. Forest plots from pairwise comparisons of treatment agents on pain intensity at 4 hours after surgery ... 12

Supplementary Figure S3. Network treatment comparisons for studies investigating the effects of opioids on pain intensity ... 15

Supplementary Table S3. Comparative efficacy of treatments on pain intensity at 4 hours after surgery ... 16

Supplementary Figure S4. Network meta-analysis forest plots of pain intensity ... 18

Supplementary Figure S5. Cumulative ranking probability of the effects of each treatment on pain intensity ... 20

Supplementary Figure S6. Forest plots from pairwise comparisons of treatment agents on pain intensity at 8 hours after surgery ... 21

Supplementary Table S4. Comparative efficacy of treatments on pain intensity at 8 hours after surgery ... 22

Supplementary Figure S7. Forest plots from pairwise comparisons of treatment agents on pain intensity at 12 hours after surgery ... 23

Supplementary Table S5. Comparative efficacy of treatments on pain intensity at 12 hours after surgery ... 24

Supplementary Figure S8. Forest plots from pairwise comparisons of treatment agents on pain intensity at 24 hours after surgery ... 25

Supplementary Table S6. Comparative efficacy of treatments on pain intensity at 24 hours after surgery ... 26

Supplementary Figure S9. Forest plots from pairwise comparisons of treatment agents on nausea/vomiting ... 28

Supplementary Figure S10. Network treatment comparisons for studies investigating the effects of opioids on adverse effects ... 30

Supplementary Table S7. Comparative efficacy of treatments on nausea/vomiting ... 31

Supplementary Figure S11. Network meta-analysis forest plots of adverse effects ... 34

Supplementary Figure S12. Cumulative ranking probability of the effects of each treatment on adverse effects ... 36

Supplementary Figure S13. Forest plots from pairwise comparisons of treatment agents on pruritus ... 37

Supplementary Table S8. Comparative efficacy of treatments on pruritus ... 38

Supplementary Figure S14. Forest plots from pairwise comparisons of treatment agents on sedation/drowsiness ... 39

Supplementary Table S9. Comparative efficacy of treatments on sedation/drowsiness ... 40

Supplementary Figure S15. Sensitivity analysis of pain intensity ... 42

Supplementary Figure S16. Clustered ranking plots....49

Supplementary Table S10. Assessment of network inconsistency....50

Supplementary Figure S17. Comparison-adjusted funnel plots....57

Supplementary Figure S18. Egger's test....61

Supplementary Table S11. GRADE approach for rating the quality of evidence....62

References....95

Supplementary Table S1. Detailed search queries

Database	Query	Results
PubMed	("analgesia, obstetrical"[MeSH Terms] OR "analgesia, obstetrical/adverse effects"[MeSH Terms] OR "analgesia, obstetrical/statistics and numerical data"[MeSH Terms] OR "analgesia, obstetrical/therapeutic use"[MeSH Terms] OR "analgesia, obstetrical/therapy"[MeSH Terms] OR "analgesia, patient controlled"[MeSH Terms] OR "analgesia, patient controlled/adverse effects"[MeSH Terms] OR "analgesia, patient controlled/statistics and numerical data"[MeSH Terms] OR "analgesia, patient controlled/therapeutic use"[MeSH Terms] OR "analgesia, patient controlled/therapy"[MeSH Terms] OR "analgesia, epidural"[MeSH Terms] OR "analgesia, epidural/adverse effects"[MeSH Terms] OR "analgesia, epidural/statistics and numerical data"[MeSH Terms] OR "analgesia, epidural/therapeutic use"[MeSH Terms] OR "analgesia, epidural/therapy"[MeSH Terms] OR ("patient-controlled analgesia"[Title/Abstract] OR "patient-controlled analgesia"[Title/Abstract] OR "intravenous patient-controlled analgesia"[Title/Abstract] OR "intravenous patient-controlled analgesia"[Title/Abstract] OR "patient-controlled intravenous analgesia"[Title/Abstract] OR "patient-controlled intravenous analgesia"[Title/Abstract] OR "patient-controlled epidural analgesia"[Title/Abstract] OR "patient-controlled epidural analgesia"[Title/Abstract] OR "epidural patient-controlled analgesia"[Title/Abstract] OR "patient-controlled epidural analgesia"[Title/Abstract] OR "epidural analgesia"[Title/Abstract] OR "epidural"[Title/Abstract]) OR ("patient-controlled analgesia"[All Fields] OR "patient-controlled analgesia"[All Fields] OR "intravenous patient-controlled analgesia"[All Fields] OR "intravenous patient-controlled analgesia"[All Fields] OR "patient-controlled intravenous analgesia"[All Fields] OR "patient-controlled intravenous analgesia"[All Fields] OR "patient-controlled epidural analgesia"[All Fields] OR "patient-controlled epidural analgesia"[All Fields] OR "epidural patient-controlled analgesia"[All Fields] OR "patient-controlled epidural analgesia"[All Fields] OR "epidural analgesia"[All Fields] OR "epidural"[All Fields])) AND ("Cesarean Section"[MeSH Terms] OR ("Cesarean Section"[Title/Abstract] OR "cesarean sections"[Title/Abstract] OR "abdominal delivery"[Title/Abstract] OR "abdominal deliveries"[Title/Abstract] OR "caesarean section"[Title/Abstract] OR "caesarean sections"[Title/Abstract] OR "c-section"[Title/Abstract] OR "c-sections"[Title/Abstract] OR "c-section"[Title/Abstract] OR "c-sections"[Title/Abstract] OR "postcesarean section"[Title/Abstract] OR "postcaesarean section"[Title/Abstract]) OR ("Cesarean Section"[All Fields] OR "cesarean sections"[All Fields] OR "abdominal delivery"[All Fields] OR "abdominal deliveries"[All Fields] OR "caesarean section"[All Fields] OR "caesarean sections"[All Fields] OR "c-section"[All Fields] OR "c-sections"[All Fields] OR "c-section"[All Fields] OR "c-sections"[All Fields] OR "postcesarean section"[All Fields] OR "postcaesarean section"[All Fields]))	4908
Embase	('cesarean section'/exp OR 'birth, abdominal operation' OR 'birth, caesarean' OR 'caesarean birth' OR 'caesarean section' OR 'caesarian birth' OR 'caesarian section' OR 'cesarean delivery' OR 'cesarean section' OR 'cesarian section' OR 'elective repeat cesarean section'	2840

	OR 'fetectomy' OR 'repeated cesarotomy' OR 'sectio caesarea') AND ('patient controlled analgesia'/exp OR 'analgesia, patient controlled' OR 'analgesia, patient-controlled' OR 'patient controlled anaesthesia' OR 'patient controlled analgesia' OR 'patient controlled anesthesia' OR 'patient-controlled analgesia' OR 'self anaesthesia' OR 'self anesthesia' OR 'intravenous patient controlled analgesia'/exp OR 'epidural analgesia'/exp OR 'analgesia, epidural' OR 'epidural analgesia' OR 'extradural analgesia' OR 'peridural analgesia' OR 'patient controlled epidural analgesia'/exp)	
Web of Science	TS=(("patient-controlled analgesia" OR "patient controlled analgesia" OR "intravenous patient-controlled analgesia" OR "intravenous patient controlled analgesia" OR "patient-controlled intravenous analgesia" OR "patient controlled intravenous analgesia" OR "patient-controlled epidural analgesia" OR "patient controlled epidural analgesia" OR "epidural patient-controlled analgesia" OR "patient controlled epidural analgesia" OR "epidural analgesia" OR "epidural") AND ("cesarean section" OR "cesarean sections" OR "abdominal delivery" OR "abdominal deliveries" OR "caesarean section" OR "caesarean sections" OR "c-section" OR "c-sections" OR "c section" OR "c sections" OR "postcesarean section" OR "postcaesarean section") AND (random* OR randomized OR "randomized control" OR "randomized controlled" OR randomised OR "randomised control" OR "randomised controlled"))	919
Scopus	TITLE-ABS-KEY(("patient-controlled analgesia" OR "patient controlled analgesia" OR "intravenous patient-controlled analgesia" OR "intravenous patient controlled analgesia" OR "patient-controlled intravenous analgesia" OR "patient controlled intravenous analgesia" OR "patient-controlled epidural analgesia" OR "patient controlled epidural analgesia" OR "epidural patient-controlled analgesia" OR "patient controlled epidural analgesia" OR "epidural analgesia" OR "epidural") AND ("cesarean section" OR "cesarean sections" OR "abdominal delivery" OR "abdominal deliveries" OR "caesarean section" OR "caesarean sections" OR "c-section" OR "c-sections" OR "c section" OR "c sections" OR "postcesarean section" OR "postcaesarean section") AND (random* OR randomized OR "randomized control" OR "randomized controlled" OR randomised OR "randomised control" OR "randomised controlled"))	1516
Cochrane Library	ID Search Hits<br>#1 MeSH descriptor: [Analgesia, Obstetrical] explode all trees 992<br>#2 MeSH descriptor: [Analgesia, Obstetrical] explode all trees and with qualifier(s): [adverse effects - AE, statistics & numerical data - SN] 213<br>#3 MeSH descriptor: [Analgesia, Patient-Controlled] explode all trees 2139<br>#4 MeSH descriptor: [Analgesia, Patient-Controlled] explode all trees and with qualifier(s): [adverse effects - AE, statistics & numerical data - SN] 235<br>#5 MeSH descriptor: [Analgesia, Epidural] explode all trees 2096<br>#6 MeSH descriptor: [Analgesia, Epidural] explode all trees and with qualifier(s): [adverse effects - AE, statistics & numerical data	886

- SN] 378
#7 "patient-controlled analgesia" 4925
#8 "patient controlled analgesia" 4925
#9 "intravenous patient-controlled analgesia" 793
#10 "patient controlled intravenous analgesia" 404
#11 "patient-controlled epidural analgesia" 753
#12 "patient controlled epidural analgesia" 753
#13 "epidural patient-controlled analgesia" 78
#14 "patient controlled epidural analgesia" 753
#15 "epidural analgesia" 4502
#16 MeSH descriptor: [Cesarean Section] explode all trees 3355
#17 "cesarean section" 12960
#18 "cesarean sections" 1170
#19 "abdominal delivery" 39
#20 "abdominal deliveries" 3
#21 "caesarean section" 12961
#22 "caesarean sections" 1170
#23 "c-section" 5835
#24 "c-sections" 5550
#25 "c section" 5835
#26 "c sections" 5550
#27 "postcesarean section" 158
#28 "postcaesarean section" 73
#29 {OR #1-#15} 10164
#30 {OR #16-#28} 18412
#31 #29 AND #30 1063

Among 1063 records, there are 163 Cochrane reviews, 4 Cochrane protocols, 886 trials, 1 special collection, and 9 Clinical answers. 886 trials were then exported for screening for eligibility, whereas the rest of the publication types were excluded without exporting.

Supplementary Table S2. Opioid consumption

Study	Comparisons	Opioid consumption
Wu et al., 2021[1]	• IVPCA tramadol (4 mg ml^{-1})	178 (155, 213) mL†
	• IVPCA hydromorphone (40 μg ml^{-1})	182 (154, 210) mL†
Chi et al., 2017[2]	• IVPCA sufentanil (1.5 μg ml^{-1})	4 hours: 8.60 (3.51) mL‡<br>8 hours: 14.49 (4.53) mL‡<br>12 hours: 18.40 (7.71) mL‡<br>24 hours: 42.90 (9.14) mL‡
	• IVPCA tramadol (10 mg ml^{-1})	4 hours: 9.49 (4.68) mL‡<br>8 hours: 19.50 (4.67) mL‡<br>12 hours: 27.47 (10.53) mL‡<br>24 hours: 42.77 (10.94) mL‡
Ebneshahidi et al., 2012[3]	• IVPCA morphine (0.1 mg ml^{-1})	NR
	• IVPCA methadone (0.05 mg ml^{-1})	NR
Saracoglu et al., 2010[4]	• IVPCA fentanyl (10 μg ml^{-1})	638.4 (179.10) μg‡
	• IVPCA tramadol (10 mg ml^{-1})	559.5 (207.04) mg‡
Cohen et al., 2002[5]	• IVPCA fentanyl (20 μg ml^{-1})	6568 (1678) μg‡
	• PCEA fentanyl (20 μg ml^{-1})	4484 (1100) μg‡
Cooper et al., 1999[6]	• IVPCA morphine (2 mg ml^{-1})	0-4 hours: 1.0 (0.3, 1.5) mg h^{-1}†<br>4-8 hours: 2.0 (1.1, 2.8) mg h^{-1}†<br>8-12 hours: 1.9 (1.2, 2.7) mg h^{-1}†<br>12-21 hours: 1.9 (1.1, 2.9) mg h^{-1}†
	• PCEA fentanyl (4 μg ml^{-1})	0-4 hours: 6 (0, 10) μg h^{-1}†<br>4-8 hours: 16 (9, 29) μg h^{-1}†<br>8-12 hours: 20 (10, 25) μg h^{-1}†<br>12-21 hours: 18 (11, 26) μg h^{-1}†

Kim et al., 1999[7]	• IVPCA morphine	12 hours: 37.4 (12.5) mg‡<br>24 hours: 57.3 (21.0) mg‡<br>48 hours: 78.3 (31.2) mg‡
	• IVPCA meperidine	12 hours: 283.0 (84.6) mg‡<br>24 hours: 403.3 (125.1) mg‡<br>48 hours: 526.7 (183.8) mg‡
Vercauteren et al., 1999[8]	• PCEA sufentanil (2 μg ml^{-1})	24 hours: 123.5 (47.20) μg‡
	• PCEA tramadol (10 mg ml^{-1})	24 hours: 652 (178.20) mg‡
James et al., 1997[9]	• IVPCA meptazinol (10 mg ml^{-1})	The author only reported that the consumption of drugs was similar in both groups.
	• IVPCA morphine (1 mg ml^{-1})	
Lee et al., 1997[10]	• IVPCA morphine	58.1 (23.8) mg‡
	• IVPCA meperidine	483.8 (211.5) mg‡
Ngan Kee et al., 1997[11]	• IVPCA meperidine (10 mg ml^{-1})	0-12 hours: 240 (160, 320) mg†
	• PCEA meperidine (10 mg ml^{-1})	0-12 hours: 220 (160, 310) mg†
	• IVPCA fentanyl (20 μg ml^{-1})	0-12 hours: 1040 (800, 1400) μg†
	• PCEA fentanyl (20 μg ml^{-1})	0-12 hours: 560 (480, 760) μg†
Goh et al., 1996[12]	• PCEA fentanyl (10 μg ml^{-1})	NR
	• PCEA meperidine (5 mg ml^{-1})	NR
Cooper et al., 1995[13]	• IVPCA fentanyl (4 μg ml^{-1})	0-4 hours: 11 (10) μg‡<br>4-8 hours: 59 (27) μg‡<br>8-12 hours: 43 (25) μg‡<br>12-24 hours: 38 (19) μg‡<br>0-24 hours: 40 (15) μg‡
	• PCEA fentanyl (4 μg ml^{-1})	0-4 hours: 13 (14) μg‡<br>4-8 hours: 38 (30) μg‡<br>8-12 hours: 37 (19) μg‡<br>12-24 hours: 33 (20) μg‡<br>0-24 hours: 32 (18) μg‡

Howell et al., 1995[14]	• IVPCA morphine (1 mg ml^{-1})	Mean 8-hour dose: 19 (6.6) mg[‡]<br>Mean 24-hour dose: 41 (16.1) mg[‡]
	• IVPCA fentanyl (25 μg ml^{-1})	Mean 8-hour dose: 710 (475) μg[‡]<br>Mean 24-hour dose: 1875 (1450) μg[‡]
Lee et al., 1995[15]	• IVPCA meperidine	12 hours: 171.5 (101.6) mg[‡]<br>24 hours: 252.5 (135.7) mg[‡]
	• PCEA meperidine[‡]	12 hours: 74.1 (18.3) mg[‡]<br>24 hours: 129 (24.7) mg[‡]
Grass et al., 1994[16]	• IVPCA morphine	NR
	• PCEA sufentanil	NR
Paech et al., 1994[17]	• PCEA meperidine (4 mg ml^{-1})	181 (155, 241) mg[†]
	• IVPCA meperidine (4 mg ml^{-1})	426 (380, 480) mg[†]
Cohen et al., 1993[18]	• PCEA fentanyl (2 μg ml^{-1})	Mean total dose: 1814 μg
	• PCEA sufentanil (0.8 μg ml^{-1})	Mean total dose: 702 μg
Cohen et al., 1992-A[19]	• PCEA buprenorphine (3 μg ml^{-1})	NR
	• PCEA fentanyl (3 μg ml^{-1})	NR
Cohen et al., 1992-B[20]	• PCEA buprenorphine (3 μg ml^{-1})	NR
	• PCEA fentanyl (2 μg ml^{-1})	NR
Parker et al., 1992[21]	• IVPCA hydromorphone (150 μg ml^{-1})	Mean 24-hour dose: 7.6 (2.7) mg[‡]
	• PCEA hydromorphone (75 μg ml^{-1})	Mean 24-hour dose: 2.1 (1.1) mg[‡]
Sinatra et al. 1989-A[22]	• IVPCA morphine (1.5 mg ml^{-1})	24 hours: 52.9 (17.1) mL[‡]

	• IVPCA meperidine (15 mg ml^{-1})	24 hours: 57.4 (18.5) mL‡
	• IVPCA oxymorphone (0.25 mg ml^{-1})	24 hours: 52.9 (14.8) mL‡
Sinatra et al. 1989-B[23]	• IVPCA morphine (1.5 mg ml^{-1})	12-hour dose: 51.5 (12.7) mg‡<br>24-hour dose: 87.45 (25.4) mg‡
	• IVPCA oxymorphone (0.25 mg ml^{-1})	12-hour dose: 57.3 (16.3) mg‡<br>24-hour dose: 97.45 (28.3) mg‡

†Data are presented as median (interquartile range).

‡Data are presented as mean (standard deviation).

IVPCA: intravenous patient-controlled analgesia; NR: not reported; PCEA: patient-controlled epidural analgesia

The opioid consumption refers to total accumulative consumption if not otherwise specified.

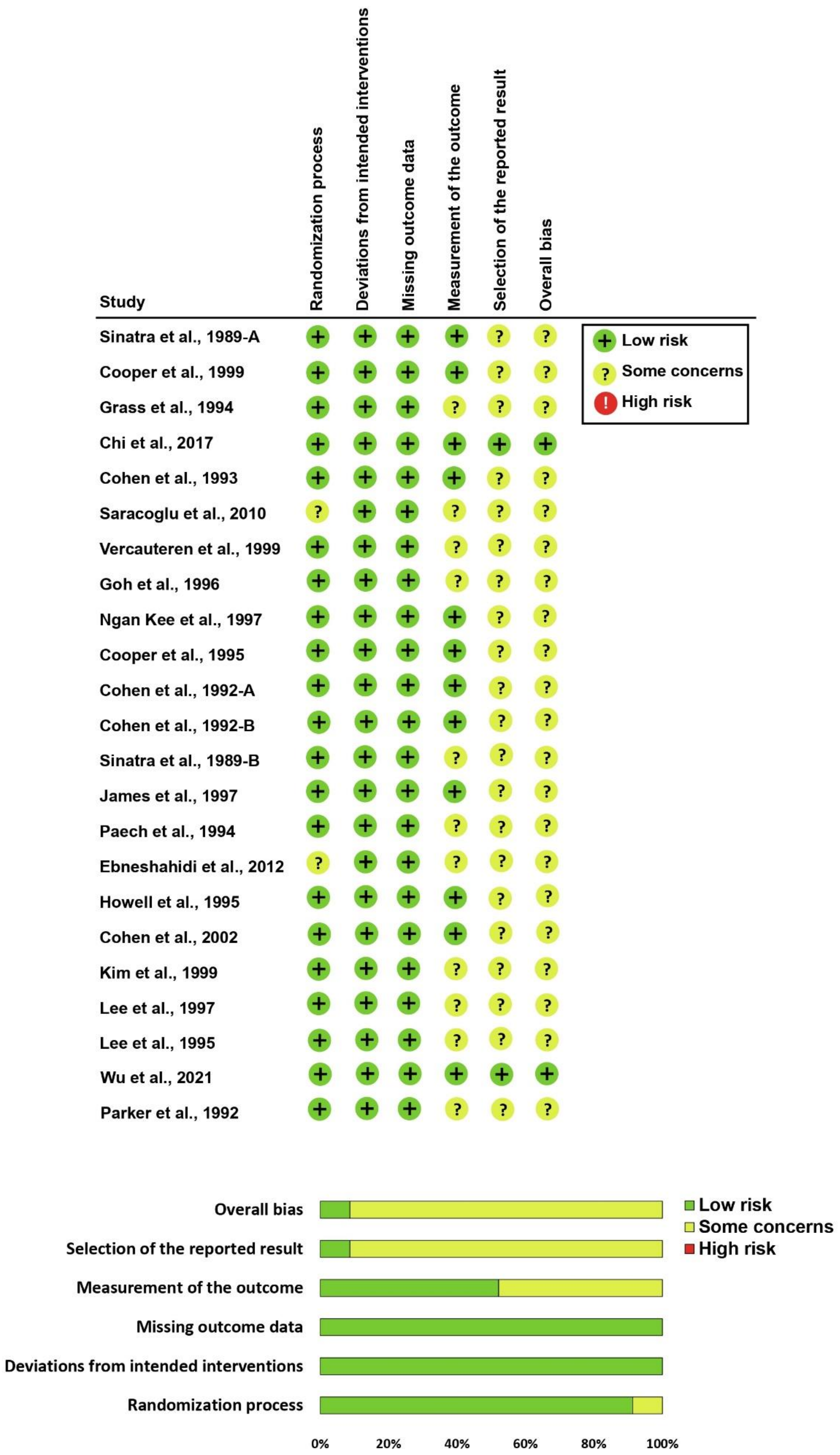

Supplementary Figure S1. Risk of bias summary and graph

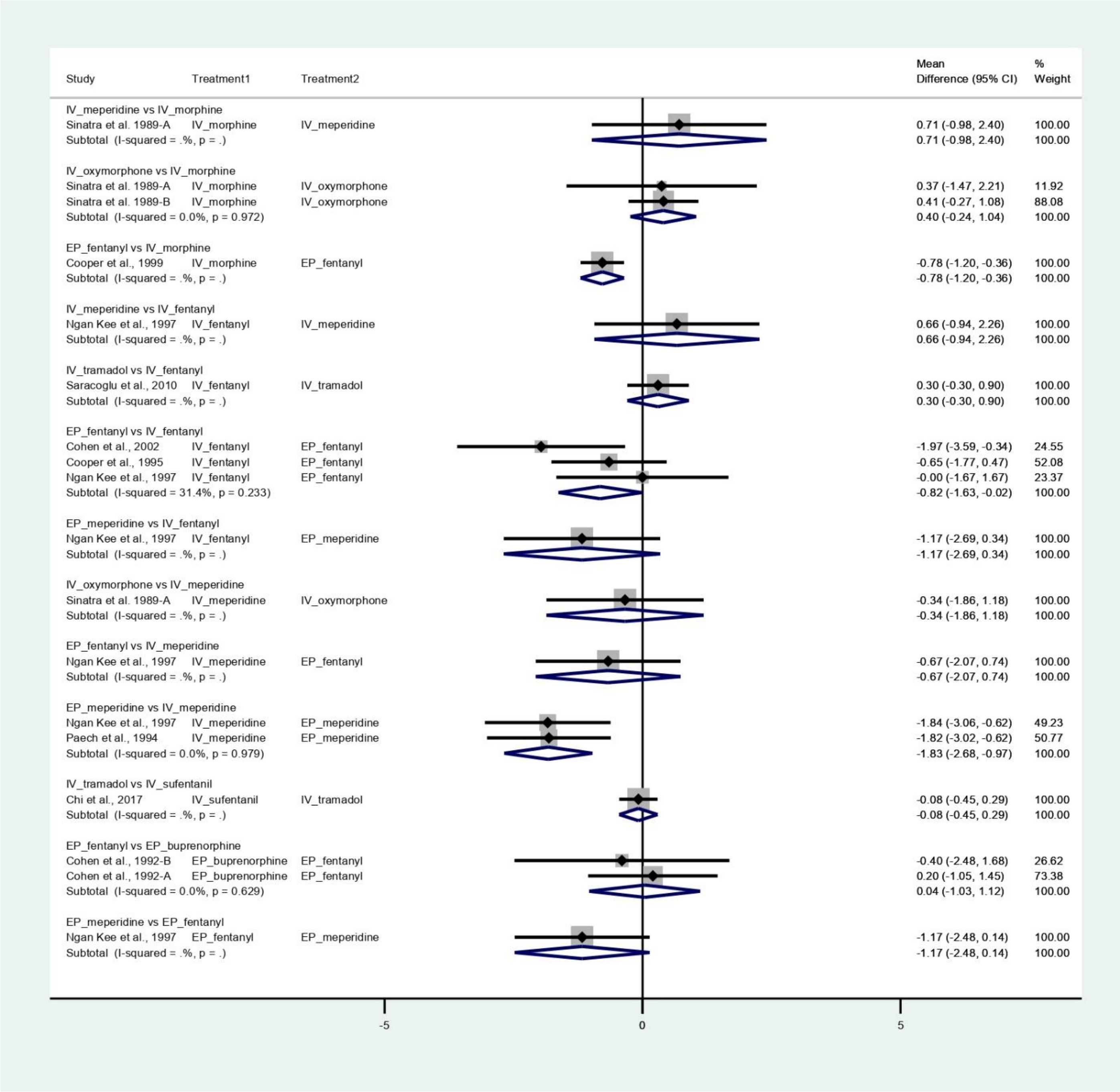

Supplementary Figure S2. Forest plots from pairwise comparisons of treatment agents on pain intensity at 4 hours after surgery

The prefix “IV_” denotes IVPCA, and the prefix “EP_” denotes PCEA. CI, confidence interval.

A.

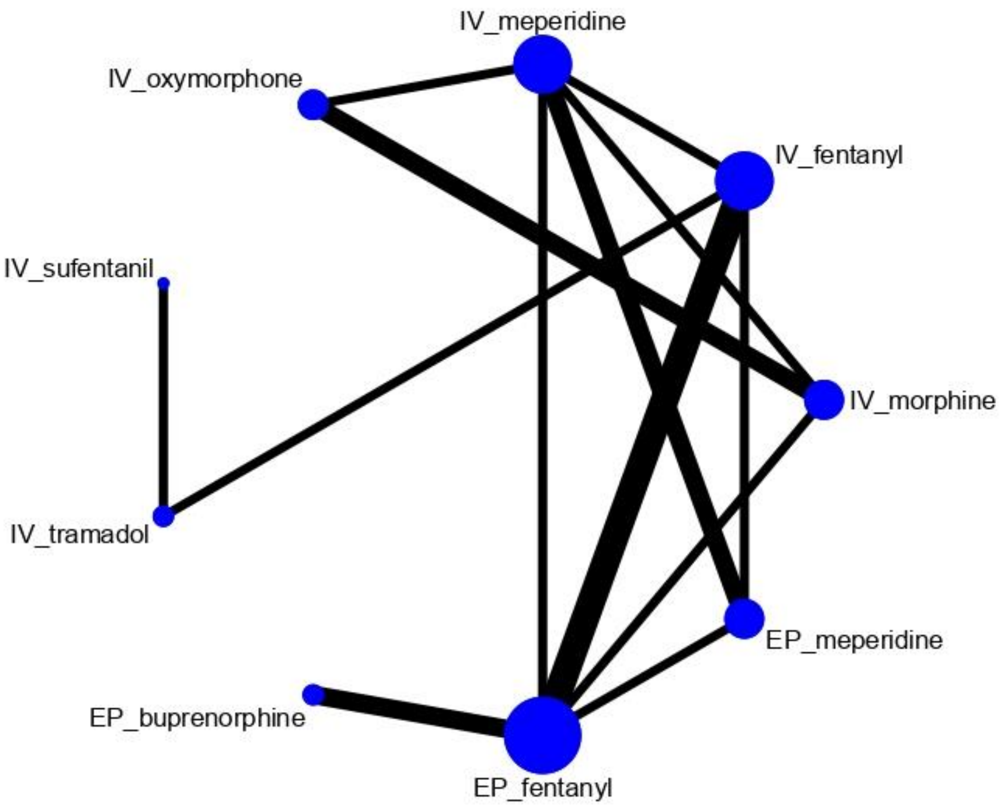
IV_meperidine
IV_oxymorphone
IV_fentanyl
IV_sufentanil
IV_morphine
IV_tramadol
EP_meperidine
EP_buprenorphine
EP_fentanyl

B.

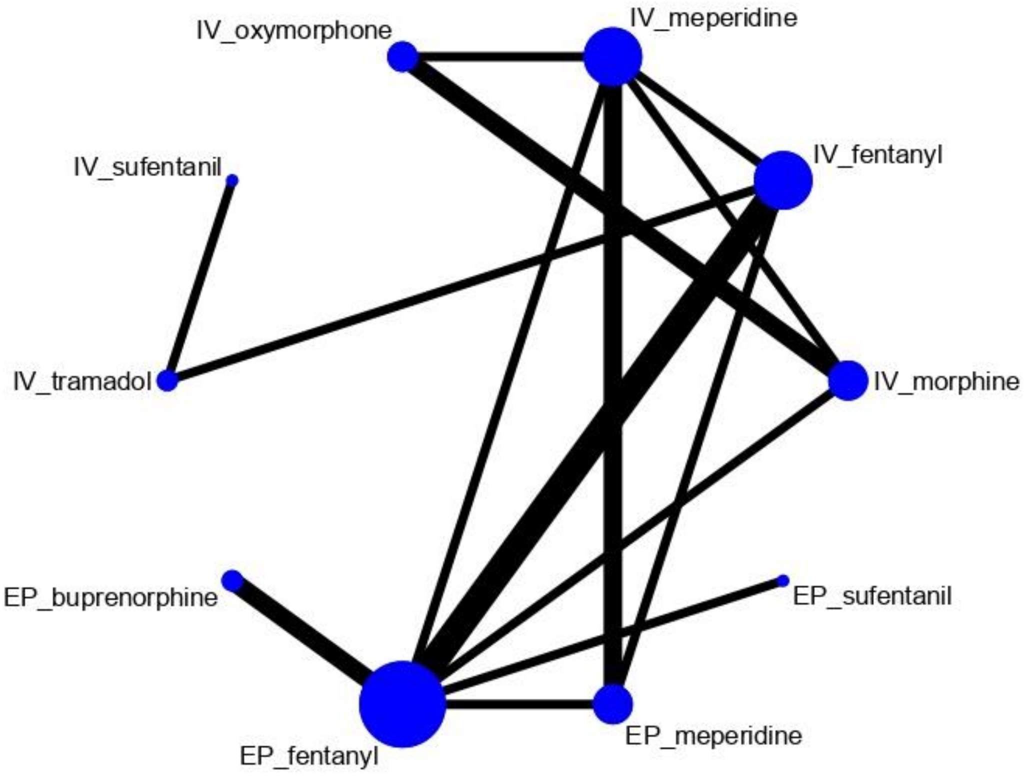
IV_oxymorphone
IV_meperidine
IV_fentanyl
IV_sufentanil
IV_tramadol
IV_morphine
EP_buprenorphine
EP_sufentanil
EP_meperidine
EP_fentanyl

C.

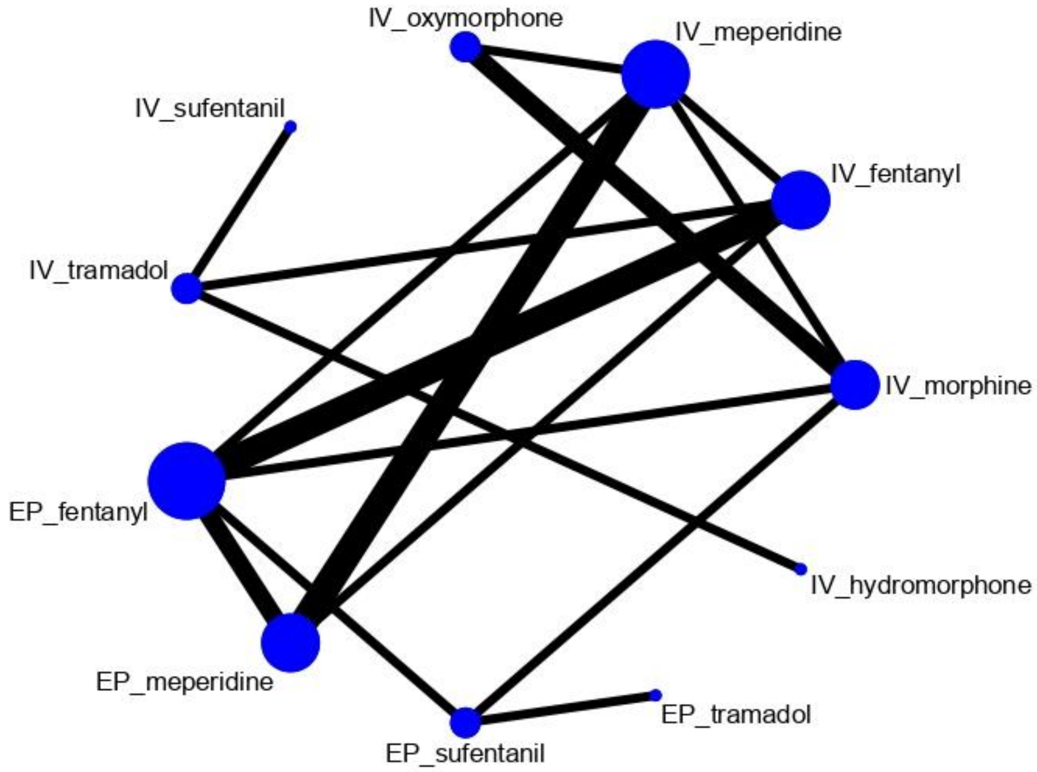
IV_oxymorphone
IV_meperidine
IV_sufentanil
IV_fentanyl
IV_tramadol
IV_morphine
EP_fentanyl
IV_hydromorphone
EP_meperidine
EP_tramadol
EP_sufentanil

D.

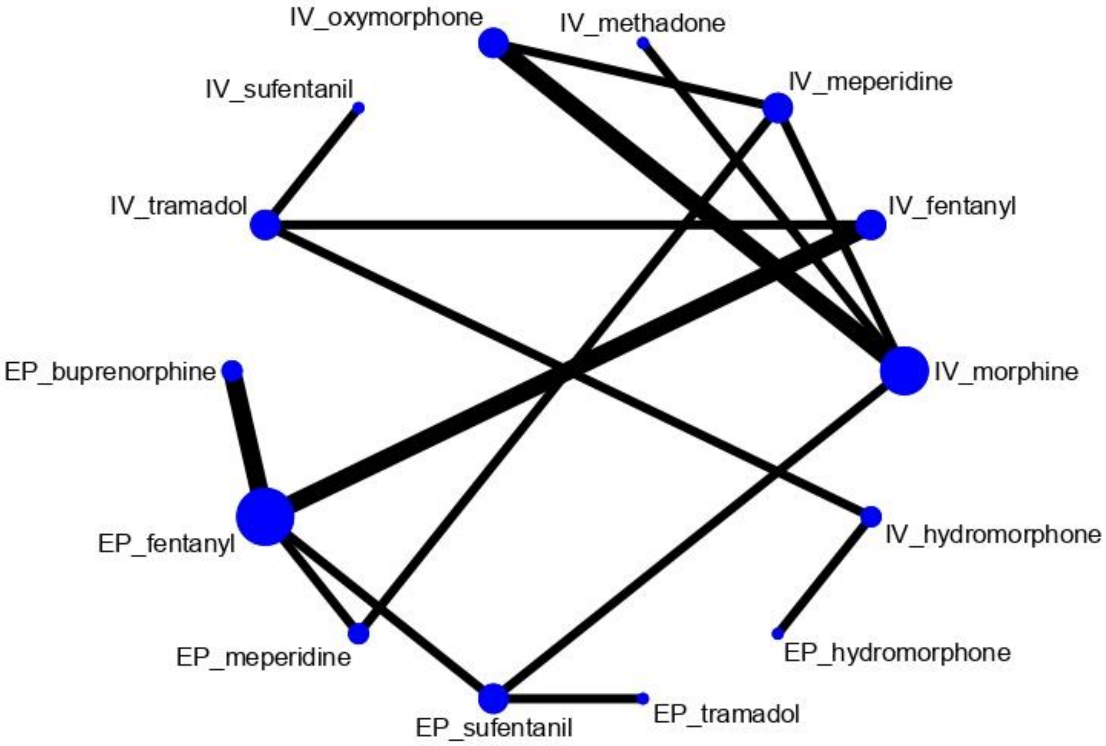
IV_oxymorphone
IV_methadone
IV_sufentanil
IV_meperidine
IV_tramadol
IV_fentanyl
EP_buprenorphine
IV_morphine
EP_fentanyl
IV_hydromorphone
EP_hydromorphone
EP_meperidine
EP_tramadol
EP_sufentanil

Supplementary Figure S3. Network treatment comparisons for studies investigating the effects of opioids on pain intensity
(A) 4 hours after surgery; (B) 8 hours after surgery; (C) 12 hours after surgery; (D) 24 hours after surgery. Each node represents a treatment, the size of which corresponds to the number of patients studied with each treatment. Treatments that are compared directly are joined with a line, the thickness of which corresponds to the number of trials that assessed the comparisons. The prefix "IV_" denotes IVPCA, and the prefix "EP_" denotes PCEA.

Supplementary Table S3. Comparative efficacy of treatments on pain intensity at 4 hours after surgery

**IVPCA morphine**		-0.71 (-2.40, 0.98)	-0.40 (-1.04, 0.24)				0.78 (0.36, 1.20)	
-0.11 (-0.99, 0.77)	**IVPCA fentanyl**	-0.66 (-2.26, 0.94)			-0.30 (-0.90, 0.30)		0.82 (0.02, 1.63)	1.17 (-0.34, 2.69)
-0.44 (-1.36, 0.48)	-0.33 (-1.40, 0.74)	**IVPCA meperidine**	0.34 (-1.18, 1.86)				0.67 (-0.74, 2.07)	1.83 (0.97, 2.68)
-0.36 (-0.99, 0.26)	-0.26 (-1.31, 0.79)	0.08 (-0.93, 1.09)	**IVPCA oxymorphone**					
-0.49 (-1.62, 0.64)	-0.38 (-1.08, 0.32)	-0.05 (-1.33, 1.23)	-0.12 (-1.39, 1.14)	**IVPCA sufentanil**	0.08 (-0.29, 0.45)			
-0.41 (-1.47, 0.65)	-0.30 (-0.90, 0.30)	0.03 (-1.19, 1.26)	-0.04 (-1.25, 1.16)	0.08 (-0.29, 0.45)	**IVPCA tramadol**			
0.79 (-0.36, 1.94)	0.90 (-0.43, 2.24)	1.24 (-0.17, 2.64)	1.16 (-0.14, 2.46)	1.28 (-0.23, 2.79)	1.20 (-0.26, 2.67)	**PCEA buprenorphine**	-0.04 (-1.12, 1.03)	
0.75 (0.34, 1.16)	0.86 (0.07, 1.66)	1.20 (0.29, 2.10)	1.12 (0.39, 1.85)	1.24 (0.18, 2.30)	1.16 (0.17, 2.16)	-0.04 (-1.12, 1.03)	**PCEA fentanyl**	1.17 (-0.14, 2.48)
1.45 (0.42, 2.48)	1.56 (0.44, 2.68)	1.89 (1.07, 2.72)	1.82 (0.68, 2.95)	1.94 (0.62, 3.26)	1.86 (0.59, 3.13)	0.66 (-0.81, 2.12)	0.70 (-0.30, 1.69)	**PCEA meperidine**

Comparisons between treatments should be read from left to right, and the estimate is in the cell in common between the column-defining treatment and the row-defining treatment. The network estimates from network meta-analysis are in the lower triangle, and the direct treatment estimates from pairwise comparisons are in the upper triangle. Estimates are presented as mean difference (95% confidence interval).

A.

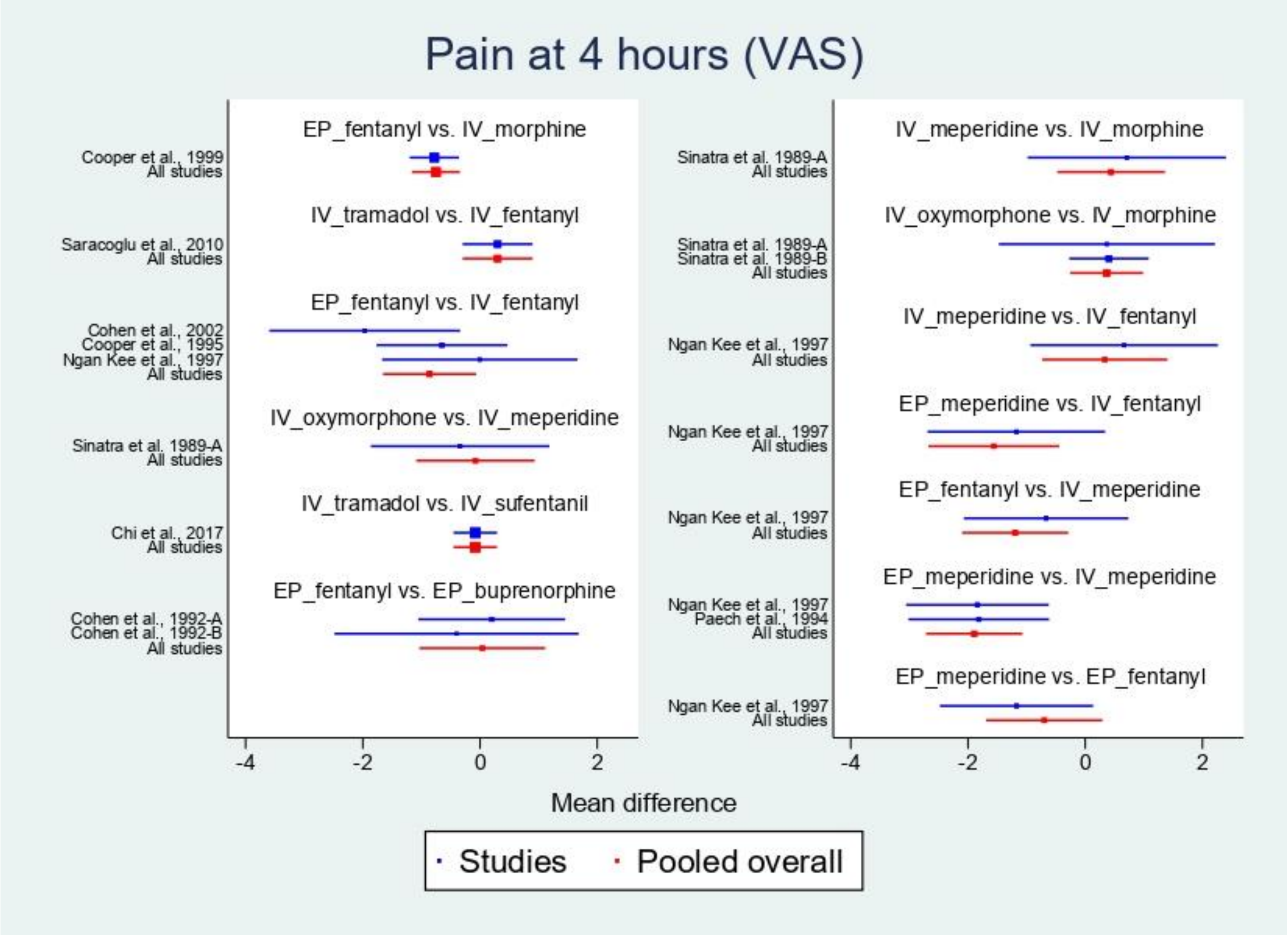

Pain at 4 hours (VAS)
EP_fentanyl vs. IV_morphine
Cooper et al., 1999
All studies
IV_tramadol vs. IV_fentanyl
Saracoglu et al., 2010
All studies
EP_fentanyl vs. IV_fentanyl
Cohen et al., 2002
Cooper et al., 1995
Ngan Kee et al., 1997
All studies
IV_oxymorphone vs. IV_meperidine
Sinatra et al. 1989-A
All studies
IV_tramadol vs. IV_sufentanil
Chi et al., 2017
All studies
EP_fentanyl vs. EP_buprenorphine
Cohen et al., 1992-A
Cohen et al., 1992-B
All studies
IV_meperidine vs. IV_morphine
Sinatra et al. 1989-A
All studies
IV_oxymorphone vs. IV_morphine
Sinatra et al. 1989-A
Sinatra et al. 1989-B
All studies
IV_meperidine vs. IV_fentanyl
Ngan Kee et al., 1997
All studies
EP_meperidine vs. IV_fentanyl
Ngan Kee et al., 1997
All studies
EP_fentanyl vs. IV_meperidine
Ngan Kee et al., 1997
All studies
EP_meperidine vs. IV_meperidine
Ngan Kee et al., 1997
Paech et al., 1994
All studies
EP_meperidine vs. EP_fentanyl
Ngan Kee et al., 1997
All studies
-4
-2
0
2
Mean difference
Studies
Pooled overall

B.

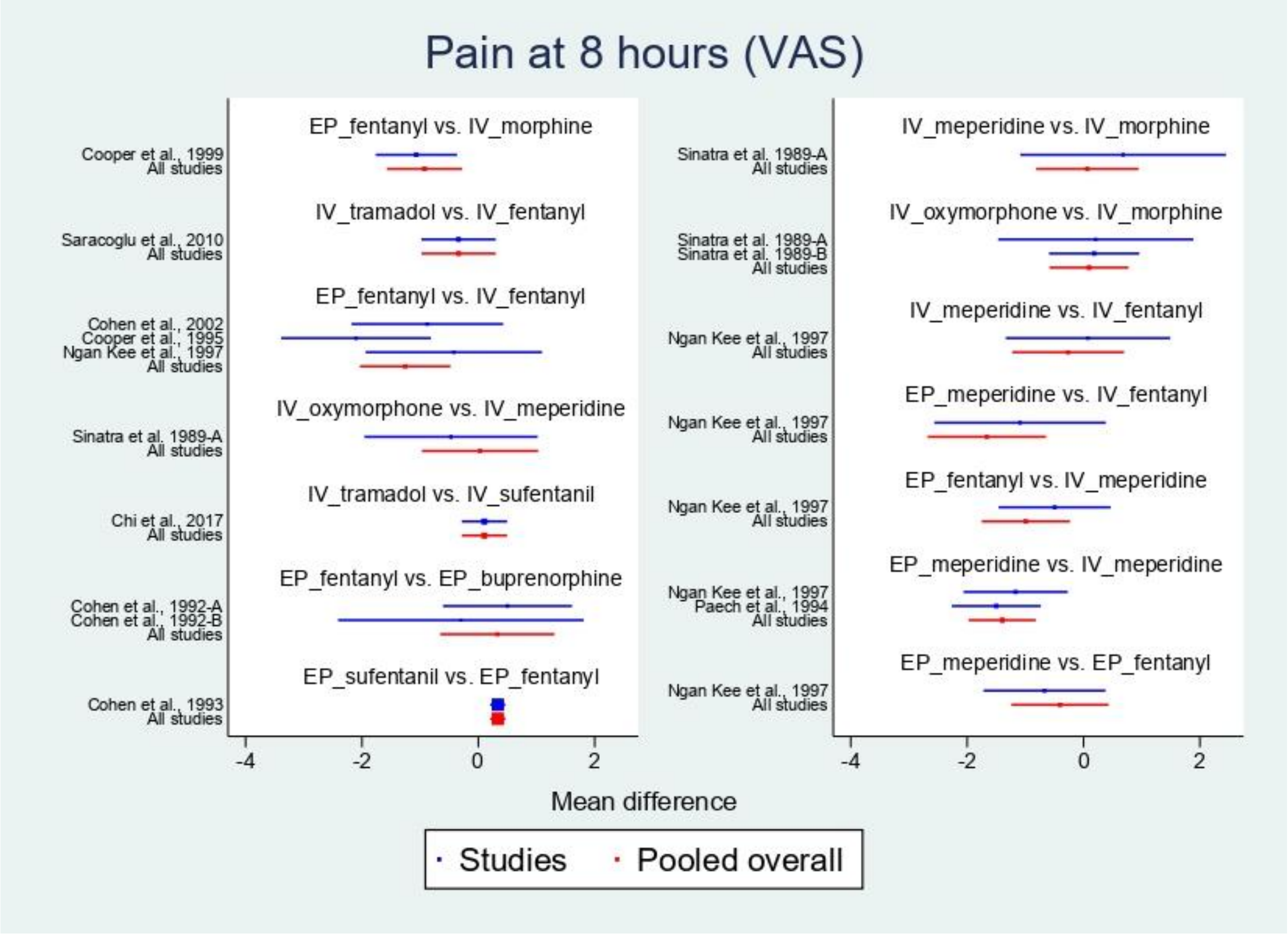

Pain at 8 hours (VAS)
EP_fentanyl vs. IV_morphine
Cooper et al., 1999
All studies
IV_tramadol vs. IV_fentanyl
Saracoglu et al., 2010
All studies
EP_fentanyl vs. IV_fentanyl
Cohen et al., 2002
Cooper et al., 1995
Ngan Kee et al., 1997
All studies
IV_oxymorphone vs. IV_meperidine
Sinatra et al. 1989-A
All studies
IV_tramadol vs. IV_sufentanil
Chi et al., 2017
All studies
EP_fentanyl vs. EP_buprenorphine
Cohen et al., 1992-A
Cohen et al., 1992-B
All studies
EP_sufentanil vs. EP_fentanyl
Cohen et al., 1993
All studies
IV_meperidine vs. IV_morphine
Sinatra et al. 1989-A
All studies
IV_oxymorphone vs. IV_morphine
Sinatra et al. 1989-A
Sinatra et al. 1989-B
All studies
IV_meperidine vs. IV_fentanyl
Ngan Kee et al., 1997
All studies
EP_meperidine vs. IV_fentanyl
Ngan Kee et al., 1997
All studies
EP_fentanyl vs. IV_meperidine
Ngan Kee et al., 1997
All studies
EP_meperidine vs. IV_meperidine
Ngan Kee et al., 1997
Paech et al., 1994
All studies
EP_meperidine vs. EP_fentanyl
Ngan Kee et al., 1997
All studies
-4
-2
0
2
Mean difference
Studies
Pooled overall

C.

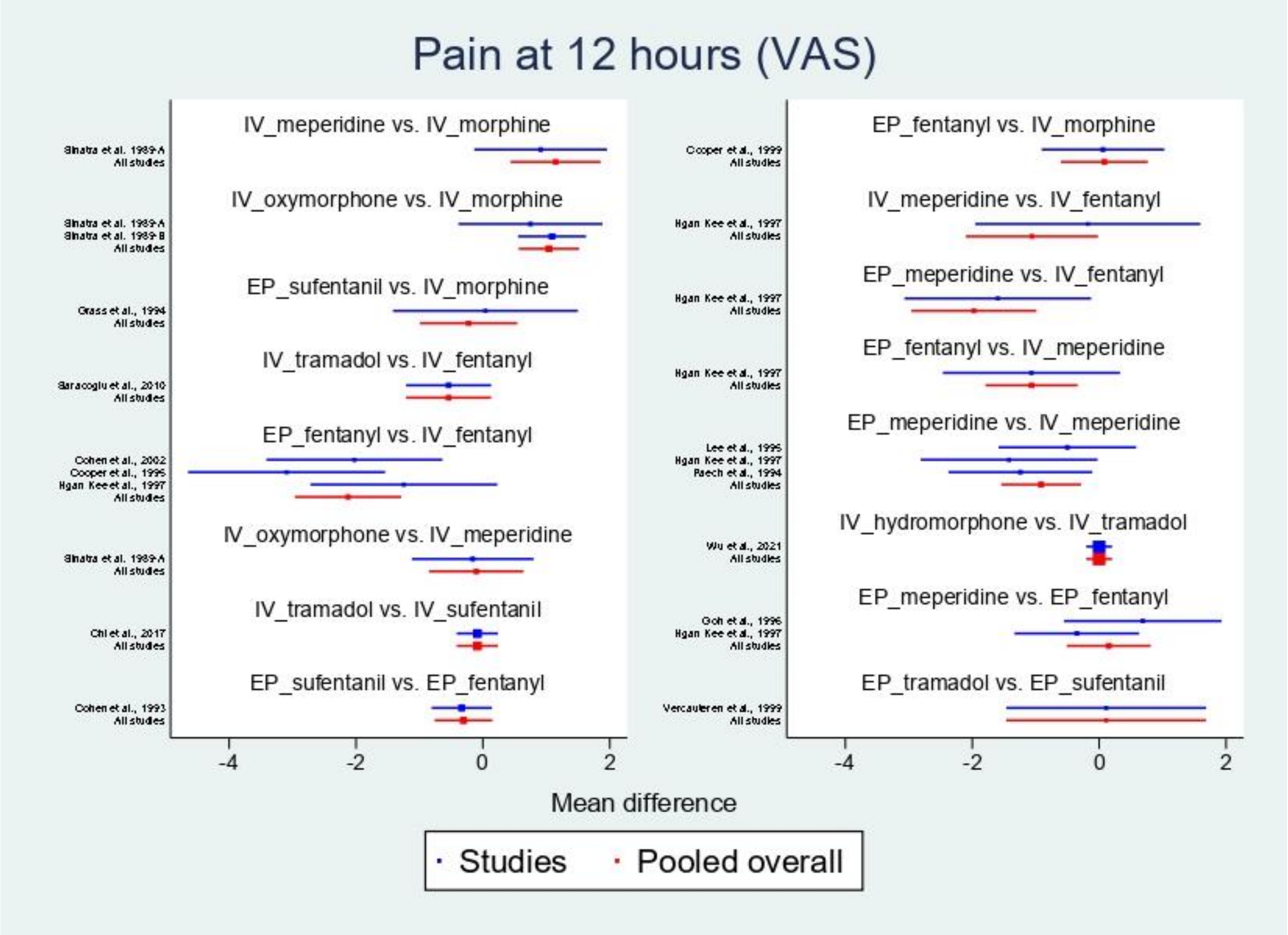

D.

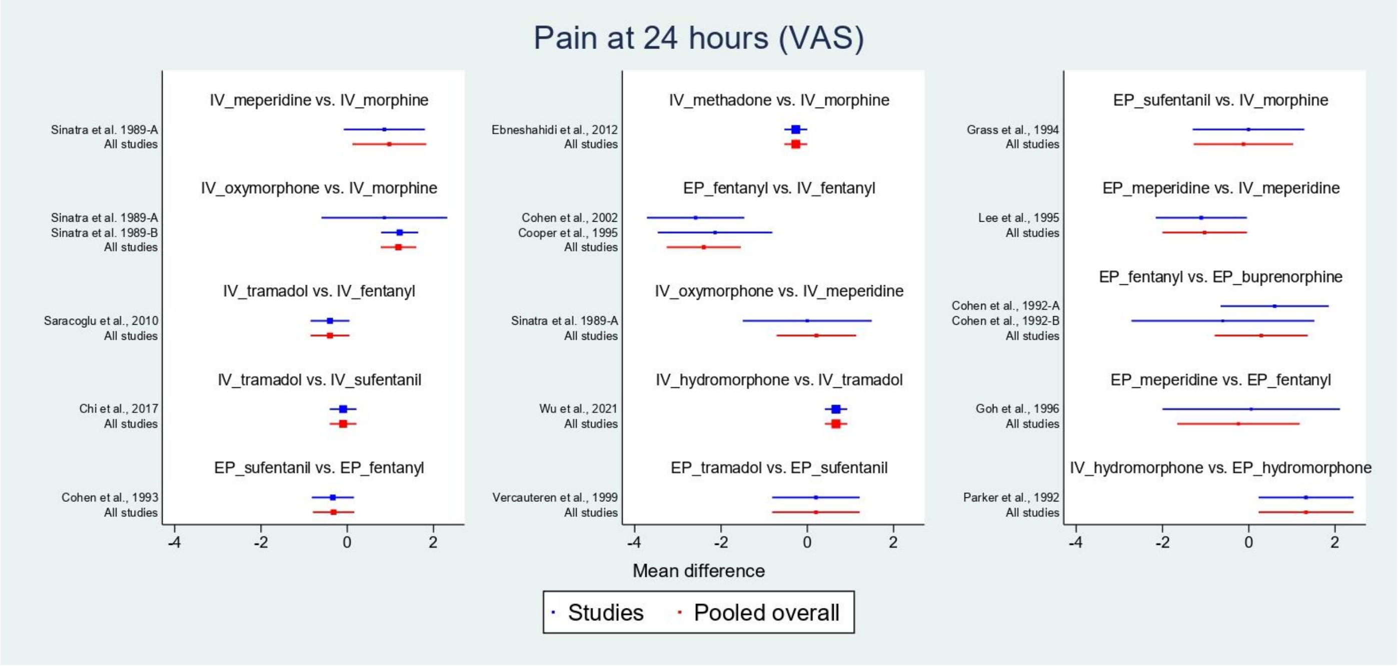

Supplementary Figure S4. Network meta-analysis forest plots of pain intensity
(A) 4 hours; (B) 8 hours; (C) 12 hours; (D) 24 hours after surgery. The blue dots and lines indicate the studies contributing the direct evidence. The red dots and lines indicate the pooled overall network estimates of the treatment effects. The prefix "IV_" denotes IVPCA, and the prefix "EP_" denotes PCEA. VAS, visual analogue scale.

A.

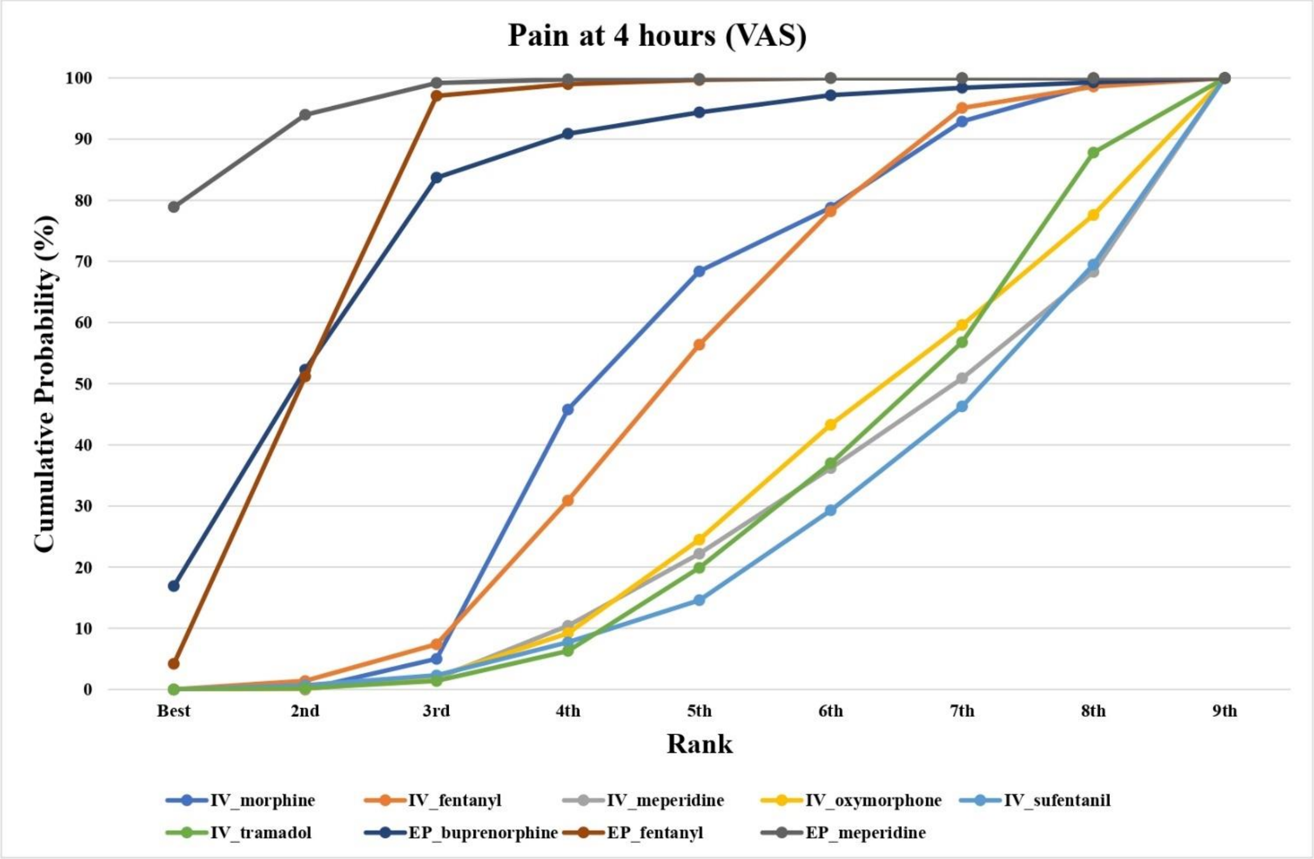
Pain at 4 hours (VAS)
Cumulative Probability (%)
100
90
80
70
60
50
40
30
20
10
0
Best
2nd
3rd
4th
5th
6th
7th
8th
9th
Rank
IV_morphine
IV_fentanyl
IV_meperidine
IV_oxymorphone
IV_sufentanil
IV_tramadol
EP_buprenorphine
EP_fentanyl
EP_meperidine

B.

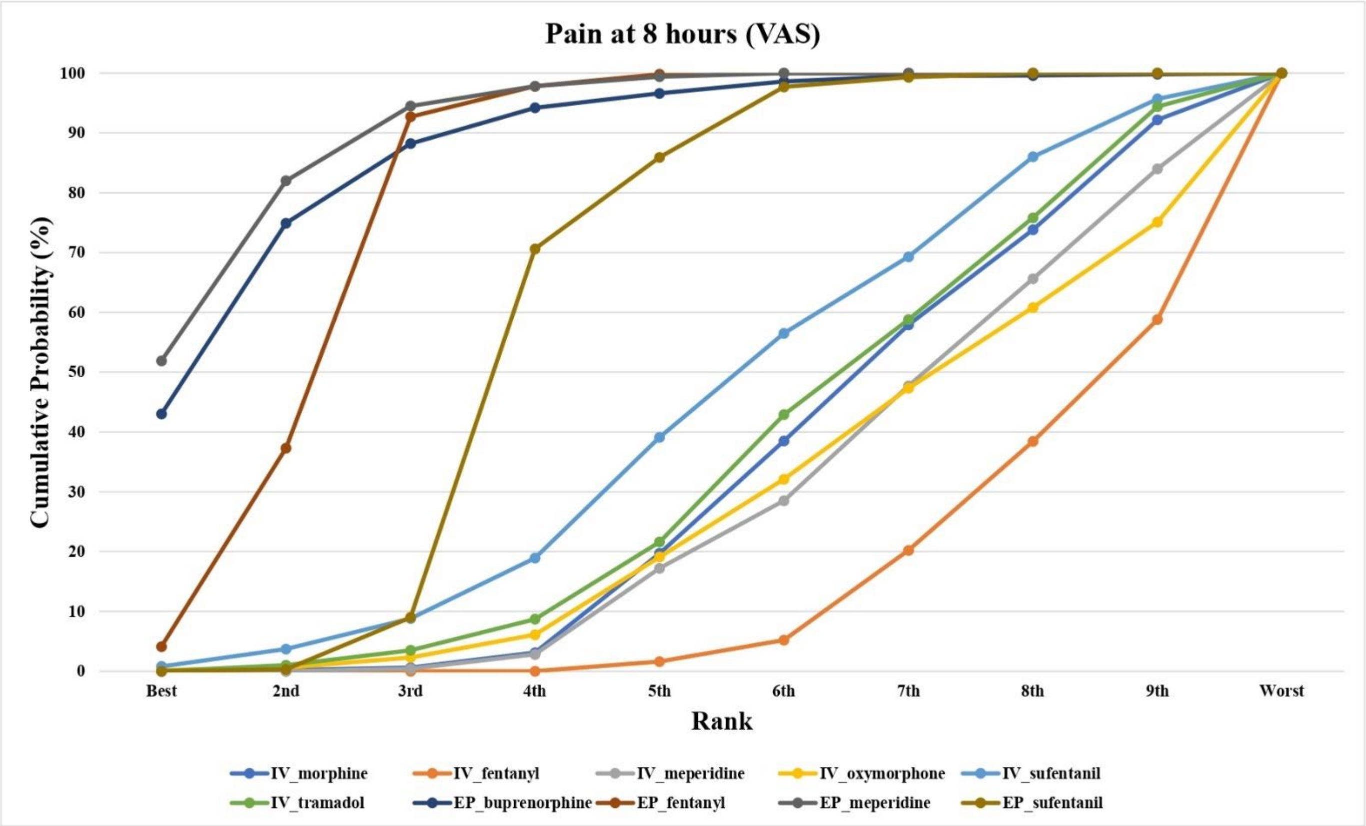
Pain at 8 hours (VAS)
Cumulative Probability (%)
100
90
80
70
60
50
40
30
20
10
0
Best
2nd
3rd
4th
5th
6th
7th
8th
9th
Worst
Rank
IV_morphine
IV_fentanyl
IV_meperidine
IV_oxymorphone
IV_sufentanil
IV_tramadol
EP_buprenorphine
EP_fentanyl
EP_meperidine
EP_sufentanil

C.

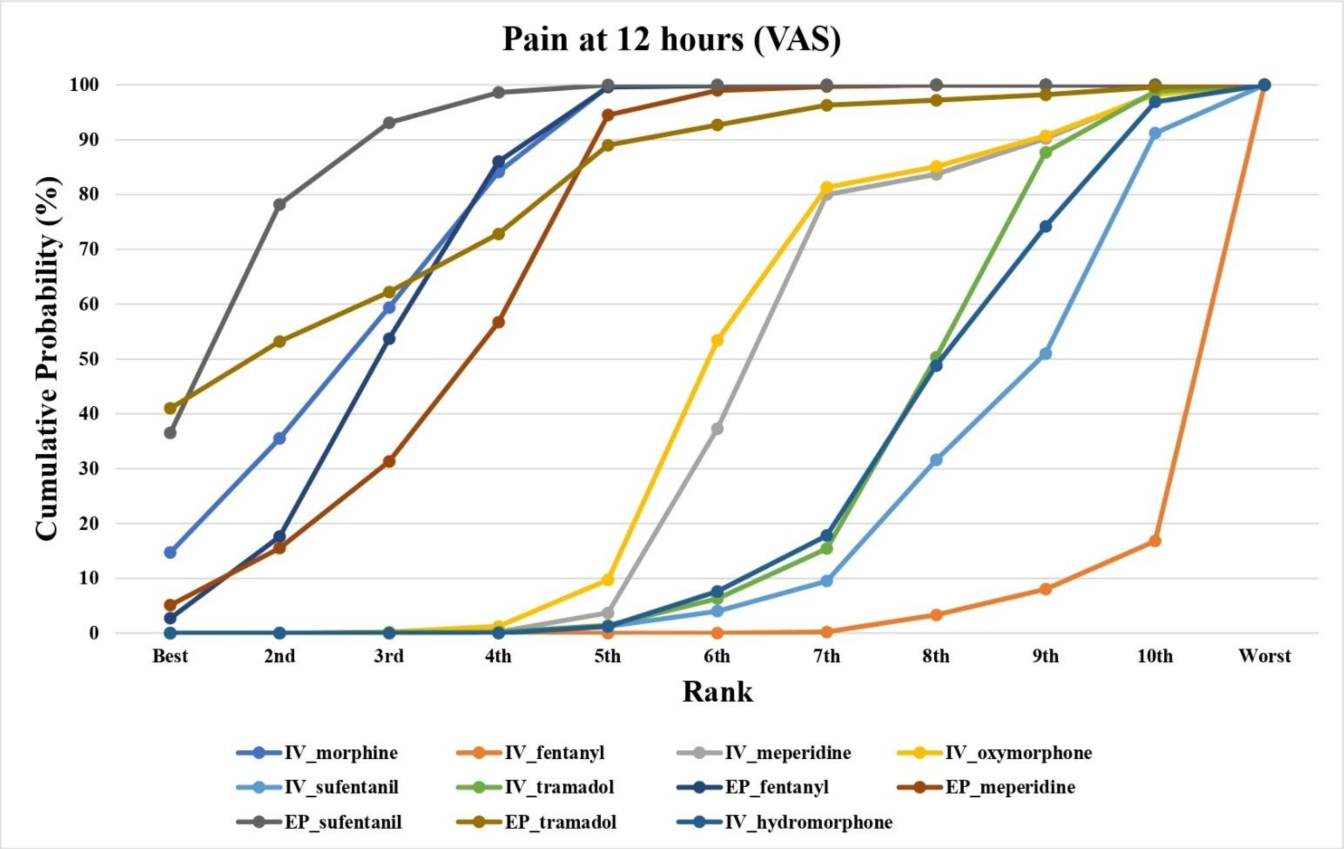

D.

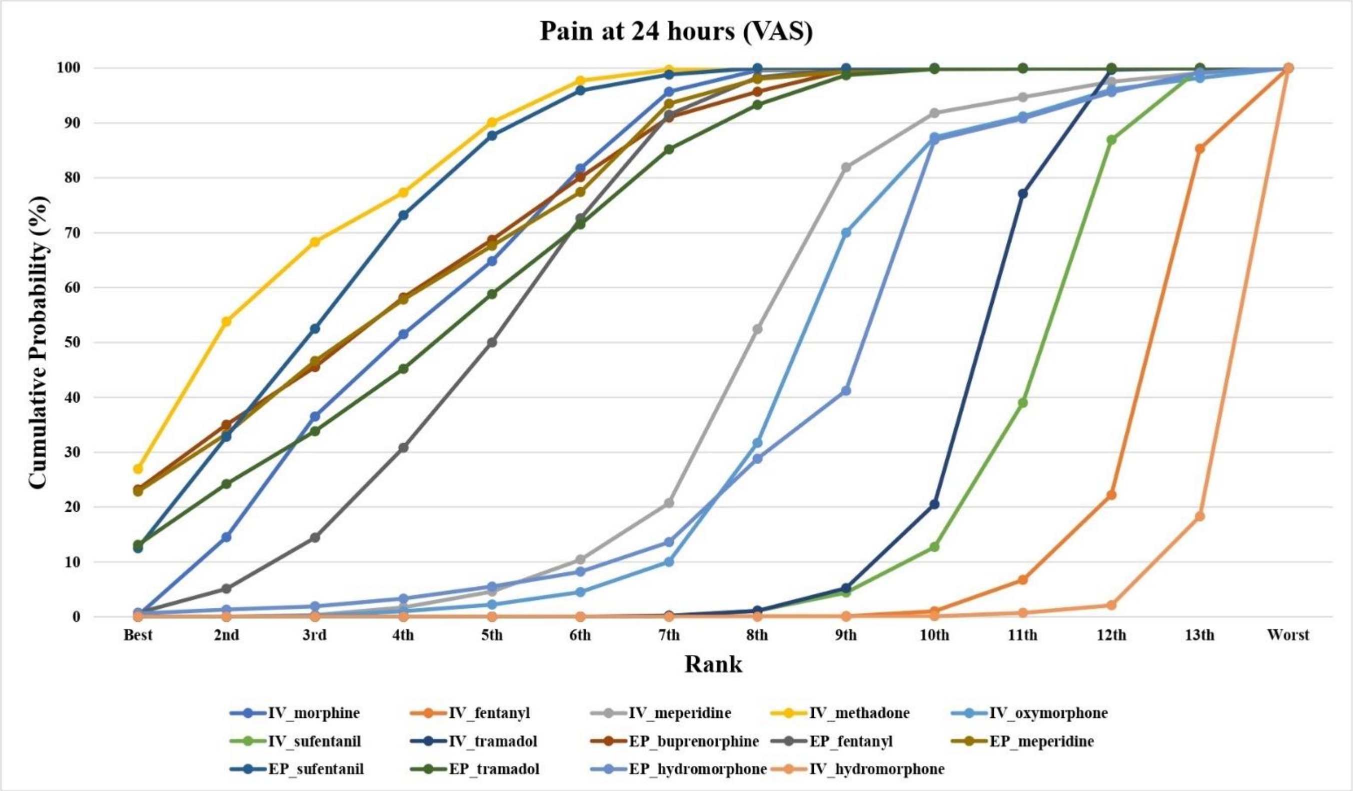

Supplementary Figure S5. Cumulative ranking probability of the effects of each treatment on pain intensity (A) 4 hours; (B) 8 hours; (C) 12 hours; (D) 24 hours after surgery. The prefix "IV_" denotes IVPCA, and the prefix "EP_" denotes PCEA. VAS, visual analogue scale.

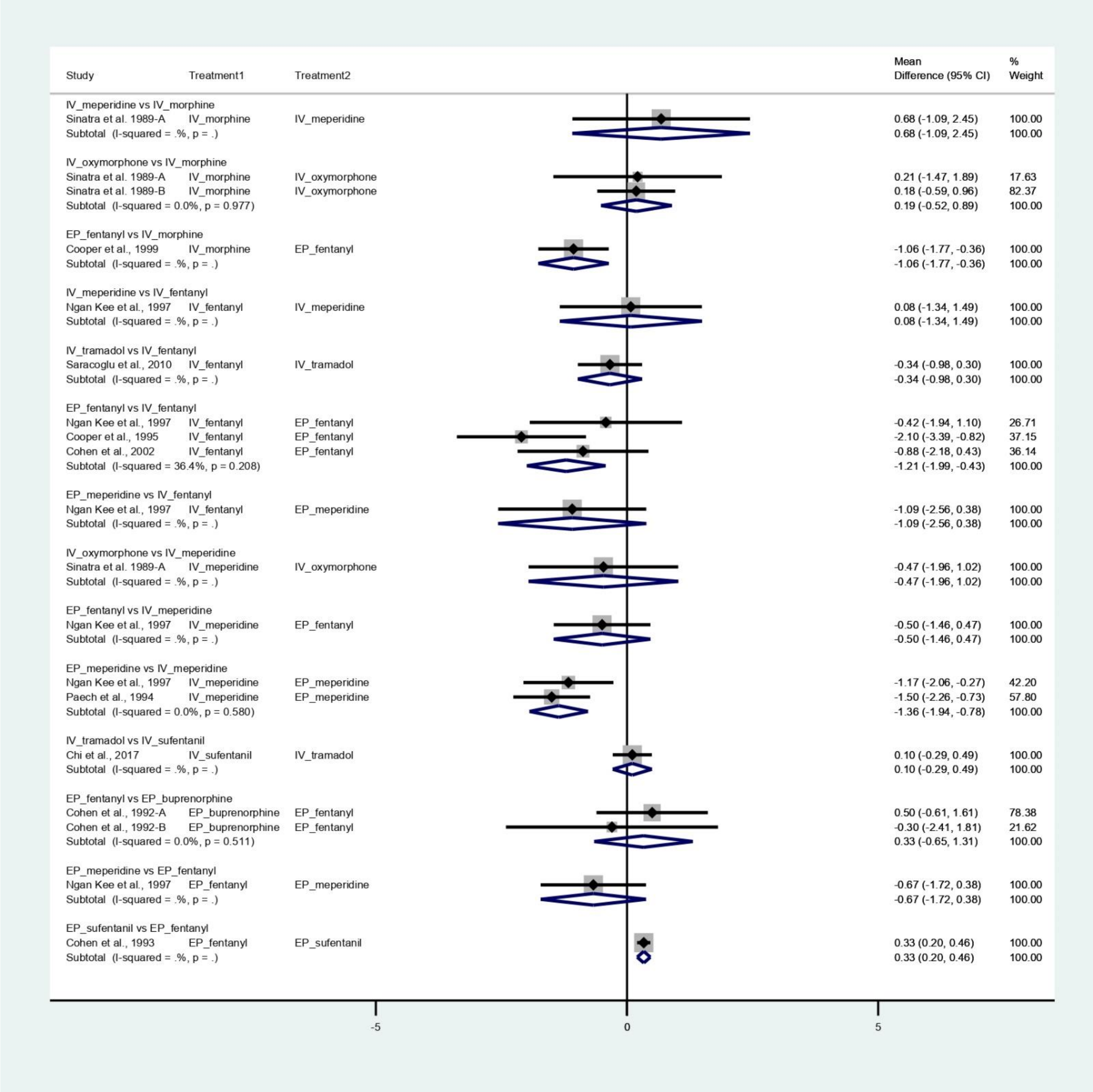

Supplementary Figure S6. Forest plots from pairwise comparisons of treatment agents on pain intensity at 8 hours after surgery

The prefix "IV_" denotes IVPCA, and the prefix "EP_" denotes PCEA. CI, confidence interval.

Supplementary Table S4. Comparative efficacy of treatments on pain intensity at 8 hours after surgery

**IVPCA morphine**		-0.68 (-2.45, 1.09)	-0.19 (-0.89, 0.52)				1.06 (0.36, 1.77)		
-0.33 (-1.32, 0.66)	**IVPCA fentanyl**	-0.08 (-1.49, 1.34)			0.34 (-0.30, 0.98)		1.21 (0.43, 1.99)	1.09 (-0.38, 2.56)	
-0.07 (-0.95, 0.82)	0.27 (-0.70, 1.23)	**IVPCA meperidine**	0.47 (-1.02, 1.96)				0.50 (-0.47, 1.46)	1.36 (0.78, 1.94)	
-0.09 (-0.78, 0.59)	0.24 (-0.91, 1.38)	-0.03 (-1.03, 0.97)	**IVPCA oxymorphone**						
0.11 (-1.13, 1.35)	0.44 (-0.30, 1.19)	0.18 (-1.04, 1.40)	0.21 (-1.16, 1.58)	**IVPCA sufentanil**	-0.10 (-0.49, 0.29)				
0.01 (-1.17, 1.19)	0.34 (-0.30, 0.98)	0.07 (-1.08, 1.23)	0.10 (-1.21, 1.42)	-0.10 (-0.49, 0.29)	**IVPCA tramadol**				
1.25 (0.08, 2.43)	1.58 (0.33, 2.84)	1.32 (0.08, 2.56)	1.35 (0.02, 2.67)	1.14 (-0.32, 2.60)	1.24 (-0.16, 2.65)	**PCEA buprenorphine**	-0.33 (-1.31, 0.65)		
0.93 (0.28, 1.57)	1.26 (0.48, 2.03)	0.99 (0.23, 1.75)	1.02 (0.13, 1.91)	0.81 (-0.27, 1.89)	0.92 (-0.09, 1.92)	-0.33 (-1.31, 0.65)	**PCEA fentanyl**	0.67 (-0.38, 1.72)	-0.33 (-0.46, -0.20)
1.33 (0.36, 2.30)	1.66 (0.64, 2.68)	1.40 (0.82, 1.97)	1.43 (0.33, 2.52)	1.22 (-0.05, 2.48)	1.32 (0.12, 2.52)	0.08 (-1.21, 1.37)	0.41 (-0.43, 1.24)	**PCEA meperidine**	
0.59 (-0.07, 1.25)	0.92 (0.13, 1.71)	0.66 (-0.11, 1.43)	0.69 (-0.21, 1.58)	0.48 (-0.61, 1.57)	0.58 (-0.43, 1.60)	-0.66 (-1.65, 0.33)	-0.33 (-0.46, -0.20)	-0.74 (-1.59, 0.11)	**PCEA sufentanil**

Comparisons between treatments should be read from left to right, and the estimate is in the cell in common between the column-defining treatment and the row-defining treatment. The network estimates from network meta-analysis are in the lower triangle, and the direct treatment estimates from pairwise comparisons are in the upper triangle. Estimates are presented as mean difference (95% confidence interval).

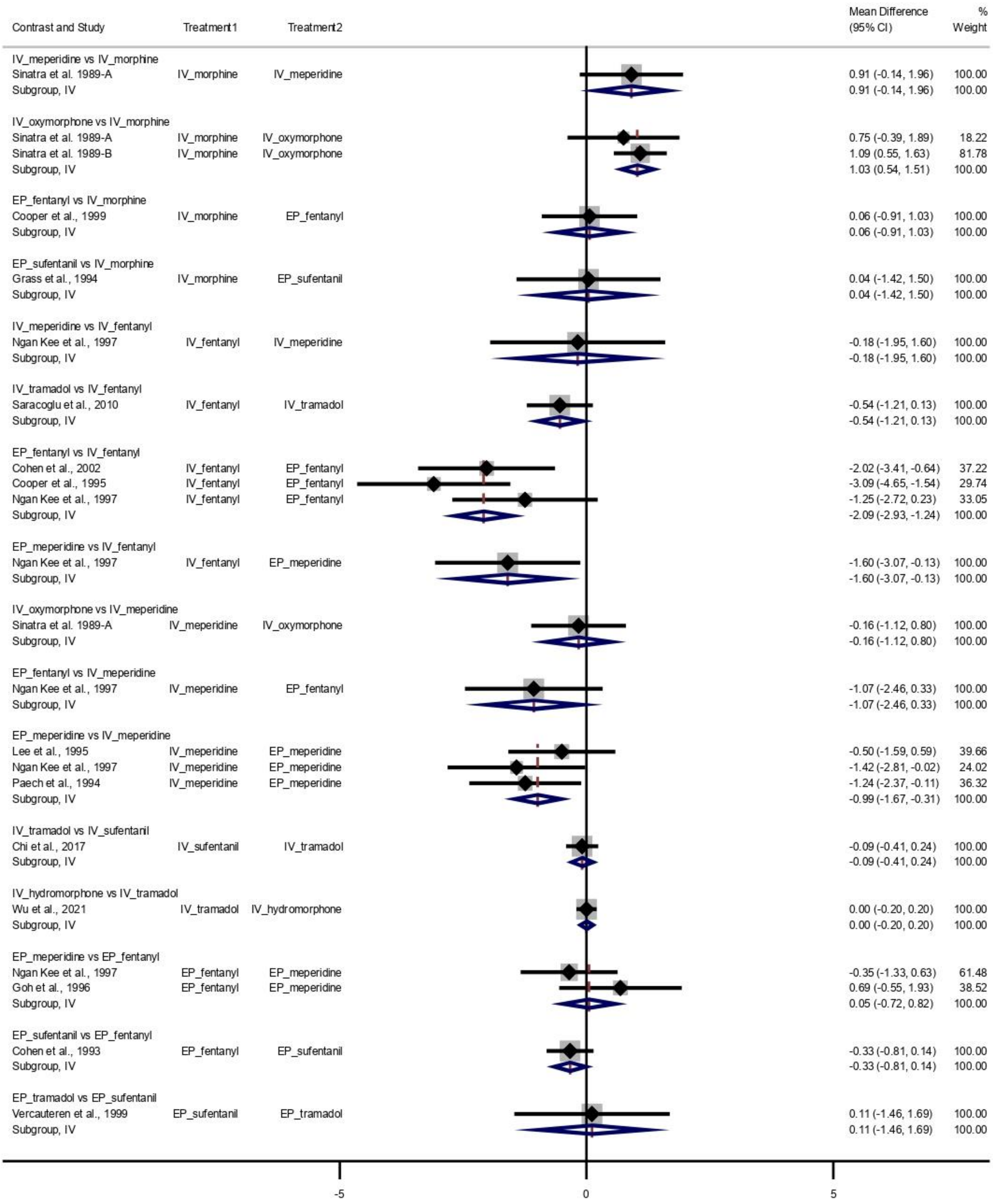

Supplementary Figure S7. Forest plots from pairwise comparisons of treatment agents on pain intensity at 12 hours after surgery

The prefix "IV_" denotes IVPCA, and the prefix "EP_" denotes PCEA. CI, confidence interval.

Supplementary Table S5. Comparative efficacy of treatments on pain intensity at 12 hours after surgery

**IVPCA morphine**		-0.91 (-1.96, 0.14)	-1.03 (-1.51, -0.54)			-0.06 (-1.03, 0.91)		-0.04 (-1.50, 1.42)		
-2.21 (-3.26, -1.15)	**IVPCA fentanyl**	0.18 (-1.60, 1.95)			0.54 (-0.13, 1.21)	2.09 (1.24, 2.93)	1.60 (0.13, 3.07)			
-1.15 (-1.86, -0.44)	1.06 (0.02, 2.10)	**IVPCA meperidine**	0.16 (-0.80, 1.12)			1.07 (-0.33, 2.46)	0.99 (0.31, 1.67)			
-1.04 (-1.52, -0.57)	1.16 (0.05, 2.28)	0.10 (-0.64, 0.85)	**IVPCA oxymorphone**							
-1.75 (-3.04, -0.47)	0.45 (-0.29, 1.20)	-0.61 (-1.89, 0.67)	-0.71 (-2.05, 0.63)	**IVPCA sufentanil**	0.09 (-0.24, 0.41)					
-1.67 (-2.91, -0.42)	0.54 (-0.13, 1.21)	-0.52 (-1.76, 0.72)	-0.62 (-1.92, 0.68)	0.09 (-0.24, 0.41)	**IVPCA tramadol**					0.00 (-0.20, 0.20)
-0.08 (-0.77, 0.61)	2.12 (1.29, 2.96)	1.06 (0.34, 1.79)	0.96 (0.17, 1.75)	1.67 (0.55, 2.79)	1.58 (0.51, 2.66)	**PCEA fentanyl**	-0.05 (-0.82, 0.72)	0.33 (-0.14, 0.81)		
-0.23 (-1.03, 0.57)	1.97 (0.99, 2.96)	0.91 (0.28, 1.54)	0.81 (-0.05, 1.67)	1.52 (0.29, 2.76)	1.43 (0.24, 2.63)	-0.15 (-0.81, 0.51)	**PCEA meperidine**			
0.22 (-0.54, 0.99)	2.43 (1.48, 3.38)	1.37 (0.54, 2.20)	1.27 (0.40, 2.13)	1.98 (0.77, 3.19)	1.89 (0.73, 3.05)	0.31 (-0.15, 0.76)	0.46 (-0.33, 1.24)	**PCEA sufentanil**	-0.11 (-1.69, 1.46)	
0.11 (-1.64, 1.86)	2.32 (0.48, 4.16)	1.26 (-0.52, 3.04)	1.15 (-0.64, 2.95)	1.87 (-0.12, 3.85)	1.78 (-0.18, 3.74)	0.19 (-1.45, 1.83)	0.35 (-1.42, 2.11)	-0.11 (-1.69, 1.46)	**PCEA tramadol**	
-1.67 (-2.93, -0.40)	0.54 (-0.16, 1.24)	-0.52 (-1.78, 0.74)	-0.62 (-1.94, 0.69)	0.09 (-0.30, 0.47)	0.00 (-0.20, 0.20)	-1.58 (-2.68, -0.49)	-1.43 (-2.64, -0.22)	-1.89 (-3.07, -0.71)	-1.78 (-3.75, 0.19)	**IVPCA hydromorphone**

Comparisons between treatments should be read from left to right, and the estimate is in the cell in common between the column-defining treatment and the row-defining treatment. The network estimates from network meta-analysis are in the lower triangle, and the direct treatment estimates from pairwise comparisons are in the upper triangle. Estimates are presented as mean difference (95% confidence interval).

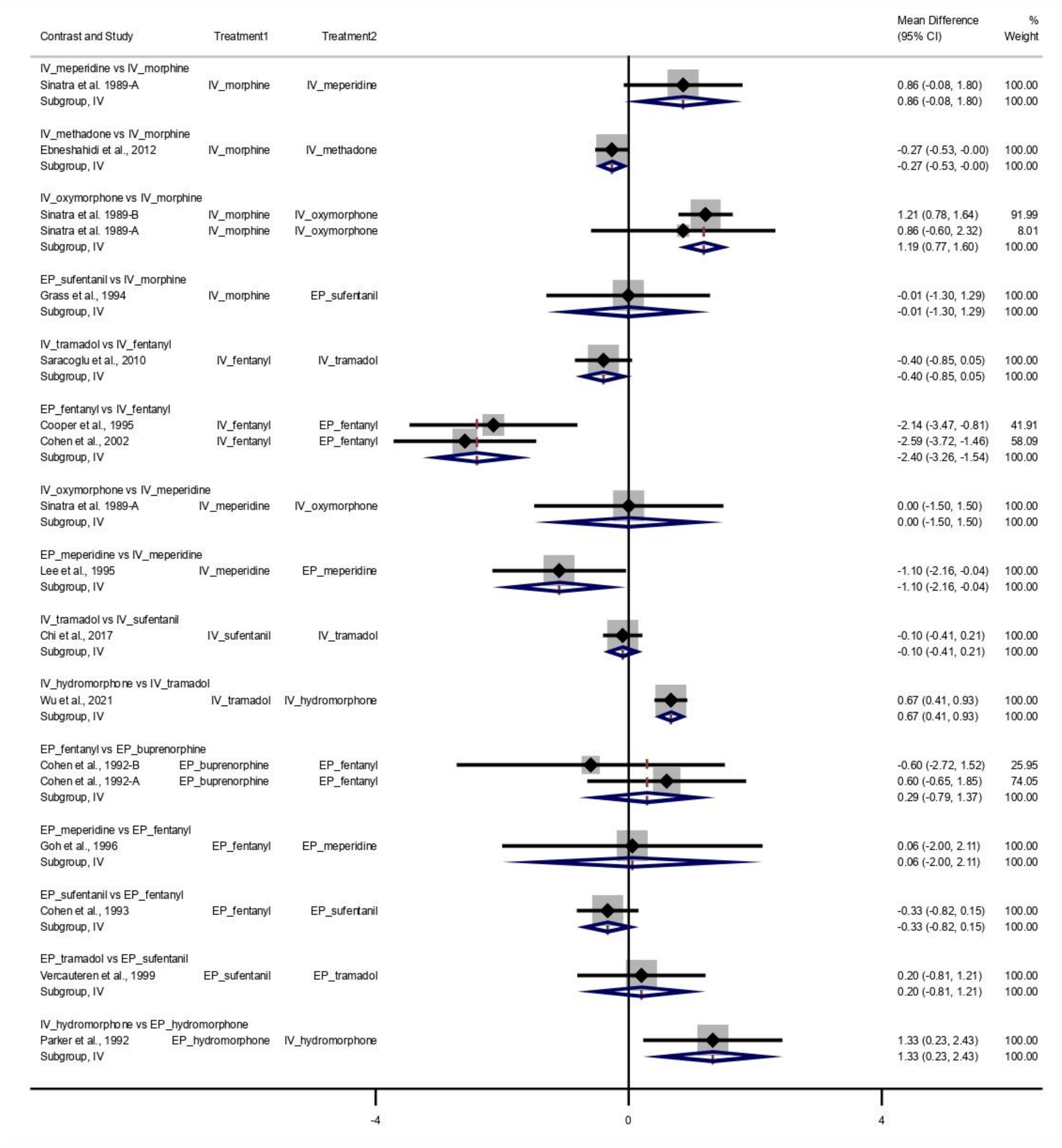

Supplementary Figure S8. Forest plots from pairwise comparisons of treatment agents on pain intensity at 24 hours after surgery

The prefix "IV_" denotes IVPCA, and the prefix "EP_" denotes PCEA. CI, confidence interval.

Supplementary Table S6. Comparative efficacy of treatments on pain intensity at 24 hours after surgery

**IVPCA morphine**		-0.86 (-1.80, 0.08)	0.27 (0.00, 0.53)	-1.19 (-1.60, -0.77)						0.01 (-1.29, 1.30)			
-2.59 (-4.08, -1.11)	**IVPCA fentanyl**					0.40 (-0.05, 0.85)		2.40 (1.54, 3.26)					
-0.98 (-1.83, -0.12)	1.62 (0.02, 3.21)	**IVPCA meperidine**		0.00 (-1.50, 1.50)					1.10 (0.04, 2.16)				
0.27 (0.00, 0.53)	2.86 (1.35, 4.37)	1.24 (0.35, 2.14)	**IVPCA methadone**										
-1.19 (-1.60, -0.78)	1.41 (-0.13, 2.94)	-0.21 (-1.13, 0.71)	-1.45 (-1.94, -0.96)	**IVPCA oxymorphone**									
-2.29 (-3.87, -0.71)	0.30 (-0.24, 0.85)	-1.31 (-3.00, 0.37)	-2.56 (-4.16, -0.95)	-1.10 (-2.73, 0.53)	**IVPCA sufentanil**	0.10 (-0.21, 0.41)							
-2.19 (-3.74, -0.64)	0.40 (-0.05, 0.85)	-1.22 (-2.88, 0.44)	-2.46 (-4.03, -0.89)	-1.01 (-2.60, 0.59)	0.10 (-0.21, 0.41)	**IVPCA tramadol**							-0.67 (-0.93, -0.41)
0.10 (-1.52, 1.72)	2.69 (1.31, 4.07)	1.07 (-0.65, 2.80)	-0.17 (-1.81, 1.47)	1.28 (-0.38, 2.95)	2.38 (0.90, 3.87)	2.29 (0.84, 3.74)	**PCEA buprenorphine**	-0.29 (-1.37, 0.79)					
-0.19 (-1.40, 1.02)	2.40 (1.54, 3.26)	0.78 (-0.56, 2.13)	-0.46 (-1.70, 0.78)	1.00 (-0.28, 2.27)	2.10 (1.08, 3.12)	2.00 (1.03, 2.97)	-0.29 (-1.37, 0.79)	**PCEA fentanyl**	-0.06 (-2.11, 2.00)	0.33 (-0.15, 0.82)			
0.05 (-1.17, 1.26)	2.64 (0.98,	1.02 (0.04, 2.00)	-0.22 (-1.46, 1.02)	1.23 (-0.03, 2.50)	2.33 (0.59, 4.08)	2.24 (0.52, 3.96)	-0.05 (-1.83, 1.73)	0.24 (-1.18, 1.66)	**PCEA meperidine**				

	4.30)												
0.12 (-1.03, 1.28)	2.72 (1.73, 3.70)	1.10 (-0.21, 2.41)	-0.14 (-1.32, 1.04)	1.31 (0.09, 2.53)	2.41 (1.29, 3.54)	2.32 (1.23, 3.40)	0.03 (-1.15, 1.21)	0.32 (-0.16, 0.80)	0.08 (-1.33, 1.49)	**PCEA sufentanil**	-0.20 (-1.21, 0.81)		
-0.08 (-1.61, 1.46)	2.52 (1.10, 3.93)	0.90 (-0.76, 2.56)	-0.34 (-1.90, 1.21)	1.11 (-0.47, 2.70)	2.21 (0.70, 3.73)	2.12 (0.63, 3.60)	-0.17 (-1.73, 1.39)	0.12 (-1.00, 1.24)	-0.12 (-1.86, 1.62)	-0.20 (-1.21, 0.81)	**PCEA tramadol**		
-1.53 (-3.45, 0.39)	1.06 (-0.16, 2.28)	-0.56 (-2.56, 1.45)	-1.80 (-3.73, 0.14)	-0.34 (-2.30, 1.61)	0.76 (-0.42, 1.93)	0.66 (-0.47, 1.79)	-1.63 (-3.47, 0.21)	-1.34 (-2.83, 0.15)	-1.58 (-3.64, 0.48)	-1.66 (-3.22, -0.09)	-1.46 (-3.32, 0.41)	**PCEA hydromorphone**	-1.33 (-2.43, -0.23)
-2.86 (-4.43, -1.29)	-0.27 (-0.79, 0.25)	-1.88 (-3.56, -0.20)	-3.13 (-4.72, -1.53)	-1.67 (-3.29, -0.05)	-0.57 (-0.98, -0.17)	-0.67 (-0.93, -0.41)	-2.96 (-4.43, -1.48)	-2.67 (-3.67, -1.66)	-2.91 (-4.64, -1.17)	-2.98 (-4.10, -1.87)	-2.78 (-4.29, -1.28)	-1.33 (-2.43, -0.23)	**IVPCA hydromorphone**

Comparisons between treatments should be read from left to right, and the estimate is in the cell in common between the column-defining treatment and the row-defining treatment. The network estimates from network meta-analysis are in the lower triangle, and the direct treatment estimates from pairwise comparisons are in the upper triangle. Estimates are presented as mean difference (95% confidence interval).

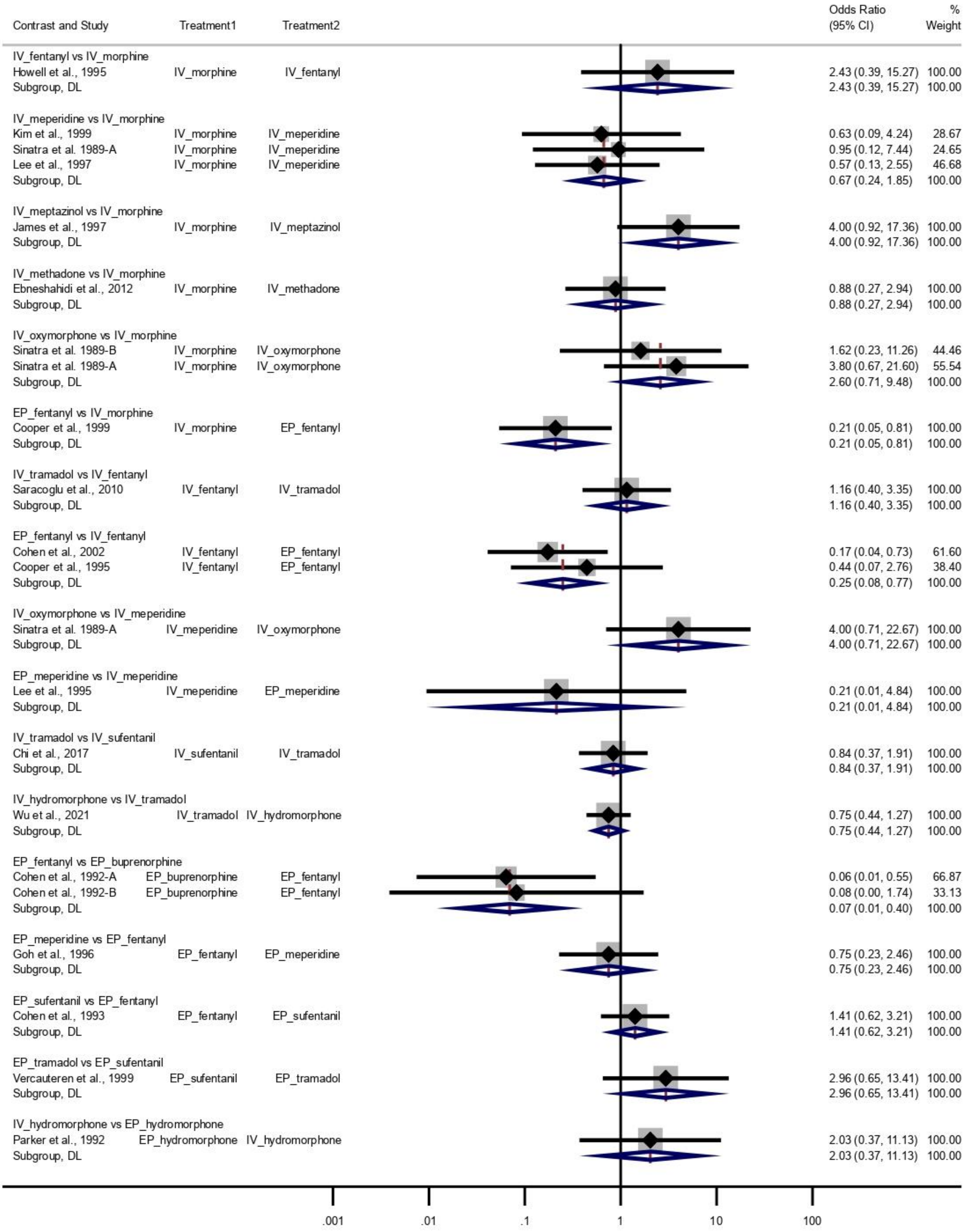

Supplementary Figure S9. Forest plots from pairwise comparisons of treatment agents on nausea/vomiting The prefix "IV_" denotes IVPCA, and the prefix "EP_" denotes PCEA. CI, confidence interval.

A.

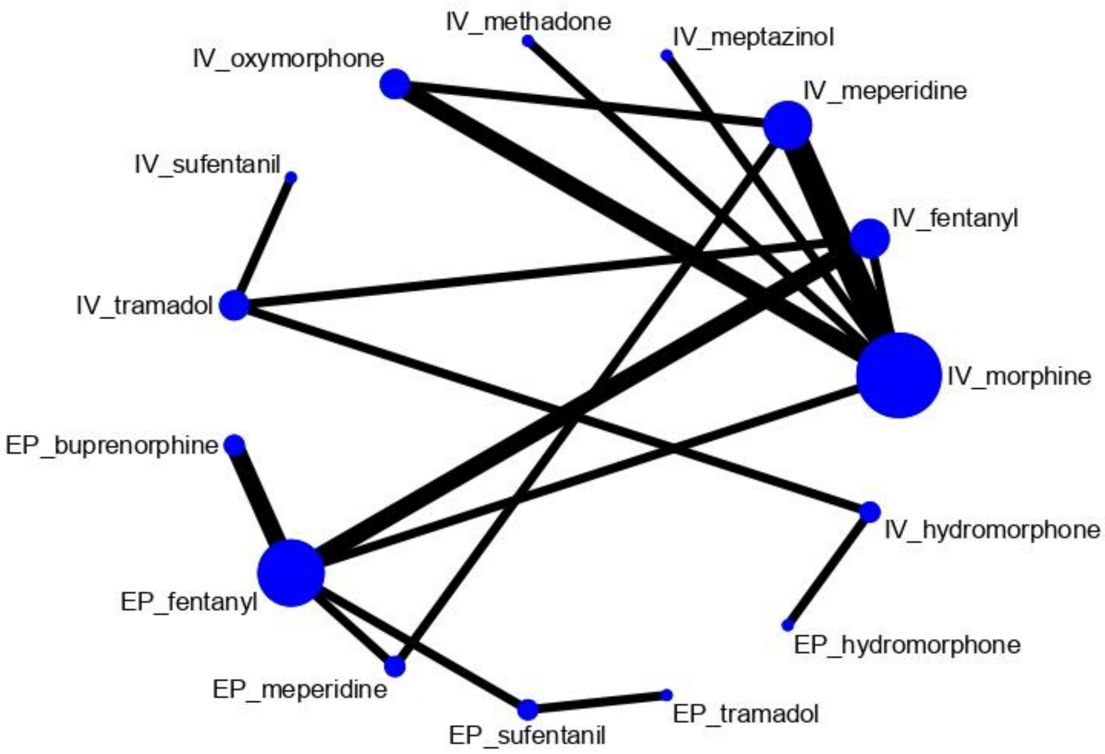
IV_methadone
IV_meptazinol
IV_oxymorphone
IV_meperidine
IV_sufentanil
IV_fentanyl
IV_tramadol
IV_morphine
EP_buprenorphine
IV_hydromorphone
EP_fentanyl
EP_hydromorphone
EP_meperidine
EP_tramadol
EP_sufentanil

B.

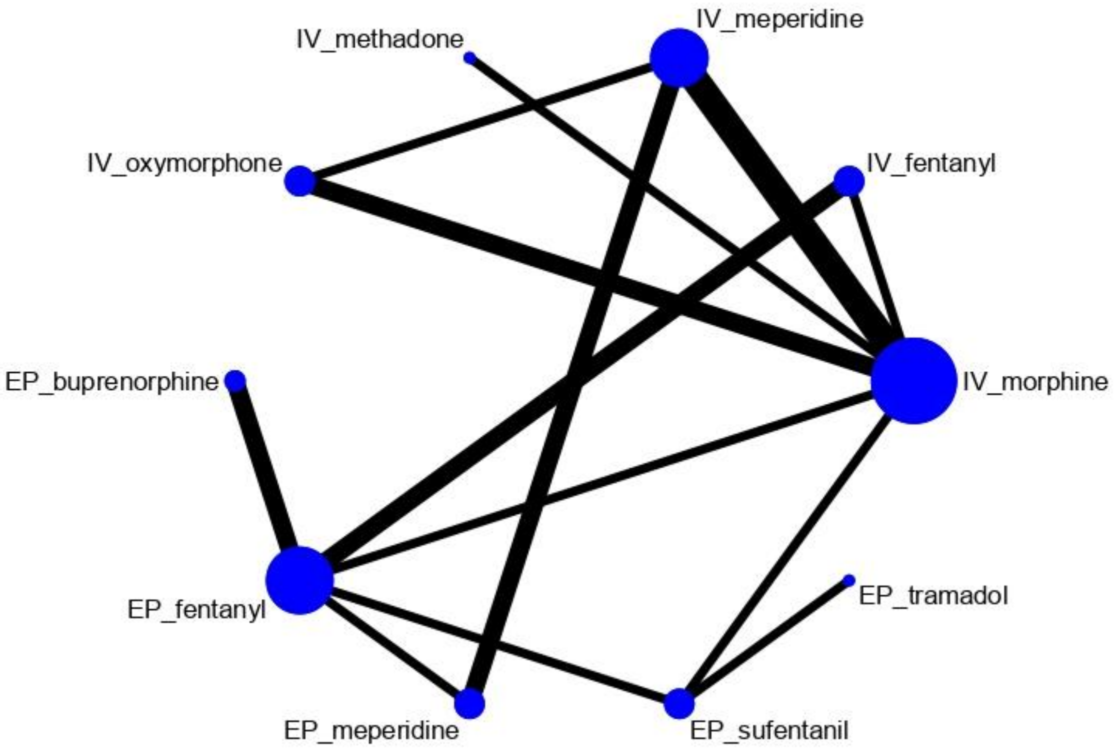
IV_meperidine
IV_methadone
IV_oxymorphone
IV_fentanyl
EP_buprenorphine
IV_morphine
EP_tramadol
EP_fentanyl
EP_meperidine
EP_sufentanil

C.

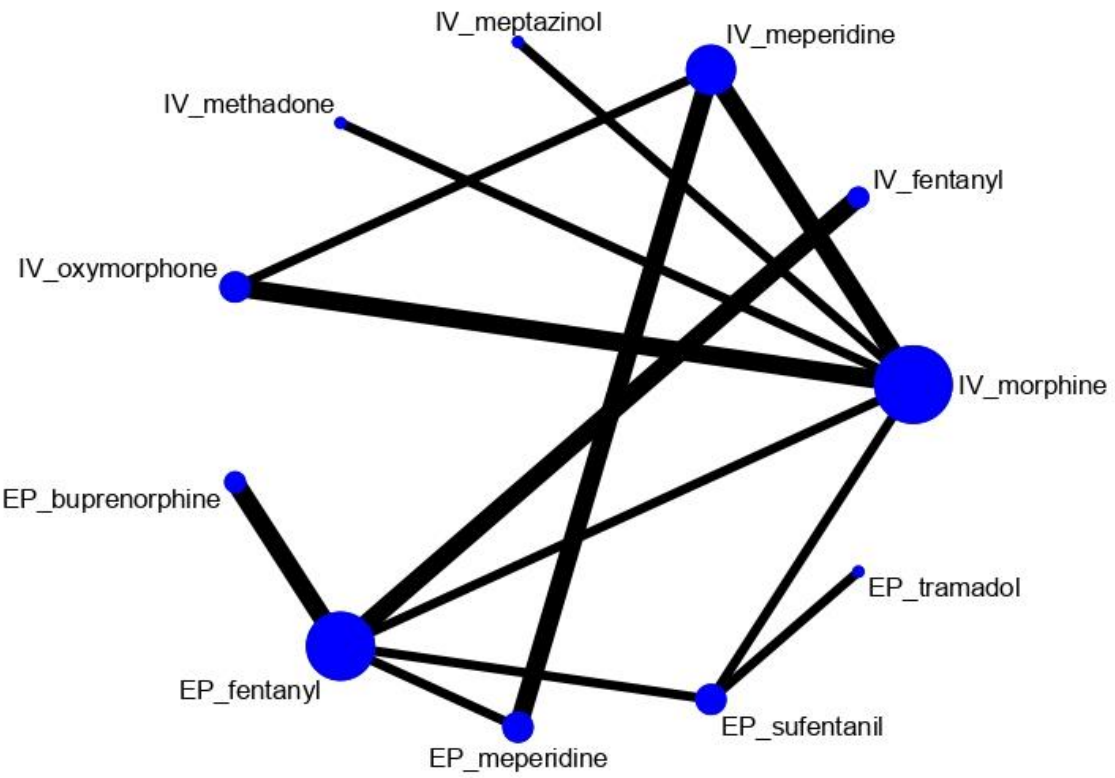

Supplementary Figure S10. Network treatment comparisons for studies investigating the effects of opioids on adverse effects

(A) nausea/vomiting; (B) pruritus; (C) sedation/drowsiness. Each node represents a treatment, the size of which corresponds to the number of patients studied with each treatment. Treatments that are compared directly are joined with a line, the thickness of which corresponds to the number of trials that assessed the comparisons. The prefix "IV_" denotes IVPCA, and the prefix "EP_" denotes PCEA.

Supplementary Table S7. Comparative efficacy of treatments on nausea/vomiting

**IVPCA morphine**	0.41 (0.07, 2.59)	1.50 (0.54, 4.17)	0.25 (0.06, 1.09)	1.13 (0.34, 3.77)	0.38 (0.11, 1.41)				4.78 (1.24, 18.49)					
0.74 (0.21, 2.57)	**IVPCA fentanyl**						0.86 (0.30, 2.50)	.	4.02 (1.30, 12.45)					
1.54 (0.59, 4.03)	2.08 (0.45, 9.60)	**IVPCA meperidine**			0.25 (0.04, 1.42)					4.67 (0.21, 111.11)				
0.25 (0.06, 1.09)	0.34 (0.05, 2.32)	0.16 (0.03, 0.94)	**IVPCA meptazinol**											
1.13 (0.34, 3.77)	1.53 (0.27, 8.64)	0.73 (0.16, 3.42)	4.52 (0.68, 30.19)	**IVPCA methadone**										
0.40 (0.12, 1.30)	0.54 (0.10, 2.96)	0.26 (0.07, 0.96)	1.60 (0.24, 10.50)	0.35 (0.07, 1.90)	**IVPCA oxymorphone**									
0.54 (0.09, 3.35)	0.72 (0.19, 2.78)	0.35 (0.05, 2.66)	2.14 (0.20, 22.42)	0.47 (0.05, 4.24)	1.34 (0.15, 11.71)	**IVPCA sufentanil**	1.19 (0.52, 2.73)							
0.64 (0.12, 3.28)	0.86 (0.30, 2.50)	0.41 (0.06, 2.67)	2.55 (0.28, 23.03)	0.56 (0.07, 4.31)	1.60 (0.22, 11.87)	1.19 (0.52, 2.73)	**IVPCA tramadol**							1.33 (0.79, 2.27)
0.26 (0.03, 2.04)	0.35 (0.05, 2.64)	0.17 (0.02, 1.57)	1.03 (0.08, 13.01)	0.23 (0.02, 2.49)	0.64 (0.06, 6.87)	0.48 (0.04, 5.48)	0.40 (0.04, 3.98)	**PCEA buprenorphine**	14.29 (2.48, 83.33)					

3.71 (1.25, 11.01)	5.01 (1.83, 13.75)	2.41 (0.60, 9.60)	14.83 (2.38, 92.21)	3.28 (0.65, 16.60)	9.27 (1.90, 45.21)	6.93 (1.29, 37.23)	5.80 (1.34, 25.11)	14.39 (2.48, 83.41)	**PCEA fentanyl**	1.33 (0.41, 4.39)	0.71 (0.31, 1.61)			
5.18 (1.19, 22.65)	7.01 (1.59, 30.98)	3.37 (0.65, 17.37)	20.74 (2.59, 166.12)	4.58 (0.68, 30.74)	12.97 (2.04, 82.65)	9.69 (1.31, 71.90)	8.12 (1.31, 50.41)	20.12 (2.50, 162.13)	1.40 (0.45, 4.31)	**PCEA meperidine**				
2.62 (0.67, 10.25)	3.55 (0.97, 13.02)	1.70 (0.34, 8.51)	10.49 (1.41, 77.80)	2.32 (0.38, 14.29)	6.56 (1.10, 39.08)	4.90 (0.75, 31.84)	4.11 (0.77, 22.02)	10.18 (1.46, 70.82)	0.71 (0.31, 1.61)	0.51 (0.13, 2.04)	**PCEA sufentanil**	0.34 (0.07, 1.54)		
0.89 (0.12, 6.80)	1.20 (0.16, 8.82)	0.58 (0.06, 5.24)	3.55 (0.29, 43.69)	0.78 (0.07, 8.35)	2.22 (0.21, 23.03)	1.66 (0.15, 18.39)	1.39 (0.15, 13.31)	3.44 (0.29, 40.30)	0.24 (0.04, 1.34)	0.17 (0.02, 1.34)	0.34 (0.07, 1.54)	**PCEA tramadol**		
1.74 (0.15, 19.49)	2.35 (0.30, 18.65)	1.13 (0.09, 14.80)	6.94 (0.41, 117.55)	1.53 (0.10, 22.86)	4.34 (0.30, 63.44)	3.24 (0.46, 23.08)	2.72 (0.46, 16.12)	6.74 (0.37, 122.29)	0.47 (0.05, 4.70)	0.33 (0.03, 4.29)	0.66 (0.06, 7.65)	1.96 (0.11, 34.73)	**PCEA hydromorphone**	0.49 (0.09, 2.69)
0.85 (0.15, 4.77)	1.15 (0.35, 3.78)	0.55 (0.08, 3.83)	3.41 (0.36, 32.76)	0.75 (0.09, 6.16)	2.13 (0.27, 16.99)	1.59 (0.60, 4.25)	1.34 (0.79, 2.27)	3.31 (0.32, 34.66)	0.23 (0.05, 1.09)	0.16 (0.02, 1.10)	0.33 (0.06, 1.89)	0.96 (0.09, 9.79)	0.49 (0.09, 2.69)	**IVPCA hydromorphone**

Comparisons between treatments should be read from left to right, and the estimate is in the cell in common between the column-defining treatment and the row-defining treatment. The network estimates from network meta-analysis are in the lower triangle, and the direct treatment estimates from pairwise comparisons are in the upper triangle. Estimates are presented as odds ratio (95% confidence interval).

A.

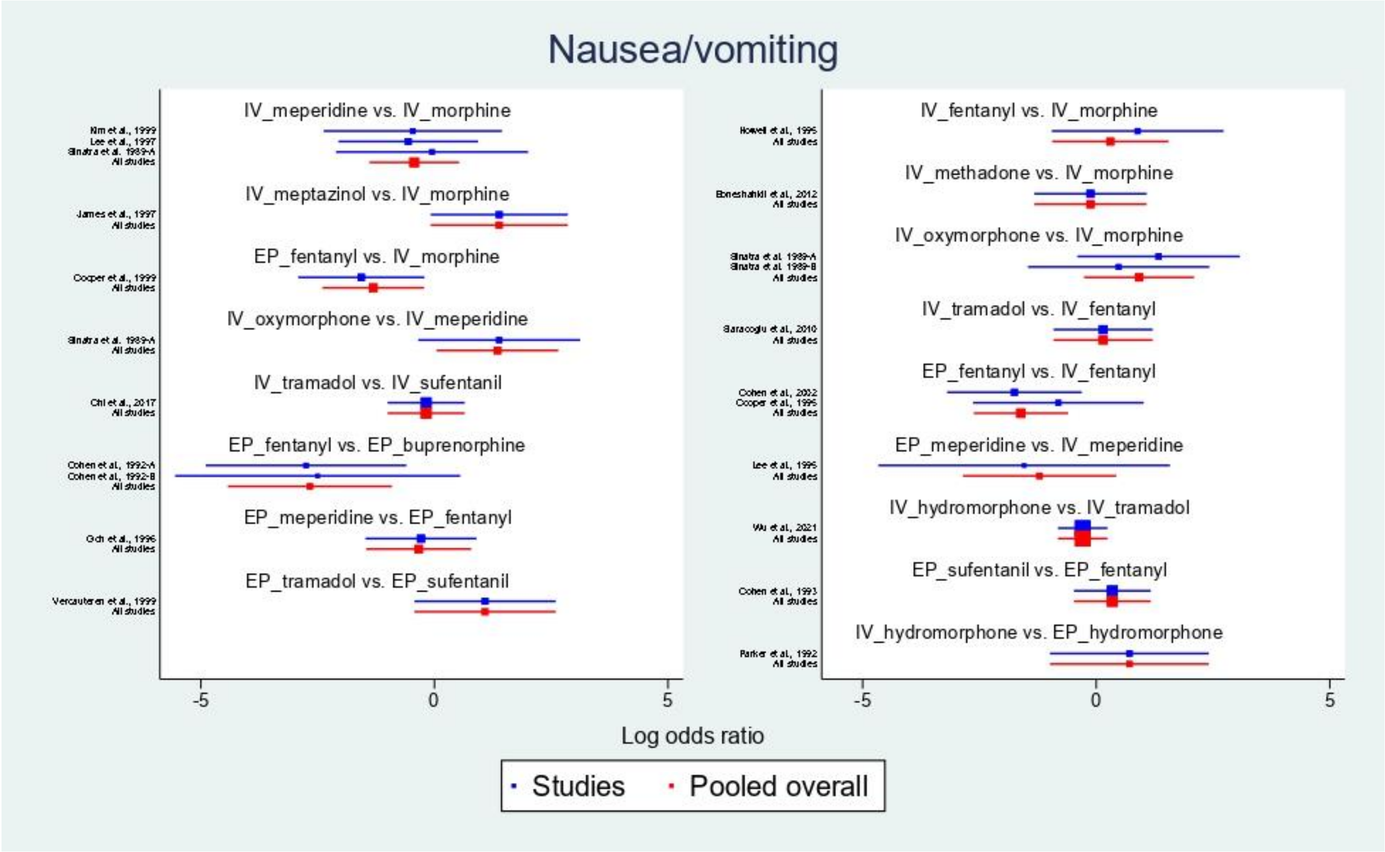
Nausea/vomiting
IV_meperidine vs. IV_morphine
IV_meptazinol vs. IV_morphine
EP_fentanyl vs. IV_morphine
IV_oxymorphone vs. IV_meperidine
IV_tramadol vs. IV_sufentanil
EP_fentanyl vs. EP_buprenorphine
EP_meperidine vs. EP_fentanyl
EP_tramadol vs. EP_sufentanil
IV_fentanyl vs. IV_morphine
IV_methadone vs. IV_morphine
IV_oxymorphone vs. IV_morphine
IV_tramadol vs. IV_fentanyl
EP_fentanyl vs. IV_fentanyl
EP_meperidine vs. IV_meperidine
IV_hydromorphone vs. IV_tramadol
EP_sufentanil vs. EP_fentanyl
IV_hydromorphone vs. EP_hydromorphone
-5
0
5
Log odds ratio
Studies
Pooled overall

B.

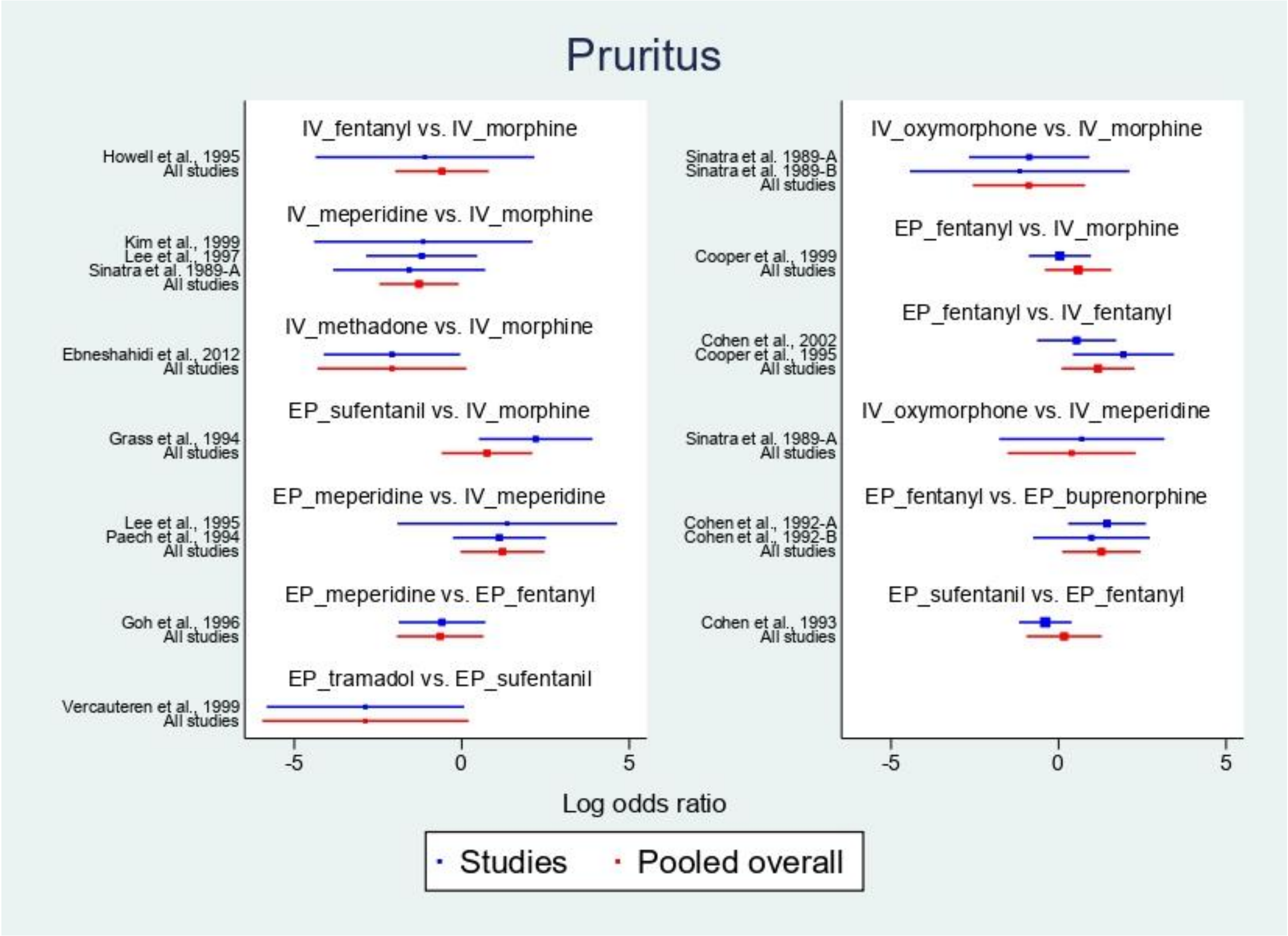
Pruritus
IV_fentanyl vs. IV_morphine
Howell et al., 1995
All studies
IV_meperidine vs. IV_morphine
Kim et al., 1999
Lee et al., 1997
Sinatra et al. 1989-A
All studies
IV_methadone vs. IV_morphine
Ebneshahidi et al., 2012
All studies
EP_sufentanil vs. IV_morphine
Grass et al., 1994
All studies
EP_meperidine vs. IV_meperidine
Lee et al., 1995
Paech et al., 1994
All studies
EP_meperidine vs. EP_fentanyl
Goh et al., 1996
All studies
EP_tramadol vs. EP_sufentanil
Vercauteren et al., 1999
All studies
IV_oxymorphone vs. IV_morphine
Sinatra et al. 1989-A
Sinatra et al. 1989-B
All studies
EP_fentanyl vs. IV_morphine
Cooper et al., 1999
All studies
EP_fentanyl vs. IV_fentanyl
Cohen et al., 2002
Cooper et al., 1995
All studies
IV_oxymorphone vs. IV_meperidine
Sinatra et al. 1989-A
All studies
EP_fentanyl vs. EP_buprenorphine
Cohen et al., 1992-A
Cohen et al., 1992-B
All studies
EP_sufentanil vs. EP_fentanyl
Cohen et al., 1993
All studies
-5
0
5
Log odds ratio
Studies
Pooled overall

C.

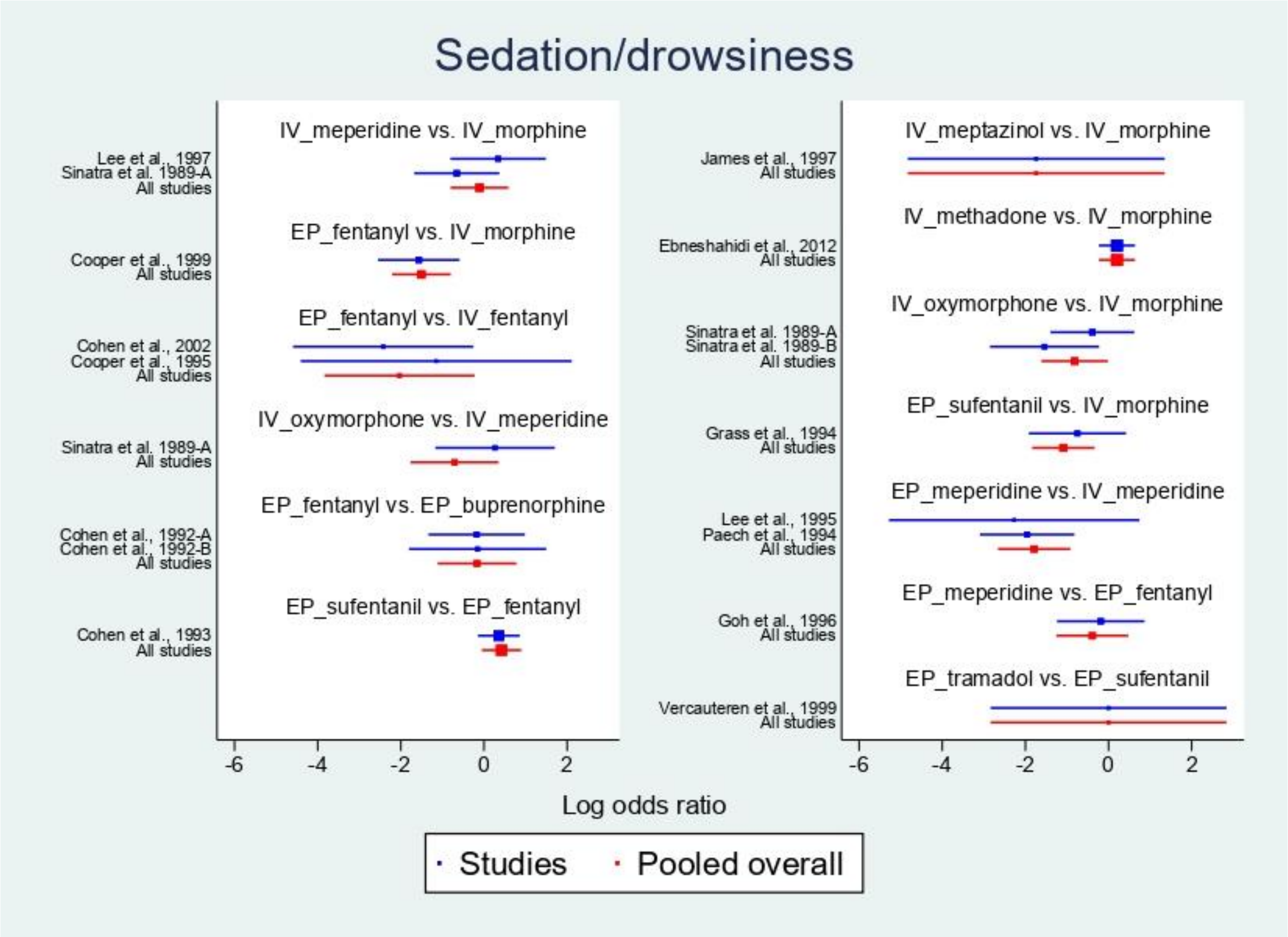

Supplementary Figure S11. Network meta-analysis forest plots of adverse effects (A) nausea/vomiting; (B) pruritus; (C) sedation/drowsiness. The blue dots and lines indicate the studies contributing the direct evidence. The red dots and lines indicate the pooled overall network estimates of the treatment effects. The prefix “IV_” denotes IVPCA, and the prefix “EP_” denotes PCEA.

A.

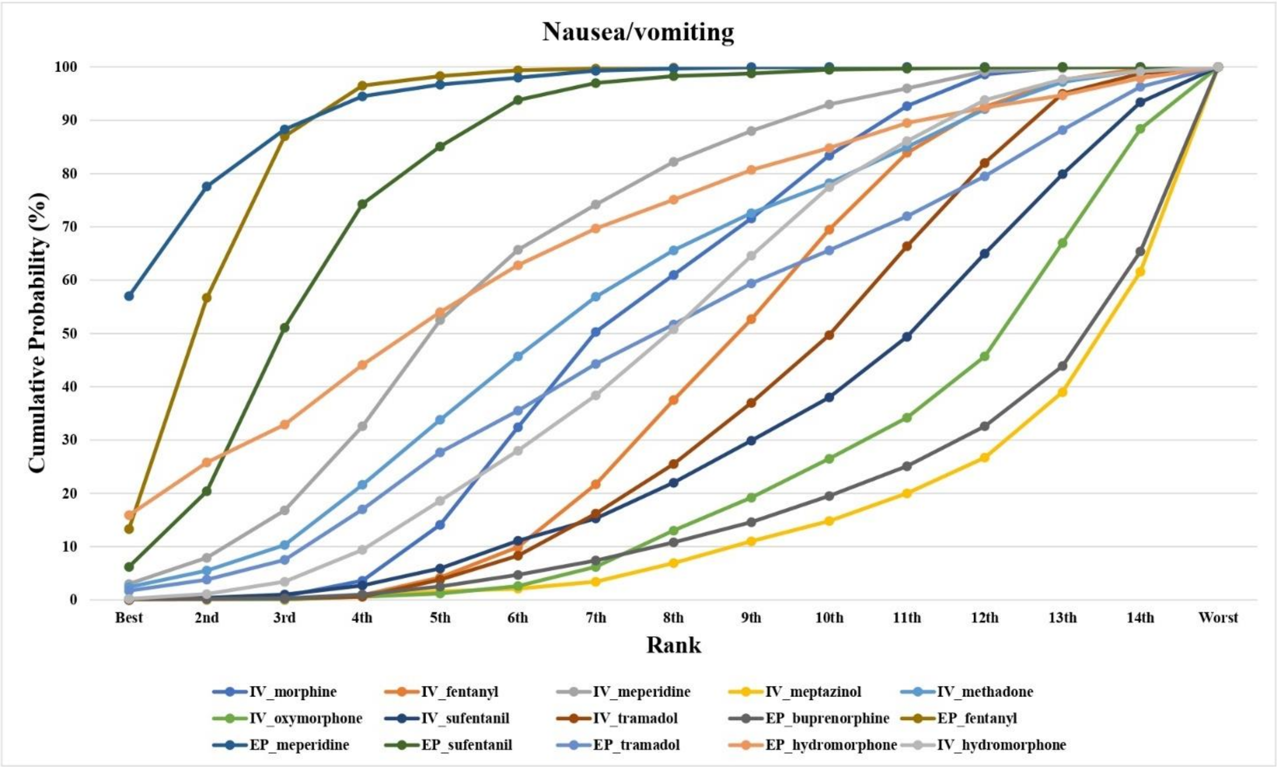
Nausea/vomiting
Cumulative Probability (%)
Rank
Best
2nd
3rd
4th
5th
6th
7th
8th
9th
10th
11th
12th
13th
14th
Worst
IV_morphine
IV_fentanyl
IV_meperidine
IV_meptazinol
IV_methadone
IV_oxymorphone
IV_sufentanil
IV_tramadol
EP_buprenorphine
EP_fentanyl
EP_meperidine
EP_sufentanil
EP_tramadol
EP_hydromorphone
IV_hydromorphone

B.

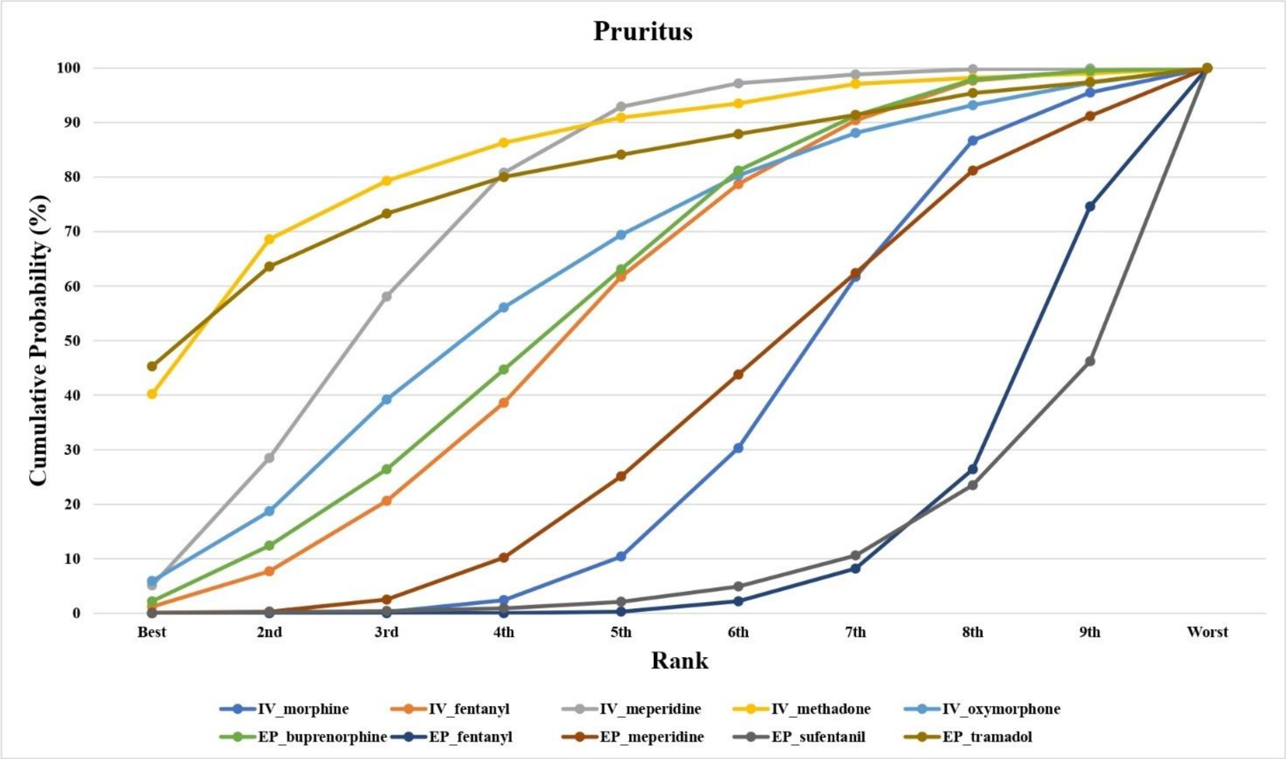
Pruritus
Cumulative Probability (%)
Rank
Best
2nd
3rd
4th
5th
6th
7th
8th
9th
Worst
IV_morphine
IV_fentanyl
IV_meperidine
IV_methadone
IV_oxymorphone
EP_buprenorphine
EP_fentanyl
EP_meperidine
EP_sufentanil
EP_tramadol

C.

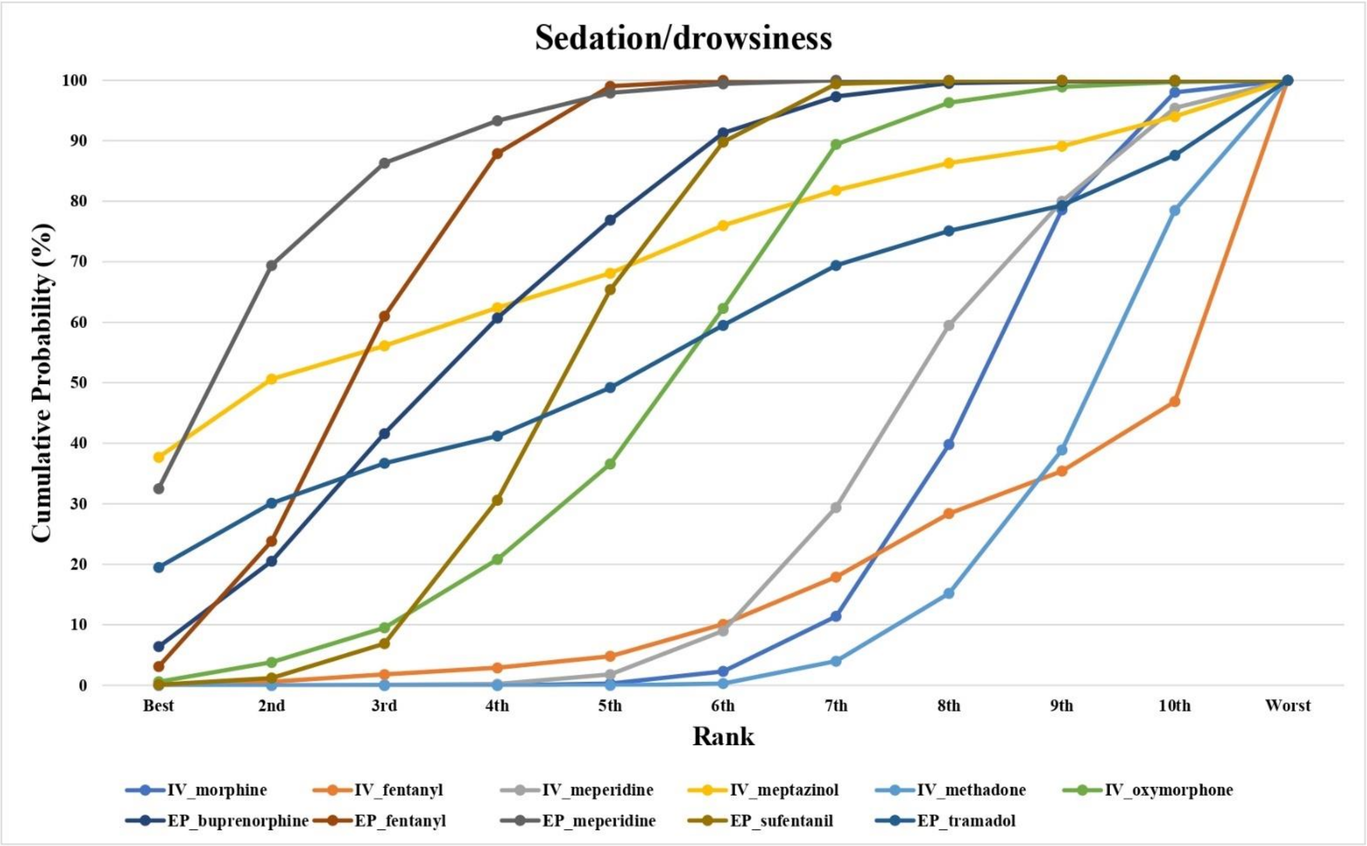

Supplementary Figure S12. Cumulative ranking probability of the effects of each treatment on adverse effects

(A) nausea/vomiting; (B) pruritus; (C) sedation/drowsiness. The prefix "IV_" denotes IVPCA, and the prefix "EP_" denotes PCEA.

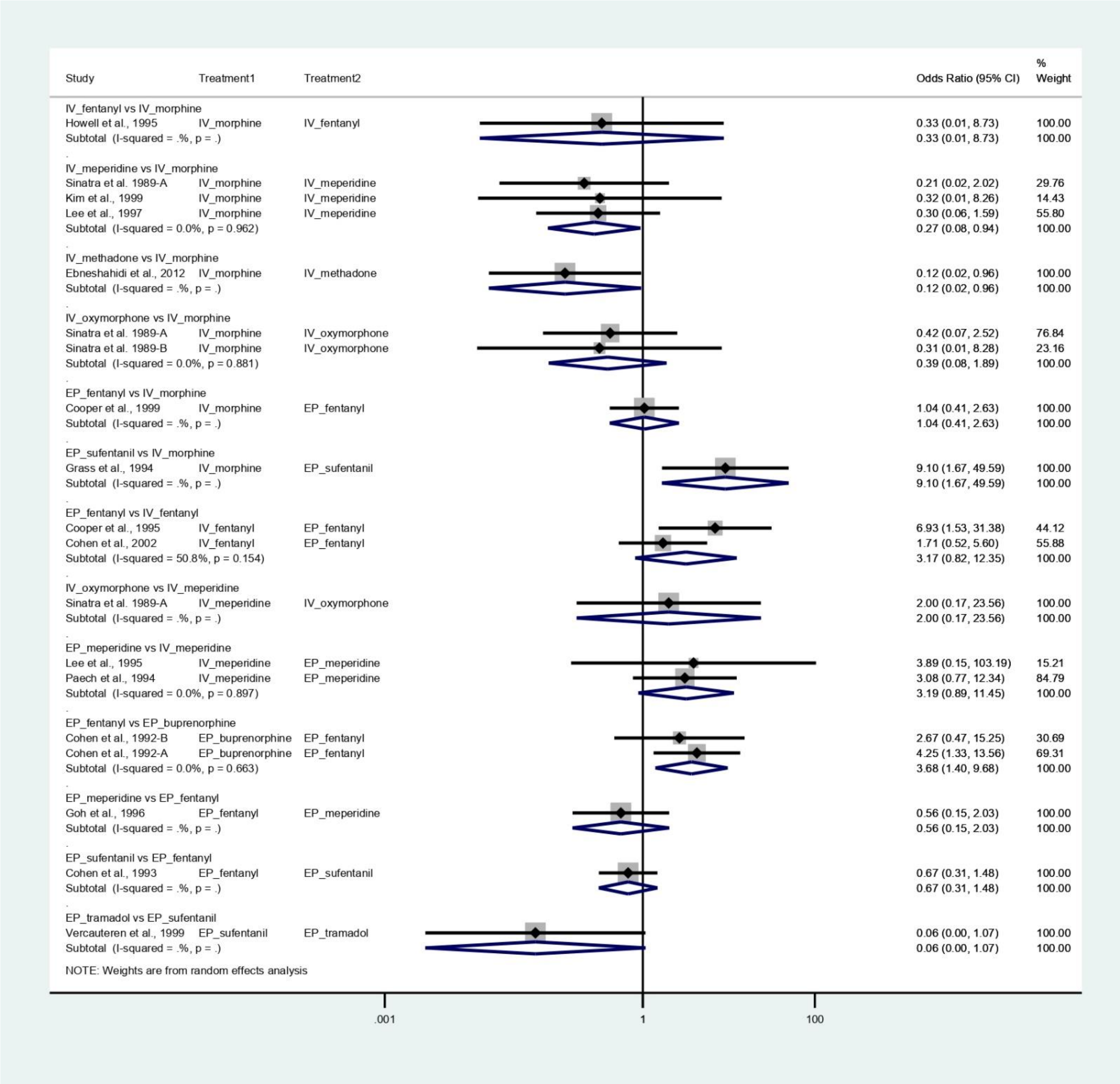

Supplementary Figure S13. Forest plots from pairwise comparisons of treatment agents on pruritus The prefix “IV_” denotes IVPCA, and the prefix “EP_” denotes PCEA. CI, confidence interval.

Supplementary Table S8. Comparative efficacy of treatments on pruritus

**IVPCA morphine**	3.00 (0.11, 76.92)	3.68 (1.06, 12.66)	8.00 (1.04, 62.50)	2.56 (0.53, 12.35)		0.96 (0.38, 2.43)		0.11 (0.02, 0.60)	
1.81 (0.45, 7.28)	**IVPCA fentanyl**					0.32 (0.08, 1.23)			
3.58 (1.09, 11.69)	1.98 (0.37, 10.72)	**IVPCA meperidine**		0.50 (0.04, 5.88)			0.31 (0.09, 1.13)		
8.03 (0.87, 74.43)	4.44 (0.32, 61.47)	2.24 (0.18, 27.95)	**IVPCA methadone**						
2.42 (0.45, 12.98)	1.34 (0.16, 11.62)	0.68 (0.10, 4.59)	0.30 (0.02, 4.91)	**IVPCA oxymorphone**					
2.00 (0.43, 9.39)	1.11 (0.22, 5.54)	0.56 (0.09, 3.38)	0.25 (0.02, 3.75)	0.82 (0.09, 7.89)	**PCEA buprenorphine**	0.27 (0.1, 0.71)			
0.56 (0.21, 1.51)	0.31 (0.10, 0.92)	0.16 (0.04, 0.60)	0.07 (0.01, 0.79)	0.23 (0.03, 1.57)	0.28 (0.09, 0.90)	**PCEA fentanyl**	1.8 (0.49, 6.58)	1.48 (0.68, 3.25)	
1.06 (0.26, 4.23)	0.59 (0.11, 3.10)	0.30 (0.08, 1.04)	0.13 (0.01, 1.82)	0.44 (0.05, 3.55)	0.53 (0.09, 3.05)	1.90 (0.52, 6.96)	**PCEA meperidine**		
0.47 (0.12, 1.82)	0.26 (0.05, 1.28)	0.13 (0.02, 0.71)	0.06 (0.00, 0.79)	0.19 (0.02, 1.65)	0.24 (0.05, 1.18)	0.85 (0.27, 2.61)	0.44 (0.08, 2.37)	**PCEA sufentanil**	17.86 (0.93, 333.33)
8.34 (0.29, 241.11)	4.62 (0.14, 147.73)	2.33 (0.07, 77.90)	1.04 (0.02, 58.72)	3.44 (0.08, 146.09)	4.17 (0.13, 134.80)	15.00 (0.57, 397.74)	7.88 (0.24, 261.96)	17.73 (0.82, 385.32)	**PCEA tramadol**

Comparisons between treatments should be read from left to right, and the estimate is in the cell in common between the column-defining treatment and the row-defining treatment. The network estimates from network meta-analysis are in the lower triangle, and the direct treatment estimates from pairwise comparisons are in the upper triangle. Estimates are presented as odds ratio (95% confidence interval).

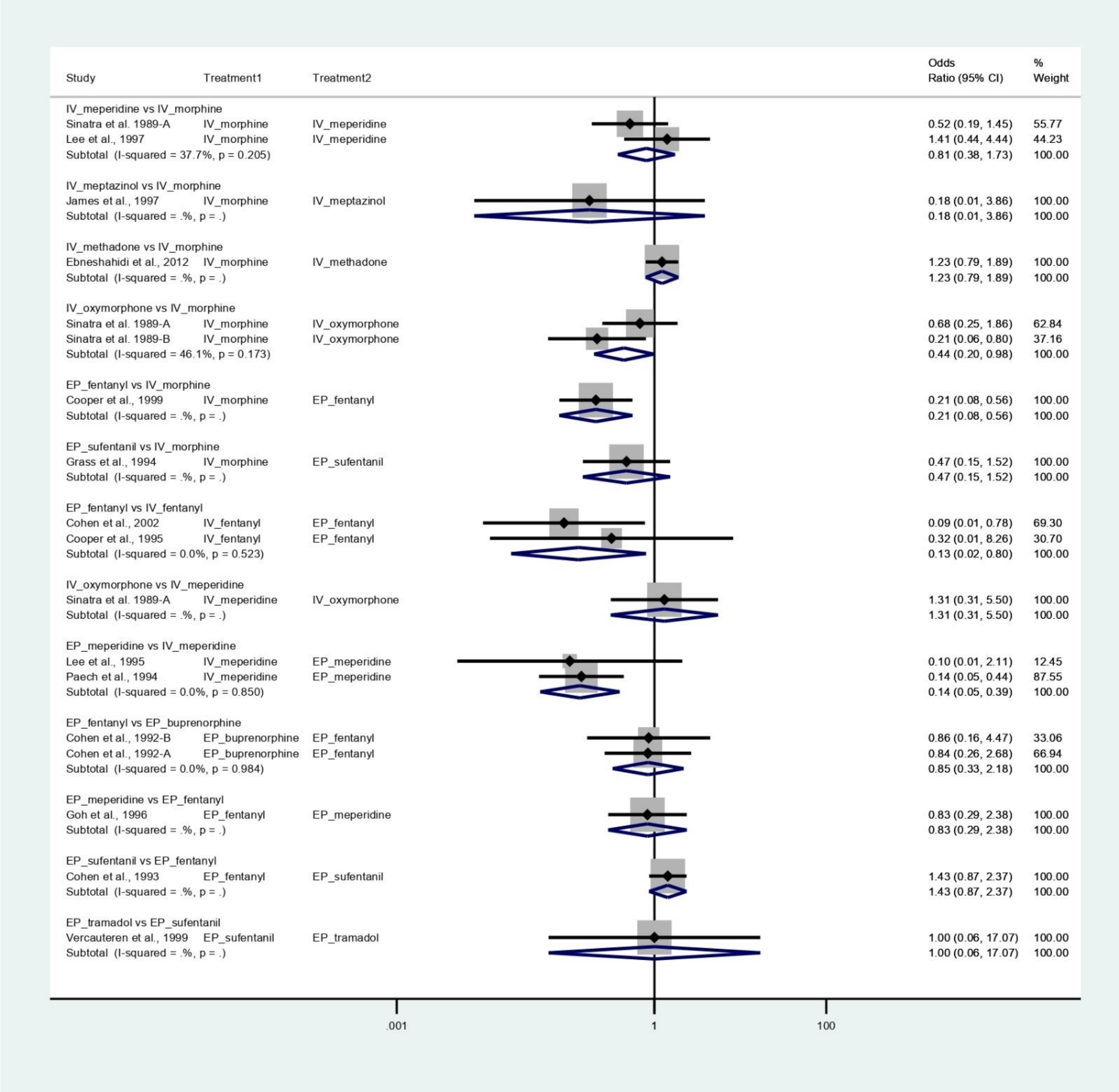

Supplementary Figure S14. Forest plots from pairwise comparisons of treatment agents on sedation/drowsiness

The prefix “IV_” denotes IVPCA, and the prefix “EP_” denotes PCEA. CI, confidence interval.

Supplementary Table S9. Comparative efficacy of treatments on sedation/drowsiness

**IVPCA morphine**		1.24 (0.58, 2.67)	5.68 (0.26, 125)	0.82 (0.53, 1.26)	2.26 (1.02, 5.03)		4.81 (1.80, 12.82)		2.11 (0.66, 6.80)	
0.59 (0.09, 4.11)	**IVPCA fentanyl**						7.63 (1.25, 45.45)			
1.11 (0.56, 2.24)	1.88 (0.25, 14.06)	**IVPCA meperidine**			0.77 (0.18, 3.23)			7.35 (2.54, 21.28)		
5.70 (0.26, 125.36)	9.64 (0.25, 370.36)	5.12 (0.22, 121.62)	**IVPCA meptazinol**							
0.82 (0.53, 1.26)	1.38 (0.19, 10.07)	0.73 (0.32, 1.67)	0.14 (0.01, 3.25)	**IVPCA methadone**						
2.26 (1.02, 5.03)	3.83 (0.47, 31.16)	2.03 (0.70, 5.87)	0.40 (0.02, 9.67)	2.77 (1.11, 6.89)	**IVPCA oxymorphone**					
3.82 (1.17, 12.45)	6.45 (0.84, 49.66)	3.43 (0.94, 12.51)	0.67 (0.02, 18.33)	4.68 (1.33, 16.49)	1.69 (0.40, 7.03)	**PCEA buprenorphine**	1.18 (0.46, 3.06)			
4.51 (2.23, 9.14)	7.63 (1.25, 46.48)	4.05 (1.68, 9.78)	0.79 (0.03, 18.87)	5.53 (2.42, 12.67)	2.00 (0.69, 5.80)	1.18 (0.46, 3.06)	**PCEA fentanyl**	1.20 (0.42, 3.45)	0.70 (0.42, 1.15)	
6.66 (2.64, 16.82)	11.27 (1.52, 83.55)	5.98 (2.51, 14.29)	1.17 (0.05, 29.47)	8.17 (2.94, 22.72)	2.95 (0.87, 10.02)	1.75 (0.48, 6.31)	1.48 (0.62, 3.51)	**PCEA meperidine**		
2.96 (1.40, 6.29)	5.01 (0.77, 32.43)	2.66 (1.04, 6.79)	0.52 (0.02, 12.52)	3.63 (1.52, 8.67)	1.31 (0.44, 3.93)	0.78 (0.27, 2.24)	0.66 (0.41, 1.05)	0.44 (0.17, 1.16)	**PCEA sufentanil**	1.00 (0.06, 16.95)
2.96 (0.16, 55.78)	5.01 (0.17, 149.61)	2.66 (0.13, 52.78)	0.52 (0.01, 36.92)	3.63 (0.19, 70.59)	1.31 (0.06, 27.45)	0.78 (0.04, 16.05)	0.66 (0.04, 11.65)	0.44 (0.02, 8.87)	1.00 (0.06, 17.07)	**PCEA tramadol**

Comparisons between treatments should be read from left to right, and the estimate is in the cell in common between the column-defining treatment and the row-defining treatment. The network estimates from network meta-analysis are in the lower triangle, and the direct treatment estimates from pairwise comparisons are in the upper triangle. Estimates are presented as odds ratio (95% confidence interval).

A.

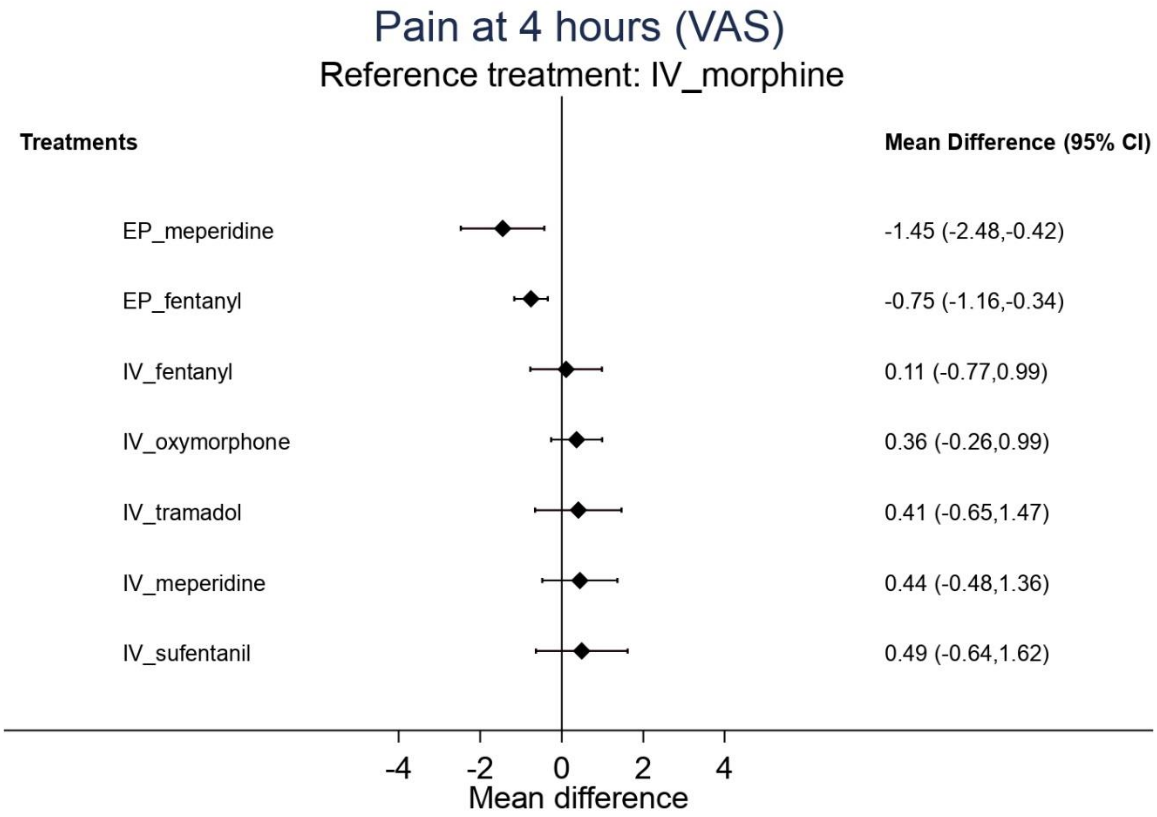
Pain at 4 hours (VAS)
Reference treatment: IV_morphine
Treatments
Mean Difference (95% CI)
EP_meperidine
-1.45 (-2.48,-0.42)
EP_fentanyl
-0.75 (-1.16,-0.34)
IV_fentanyl
0.11 (-0.77,0.99)
IV_oxymorphone
0.36 (-0.26,0.99)
IV_tramadol
0.41 (-0.65,1.47)
IV_meperidine
0.44 (-0.48,1.36)
IV_sufentanil
0.49 (-0.64,1.62)
-4
-2
0
2
4
Mean difference

B.

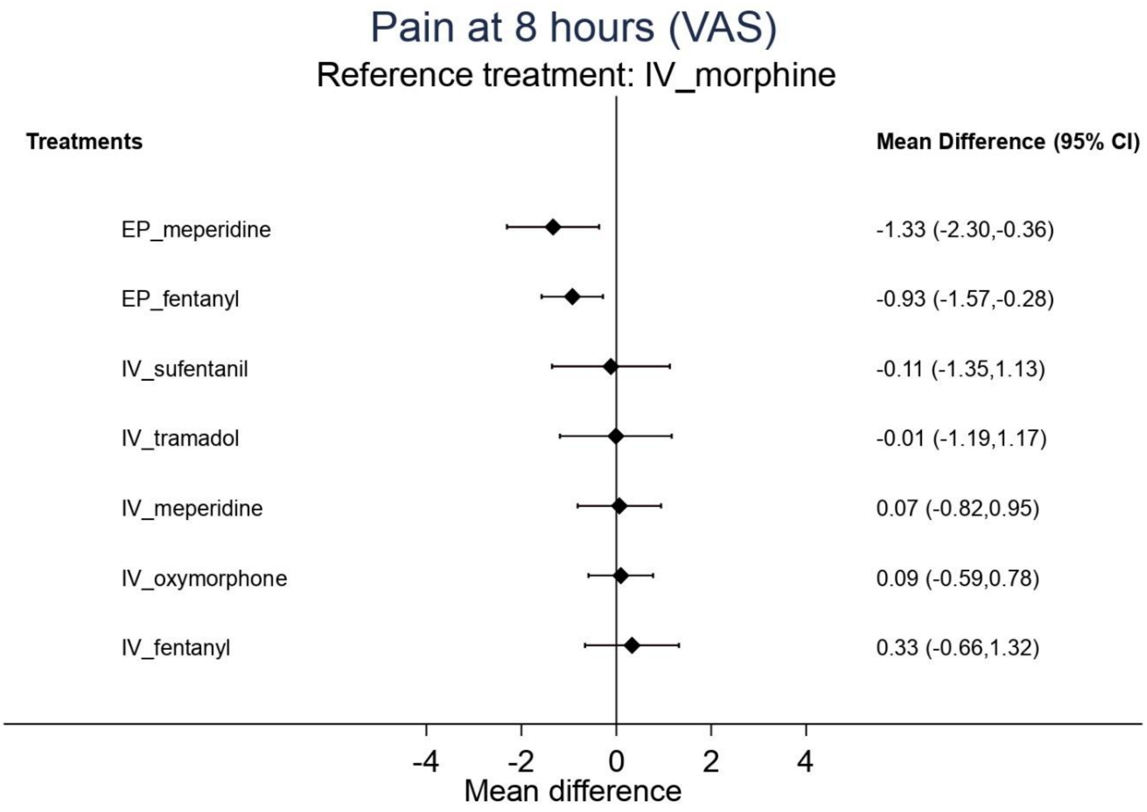
Pain at 8 hours (VAS)
Reference treatment: IV_morphine
Treatments
Mean Difference (95% CI)
EP_meperidine
-1.33 (-2.30,-0.36)
EP_fentanyl
-0.93 (-1.57,-0.28)
IV_sufentanil
-0.11 (-1.35,1.13)
IV_tramadol
-0.01 (-1.19,1.17)
IV_meperidine
0.07 (-0.82,0.95)
IV_oxymorphone
0.09 (-0.59,0.78)
IV_fentanyl
0.33 (-0.66,1.32)
-4
-2
0
2
4
Mean difference

C.

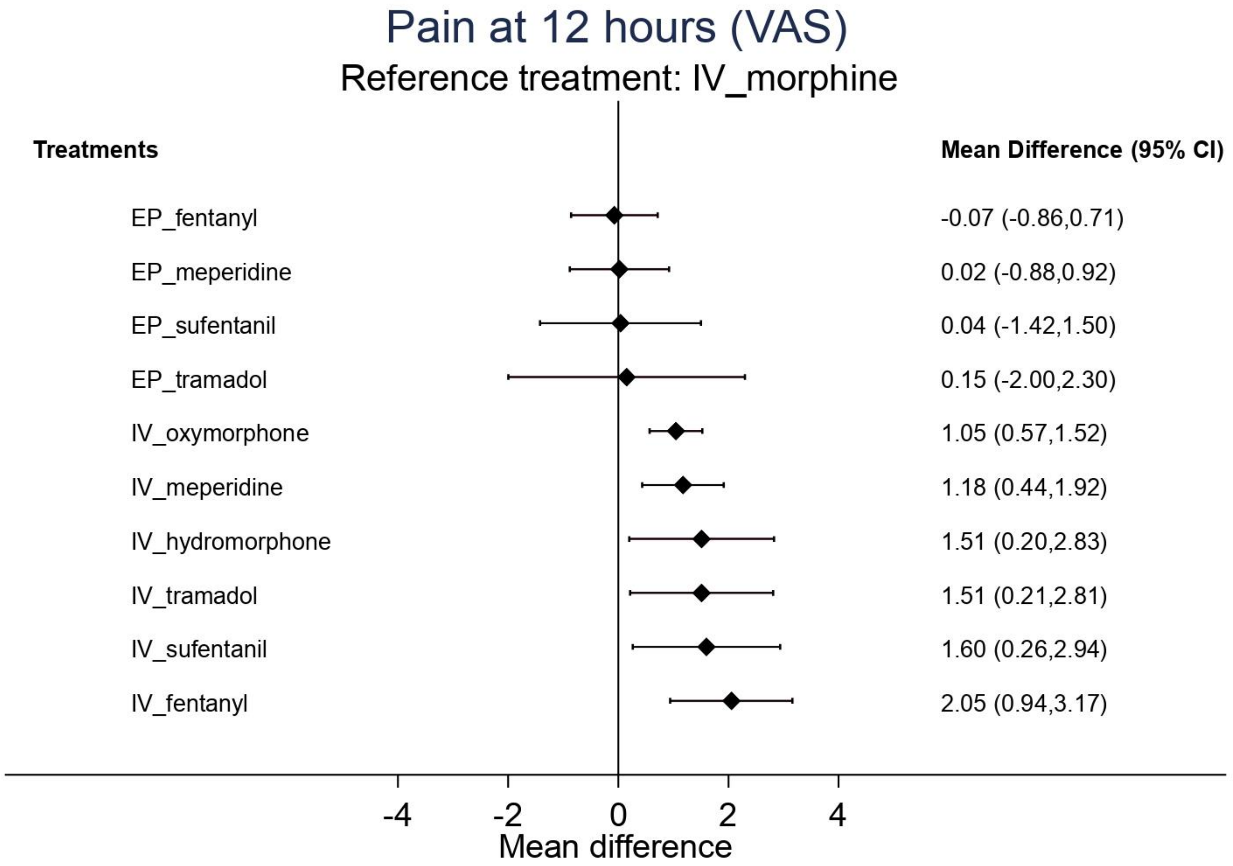

Supplementary Figure S15. Sensitivity analysis of pain intensity

(A) Forest plot of the effects of each treatment relative to IVPCA morphine at 4 hours after surgery. (B) Forest plot of the effects of each treatment relative to IVPCA morphine at 8 hours after surgery. (C) Forest plot of the effects of each treatment relative to IVPCA morphine at 12 hours after surgery.

A.

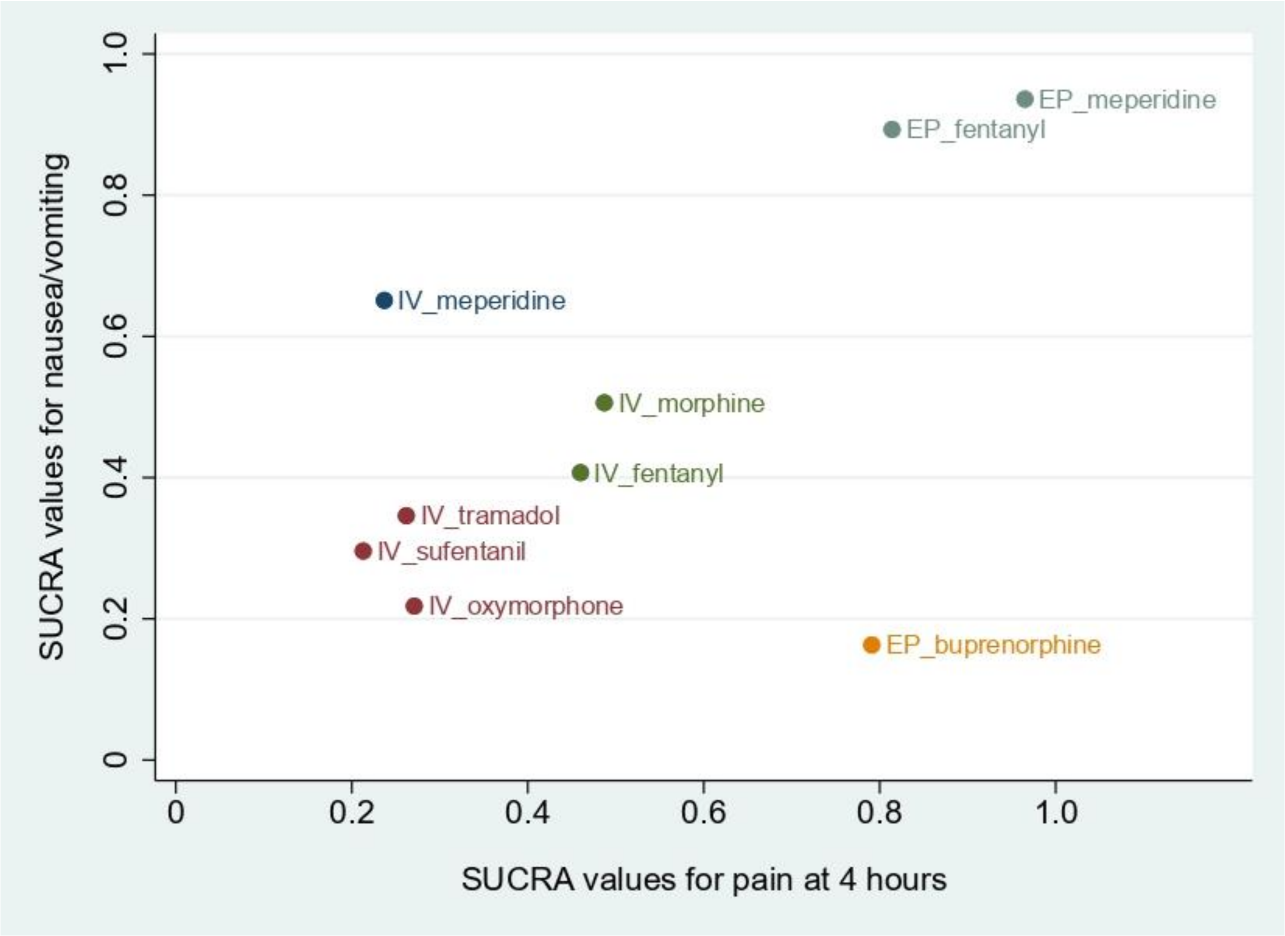
EP_meperidine
EP_fentanyl
IV_meperidine
IV_morphine
IV_fentanyl
IV_tramadol
IV_sufentanil
IV_oxymorphone
EP_buprenorphine
SUCRA values for nausea/vomiting
SUCRA values for pain at 4 hours
0
0.2
0.4
0.6
0.8
1.0

B.

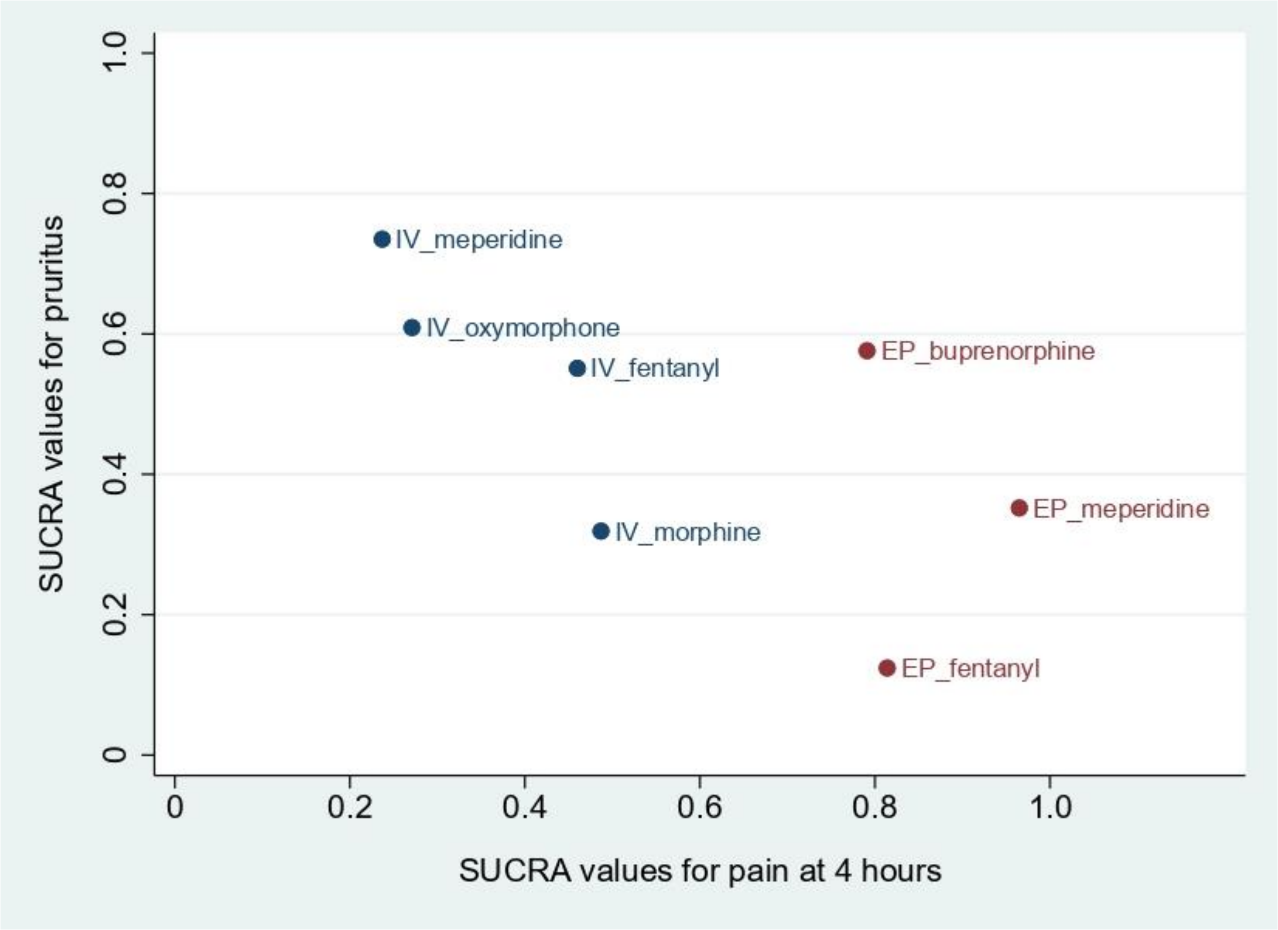
IV_meperidine
IV_oxymorphone
IV_fentanyl
EP_buprenorphine
EP_meperidine
IV_morphine
EP_fentanyl
SUCRA values for pruritus
SUCRA values for pain at 4 hours
0
0.2
0.4
0.6
0.8
1.0

C.

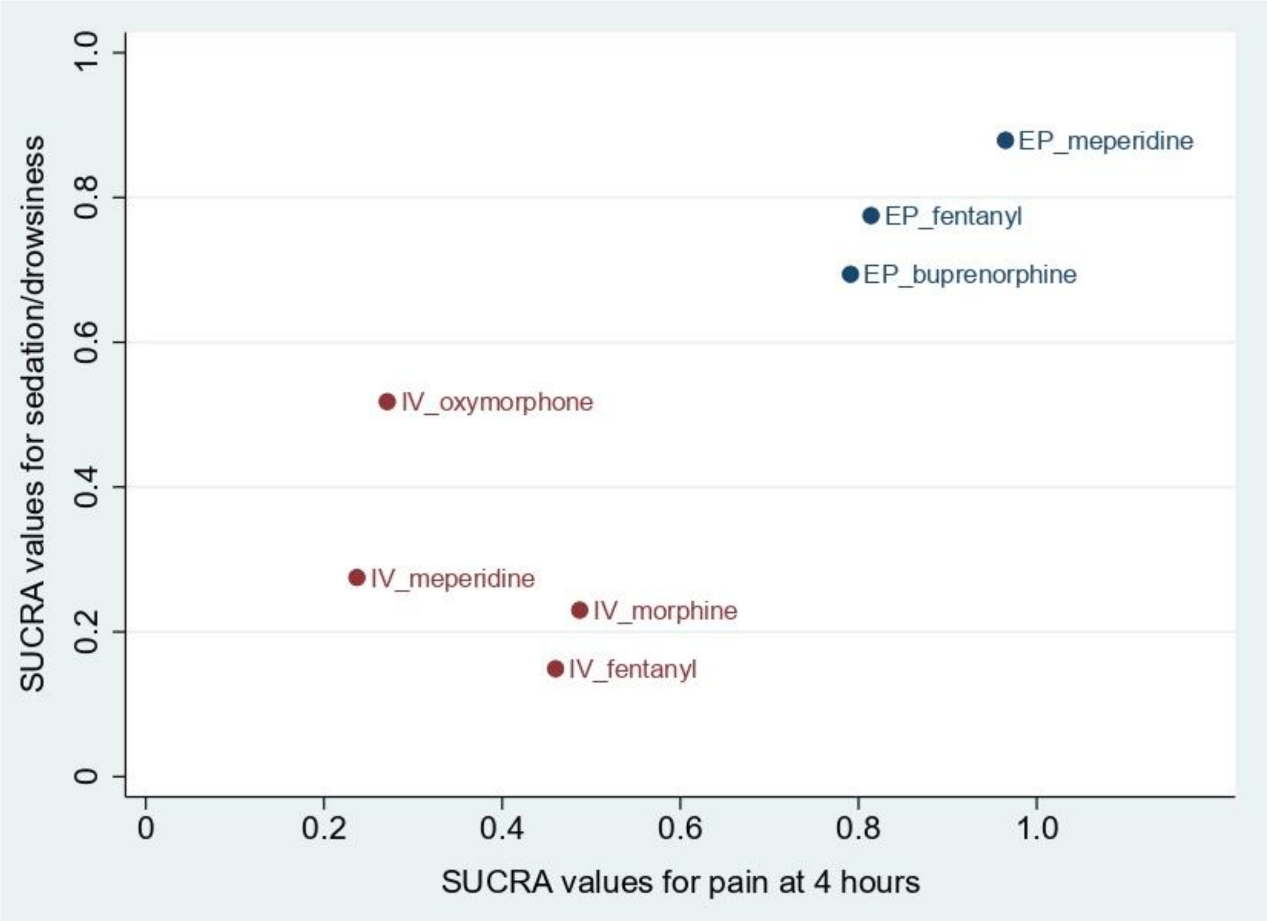
SUCRA values for sedation/drowsiness
SUCRA values for pain at 4 hours
0
0.2
0.4
0.6
0.8
1.0
EP_meperidine
EP_fentanyl
EP_buprenorphine
IV_oxymorphone
IV_meperidine
IV_morphine
IV_fentanyl

D.

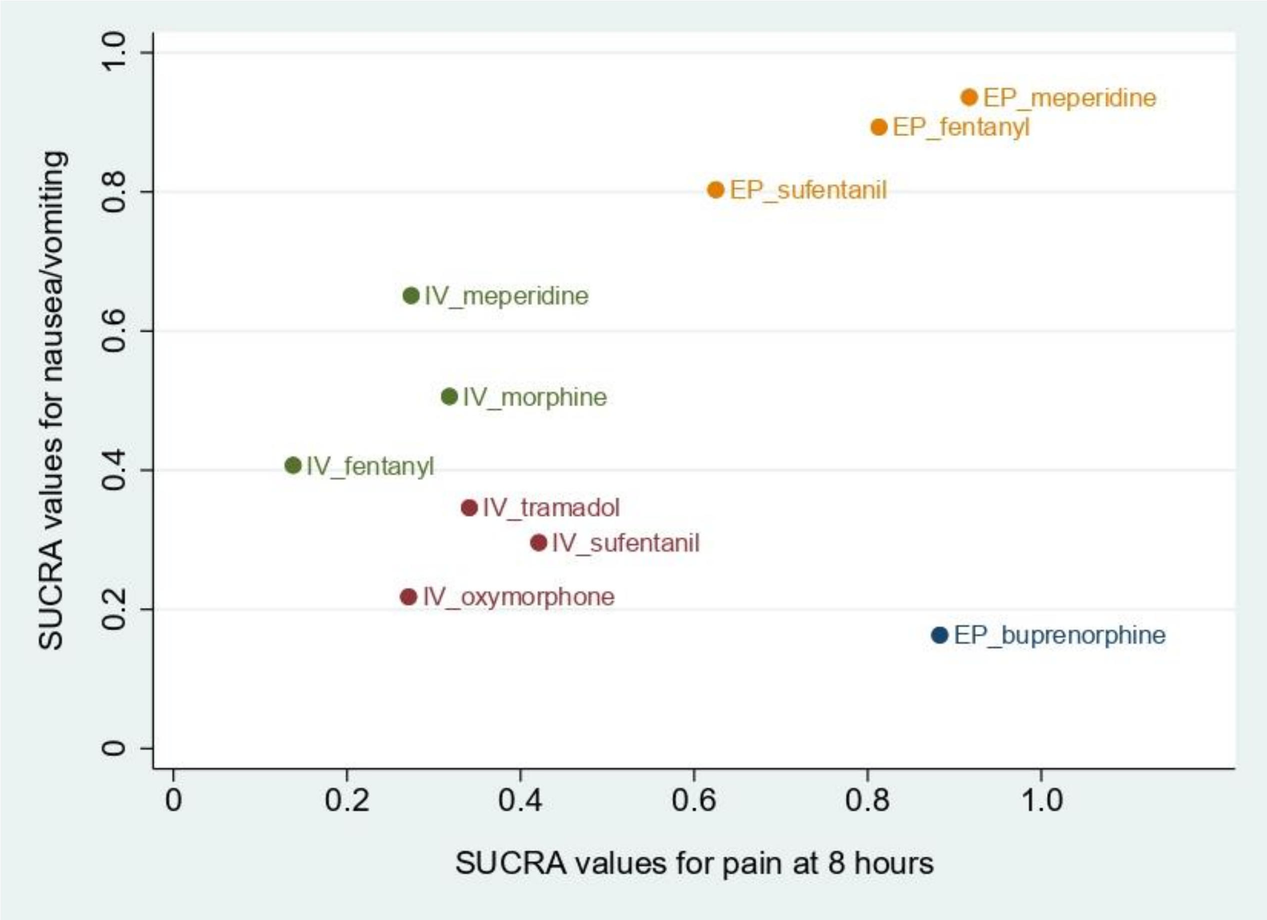
SUCRA values for nausea/vomiting
SUCRA values for pain at 8 hours
0
0.2
0.4
0.6
0.8
1.0
EP_meperidine
EP_fentanyl
EP_sufentanil
IV_meperidine
IV_morphine
IV_fentanyl
IV_tramadol
IV_sufentanil
IV_oxymorphone
EP_buprenorphine

E.

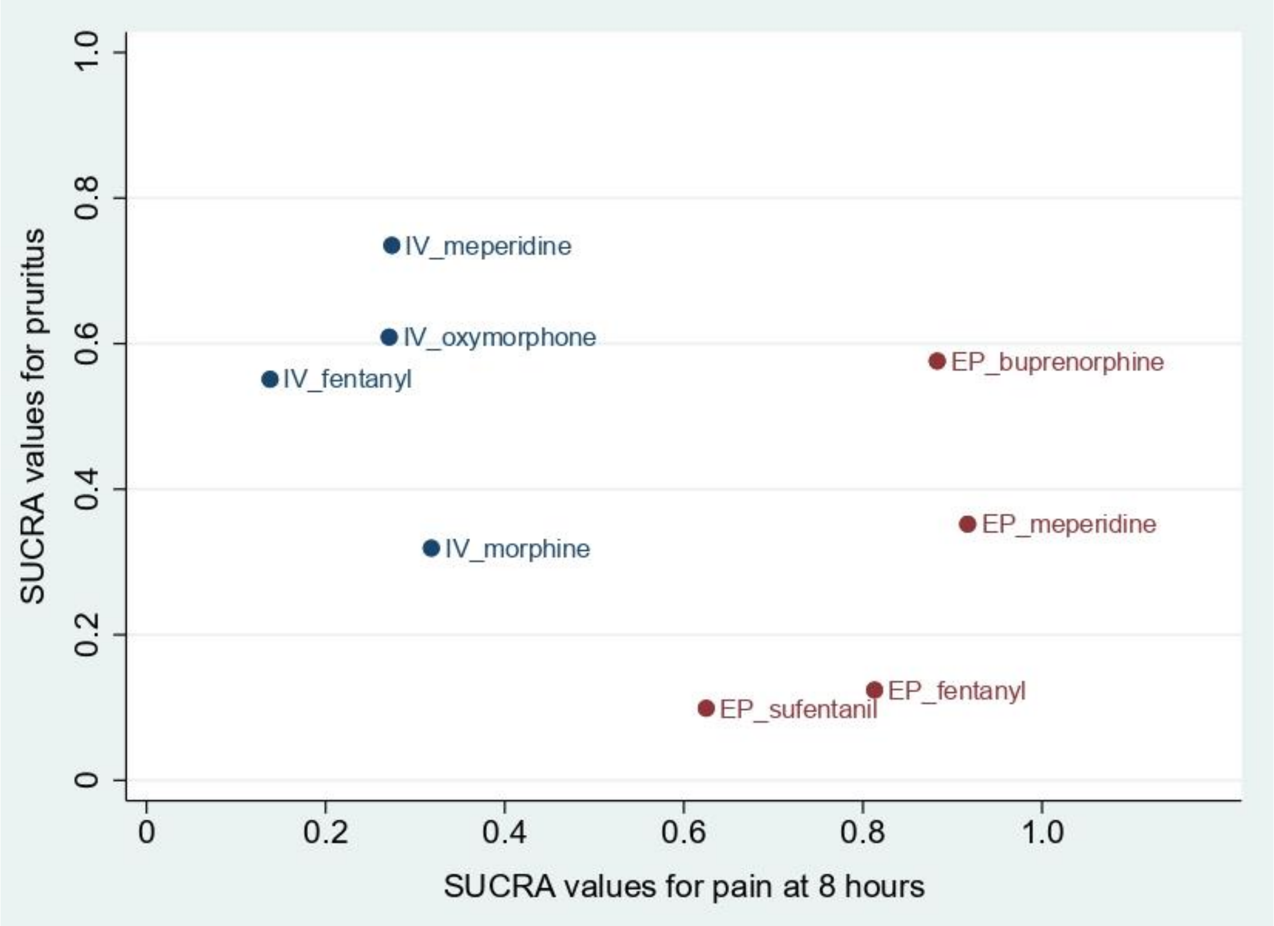
SUCRA values for pruritus
SUCRA values for pain at 8 hours
0
0.2
0.4
0.6
0.8
1.0
IV_meperidine
IV_oxymorphone
IV_fentanyl
EP_buprenorphine
EP_meperidine
IV_morphine
EP_sufentanil
EP_fentanyl

F.

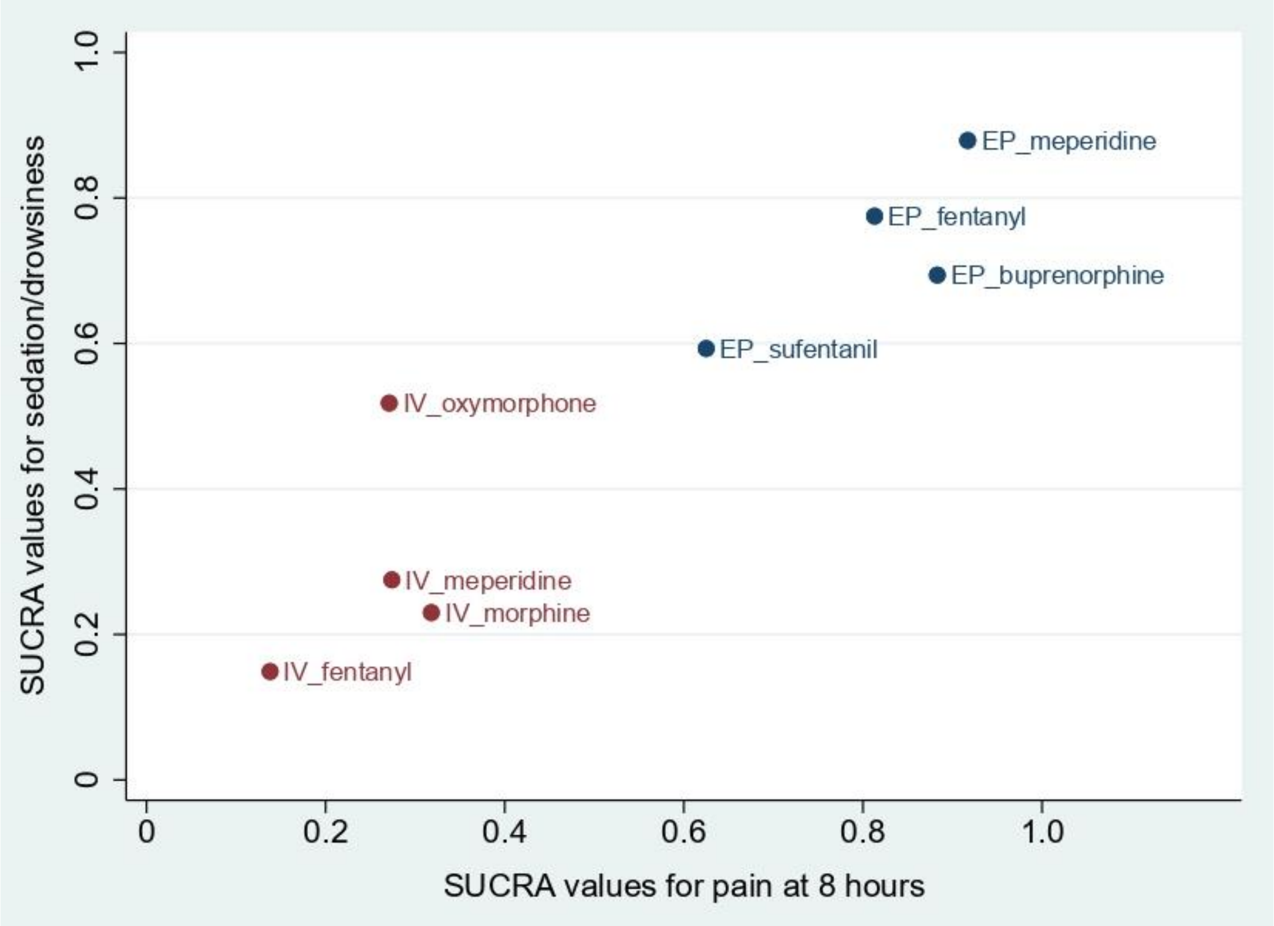
SUCRA values for sedation/drowsiness
SUCRA values for pain at 8 hours
0
0.2
0.4
0.6
0.8
1.0
EP_meperidine
EP_fentanyl
EP_buprenorphine
EP_sufentanil
IV_oxymorphone
IV_meperidine
IV_morphine
IV_fentanyl

G.

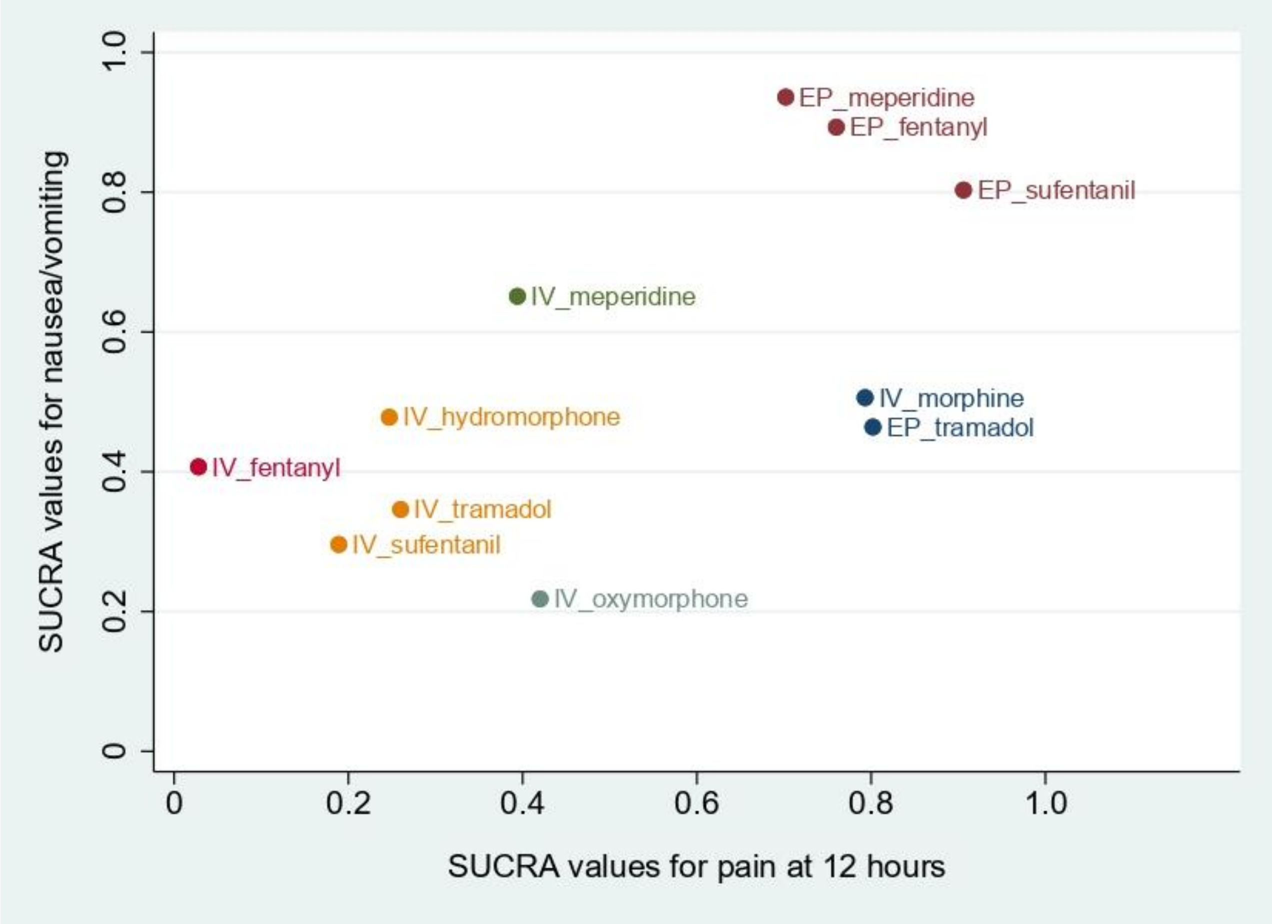
SUCRA values for nausea/vomiting
0
0.2
0.4
0.6
0.8
1.0
SUCRA values for pain at 12 hours
EP_meperidine
EP_fentanyl
EP_sufentanil
IV_meperidine
IV_morphine
EP_tramadol
IV_hydromorphone
IV_fentanyl
IV_tramadol
IV_sufentanil
IV_oxymorphone

H.

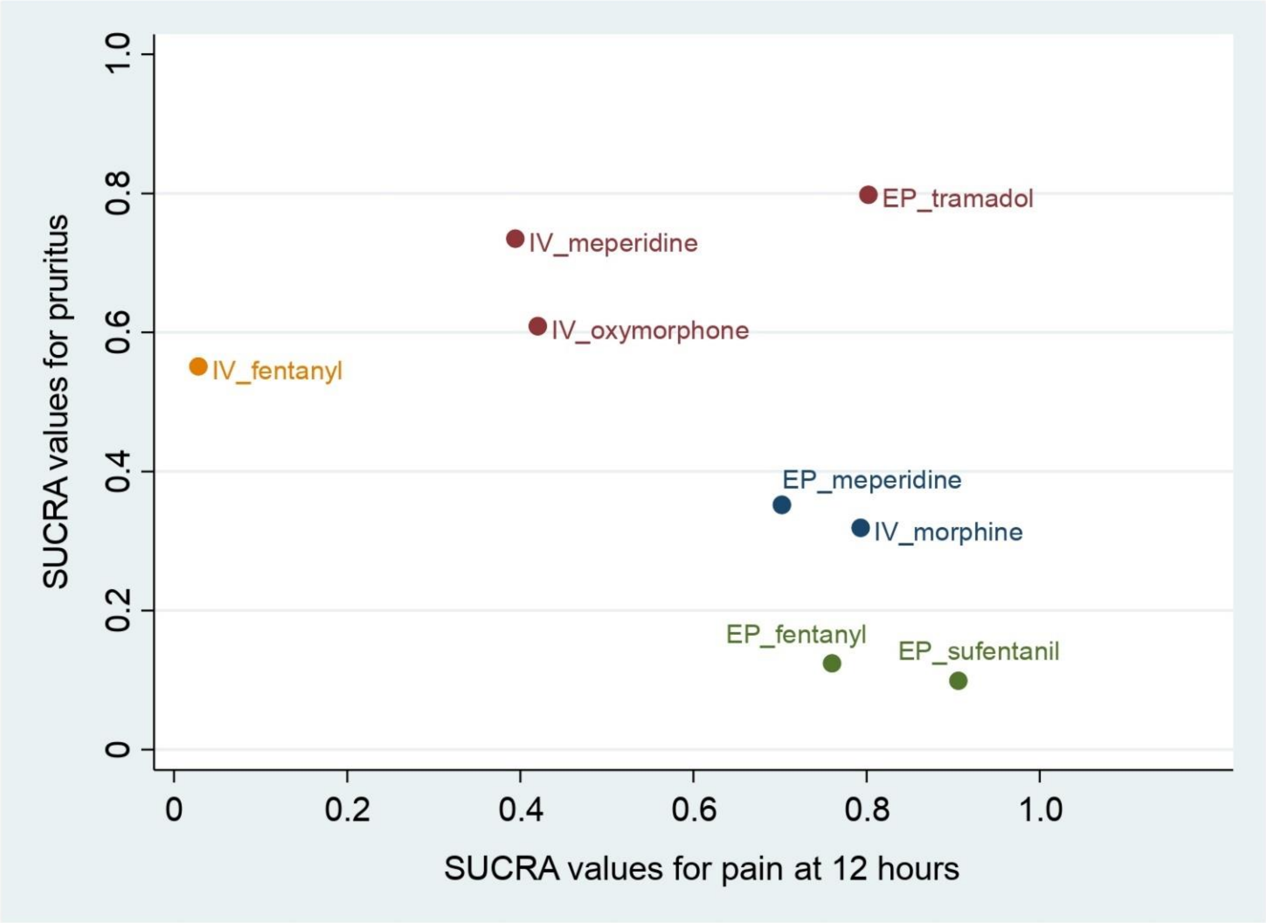
SUCRA values for pruritus
0
0.2
0.4
0.6
0.8
1.0
SUCRA values for pain at 12 hours
EP_tramadol
IV_meperidine
IV_oxymorphone
IV_fentanyl
EP_meperidine
IV_morphine
EP_fentanyl
EP_sufentanil

I.

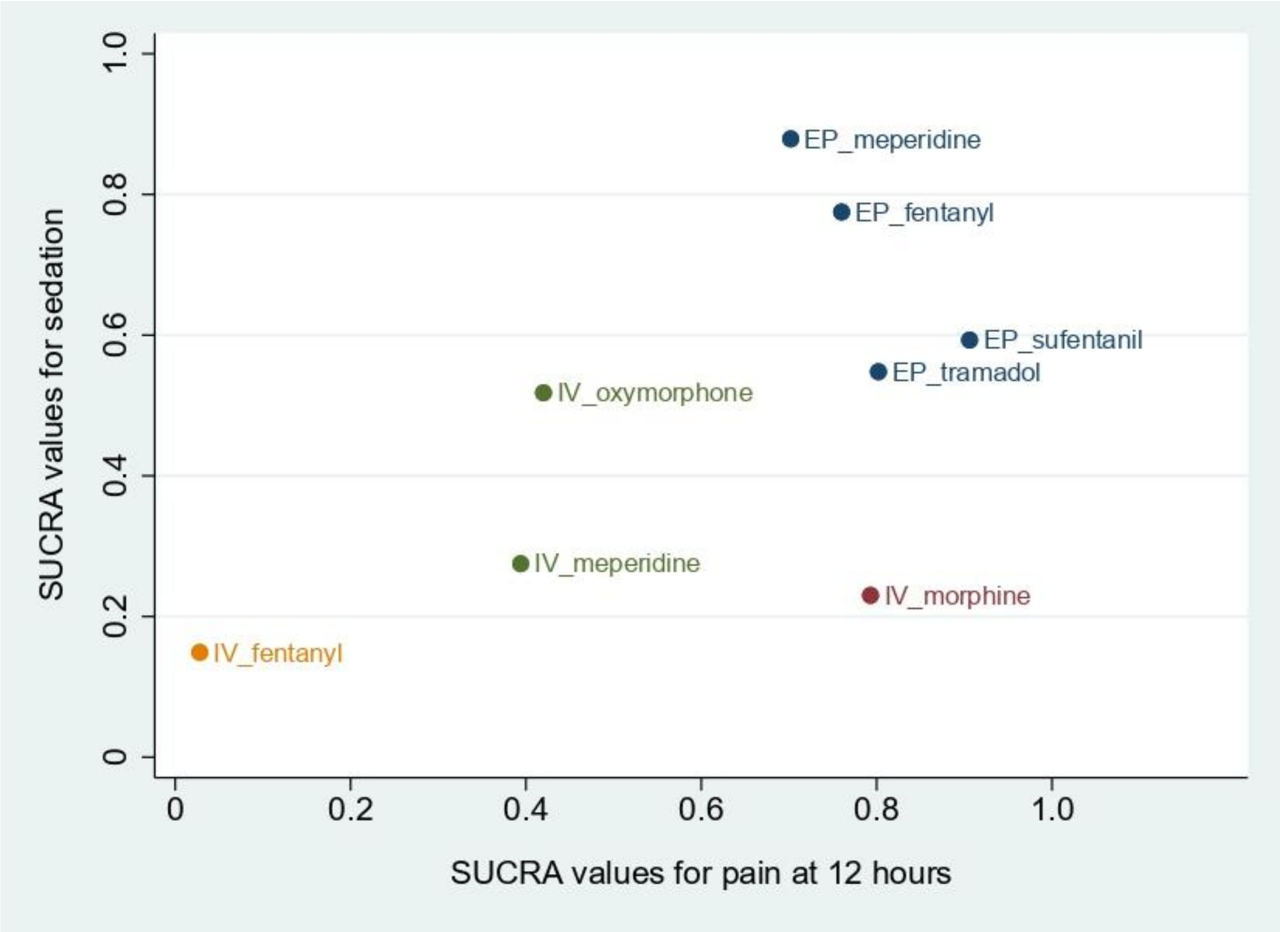
EP_meperidine
EP_fentanyl
EP_sufentanil
EP_tramadol
IV_oxymorphone
IV_meperidine
IV_morphine
IV_fentanyl
SUCRA values for sedation
0
0.2
0.4
0.6
0.8
1.0
SUCRA values for pain at 12 hours

J.

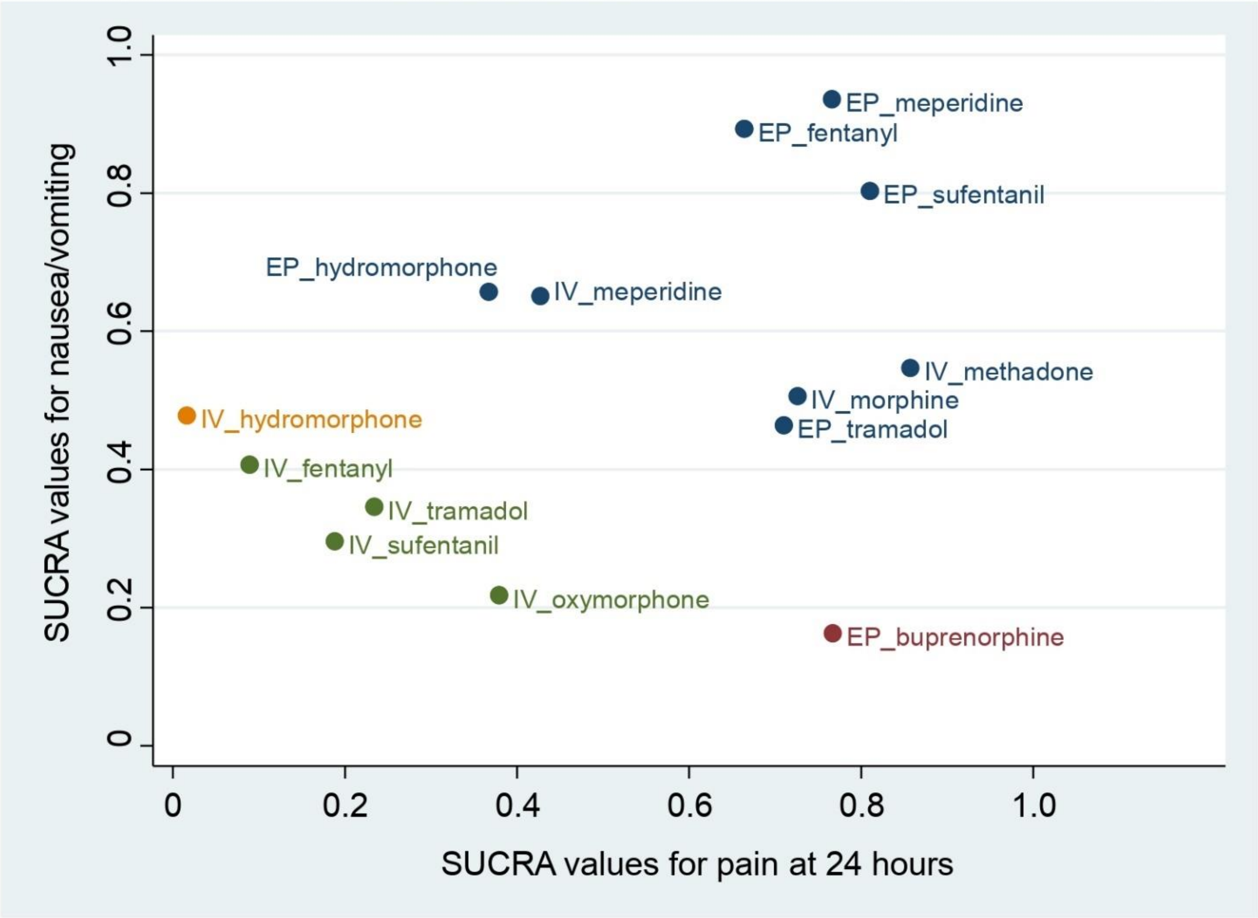
EP_meperidine
EP_fentanyl
EP_sufentanil
EP_hydromorphone
IV_meperidine
IV_methadone
IV_morphine
EP_tramadol
IV_hydromorphone
IV_fentanyl
IV_tramadol
IV_sufentanil
IV_oxymorphone
EP_buprenorphine
SUCRA values for nausea/vomiting
0
0.2
0.4
0.6
0.8
1.0
SUCRA values for pain at 24 hours

K.

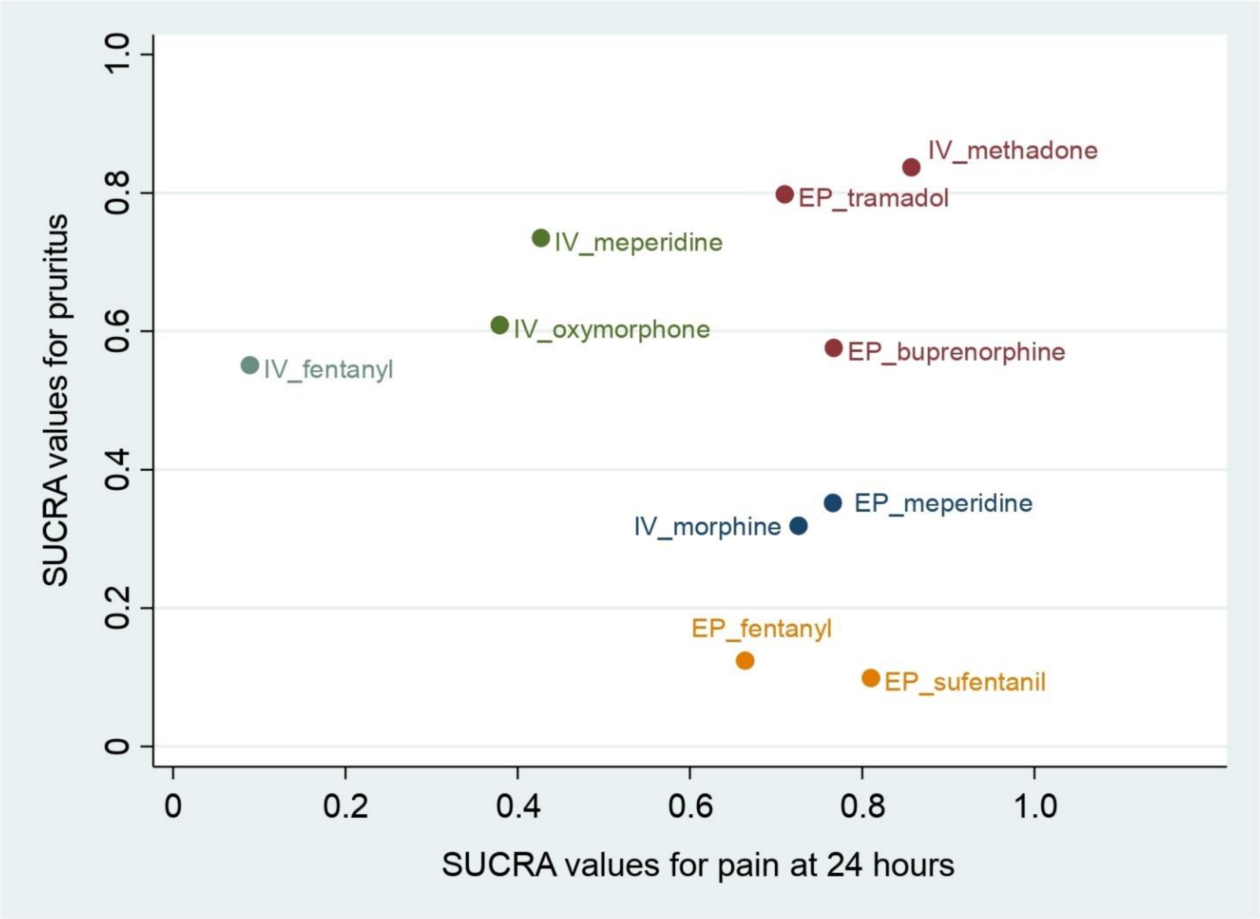
IV_methadone
EP_tramadol
IV_meperidine
IV_oxymorphone
EP_buprenorphine
IV_fentanyl
EP_meperidine
IV_morphine
EP_fentanyl
EP_sufentanil
SUCRA values for pruritus
SUCRA values for pain at 24 hours
0
0.2
0.4
0.6
0.8
1.0

L.

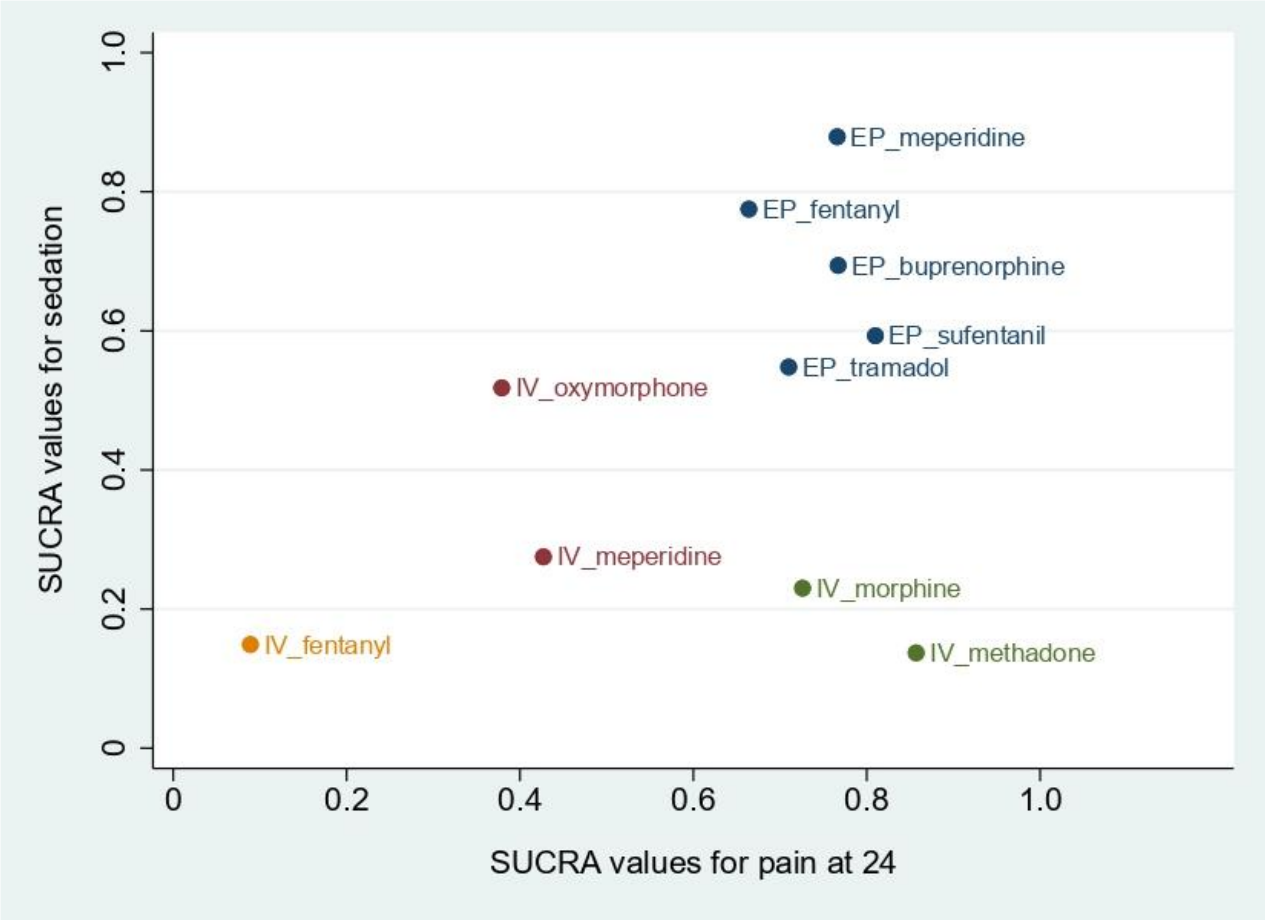
EP_meperidine
EP_fentanyl
EP_buprenorphine
EP_sufentanil
EP_tramadol
IV_oxymorphone
IV_meperidine
IV_morphine
IV_fentanyl
IV_methadone
SUCRA values for sedation
SUCRA values for pain at 24
0
0.2
0.4
0.6
0.8
1.0

Supplementary Figure S16. Clustered ranking plots

Clustered ranking plots are based on cluster analysis of SUCRA values for two different outcomes: analgesic efficacy and adverse effects. Each colour represents a group of treatments that belong to the same cluster. Treatments lying at the upper right corner are more effective and safer (i.e., with lower odds of adverse effects) than the other treatments. (A) Pain intensity at 4 hours after surgery and nausea/vomiting; (B) Pain intensity at 4 hours after surgery and pruritus; (C) Pain intensity at 4 hours after surgery and sedation/drowsiness; (D) Pain intensity at 8 hours after surgery and nausea/vomiting; (E) Pain intensity at 8 hours after surgery and pruritus; (F) Pain intensity at 8 hours after surgery and sedation/drowsiness; (G) Pain intensity at 12 hours after surgery and nausea/vomiting; (H) Pain intensity at 12 hours after surgery and pruritus; (I) Pain intensity at 12 hours after surgery and sedation/drowsiness; (J) Pain intensity at 24 hours after surgery and nausea/vomiting; (K) Pain intensity at 24 hours after surgery and pruritus; (L) Pain intensity at 24 hours after surgery and sedation/drowsiness. The prefix "IV_" denotes IVPCA, and the prefix "EP_" denotes PCEA. SUCRA, the surface under the cumulative ranking curve.

Supplementary Table S10. Assessment of network inconsistency

A. Design-by-treatment interaction model

Outcomes	Chi-square	*p*-value for test of global inconsistency
Pain at 4 hours	1.37	0.8492
Pain at 8 hours	2.13	0.7120
Pain at 12 hours	3.74	0.7117
Pain at 24 hours	0.36	0.8351
Nausea/vomiting	1.17	0.8826
Pruritus	6.01	0.3050
Sedation/drowsiness	4.22	0.3771

A p-value < 0.05 indicates that the inconsistency is statistically significant.

B. Loop inconsistency model

Outcomes	Chi-square	*p*-value for test of global inconsistency
Pain at 4 hours	0.31	0.8564
Pain at 8 hours	1.34	0.5121
Pain at 12 hours	1.60	0.8096
Pain at 24 hours	0.15	0.9269
Nausea/vomiting	0.73	0.6954
Pruritus	5.90	0.1164
Sedation/drowsiness	0.76	0.6844

A p-value < 0.05 indicates that the inconsistency is statistically significant.

C. Side-splitting model on pain at 4 hours

Side	Direct		Indirect		Difference		*p*-value
	Coefficient	SE	Coefficient	SE	Coefficient	SE	
A C	0.762	0.849	0.279	0.594	0.483	1.070	0.652
A D	0.401	0.325	-0.648	1.848	1.049	1.885	0.578
A H	-0.780	0.215	-0.256	0.918	-0.524	0.943	0.578
B C	0.511	0.799	0.187	0.731	0.324	1.068	0.761
B H	-0.822	0.412	-1.871	1.859	1.049	1.885	0.578
B I	-1.346	0.743	-1.883	0.921	0.537	1.200	0.654
C D	-0.361	0.773	0.159	0.707	-0.520	1.060	0.624
C H	-0.704	0.715	-1.547	0.605	0.842	0.935	0.368
C I	-1.827	0.436	-2.876	1.816	1.049	1.885	0.578
H I	-1.093	0.660	-0.090	0.823	-1.003	1.071	0.349

A *p*-value < 0.05 indicates that the difference coefficient is statistically significant; Side A C means C vs. A; A: IVPCA morphine; B: IVPCA fentanyl; C: IVPCA meperidine; D: IVPCA oxymorphone; E: IVPCA sufentanil; F: IVPCA tramadol; G: PCEA buprenorphine; H: PCEA fentanyl; I: PCEA meperidine; SE: standard error

D. Side-splitting model on pain at 8 hours

Side	Direct		Indirect		Difference		*p*-value
	Coefficient	SE	Coefficient	SE	Coefficient	SE	
A C	0.731	0.897	-0.177	0.532	0.908	1.060	0.392
A D	0.188	0.359	-1.708	1.807	1.896	1.865	0.309
A H	-1.065	0.358	-0.117	0.861	-0.948	0.933	0.309
B C	-0.385	0.641	-0.127	0.683	-0.259	0.888	0.771
B H	-1.210	0.400	-3.106	1.862	1.895	1.865	0.309
B I	-1.262	0.694	-2.109	0.732	0.847	0.976	0.385
C D	-0.443	0.759	0.412	0.684	-0.855	1.017	0.401
C H	-0.547	0.486	-1.701	0.608	1.155	0.764	0.131
C I	-1.359	0.297	-3.254	1.852	1.896	1.865	0.309
H I	-0.625	0.564	0.012	0.804	-0.637	0.989	0.520

A *p*-value < 0.05 indicates that the difference coefficient is statistically significant; Side A C means C vs. A; A: IVPCA morphine; B: IVPCA fentanyl; C: IVPCA meperidine; D: IVPCA oxymorphone; E: IVPCA sufentanil; F: IVPCA tramadol; G: PCEA buprenorphine; H: PCEA fentanyl; I: PCEA meperidine; SE: standard error

E. Side-splitting model on pain at 12 hours

Side	Direct		Indirect		Difference		*p*-value
	Coefficient	SE	Coefficient	SE	Coefficient	SE	
A C	0.928	0.529	1.373	0.542	-0.446	0.788	0.572
A D	1.028	0.247	1.493	1.510	-0.465	1.535	0.762
A G	0.061	0.494	0.101	0.497	-0.040	0.701	0.954
A I	0.040	0.744	-0.326	0.461	0.366	0.875	0.676
B C	-0.205	0.904	-1.499	0.651	1.294	1.107	0.243
B G	-2.085	0.432	-2.844	1.276	0.759	1.268	0.550
B H	-2.057	0.700	-1.896	0.677	-0.160	0.940	0.865
C D	-0.135	0.488	-0.053	0.631	-0.082	0.811	0.920
C G	-0.716	0.674	-1.219	0.447	0.503	0.812	0.536
C H	-1.018	0.345	-0.244	0.879	-0.774	0.945	0.413
G H	0.049	0.393	0.437	0.658	-0.388	0.767	0.613
G I	-0.333	0.243	0.032	0.841	-0.366	0.875	0.676

A *p*-value < 0.05 indicates that the difference coefficient is statistically significant; Side A C means C vs. A; A: IVPCA morphine; B: IVPCA fentanyl; C: IVPCA meperidine; D: IVPCA oxymorphone; E: IVPCA sufentanil; F: IVPCA tramadol; G: PCEA fentanyl; H: PCEA meperidine; I: PCEA sufentanil; J: PCEA tramadol; K: IVPCA hydromorphone; SE: standard error

F. Side-splitting model on pain at 24 hours

Side	Direct		Indirect		Difference		*p*-value
	Coefficient	SE	Coefficient	SE	Coefficient	SE	

A C	0.859	0.479	1.522	1.012	-0.663	1.107	0.549
A E	1.186	0.210	2.315	2.902	-1.129	2.900	0.697
A K	-0.007	0.660	-0.572	1.291	0.565	1.450	0.697
C E	0.108	0.739	0.347	0.881	-0.239	1.315	0.856
C J	-1.100	0.539	-0.535	1.346	-0.565	1.450	0.697
I J	0.057	1.050	-0.507	1.000	0.565	1.450	0.697
I K	-0.333	0.249	0.231	1.428	-0.565	1.450	0.697

A *p*-value < 0.05 indicates that the difference coefficient is statistically significant; Comparison A C means C vs. A; A: IVPCA morphine; B: IVPCA fentanyl; C: IVPCA meperidine; D: IVPCA methadone; E: IVPCA oxymorphone; F: IVPCA sufentanil; G: IVPCA tramadol; H: PCEA buprenorphine; I: PCEA fentanyl; J: PCEA meperidine; K: PCEA sufentanil; L: PCEA tramadol; M: PCEA hydromorphone; N: IVPCA hydromorphone; SE: standard error

G. Side-splitting model on nausea/vomiting

Side	Direct		Indirect		Difference		*p*-value
	Coefficient	SE	Coefficient	SE	Coefficient	SE	
A B	0.887	0.938	-0.195	0.864	1.082	1.276	0.396
A C	-0.407	0.521	-0.627	1.449	0.221	1.540	0.886
A F	0.955	0.661	0.649	2.056	0.305	2.240	0.892
A J	-1.565	0.690	-0.841	0.936	-0.724	1.163	0.534
B J	-1.390	0.577	-2.472	1.138	1.082	1.276	0.396
C F	1.480	0.872	1.146	1.098	0.334	1.436	0.816
C K	-1.543	1.592	-1.088	0.985	-0.455	1.872	0.808
J K	-0.288	0.607	-0.743	1.771	0.455	1.872	0.808

A *p*-value < 0.05 indicates that the difference coefficient is statistically significant; Side A B means B vs. A; A: IVPCA morphine; B: IVPCA fentanyl; C: IVPCA meperidine; D: IVPCA meptazinol; E: IVPCA methadone; F: IVPCA oxymorphone; G: IVPCA sufentanil; H: IVPCA tramadol; I: PCEA buprenorphine; J: PCEA fentanyl; K: PCEA meperidine; L: PCEA sufentanil; M: PCEA tramadol; N: PCEA hydromorphone; O: IVPCA hydromorphone; SE: standard error

H. Side-splitting model on pruritus

Side	Direct		Indirect		Difference		*p*-value
	Coefficient	SE	Coefficient	SE	Coefficient	SE	
A B	-1.099	1.737	-0.484	0.804	-0.615	1.914	0.748
A C	-1.295	0.717	-1.212	1.243	-0.083	1.420	0.953
A E	-0.951	0.897	-0.093	2.788	-0.858	2.868	0.765
A G	0.041	0.516	1.417	0.709	-1.377	0.877	0.116
A I	2.208	0.865	-0.301	0.582	2.509	1.043	0.016
B G	1.127	0.596	1.741	1.822	-0.615	1.914	0.748
C E	0.685	1.338	-0.032	1.619	0.717	2.184	0.743
C H	1.172	0.797	1.363	1.267	-0.191	1.496	0.898
G H	-0.588	0.857	-0.779	1.226	0.191	1.496	0.898

G I	-0.394	0.400	2.115	0.963	-2.509	1.043	0.016

A p-value < 0.05 indicates that the difference coefficient is statistically significant; Side A B means B vs. A; A: IVPCA morphine; B: IVPCA fentanyl; C: IVPCA meperidine; D: IVPCA methadone; E: IVPCA oxymorphone; F: PCEA buprenorphine; G: PCEA fentanyl; H: PCEA meperidine; I: PCEA sufentanil; J: PCEA tramadol; SE: standard error

I. Side-splitting model on sedation/drowsiness

Side	Direct		Indirect		Difference		*p*-value
	Coefficient	SE	Coefficient	SE	Coefficient	SE	
A C	-0.096	0.382	-0.166	0.771	0.070	0.824	0.932
A F	-0.816	0.408	1.221	1.483	-2.037	1.426	0.153
A H	-1.569	0.500	-1.441	0.518	-0.127	0.720	0.860
A J	-0.748	0.597	-1.326	0.502	0.577	0.780	0.459
C F	0.268	0.734	-1.879	0.804	2.147	1.089	0.049
C I	-1.996	0.543	-1.368	0.773	-0.628	0.945	0.506
H I	-0.187	0.537	-0.814	0.777	0.628	0.945	0.506
H J	0.359	0.256	0.936	0.737	-0.577	0.780	0.459

A p-value < 0.05 indicates that the difference coefficient is statistically significant; Side A C means C vs. A; A: IVPCA morphine; B: IVPCA fentanyl; C: IVPCA meperidine; D: IVPCA meptazinol; E: IVPCA methadone; F: IVPCA oxymorphone; G: PCEA buprenorphine; H: PCEA fentanyl; I: PCEA meperidine; J: PCEA sufentanil; K: PCEA tramadol; SE: standard error

A.

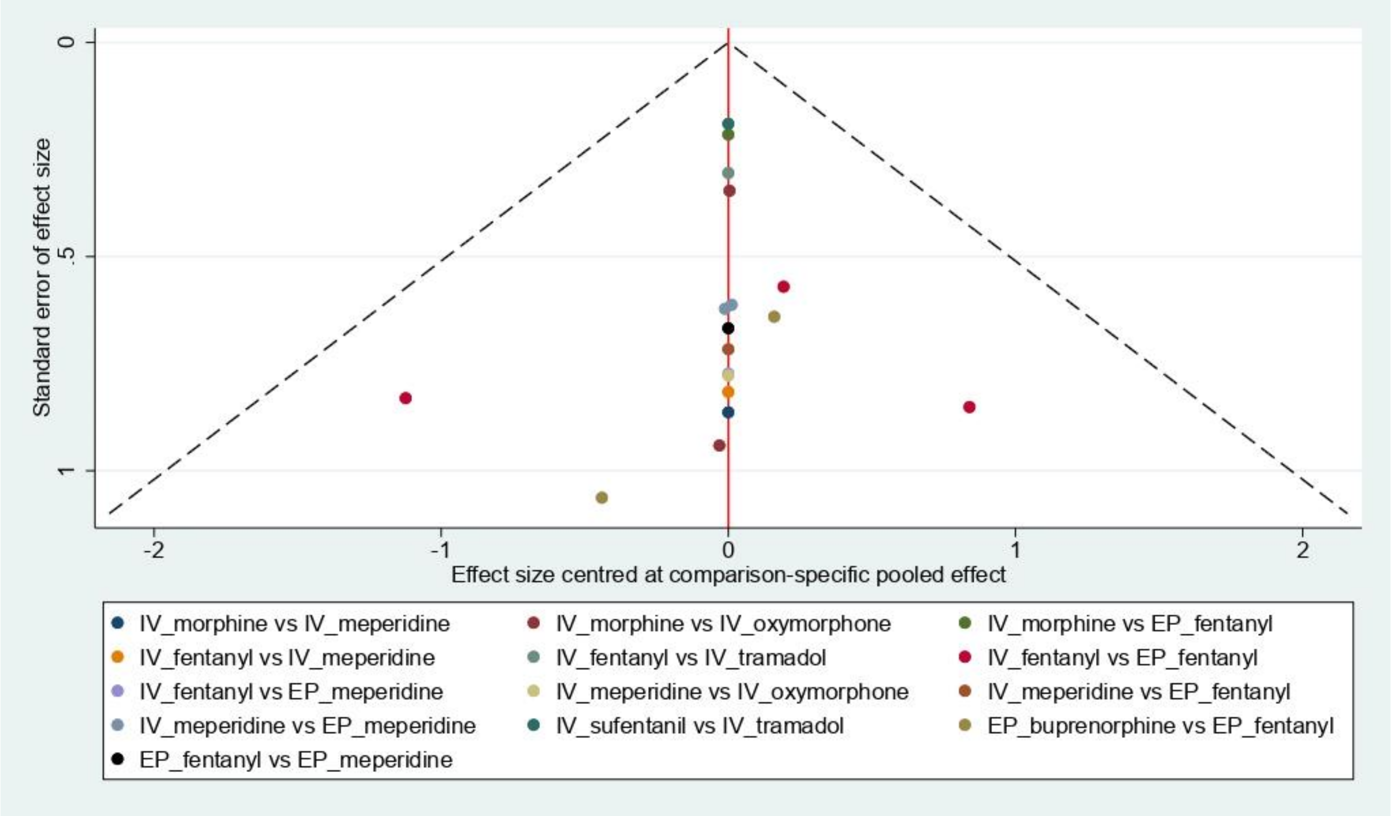
Standard error of effect size
0
.5
1
-2
-1
0
1
2
Effect size centred at comparison-specific pooled effect
IV_morphine vs IV_meperidine
IV_morphine vs IV_oxymorphone
IV_morphine vs EP_fentanyl
IV_fentanyl vs IV_meperidine
IV_fentanyl vs IV_tramadol
IV_fentanyl vs EP_fentanyl
IV_fentanyl vs EP_meperidine
IV_meperidine vs IV_oxymorphone
IV_meperidine vs EP_fentanyl
IV_meperidine vs EP_meperidine
IV_sufentanil vs IV_tramadol
EP_buprenorphine vs EP_fentanyl
EP_fentanyl vs EP_meperidine

B.

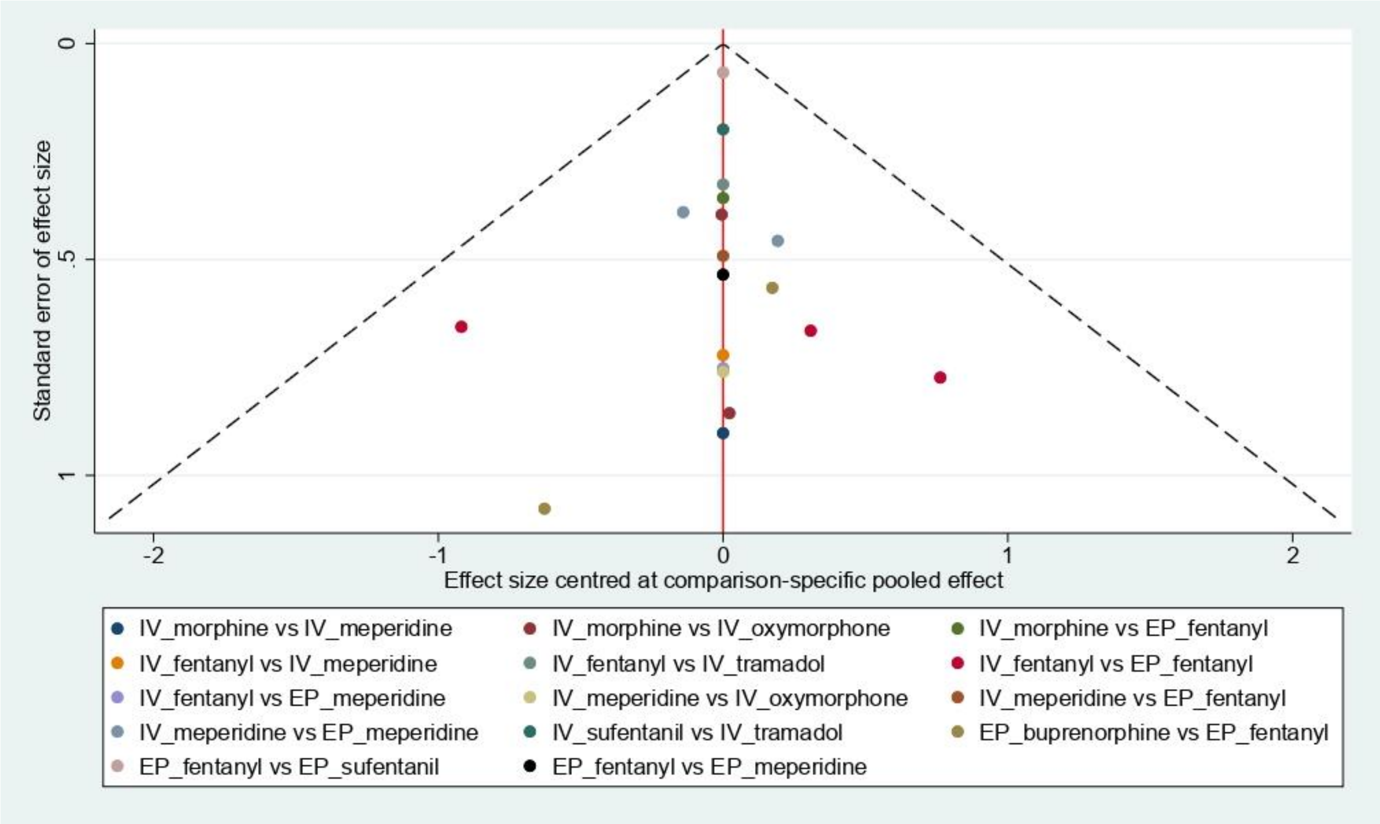
Standard error of effect size
0
.5
1
-2
-1
0
1
2
Effect size centred at comparison-specific pooled effect
IV_morphine vs IV_meperidine
IV_morphine vs IV_oxymorphone
IV_morphine vs EP_fentanyl
IV_fentanyl vs IV_meperidine
IV_fentanyl vs IV_tramadol
IV_fentanyl vs EP_fentanyl
IV_fentanyl vs EP_meperidine
IV_meperidine vs IV_oxymorphone
IV_meperidine vs EP_fentanyl
IV_meperidine vs EP_meperidine
IV_sufentanil vs IV_tramadol
EP_buprenorphine vs EP_fentanyl
EP_fentanyl vs EP_sufentanil
EP_fentanyl vs EP_meperidine

C.

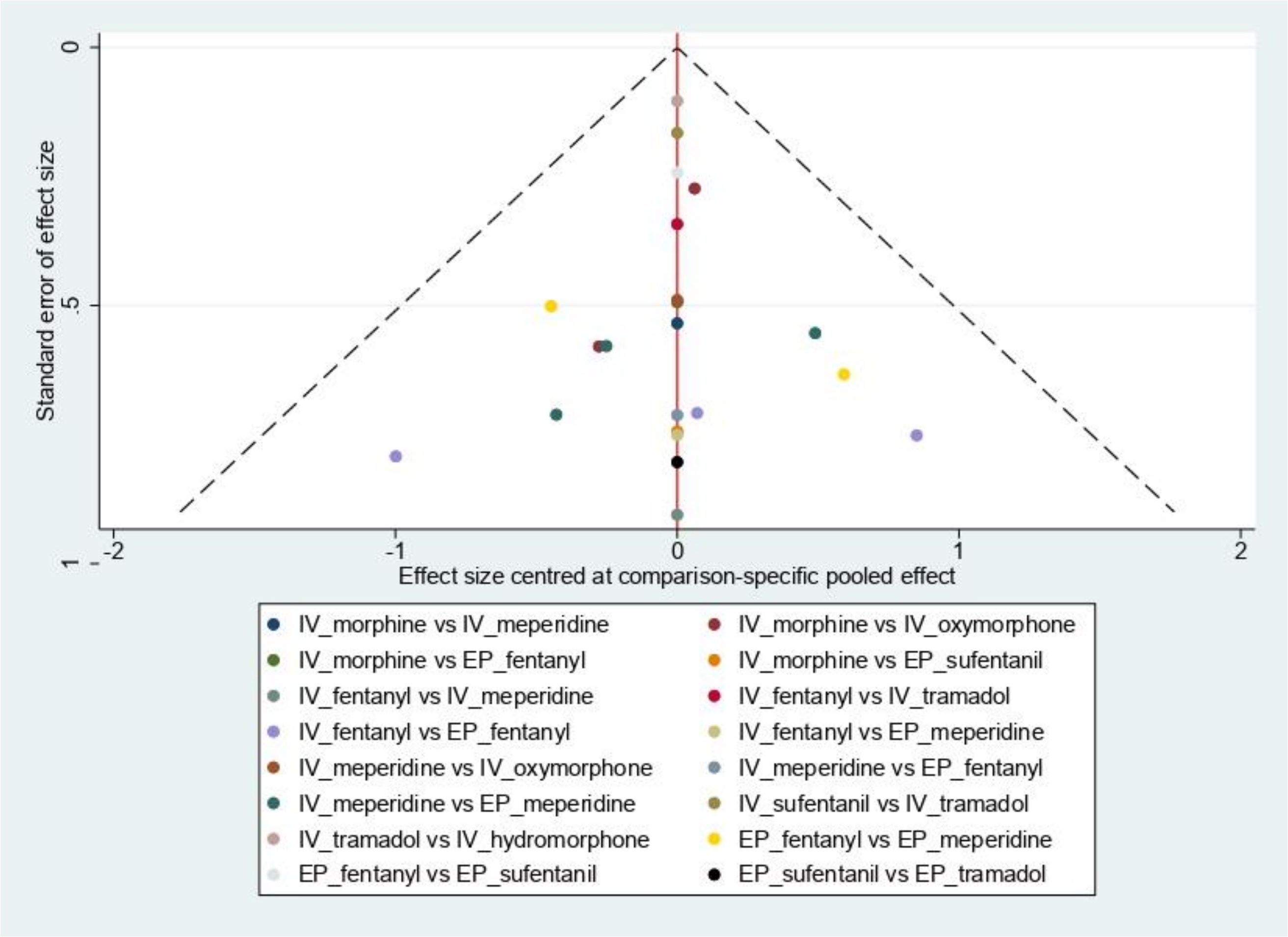
Standard error of effect size
0
.5
1
-2
-1
0
1
2
Effect size centred at comparison-specific pooled effect
IV_morphine vs IV_meperidine
IV_morphine vs IV_oxymorphone
IV_morphine vs EP_fentanyl
IV_morphine vs EP_sufentanil
IV_fentanyl vs IV_meperidine
IV_fentanyl vs IV_tramadol
IV_fentanyl vs EP_fentanyl
IV_fentanyl vs EP_meperidine
IV_meperidine vs IV_oxymorphone
IV_meperidine vs EP_fentanyl
IV_meperidine vs EP_meperidine
IV_sufentanil vs IV_tramadol
IV_tramadol vs IV_hydromorphone
EP_fentanyl vs EP_meperidine
EP_fentanyl vs EP_sufentanil
EP_sufentanil vs EP_tramadol

D.

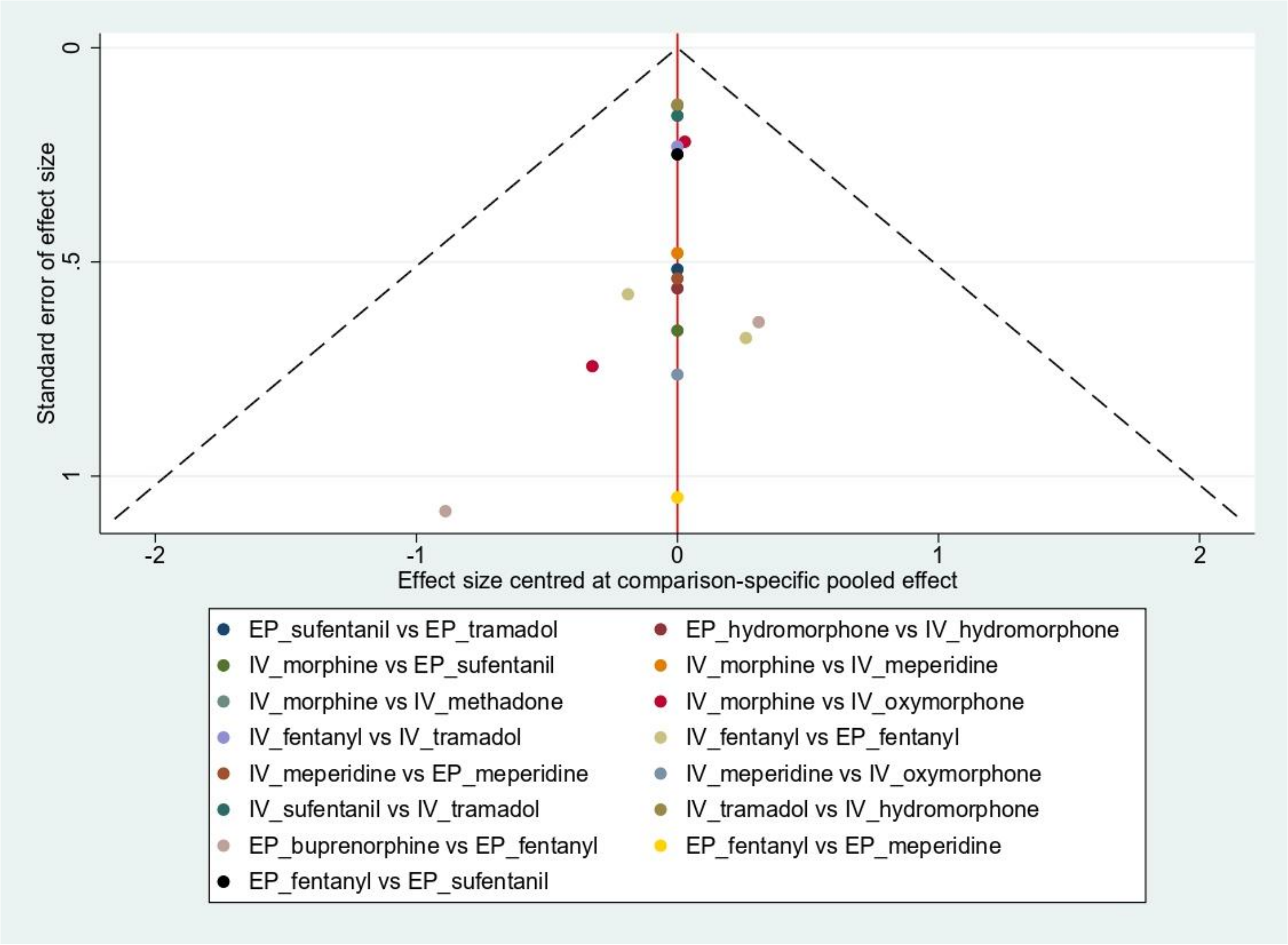
Standard error of effect size
0
.5
1
-2
-1
0
1
2
Effect size centred at comparison-specific pooled effect
EP_sufentanil vs EP_tramadol
EP_hydromorphone vs IV_hydromorphone
IV_morphine vs EP_sufentanil
IV_morphine vs IV_meperidine
IV_morphine vs IV_methadone
IV_morphine vs IV_oxymorphone
IV_fentanyl vs IV_tramadol
IV_fentanyl vs EP_fentanyl
IV_meperidine vs EP_meperidine
IV_meperidine vs IV_oxymorphone
IV_sufentanil vs IV_tramadol
IV_tramadol vs IV_hydromorphone
EP_buprenorphine vs EP_fentanyl
EP_fentanyl vs EP_meperidine
EP_fentanyl vs EP_sufentanil

E.

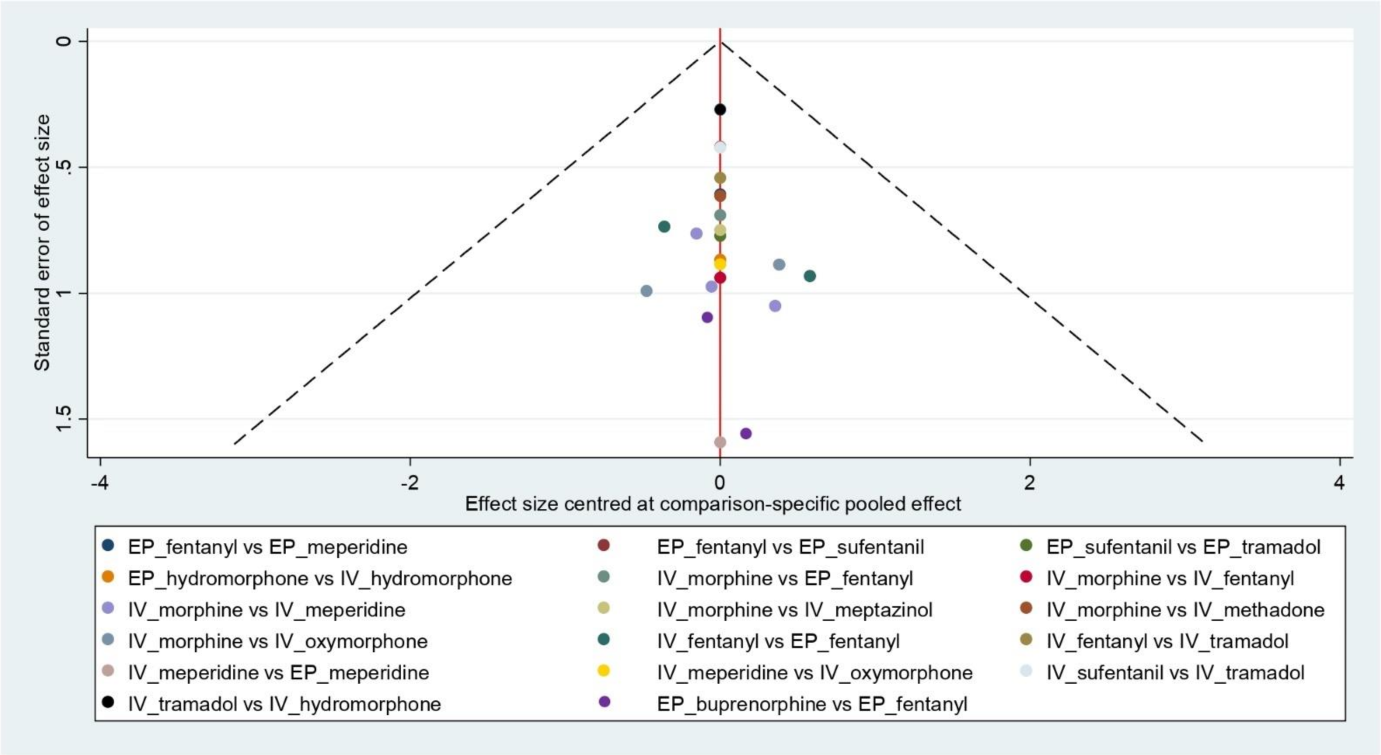
Standard error of effect size
0
.5
1
1.5
-4
-2
0
2
4
Effect size centred at comparison-specific pooled effect
EP_fentanyl vs EP_meperidine
EP_fentanyl vs EP_sufentanil
EP_sufentanil vs EP_tramadol
EP_hydromorphone vs IV_hydromorphone
IV_morphine vs EP_fentanyl
IV_morphine vs IV_fentanyl
IV_morphine vs IV_meperidine
IV_morphine vs IV_meptazinol
IV_morphine vs IV_methadone
IV_morphine vs IV_oxymorphone
IV_fentanyl vs EP_fentanyl
IV_fentanyl vs IV_tramadol
IV_meperidine vs EP_meperidine
IV_meperidine vs IV_oxymorphone
IV_sufentanil vs IV_tramadol
IV_tramadol vs IV_hydromorphone
EP_buprenorphine vs EP_fentanyl

F.

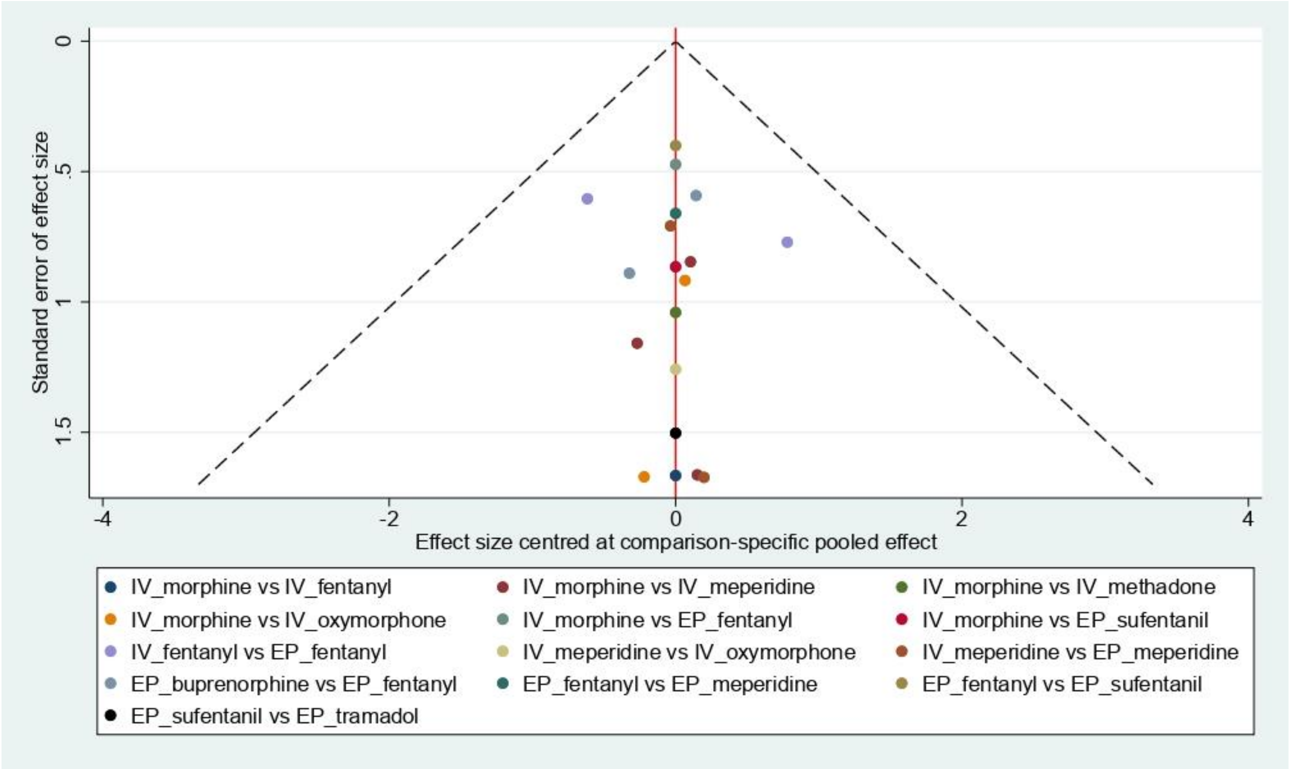
Standard error of effect size
0
.5
1
1.5
-4
-2
0
2
4
Effect size centred at comparison-specific pooled effect
IV_morphine vs IV_fentanyl
IV_morphine vs IV_meperidine
IV_morphine vs IV_methadone
IV_morphine vs IV_oxymorphone
IV_morphine vs EP_fentanyl
IV_morphine vs EP_sufentanil
IV_fentanyl vs EP_fentanyl
IV_meperidine vs IV_oxymorphone
IV_meperidine vs EP_meperidine
EP_buprenorphine vs EP_fentanyl
EP_fentanyl vs EP_meperidine
EP_fentanyl vs EP_sufentanil
EP_sufentanil vs EP_tramadol

G.

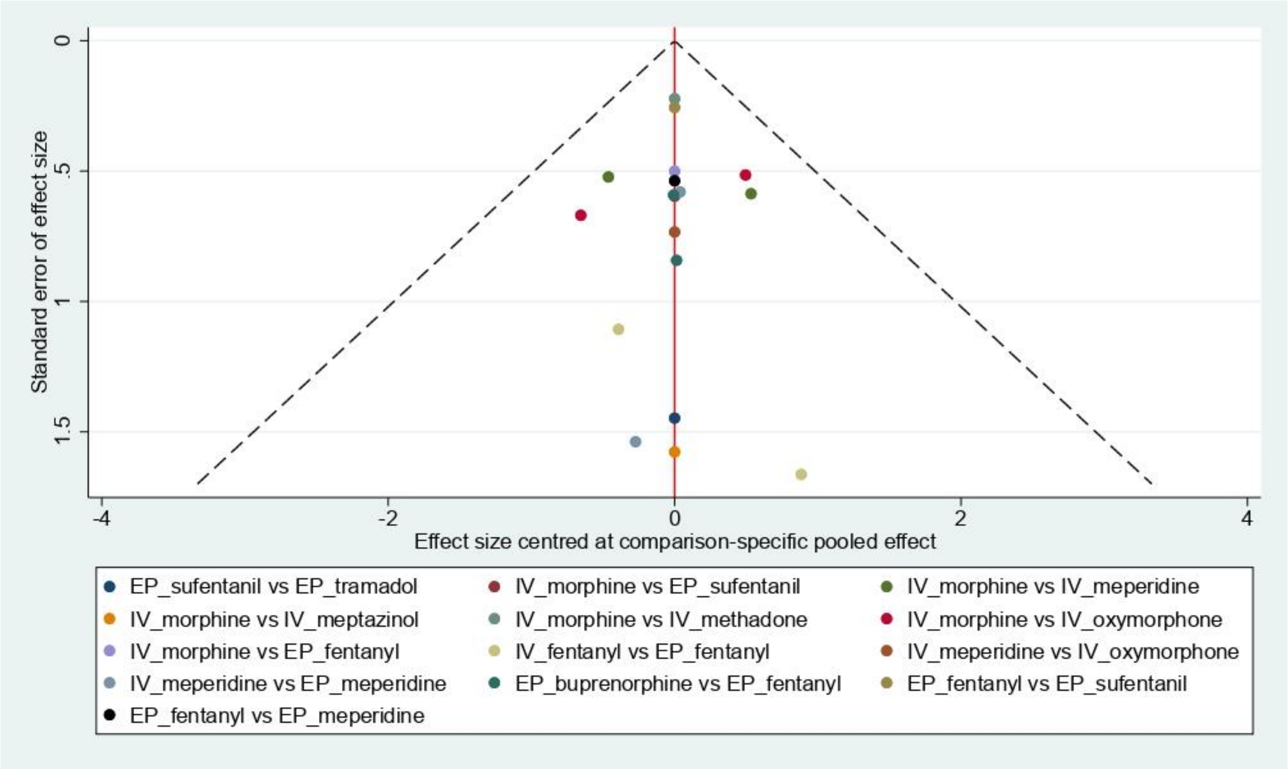

Supplementary Figure S17. Comparison-adjusted funnel plots

(A) pain intensity at 4 hours after surgery, (B) pain intensity at 8 hours after surgery, (C) pain intensity at 12 hours after surgery, (D) pain intensity at 24 hours after surgery, (E) nausea/vomiting, (F) pruritus, and (G) sedation/drowsiness.

A.

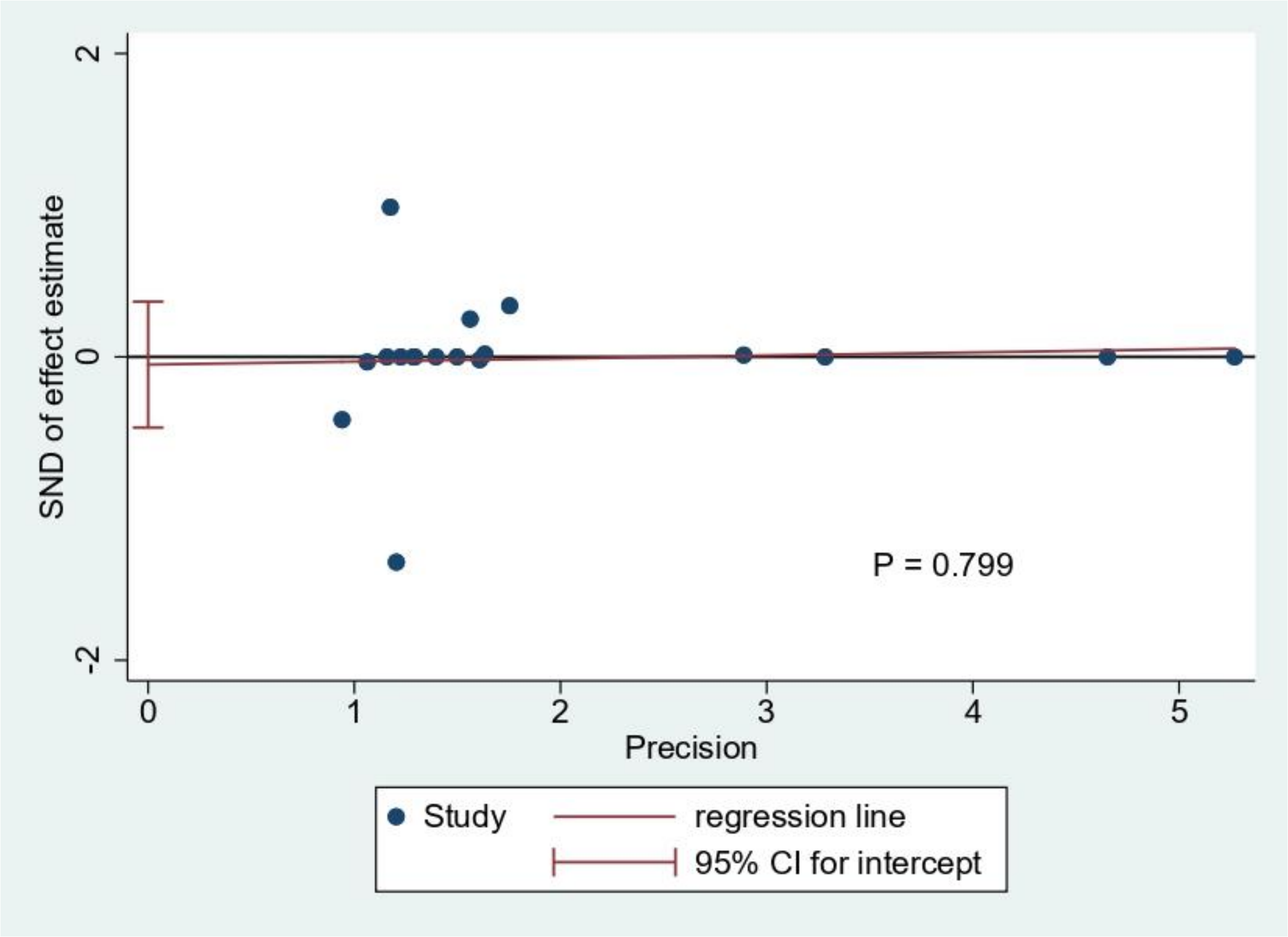
2
0
-2
SND of effect estimate
0
1
2
3
4
5
Precision
P = 0.799
Study
regression line
95% CI for intercept

B.

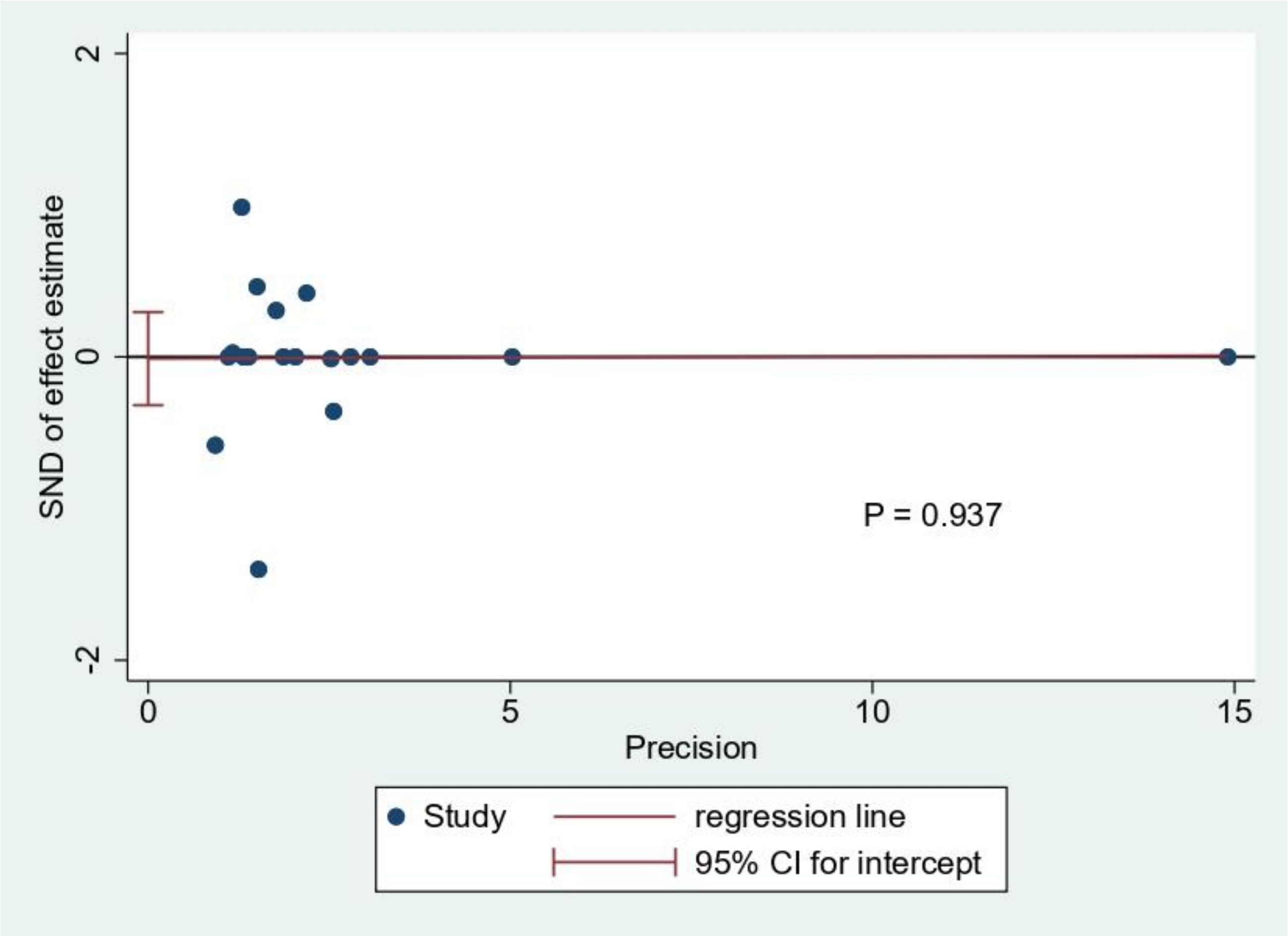
2
0
-2
SND of effect estimate
0
5
10
15
Precision
P = 0.937
Study
regression line
95% CI for intercept

C.

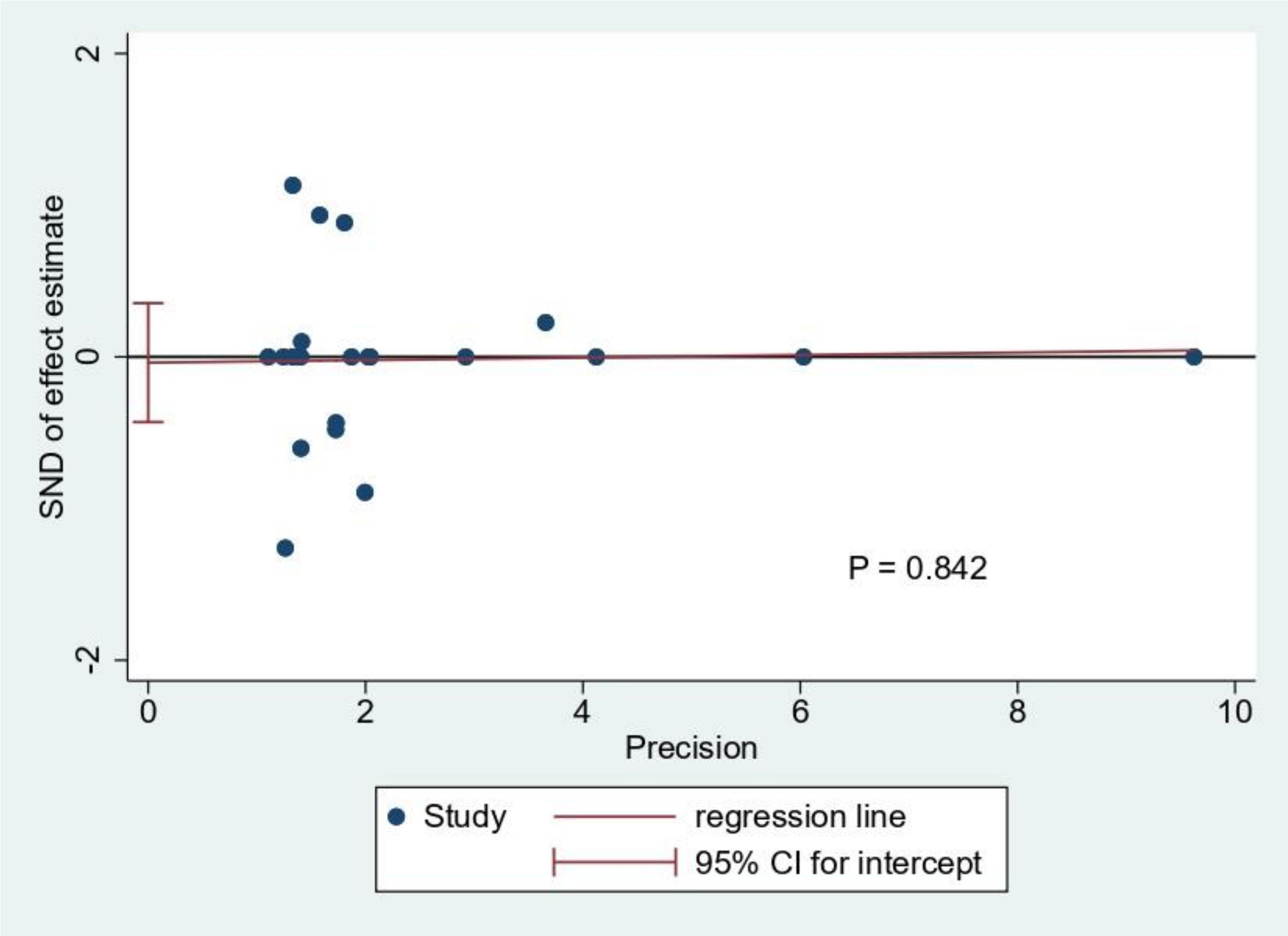
SND of effect estimate
2
0
-2
0
2
4
6
8
10
Precision
P = 0.842
Study
regression line
95% CI for intercept

D.

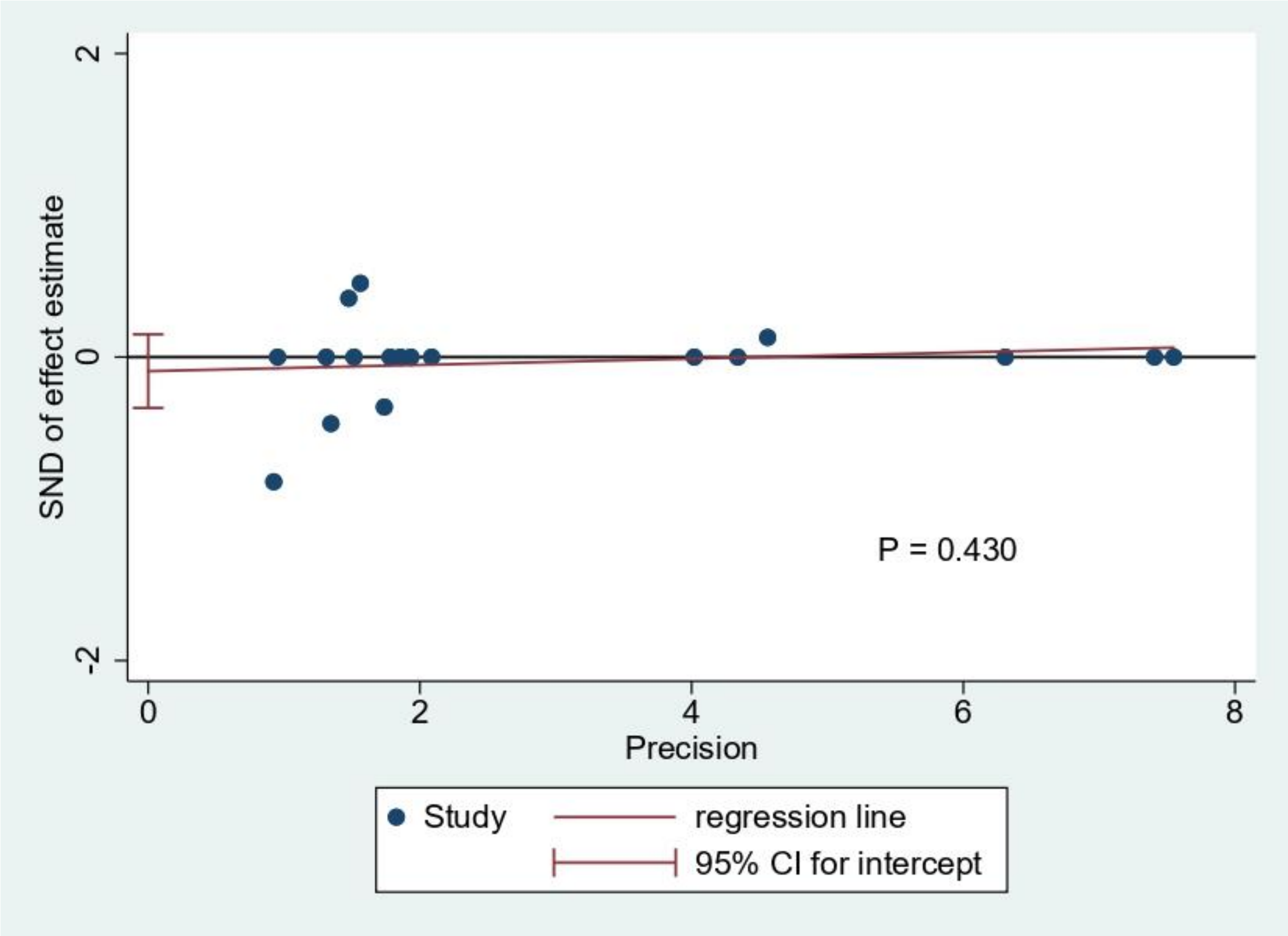
SND of effect estimate
2
0
-2
0
2
4
6
8
Precision
P = 0.430
Study
regression line
95% CI for intercept

E.

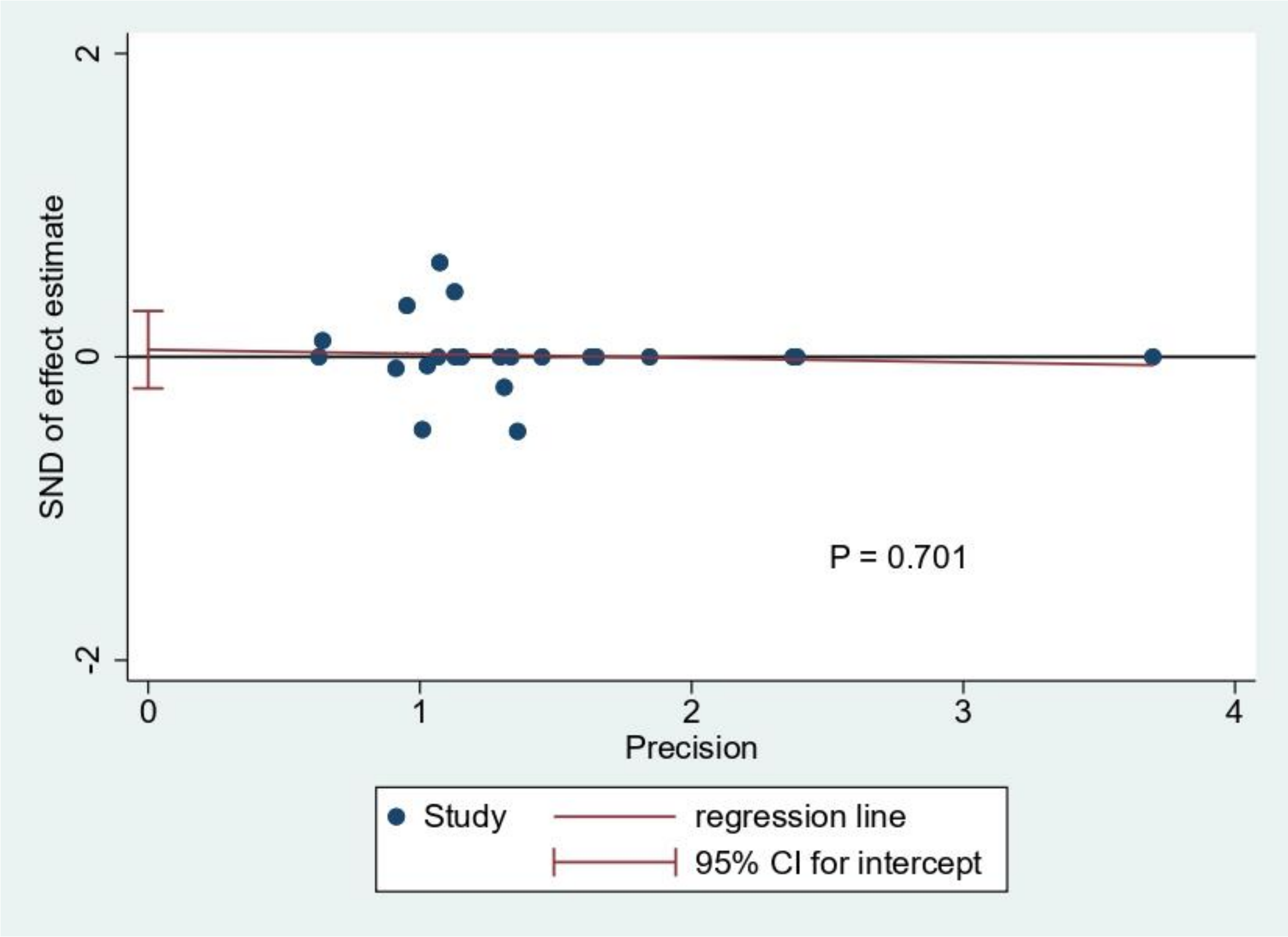
SND of effect estimate
2
0
-2
0
1
2
3
4
Precision
P = 0.701
Study
regression line
95% CI for intercept

F.

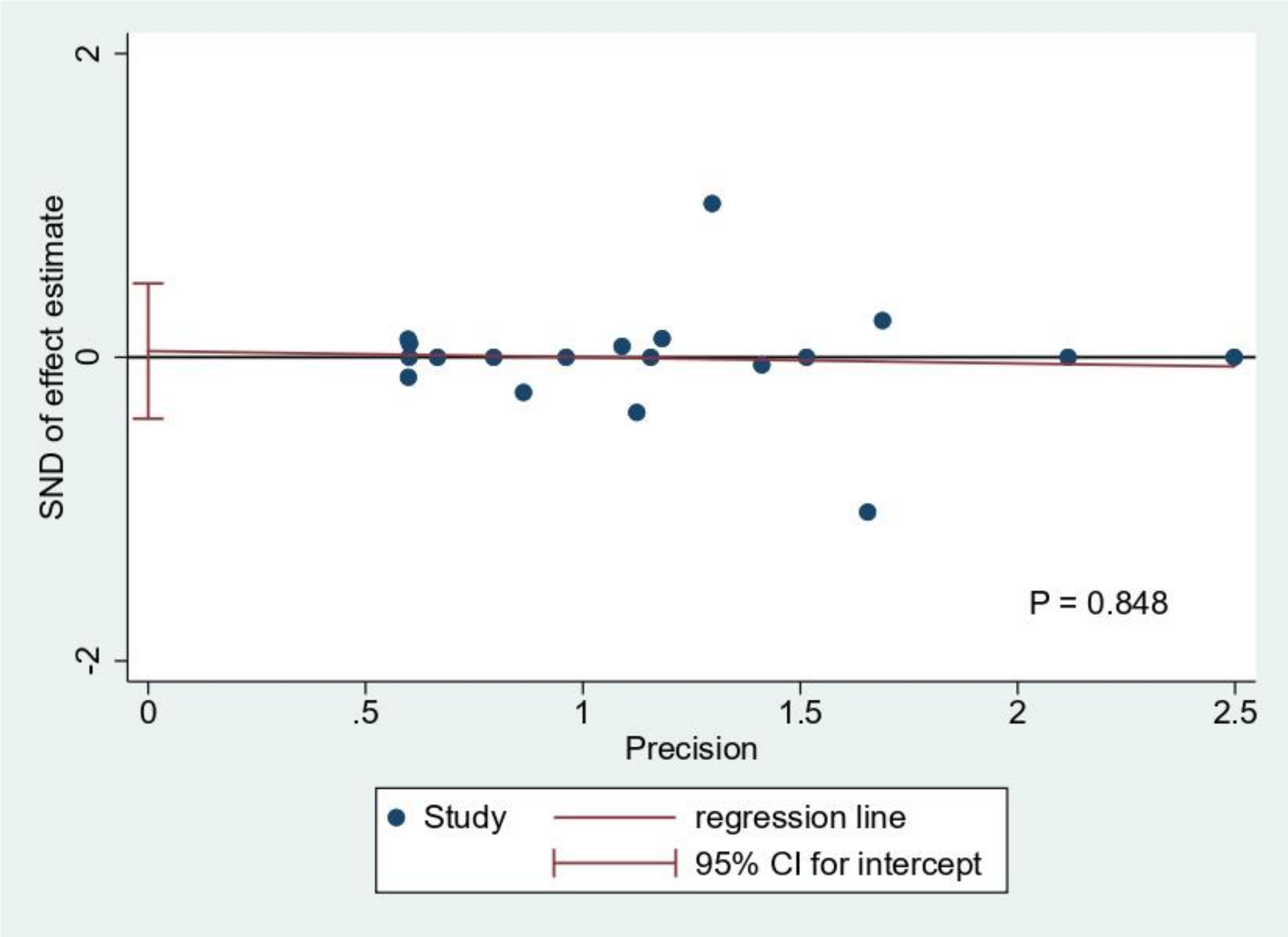
SND of effect estimate
2
0
-2
0
.5
1
1.5
2
2.5
Precision
P = 0.848
Study
regression line
95% CI for intercept

G.

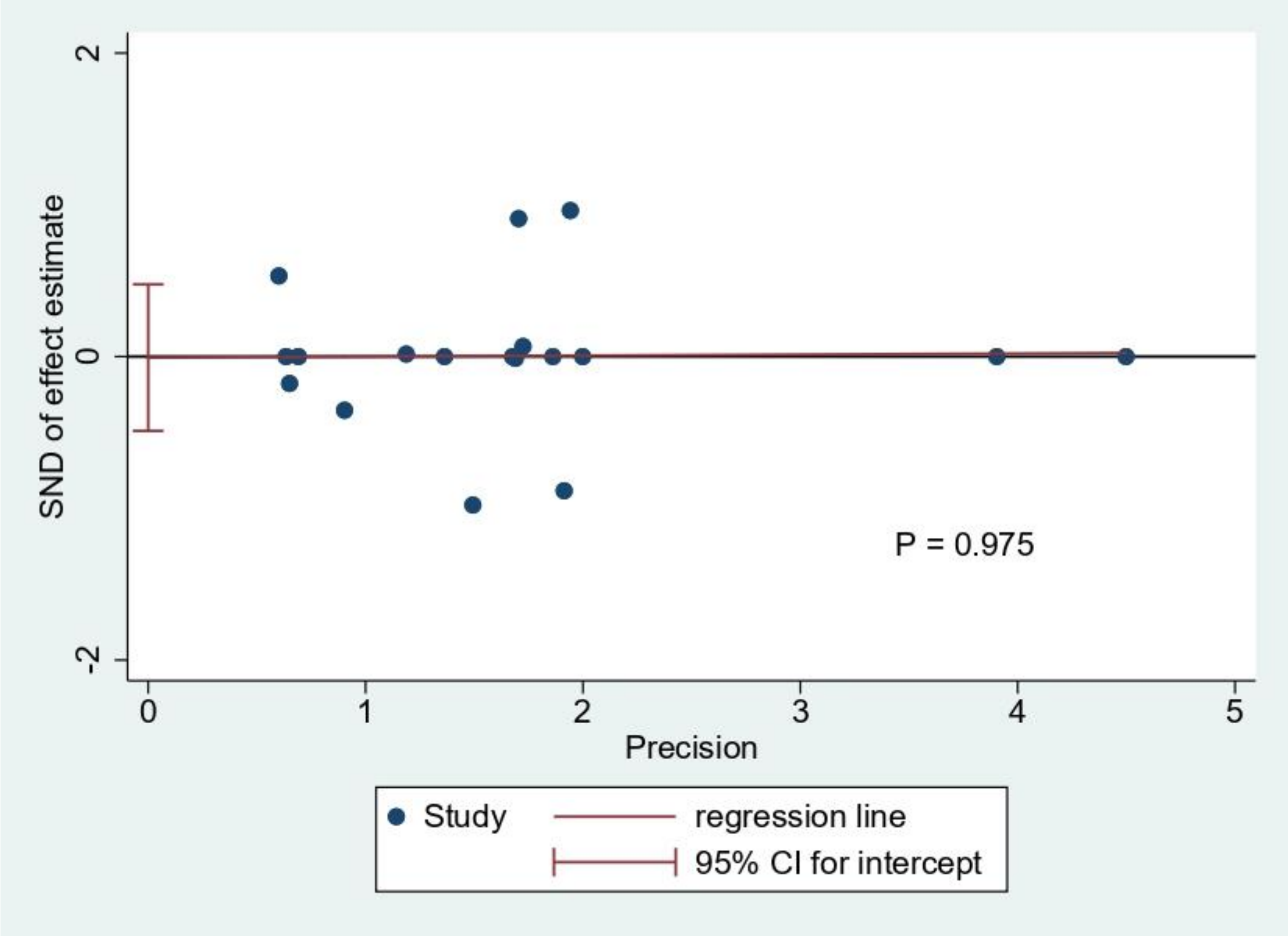

Supplementary Figure S18. Egger’s test

(A) pain intensity at 4 hours after surgery, (B) pain intensity at 8 hours after surgery, (C) pain intensity at 12 hours after surgery, (D) pain intensity at 24 hours after surgery, (E) nausea/vomiting, (F) pruritus, and (G) sedation/drowsiness.

Supplementary Table S11. GRADE approach for rating the quality of evidence

A. Pain intensity at 4 hours after surgery

Comparison	Direct evidence		Indirect evidence		Network evidence	
	Mean difference (95% confidence interval)	Quality of evidence	Mean difference (95% confidence interval)	Quality of evidence	Mean difference (95% confidence interval)	Quality of evidence
IVPCA morphine vs. IVPCA fentanyl	-	-	-0.11 (-0.99 to 0.77)	Moderate	-0.11 (-0.99 to 0.77)	Moderate
IVPCA morphine vs. IVPCA meperidine	-0.71 (-2.40 to 0.98)	Moderate	-0.33 (-1.43 to 0.77)	Moderate	-0.44 (-1.36 to 0.48)	Moderate
IVPCA morphine vs. IVPCA oxymorphone	-0.40 (-1.04 to 0.24)	Low	0.50 (-2.62 to 3.63)	Moderate	-0.36 (-0.99 to 0.26)	Moderate
IVPCA morphine vs. IVPCA sufentanil	-	-	-0.49 (-1.62 to 0.64)	Very low	-0.49 (-1.62 to 0.64)	Very low
IVPCA morphine vs. IVPCA tramadol	-	-	-0.41 (-1.47 to 0.65)	Low	-0.41 (-1.47 to 0.65)	Low
IVPCA morphine vs. PCEA buprenorphine	-	-	0.79 (-0.36 to 1.94)	Moderate	0.79 (-0.36 to 1.94)	Moderate
IVPCA morphine vs. PCEA fentanyl	0.78 (0.36 to 1.20)	High	0.26 (-1.54 to 2.05)	Moderate	0.75 (0.34 to 1.16)	High
IVPCA morphine vs. PCEA meperidine	-	-	1.45 (0.42 to 2.48)	High	1.45 (0.42 to 2.48)	High
IVPCA fentanyl vs. IVPCA meperidine	-0.66 (-2.26 to 0.94)	Moderate	-0.07 (-1.51 to 1.38)	Moderate	-0.33 (-1.40 to 0.74)	Moderate
IVPCA fentanyl vs. IVPCA oxymorphone	-	-	-0.26 (-1.31 to 0.79)	Moderate	-0.26 (-1.31 to 0.79)	Moderate
IVPCA fentanyl vs. IVPCA sufentanil	-	-	-0.38 (-1.08 to 0.32)	Low	-0.38 (-1.08 to 0.32)	Low
IVPCA fentanyl vs. IVPCA	-0.30 (-0.90 to 0.30)	Low	-	-	-0.30 (-0.90 to 0.30)	Low

tramadol						
IVPCA fentanyl vs. PCEA buprenorphine	-	-	0.90 (-0.43 to 2.24)	Moderate	0.90 (-0.43 to 2.24)	Moderate
IVPCA fentanyl vs. PCEA fentanyl	0.82 (0.02 to 1.63)	High	2.15 (-2.46 to 6.76)	Moderate	0.86 (0.07 to 1.66)	High
IVPCA fentanyl vs. PCEA meperidine	1.17 (-0.34 to 2.69)	Moderate	2.02 (0.36 to 3.67)	High	1.56 (0.44 to 2.68)	High
IVPCA meperidine vs. IVPCA oxymorphone	0.34 (-1.18 to 1.86)	Moderate	-0.13 (-1.48 to 1.22)	Low	0.08 (-0.93 to 1.09)	Moderate
IVPCA meperidine vs. IVPCA sufentanil	-	-	-0.05 (-1.33 to 1.23)	Low	-0.05 (-1.33 to 1.23)	Low
IVPCA meperidine vs. IVPCA tramadol	-	-	0.03 (-1.19 to 1.26)	Low	0.03 (-1.19 to 1.26)	Low
IVPCA meperidine vs. PCEA buprenorphine	-	-	1.24 (-0.17 to 2.64)	Moderate	1.24 (-0.17 to 2.64)	Moderate
IVPCA meperidine vs. PCEA fentanyl	0.67 (-0.74 to 2.07)	Moderate	1.57 (0.39 to 2.76)	High	1.20 (0.29 to 2.10)	High
IVPCA meperidine vs. PCEA meperidine	1.83 (0.97 to 2.68)	Moderate	2.72 (-0.31 to 5.74)	Moderate	1.89 (1.07 to 2.72)	High
IVPCA oxymorphone vs. IVPCA sufentanil	-	-	-0.12 (-1.39 to 1.14)	Very low	-0.12 (-1.39 to 1.14)	Very low
IVPCA oxymorphone vs. IVPCA tramadol	-	-	-0.04 (-1.25 to 1.16)	Low	-0.04 (-1.25 to 1.16)	Low
IVPCA oxymorphone vs. PCEA buprenorphine	-	-	1.16 (-0.14 to 2.46)	Low	1.16 (-0.14 to 2.46)	Low
IVPCA oxymorphone vs. PCEA fentanyl	-	-	1.12 (0.39 to 1.85)	Moderate	1.12 (0.39 to 1.85)	Moderate
IVPCA oxymorphone vs.	-	-	1.82 (0.68 to 2.95)	Moderate	1.82 (0.68 to 2.95)	Moderate

PCEA meperidine						
IVPCA sufentanil vs. IVPCA tramadol	0.08 (-0.29 to 0.45)	Moderate	-	-	0.08 (-0.29 to 0.45)	Moderate
IVPCA sufentanil vs. PCEA buprenorphine	-	-	1.28 (-0.23 to 2.79)	Very low	1.28 (-0.23 to 2.79)	Very low
IVPCA sufentanil vs. PCEA fentanyl	-	-	1.24 (0.18 to 2.30)	Moderate	1.24 (0.18 to 2.30)	Moderate
IVPCA sufentanil vs. PCEA meperidine	-	-	1.94 (0.62 to 3.26)	Moderate	1.94 (0.62 to 3.26)	Moderate
IVPCA tramadol vs. PCEA buprenorphine	-	-	1.20 (-0.26 to 2.67)	Low	1.20 (-0.26 to 2.67)	Low
IVPCA tramadol vs. PCEA fentanyl	-	-	1.16 (0.17 to 2.16)	Moderate	1.16 (0.17 to 2.16)	Moderate
IVPCA tramadol vs. PCEA meperidine	-	-	1.86 (0.59 to 3.13)	Moderate	1.86 (0.59 to 3.13)	Moderate
PCEA buprenorphine vs. PCEA fentanyl	-0.04 (-1.12 to 1.03)	Moderate	-	-	-0.04 (-1.12 to 1.03)	Moderate
PCEA buprenorphine vs. PCEA meperidine	-	-	0.66 (-0.81 to 2.12)	Moderate	0.66 (-0.81 to 2.12)	Moderate
PCEA fentanyl vs. PCEA meperidine	1.17 (-0.14 to 2.48)	Moderate	0.05 (-1.47 to 1.58)	Moderate	0.70 (-0.30 to 1.69)	Moderate

IVPCA: intravenous patient-controlled analgesia; PCEA: patient-controlled epidural analgesia

B. Pain intensity at 8 hours after surgery

Comparison	Direct evidence		Indirect evidence		Network evidence	
	Mean difference (95% confidence interval)	Quality of evidence	Mean difference (95% confidence interval)	Quality of evidence	Mean difference (95% confidence interval)	Quality of evidence
IVPCA morphine vs. IVPCA	-	-	-0.33 (-1.32 to 0.66)	Moderate	-0.33 (-1.32 to 0.66)	Moderate

fentanyl						
IVPCA morphine vs. IVPCA meperidine	-0.68 (-2.45 to 1.09)	Moderate	0.14 (-0.88 to 1.15)	Moderate	-0.07 (-0.95 to 0.82)	Moderate
IVPCA morphine vs. IVPCA oxymorphone	-0.19 (-0.89 to 0.52)	Low	1.25 (-1.43 to 3.92)	Moderate	-0.09 (-0.78 to 0.59)	Moderate
IVPCA morphine vs. IVPCA sufentanil	-	-	0.11 (-1.13 to 1.35)	Very low	0.11 (-1.13 to 1.35)	Very low
IVPCA morphine vs. IVPCA tramadol	-	-	0.01 (-1.17 to 1.19)	Moderate	0.01 (-1.17 to 1.19)	Low
IVPCA morphine vs. PCEA buprenorphine	-	-	1.25 (0.08 to 2.43)	High	1.25 (0.08 to 2.43)	High
IVPCA morphine vs. PCEA fentanyl	1.06 (0.36 to 1.77)	High	0.12 (-1.57 to 1.81)	High	0.93 (0.28 to 1.57)	High
IVPCA morphine vs. PCEA meperidine	-	-	1.33 (0.36 to 2.30)	High	1.33 (0.36 to 2.30)	High
IVPCA morphine vs. PCEA sufentanil	-	-	0.59 (-0.07 to 1.25)	Moderate	0.59 (-0.07 to 1.25)	Moderate
IVPCA fentanyl vs. IVPCA meperidine	-0.08 (-1.49 to 1.34)	Moderate	0.56 (-0.75 to 1.87)	Moderate	0.27 (-0.70 to 1.23)	Moderate
IVPCA fentanyl vs. IVPCA oxymorphone	-	-	0.24 (-0.91 to 1.38)	Moderate	0.24 (-0.91 to 1.38)	Moderate
IVPCA fentanyl vs. IVPCA sufentanil	-	-	0.44 (-0.30 to 1.19)	Low	0.44 (-0.30 to 1.19)	Low
IVPCA fentanyl vs. IVPCA tramadol	0.34 (-0.30 to 0.98)	Low	-	-	0.34 (-0.30 to 0.98)	Low
IVPCA fentanyl vs. PCEA buprenorphine	-	-	1.58 (0.33 to 2.84)	High	1.58 (0.33 to 2.84)	High
IVPCA fentanyl vs. PCEA	1.21 (0.43 to 1.99)	High	4.76 (-2.04 to 11.57)	Moderate	1.26 (0.48 to 2.03)	High

fentanyl						
IVPCA fentanyl vs. PCEA meperidine	1.09 (-0.38 to 2.56)	Moderate	2.18 (0.77 to 3.59)	High	1.66 (0.64 to 2.68)	High
IVPCA fentanyl vs. PCEA sufentanil	-	-	0.92 (0.13 to 1.71)	High	0.92 (0.13 to 1.71)	High
IVPCA meperidine vs. IVPCA oxymorphone	0.47 (-1.02 to 1.96)	Moderate	-0.44 (-1.79 to 0.91)	Low	-0.03 (-1.03 to 0.97)	Moderate
IVPCA meperidine vs. IVPCA sufentanil	-	-	0.18 (-1.04 to 1.40)	Low	0.18 (-1.04 to 1.40)	Low
IVPCA meperidine vs. IVPCA tramadol	-	-	0.07 (-1.08 to 1.23)	Low	0.07 (-1.08 to 1.23)	Low
IVPCA meperidine vs. PCEA buprenorphine	-	-	1.32 (0.08 to 2.56)	High	1.32 (0.08 to 2.56)	High
IVPCA meperidine vs. PCEA fentanyl	0.50 (-0.47 to 1.46)	Moderate	1.79 (0.57 to 3.02)	High	0.99 (0.23 to 1.75)	High
IVPCA meperidine vs. PCEA meperidine	1.36 (0.78 to 1.94)	Moderate	3.77 (-0.84 to 8.39)	Moderate	1.40 (0.82 to 1.97)	High
IVPCA meperidine vs. PCEA sufentanil	-	-	0.66 (-0.11 to 1.43)	Moderate	0.66 (-0.11 to 1.43)	Moderate
IVPCA oxymorphone vs. IVPCA sufentanil	-	-	0.21 (-1.16 to 1.58)	Very low	0.21 (-1.16 to 1.58)	Very low
IVPCA oxymorphone vs. IVPCA tramadol	-	-	0.10 (-1.21 to 1.42)	Low	0.10 (-1.21 to 1.42)	Low
IVPCA oxymorphone vs. PCEA buprenorphine	-	-	1.35 (0.02 to 2.67)	Moderate	1.35 (0.02 to 2.67)	Moderate
IVPCA oxymorphone vs. PCEA fentanyl	-	-	1.02 (0.13 to 1.91)	Moderate	1.02 (0.13 to 1.91)	Moderate
IVPCA oxymorphone vs.	-	-	1.43 (0.33 to 2.52)	Moderate	1.43 (0.33 to 2.52)	Moderate

PCEA meperidine						
IVPCA oxymorphone vs. PCEA sufentanil	-	-	0.69 (-0.21 to 1.58)	Very low	0.69 (-0.21 to 1.58)	Very low
IVPCA sufentanil vs. IVPCA tramadol	-0.10 (-0.49 to 0.29)	Moderate	-	-	-0.10 (-0.49 to 0.29)	Moderate
IVPCA sufentanil vs. PCEA buprenorphine	-	-	1.14 (-0.32 to 2.60)	Very low	1.14 (-0.32 to 2.60)	Very low
IVPCA sufentanil vs. PCEA fentanyl	-	-	0.81 (-0.27 to 1.89)	Low	0.81 (-0.27 to 1.89)	Low
IVPCA sufentanil vs. PCEA meperidine	-	-	1.22 (-0.05 to 2.48)	Low	1.22 (-0.05 to 2.48)	Low
IVPCA sufentanil vs. PCEA sufentanil	-	-	0.48 (-0.61 to 1.57)	Very low	0.48 (-0.61 to 1.57)	Very low
IVPCA tramadol vs. PCEA buprenorphine	-	-	1.24 (-0.16 to 2.65)	Low	1.24 (-0.16 to 2.65)	Low
IVPCA tramadol vs. PCEA fentanyl	-	-	0.92 (-0.09 to 1.92)	Low	0.92 (-0.09 to 1.92)	Low
IVPCA tramadol vs. PCEA meperidine	-	-	1.32 (0.12 to 2.52)	Moderate	1.32 (0.12 to 2.52)	Moderate
IVPCA tramadol vs. PCEA sufentanil	-	-	0.58 (-0.43 to 1.60)	Low	0.58 (-0.43 to 1.60)	Low
PCEA buprenorphine vs. PCEA fentanyl	-0.33 (-1.31 to 0.65)	Moderate	-	-	-0.33 (-1.31 to 0.65)	Moderate
PCEA buprenorphine vs. PCEA meperidine	-	-	0.08 (-1.21 to 1.37)	Moderate	0.08 (-1.21 to 1.37)	Moderate
PCEA buprenorphine vs. PCEA sufentanil	-	-	-0.66 (-1.65 to 0.33)	Moderate	-0.66 (-1.65 to 0.33)	Moderate
PCEA fentanyl vs. PCEA	0.67 (-0.38 to 1.72)	Moderate	-0.06 (-1.45 to 1.33)	Low	0.41 (-0.43 to 1.24)	Moderate

meperidine						
PCEA fentanyl vs. PCEA sufentanil	-0.33 (-0.46 to -0.20)	High	-	-	-0.33 (-0.46 to -0.20)	High
PCEA meperidine vs. PCEA sufentanil	-	-	-0.74 (-1.59 to 0.11)	Moderate	-0.74 (-1.59 to 0.11)	Moderate

IVPCA: intravenous patient-controlled analgesia; PCEA: patient-controlled epidural analgesia

C. Pain intensity at 12 hours after surgery

Comparison	Direct evidence		Indirect evidence		Network evidence	
	Mean difference (95% confidence interval)	Quality of evidence	Mean difference (95% confidence interval)	Quality of evidence	Mean difference (95% confidence interval)	Quality of evidence
IVPCA morphine vs. IVPCA fentanyl	-	-	-2.21 (-3.26 to -1.15)	High	-2.21 (-3.26 to -1.15)	High
IVPCA morphine vs. IVPCA meperidine	-0.91 (-1.96 to 0.14)	Moderate	-1.35 (-2.31 to -0.38)	High	-1.15 (-1.86 to -0.44)	High
IVPCA morphine vs. IVPCA oxymorphone	-1.03 (-1.51 to -0.54)	Moderate	-1.44 (-4.04 to 1.16)	Moderate	-1.04 (-1.52 to -0.57)	High
IVPCA morphine vs. IVPCA sufentanil	-	-	-1.75 (-3.04 to -0.47)	Low	-1.75 (-3.04 to -0.47)	Low
IVPCA morphine vs. IVPCA tramadol	-	-	-1.67 (-2.91 to -0.42)	Moderate	-1.67 (-2.91 to -0.42)	Moderate
IVPCA morphine vs. PCEA fentanyl	-0.06 (-1.03 to 0.91)	Moderate	-0.1 (-1.08 to 0.87)	Moderate	-0.08 (-0.77 to 0.61)	Moderate
IVPCA morphine vs. PCEA meperidine	-	-	-0.23 (-1.03 to 0.57)	Low	-0.23 (-1.03 to 0.57)	Low
IVPCA morphine vs. PCEA sufentanil	-0.04 (-1.50 to 1.42)	Low	0.33 (-0.58 to 1.23)	Moderate	0.22 (-0.54 to 0.99)	Moderate
IVPCA morphine vs. PCEA	-	-	0.11 (-1.64 to 1.86)	Low	0.11 (-1.64 to 1.86)	Low

tramadol						
IVPCA morphine vs. IVPCA hydromorphone	-	-	-1.67 (-2.93 to -0.40)	Low	-1.67 (-2.93 to -0.40)	Low
IVPCA fentanyl vs. IVPCA meperidine	0.18 (-1.60 to 1.95)	Moderate	1.52 (0.24 to 2.81)	High	1.06 (0.02 to 2.10)	High
IVPCA fentanyl vs. IVPCA oxymorphone	-	-	1.16 (0.05 to 2.28)	High	1.16 (0.05 to 2.28)	High
IVPCA fentanyl vs. IVPCA sufentanil	-	-	0.45 (-0.29 to 1.20)	Low	0.45 (-0.29 to 1.20)	Low
IVPCA fentanyl vs. IVPCA tramadol	0.54 (-0.13 to 1.21)	Low	-	-	0.54 (-0.13 to 1.21)	Low
IVPCA fentanyl vs. PCEA fentanyl	2.09 (1.24 to 2.93)	High	3.78 (-1.71 to 9.28)	Moderate	2.12 (1.29 to 2.96)	High
IVPCA fentanyl vs. PCEA meperidine	1.60 (0.13 to 3.07)	High	2.28 (0.95 to 3.61)	Moderate	1.97 (0.99 to 2.96)	High
IVPCA fentanyl vs. PCEA sufentanil	-	-	2.43 (1.48 to 3.38)	High	2.43 (1.48 to 3.38)	High
IVPCA fentanyl vs. PCEA tramadol	-	-	2.32 (0.48 to 4.16)	High	2.32 (0.48 to 4.16)	High
IVPCA fentanyl vs. IVPCA hydromorphone	-	-	0.54 (-0.16 to 1.24)	Low	0.54 (-0.16 to 1.24)	Low
IVPCA meperidine vs. IVPCA oxymorphone	0.16 (-0.80 to 1.12)	Moderate	0.02 (-1.16 to 1.2)	Low	0.10 (-0.64 to 0.85)	Moderate
IVPCA meperidine vs. IVPCA sufentanil	-	-	-0.61 (-1.89 to 0.67)	Low	-0.61 (-1.89 to 0.67)	Low
IVPCA meperidine vs. IVPCA tramadol	-	-	-0.52 (-1.76 to 0.72)	Low	-0.52 (-1.76 to 0.72)	Low
IVPCA meperidine vs. PCEA	1.07 (-0.33 to 2.46)	Moderate	1.06 (0.21 to 1.91)	High	1.06 (0.34 to 1.79)	High

fentanyl						
IVPCA meperidine vs. PCEA meperidine	0.99 (0.31 to 1.67)	Moderate	0.49 (-1.12 to 2.1)	Moderate	0.91 (0.28 to 1.54)	High
IVPCA meperidine vs. PCEA sufentanil	-	-	1.37 (0.54 to 2.20)	High	1.37 (0.54 to 2.20)	High
IVPCA meperidine vs. PCEA tramadol	-	-	1.26 (-0.52 to 3.04)	Moderate	1.26 (-0.52 to 3.04)	Moderate
IVPCA meperidine vs. IVPCA hydromorphone	-	-	-0.52 (-1.78 to 0.74)	Low	-0.52 (-1.78 to 0.74)	Low
IVPCA oxymorphone vs. IVPCA sufentanil	-	-	-0.71 (-2.05 to 0.63)	Very low	-0.71 (-2.05 to 0.63)	Very low
IVPCA oxymorphone vs. IVPCA tramadol	-	-	-0.62 (-1.92 to 0.68)	Low	-0.62 (-1.92 to 0.68)	Low
IVPCA oxymorphone vs. PCEA fentanyl	-	-	0.96 (0.17 to 1.75)	Moderate	0.96 (0.17 to 1.75)	Moderate
IVPCA oxymorphone vs. PCEA meperidine	-	-	0.81 (-0.05 to 1.67)	Low	0.81 (-0.05 to 1.67)	Low
IVPCA oxymorphone vs. PCEA sufentanil	-	-	1.27 (0.40 to 2.13)	Moderate	1.27 (0.40 to 2.13)	Moderate
IVPCA oxymorphone vs. PCEA tramadol	-	-	1.15 (-0.64 to 2.95)	Low	1.15 (-0.64 to 2.95)	Low
IVPCA oxymorphone vs. IVPCA hydromorphone	-	-	-0.62 (-1.94 to 0.69)	Very low	-0.62 (-1.94 to 0.69)	Very low
IVPCA sufentanil vs. IVPCA tramadol	0.09 (-0.24 to 0.41)	Moderate	-	-	0.09 (-0.24 to 0.41)	Moderate
IVPCA sufentanil vs. PCEA fentanyl	-	-	1.67 (0.55 to 2.79)	Moderate	1.67 (0.55 to 2.79)	Moderate
IVPCA sufentanil vs. PCEA	-	-	1.52 (0.29 to 2.76)	Low	1.52 (0.29 to 2.76)	Low

meperidine						
IVPCA sufentanil vs. PCEA sufentanil	-	-	1.98 (0.77 to 3.19)	Low	1.98 (0.77 to 3.19)	Low
IVPCA sufentanil vs. PCEA tramadol	-	-	1.87 (-0.12 to 3.85)	Very low	1.87 (-0.12 to 3.85)	Very low
IVPCA sufentanil vs. IVPCA hydromorphone	-	-	0.09 (-0.30 to 0.47)	Moderate	0.09 (-0.30 to 0.47)	Moderate
IVPCA tramadol vs. PCEA fentanyl	-	-	1.58 (0.51 to 2.66)	Moderate	1.58 (0.51 to 2.66)	Moderate
IVPCA tramadol vs. PCEA meperidine	-	-	1.43 (0.24 to 2.63)	Moderate	1.43 (0.24 to 2.63)	Moderate
IVPCA tramadol vs. PCEA sufentanil	-	-	1.89 (0.73 to 3.05)	Moderate	1.89 (0.73 to 3.05)	Moderate
IVPCA tramadol vs. PCEA tramadol	-	-	1.78 (-0.18 to 3.74)	Very low	1.78 (-0.18 to 3.74)	Very low
IVPCA tramadol vs. IVPCA hydromorphone	0.00 (-0.20 to 0.20)	Moderate	-	-	0.00 (-0.20 to 0.20)	Moderate
PCEA fentanyl vs. PCEA meperidine	-0.05 (-0.82 to 0.72)	Low	-0.44 (-1.73 to 0.85)	Low	-0.15 (-0.81 to 0.51)	Low
PCEA fentanyl vs. PCEA sufentanil	0.33 (-0.14 to 0.81)	Moderate	-0.03 (-1.68 to 1.62)	Low	0.31 (-0.15 to 0.76)	Moderate
PCEA fentanyl vs. PCEA tramadol	-	-	0.19 (-1.45 to 1.83)	Moderate	0.19 (-1.45 to 1.83)	Moderate
PCEA fentanyl vs. IVPCA hydromorphone	-	-	-1.58 (-2.68 to -0.49)	Moderate	-1.58 (-2.68 to -0.49)	Moderate
PCEA meperidine vs. PCEA sufentanil	-	-	0.46 (-0.33 to 1.24)	Low	0.46 (-0.33 to 1.24)	Low
PCEA meperidine vs. PCEA	-	-	0.35 (-1.42 to 2.11)	Low	0.35 (-1.42 to 2.11)	Low

tramadol						
PCEA meperidine vs. IVPCA hydromorphone	-	-	-1.43 (-2.64 to -0.22)	Moderate	-1.43 (-2.64 to -0.22)	Moderate
PCEA sufentanil vs. PCEA tramadol	-0.11 (-1.69 to 1.46)	Moderate	-	-	-0.11 (-1.69 to 1.46)	Moderate
PCEA sufentanil vs. IVPCA hydromorphone	-	-	-1.89 (-3.07 to -0.71)	Low	-1.89 (-3.07 to -0.71)	Low
PCEA tramadol vs. IVPCA hydromorphone	-	-	-1.78 (-3.75 to 0.19)	Very low	-1.78 (-3.75 to 0.19)	Very low

IVPCA: intravenous patient-controlled analgesia; PCEA: patient-controlled epidural analgesia

D. Pain intensity at 24 hours after surgery

Comparison	Direct evidence		Indirect evidence		Network evidence	
	Mean difference (95% confidence interval)	Quality of evidence	Mean difference (95% confidence interval)	Quality of evidence	Mean difference (95% confidence interval)	Quality of evidence
IVPCA morphine vs. IVPCA fentanyl	-	-	-2.59 (-4.08 to -1.11)	Low	-2.59 (-4.08 to -1.11)	Low
IVPCA morphine vs. IVPCA meperidine	-0.86 (-1.80 to 0.08)	Moderate	-1.56 (-3.66 to 0.54)	Very low	-0.98 (-1.83 to -0.12)	High
IVPCA morphine vs. IVPCA methadone	0.27 (0.00 to 0.53)	Low	-	-	0.27 (0.00 to 0.53)	Low
IVPCA morphine vs. IVPCA oxymorphone	-1.19 (-1.60 to -0.77)	Moderate	-4.30 (-19.99 to 11.38)	Low	-1.19 (-1.60 to -0.78)	Moderate
IVPCA morphine vs. IVPCA sufentanil	-	-	-2.29 (-3.87 to -0.71)	Low	-2.29 (-3.87 to -0.71)	Low
IVPCA morphine vs. IVPCA tramadol	-	-	-2.19 (-3.74 to -0.64)	Low	-2.19 (-3.74 to -0.64)	Low
IVPCA morphine vs. PCEA	-	-	0.10 (-1.52 to 1.72)	Very low	0.10 (-1.52 to 1.72)	Very low

buprenorphine						
IVPCA morphine vs. PCEA fentanyl	-	-	-0.19 (-1.40 to 1.02)	Low	-0.19 (-1.40 to 1.02)	Low
IVPCA morphine vs. PCEA meperidine	-	-	0.05 (-1.17 to 1.26)	Very low	0.05 (-1.17 to 1.26)	Very low
IVPCA morphine vs. PCEA sufentanil	0.01 (-1.29 to 1.30)	Low	0.57 (-1.96 to 3.10)	Very low	0.12 (-1.03 to 1.28)	Low
IVPCA morphine vs. PCEA tramadol	-	-	-0.08 (-1.61 to 1.46)	Very low	-0.08 (-1.61 to 1.46)	Very low
IVPCA morphine vs. PCEA hydromorphone	-	-	-1.53 (-3.45 to 0.39)	Very low	-1.53 (-3.45 to 0.39)	Very low
IVPCA morphine vs. IVPCA hydromorphone	-	-	-2.86 (-4.43 to -1.29)	Low	-2.86 (-4.43 to -1.29)	Low
IVPCA fentanyl vs. IVPCA meperidine	-	-	1.62 (0.02 to 3.21)	Moderate	1.62 (0.02 to 3.21)	Moderate
IVPCA fentanyl vs. IVPCA methadone	-	-	2.86 (1.35 to 4.37)	Low	2.86 (1.35 to 4.37)	Low
IVPCA fentanyl vs. IVPCA oxymorphone	-	-	1.41 (-0.13 to 2.94)	Very low	1.41 (-0.13 to 2.94)	Very low
IVPCA fentanyl vs. IVPCA sufentanil	-	-	0.30 (-0.24 to 0.85)	Low	0.30 (-0.24 to 0.85)	Low
IVPCA fentanyl vs. IVPCA tramadol	0.40 (-0.05 to 0.85)	Low	-	-	0.40 (-0.05 to 0.85)	Low
IVPCA fentanyl vs. PCEA buprenorphine	-	-	2.69 (1.31 to 4.07)	High	2.69 (1.31 to 4.07)	High
IVPCA fentanyl vs. PCEA fentanyl	2.40 (1.54 to 3.26)	High	-	-	2.40 (1.54 to 3.26)	High
IVPCA fentanyl vs. PCEA	-	-	2.64 (0.98 to 4.30)	Moderate	2.64 (0.98 to 4.30)	Moderate

meperidine						
IVPCA fentanyl vs. PCEA sufentanil	-	-	2.72 (1.73 to 3.70)	High	2.72 (1.73 to 3.70)	High
IVPCA fentanyl vs. PCEA tramadol	-	-	2.52 (1.10 to 3.93)	Moderate	2.52 (1.10 to 3.93)	Moderate
IVPCA fentanyl vs. PCEA hydromorphone	-	-	1.06 (-0.16 to 2.28)	Low	1.06 (-0.16 to 2.28)	Low
IVPCA fentanyl vs. IVPCA hydromorphone	-	-	-0.27 (-0.79 to 0.25)	Low	-0.27 (-0.79 to 0.25)	Low
IVPCA meperidine vs. IVPCA methadone	-	-	1.24 (0.35 to 2.14)	Moderate	1.24 (0.35 to 2.14)	Moderate
IVPCA meperidine vs. IVPCA oxymorphone	0.00 (-1.50 to 1.50)	Moderate	-0.34 (-1.51 to 0.83)	Very low	-0.21 (-1.13 to 0.71)	Moderate
IVPCA meperidine vs. IVPCA sufentanil	-	-	-1.31 (-3.00 to 0.37)	Very low	-1.31 (-3.00 to 0.37)	Very low
IVPCA meperidine vs. IVPCA tramadol	-	-	-1.22 (-2.88 to 0.44)	Very low	-1.22 (-2.88 to 0.44)	Very low
IVPCA meperidine vs. PCEA buprenorphine	-	-	1.07 (-0.65 to 2.80)	Low	1.07 (-0.65 to 2.80)	Low
IVPCA meperidine vs. PCEA fentanyl	-	-	0.78 (-0.56 to 2.13)	Low	0.78 (-0.56 to 2.13)	Low
IVPCA meperidine vs. PCEA meperidine	1.10 (0.04 to 2.16)	Moderate	0.54 (-2.10 to 3.17)	Very low	1.02 (0.04 to 2.00)	Moderate
IVPCA meperidine vs. PCEA sufentanil	-	-	1.10 (-0.21 to 2.41)	Low	1.10 (-0.21 to 2.41)	Low
IVPCA meperidine vs. PCEA tramadol	-	-	0.90 (-0.76 to 2.56)	Low	0.90 (-0.76 to 2.56)	Low
IVPCA meperidine vs. PCEA	-	-	-0.56 (-2.56 to 1.45)	Very low	-0.56 (-2.56 to 1.45)	Very low

hydromorphone						
IVPCA meperidine vs. IVPCA hydromorphone	-	-	-1.88 (-3.56 to -0.20)	Low	-1.88 (-3.56 to -0.20)	Low
IVPCA methadone vs. IVPCA oxymorphone	-	-	-1.45 (-1.94 to -0.96)	Moderate	-1.45 (-1.94 to -0.96)	Moderate
IVPCA methadone vs. IVPCA sufentanil	-	-	-2.56 (-4.16 to -0.95)	Low	-2.56 (-4.16 to -0.95)	Low
IVPCA methadone vs. IVPCA tramadol	-	-	-2.46 (-4.03 to -0.89)	Low	-2.46 (-4.03 to -0.89)	Low
IVPCA methadone vs. PCEA buprenorphine	-	-	-0.17 (-1.81 to 1.47)	Very low	-0.17 (-1.81 to 1.47)	Very low
IVPCA methadone vs. PCEA fentanyl	-	-	-0.46 (-1.70 to 0.78)	Low	-0.46 (-1.70 to 0.78)	Low
IVPCA methadone vs. PCEA meperidine	-	-	-0.22 (-1.46 to 1.02)	Low	-0.22 (-1.46 to 1.02)	Low
IVPCA methadone vs. PCEA sufentanil	-	-	-0.14 (-1.32 to 1.04)	Low	-0.14 (-1.32 to 1.04)	Low
IVPCA methadone vs. PCEA tramadol	-	-	-0.34 (-1.90 to 1.21)	Low	-0.34 (-1.90 to 1.21)	Low
IVPCA methadone vs. PCEA hydromorphone	-	-	-1.80 (-3.73 to 0.14)	Very low	-1.80 (-3.73 to 0.14)	Very low
IVPCA methadone vs. IVPCA hydromorphone	-	-	-3.13 (-4.72 to -1.53)	Low	-3.13 (-4.72 to -1.53)	Low
IVPCA oxymorphone vs. IVPCA sufentanil	-	-	-1.10 (-2.73 to 0.53)	Very low	-1.10 (-2.73 to 0.53)	Very low
IVPCA oxymorphone vs. IVPCA tramadol	-	-	-1.01 (-2.60 to 0.59)	Very low	-1.01 (-2.60 to 0.59)	Very low
IVPCA oxymorphone vs.	-	-	1.28 (-0.38 to 2.95)	Very low	1.28 (-0.38 to 2.95)	Very low

PCEA buprenorphine						
IVPCA oxymorphone vs. PCEA fentanyl	-	-	1.00 (-0.28 to 2.27)	Low	1.00 (-0.28 to 2.27)	Low
IVPCA oxymorphone vs. PCEA meperidine	-	-	1.23 (-0.03 to 2.50)	Low	1.23 (-0.03 to 2.50)	Low
IVPCA oxymorphone vs. PCEA sufentanil	-	-	1.31 (0.09 to 2.53)	Moderate	1.31 (0.09 to 2.53)	Moderate
IVPCA oxymorphone vs. PCEA tramadol	-	-	1.11 (-0.47 to 2.70)	Low	1.11 (-0.47 to 2.70)	Low
IVPCA oxymorphone vs. PCEA hydromorphone	-	-	-0.34 (-2.30 to 1.61)	Very low	-0.34 (-2.30 to 1.61)	Very low
IVPCA oxymorphone vs. IVPCA hydromorphone	-	-	-1.67 (-3.29 to -0.05)	Low	-1.67 (-3.29 to -0.05)	Low
IVPCA sufentanil vs. IVPCA tramadol	0.10 (-0.21 to 0.41)	Moderate	-	-	0.10 (-0.21 to 0.41)	Moderate
IVPCA sufentanil vs. PCEA buprenorphine	-	-	2.38 (0.90 to 3.87)	Moderate	2.38 (0.90 to 3.87)	Moderate
IVPCA sufentanil vs. PCEA fentanyl	-	-	2.10 (1.08 to 3.12)	Moderate	2.10 (1.08 to 3.12)	Moderate
IVPCA sufentanil vs. PCEA meperidine	-	-	2.33 (0.59 to 4.08)	Moderate	2.33 (0.59 to 4.08)	Moderate
IVPCA sufentanil vs. PCEA sufentanil	-	-	2.41 (1.29 to 3.54)	Moderate	2.41 (1.29 to 3.54)	Moderate
IVPCA sufentanil vs. PCEA tramadol	-	-	2.21 (0.70 to 3.73)	Low	2.21 (0.70 to 3.73)	Low
IVPCA sufentanil vs. PCEA hydromorphone	-	-	0.76 (-0.42 to 1.93)	Low	0.76 (-0.42 to 1.93)	Low
IVPCA sufentanil vs. IVPCA	-	-	-0.57 (-0.98 to -0.17)	High	-0.57 (-0.98 to -0.17)	High

hydromorphone						
IVPCA tramadol vs. PCEA buprenorphine	-	-	2.29 (0.84 to 3.74)	Moderate	2.29 (0.84 to 3.74)	Moderate
IVPCA tramadol vs. PCEA fentanyl	-	-	2.00 (1.03 to 2.97)	Moderate	2.00 (1.03 to 2.97)	Moderate
IVPCA tramadol vs. PCEA meperidine	-	-	2.24 (0.52 to 3.96)	Moderate	2.24 (0.52 to 3.96)	Moderate
IVPCA tramadol vs. PCEA sufentanil	-	-	2.32 (1.23 to 3.40)	Moderate	2.32 (1.23 to 3.40)	Moderate
IVPCA tramadol vs. PCEA tramadol	-	-	2.12 (0.63 to 3.60)	Low	2.12 (0.63 to 3.60)	Low
IVPCA tramadol vs. PCEA hydromorphone	-	-	0.66 (-0.47 to 1.79)	Low	0.66 (-0.47 to 1.79)	Low
IVPCA tramadol vs. IVPCA hydromorphone	-0.67 (-0.93 to -0.41)	High	-	-	-0.67 (-0.93 to -0.41)	High
PCEA buprenorphine vs. PCEA fentanyl	-0.29 (-1.37 to 0.79)	Moderate	-	-	-0.29 (-1.37 to 0.79)	Moderate
PCEA buprenorphine vs. PCEA meperidine	-	-	-0.05 (-1.83 to 1.73)	Low	-0.05 (-1.83 to 1.73)	Low
PCEA buprenorphine vs. PCEA sufentanil	-	-	0.03 (-1.15 to 1.21)	Moderate	0.03 (-1.15 to 1.21)	Moderate
PCEA buprenorphine vs. PCEA tramadol	-	-	-0.17 (-1.73 to 1.39)	Low	-0.17 (-1.73 to 1.39)	Low
PCEA buprenorphine vs. PCEA hydromorphone	-	-	-1.63 (-3.47 to 0.21)	Very low	-1.63 (-3.47 to 0.21)	Very low
PCEA buprenorphine vs. IVPCA hydromorphone	-	-	-2.96 (-4.43 to -1.48)	Low	-2.96 (-4.43 to -1.48)	Low
PCEA fentanyl vs. PCEA	-0.06 (-2.11 to 2.00)	Low	0.51 (-1.45 to 2.47)	Very low	0.24 (-1.18 to 1.66)	Low

meperidine						
PCEA fentanyl vs. PCEA sufentanil	0.33 (-0.15 to 0.82)	Moderate	-0.23 (-3.03 to 2.57)	Very low	0.32 (-0.16 to 0.80)	Moderate
PCEA fentanyl vs. PCEA tramadol	-	-	0.12 (-1.00 to 1.24)	Low	0.12 (-1.00 to 1.24)	Low
PCEA fentanyl vs. PCEA hydromorphone	-	-	-1.34 (-2.83 to 0.15)	Very low	-1.34 (-2.83 to 0.15)	Very low
PCEA fentanyl vs. IVPCA hydromorphone	-	-	-2.67 (-3.67 to -1.66)	Moderate	-2.67 (-3.67 to -1.66)	Moderate
PCEA meperidine vs. PCEA sufentanil	-	-	0.08 (-1.33 to 1.49)	Low	0.08 (-1.33 to 1.49)	Low
PCEA meperidine vs. PCEA tramadol	-	-	-0.12 (-1.86 to 1.62)	Low	-0.12 (-1.86 to 1.62)	Low
PCEA meperidine vs. PCEA hydromorphone	-	-	-1.58 (-3.64 to 0.48)	Very low	-1.58 (-3.64 to 0.48)	Very low
PCEA meperidine vs. IVPCA hydromorphone	-	-	-2.91 (-4.64 to -1.17)	Low	-2.91 (-4.64 to -1.17)	Low
PCEA sufentanil vs. PCEA tramadol	-0.20 (-1.21 to 0.81)	Low	-	-	-0.20 (-1.21 to 0.81)	Low
PCEA sufentanil vs. PCEA hydromorphone	-	-	-1.66 (-3.22 to -0.09)	Low	-1.66 (-3.22 to -0.09)	Low
PCEA sufentanil vs. IVPCA hydromorphone	-	-	-2.98 (-4.10 to -1.87)	Low	-2.98 (-4.10 to -1.87)	Low
PCEA tramadol vs. PCEA hydromorphone	-	-	-1.46 (-3.32 to 0.41)	Very low	-1.46 (-3.32 to 0.41)	Very low
PCEA tramadol vs. IVPCA hydromorphone	-	-	-2.78 (-4.29 to -1.28)	Low	-2.78 (-4.29 to -1.28)	Low
PCEA hydromorphone vs.	-1.33 (-2.43 to -0.23)	Low	-	-	-1.33 (-2.43 to -0.23)	Low

IVPCA hydromorphone						

IVPCA: intravenous patient-controlled analgesia; PCEA: patient-controlled epidural analgesia

E. Nausea/vomiting

Comparison	Direct evidence		Indirect evidence		Network evidence	
	Odds ratio (95% confidence interval)	Quality of evidence	Odds ratio (95% confidence interval)	Quality of evidence	Odds ratio (95% confidence interval)	Quality of evidence
IVPCA morphine vs. IVPCA fentanyl	0.41 (0.07 to 2.59)	Moderate	1.22 (0.22 to 6.67)	Moderate	0.74 (0.21 to 2.57)	Moderate
IVPCA morphine vs. IVPCA meperidine	1.50 (0.54 to 4.17)	Low	1.89 (0.11 to 33.33)	Low	1.54 (0.59 to 4.03)	Low
IVPCA morphine vs. IVPCA meptazinol	0.25 (0.06 to 1.09)	Moderate	-	-	0.25 (0.06 to 1.09)	Moderate
IVPCA morphine vs. IVPCA methadone	1.13 (0.34 to 3.77)	Low	-	-	1.13 (0.34 to 3.77)	Low
IVPCA morphine vs. IVPCA oxymorphone	0.38 (0.11 to 1.41)	Low	0.48 (0.03 to 8.33)	Low	0.40 (0.12 to 1.30)	Low
IVPCA morphine vs. IVPCA sufentanil	-	-	0.54 (0.09 to 3.35)	Low	0.54 (0.09 to 3.35)	Low
IVPCA morphine vs. IVPCA tramadol	-	-	0.64 (0.12 to 3.28)	Low	0.64 (0.12 to 3.28)	Low
IVPCA morphine vs. PCEA buprenorphine	-	-	0.26 (0.03 to 2.04)	Moderate	0.26 (0.03 to 2.04)	Moderate
IVPCA morphine vs. PCEA fentanyl	4.78 (1.24 to 18.49)	High	2.33 (0.37 to 14.29)	Moderate	3.71 (1.25 to 11.01)	High
IVPCA morphine vs. PCEA meperidine	-	-	5.18 (1.19 to 22.65)	Moderate	5.18 (1.19 to 22.65)	Moderate
IVPCA morphine vs. PCEA	-	-	2.62 (0.67 to 10.25)	Moderate	2.62 (0.67 to 10.25)	Moderate

sufentanil						
IVPCA morphine vs. PCEA tramadol	-	-	0.89 (0.12 to 6.80)	Low	0.89 (0.12 to 6.80)	Low
IVPCA morphine vs. PCEA hydromorphone	-	-	1.74 (0.15 to 19.49)	Very low	1.74 (0.15 to 19.49)	Very low
IVPCA morphine vs. IVPCA hydromorphone	-	-	0.85 (0.15 to 4.77)	Low	0.85 (0.15 to 4.77)	Low
IVPCA fentanyl vs. IVPCA meperidine	-	-	2.08 (0.45 to 9.60)	Low	2.08 (0.45 to 9.60)	Low
IVPCA fentanyl vs. IVPCA meptazinol	-	-	0.34 (0.05 to 2.32)	Moderate	0.34 (0.05 to 2.32)	Moderate
IVPCA fentanyl vs. IVPCA methadone	-	-	1.53 (0.27 to 8.64)	Low	1.53 (0.27 to 8.64)	Low
IVPCA fentanyl vs. IVPCA oxymorphone	-	-	0.54 (0.10 to 2.96)	Low	0.54 (0.10 to 2.96)	Low
IVPCA fentanyl vs. IVPCA sufentanil	-	-	0.72 (0.19 to 2.78)	Low	0.72 (0.19 to 2.78)	Low
IVPCA fentanyl vs. IVPCA tramadol	0.86 (0.30 to 2.50)	Low	-	-	0.86 (0.30 to 2.50)	Low
IVPCA fentanyl vs. PCEA buprenorphine	-	-	0.35 (0.05 to 2.64)	Moderate	0.35 (0.05 to 2.64)	Moderate
IVPCA fentanyl vs. PCEA fentanyl	4.02 (1.30 to 12.45)	High	12.5 (1.28 to 100)	Moderate	5.01 (1.83 to 13.75)	High
IVPCA fentanyl vs. PCEA meperidine	-	-	7.01 (1.59 to 30.98)	Moderate	7.01 (1.59 to 30.98)	Moderate
IVPCA fentanyl vs. PCEA sufentanil	-	-	3.55 (0.97 to 13.02)	Moderate	3.55 (0.97 to 13.02)	Moderate
IVPCA fentanyl vs. PCEA	-	-	1.20 (0.16 to 8.82)	Low	1.20 (0.16 to 8.82)	Low

tramadol						
IVPCA fentanyl vs. PCEA hydromorphone	-	-	2.35 (0.30 to 18.65)	Low	2.35 (0.30 to 18.65)	Low
IVPCA fentanyl vs. IVPCA hydromorphone	-	-	1.15 (0.35 to 3.78)	Low	1.15 (0.35 to 3.78)	Low
IVPCA meperidine vs. IVPCA meptazinol	-	-	0.16 (0.03 to 0.94)	Moderate	0.16 (0.03 to 0.94)	Moderate
IVPCA meperidine vs. IVPCA methadone	-	-	0.73 (0.16 to 3.42)	Low	0.73 (0.16 to 3.42)	Low
IVPCA meperidine vs. IVPCA oxymorphone	0.25 (0.04 to 1.42)	Moderate	0.27 (0.04 to 1.97)	Low	0.26 (0.07 to 0.96)	High
IVPCA meperidine vs. IVPCA sufentanil	-	-	0.35 (0.05 to 2.66)	Very low	0.35 (0.05 to 2.66)	Very low
IVPCA meperidine vs. IVPCA tramadol	-	-	0.41 (0.06 to 2.67)	Low	0.41 (0.06 to 2.67)	Low
IVPCA meperidine vs. PCEA buprenorphine	-	-	0.17 (0.02 to 1.57)	Low	0.17 (0.02 to 1.57)	Low
IVPCA meperidine vs. PCEA fentanyl	-	-	2.41 (0.60 to 9.60)	Low	2.41 (0.60 to 9.60)	Low
IVPCA meperidine vs. PCEA meperidine	4.67 (0.21 to 111.11)	Low	2.94 (0.43 to 20)	Low	3.37 (0.65 to 17.37)	Low
IVPCA meperidine vs. PCEA sufentanil	-	-	1.70 (0.34 to 8.51)	Low	1.70 (0.34 to 8.51)	Low
IVPCA meperidine vs. PCEA tramadol	-	-	0.58 (0.06 to 5.24)	Very low	0.58 (0.06 to 5.24)	Very low
IVPCA meperidine vs. PCEA hydromorphone	-	-	1.13 (0.09 to 14.80)	Very low	1.13 (0.09 to 14.80)	Very low
IVPCA meperidine vs. IVPCA	-	-	0.55 (0.08 to 3.83)	Very low	0.55 (0.08 to 3.83)	Very low

hydromorphone						
IVPCA meptazinol vs. IVPCA methadone	-	-	4.52 (0.68 to 30.19)	Low	4.52 (0.68 to 30.19)	Low
IVPCA meptazinol vs. IVPCA oxymorphone	-	-	1.60 (0.24 to 10.50)	Low	1.60 (0.24 to 10.50)	Low
IVPCA meptazinol vs. IVPCA sufentanil	-	-	2.14 (0.20 to 22.42)	Very low	2.14 (0.20 to 22.42)	Very low
IVPCA meptazinol vs. IVPCA tramadol	-	-	2.55 (0.28 to 23.03)	Low	2.55 (0.28 to 23.03)	Low
IVPCA meptazinol vs. PCEA buprenorphine	-	-	1.03 (0.08 to 13.01)	Moderate	1.03 (0.08 to 13.01)	Moderate
IVPCA meptazinol vs. PCEA fentanyl	-	-	14.83 (2.38 to 92.21)	Moderate	14.83 (2.38 to 92.21)	Moderate
IVPCA meptazinol vs. PCEA meperidine	-	-	20.74 (2.59 to 166.12)	Low	20.74 (2.59 to 166.12)	Low
IVPCA meptazinol vs. PCEA sufentanil	-	-	10.49 (1.41 to 77.80)	Low	10.49 (1.41 to 77.80)	Moderate
IVPCA meptazinol vs. PCEA tramadol	-	-	3.55 (0.29 to 43.69)	Very low	3.55 (0.29 to 43.69)	Very low
IVPCA meptazinol vs. PCEA hydromorphone	-	-	6.94 (0.41 to 117.55)	Very low	6.94 (0.41 to 117.55)	Very low
IVPCA meptazinol vs. IVPCA hydromorphone	-	-	3.41 (0.36 to 32.76)	Very low	3.41 (0.36 to 32.76)	Very low
IVPCA methadone vs. IVPCA oxymorphone	-	-	0.35 (0.07 to 1.90)	Low	0.35 (0.07 to 1.90)	Low
IVPCA methadone vs. IVPCA sufentanil	-	-	0.47 (0.05 to 4.24)	Very low	0.47 (0.05 to 4.24)	Very low
IVPCA methadone vs. IVPCA	-	-	0.56 (0.07 to 4.31)	Low	0.56 (0.07 to 4.31)	Low

tramadol						
IVPCA methadone vs. PCEA buprenorphine	-	-	0.23 (0.02 to 2.49)	Low	0.23 (0.02 to 2.49)	Low
IVPCA methadone vs. PCEA fentanyl	-	-	3.28 (0.65 to 16.60)	Low	3.28 (0.65 to 16.60)	Low
IVPCA methadone vs. PCEA meperidine	-	-	4.58 (0.68 to 30.74)	Low	4.58 (0.68 to 30.74)	Low
IVPCA methadone vs. PCEA sufentanil	-	-	2.32 (0.38 to 14.29)	Low	2.32 (0.38 to 14.29)	Low
IVPCA methadone vs. PCEA tramadol	-	-	0.78 (0.07 to 8.35)	Very low	0.78 (0.07 to 8.35)	Very low
IVPCA methadone vs. PCEA hydromorphone	-	-	1.53 (0.10 to 22.86)	Very low	1.53 (0.10 to 22.86)	Very low
IVPCA methadone vs. IVPCA hydromorphone	-	-	0.75 (0.09 to 6.16)	Very low	0.75 (0.09 to 6.16)	Very low
IVPCA oxymorphone vs. IVPCA sufentanil	-	-	1.34 (0.15 to 11.71)	Very low	1.34 (0.15 to 11.71)	Very low
IVPCA oxymorphone vs. IVPCA tramadol	-	-	1.60 (0.22 to 11.87)	Low	1.60 (0.22 to 11.87)	Low
IVPCA oxymorphone vs. PCEA buprenorphine	-	-	0.64 (0.06 to 6.87)	Low	0.64 (0.06 to 6.87)	Low
IVPCA oxymorphone vs. PCEA fentanyl	-	-	9.27 (1.90 to 45.21)	Moderate	9.27 (1.90 to 45.21)	Moderate
IVPCA oxymorphone vs. PCEA meperidine	-	-	12.97 (2.04 to 82.65)	Moderate	12.97 (2.04 to 82.65)	Moderate
IVPCA oxymorphone vs. PCEA sufentanil	-	-	6.56 (1.10 to 39.08)	Moderate	6.56 (1.10 to 39.08)	Moderate
IVPCA oxymorphone vs. PCEA	-	-	2.22 (0.21 to 23.03)	Very low	2.22 (0.21 to 23.03)	Very low

tramadol						
IVPCA oxymorphone vs. PCEA hydromorphone	-	-	4.34 (0.30 to 63.44)	Very low	4.34 (0.30 to 63.44)	Very low
IVPCA oxymorphone vs. IVPCA hydromorphone	-	-	2.13 (0.27 to 16.99)	Very low	2.13 (0.27 to 16.99)	Very low
IVPCA sufentanil vs. IVPCA tramadol	1.19 (0.52 to 2.73)	Moderate	-	-	1.19 (0.52 to 2.73)	Moderate
IVPCA sufentanil vs. PCEA buprenorphine	-	-	0.48 (0.04 to 5.48)	Very low	0.48 (0.04 to 5.48)	Very low
IVPCA sufentanil vs. PCEA fentanyl	-	-	6.93 (1.29 to 37.23)	Moderate	6.93 (1.29 to 37.23)	Moderate
IVPCA sufentanil vs. PCEA meperidine	-	-	9.69 (1.31 to 71.90)	Low	9.69 (1.31 to 71.90)	Low
IVPCA sufentanil vs. PCEA sufentanil	-	-	4.90 (0.75 to 31.84)	Very low	4.90 (0.75 to 31.84)	Very low
IVPCA sufentanil vs. PCEA tramadol	-	-	1.66 (0.15 to 18.39)	Very low	1.66 (0.15 to 18.39)	Very low
IVPCA sufentanil vs. PCEA hydromorphone	-	-	3.24 (0.46 to 23.08)	Low	3.24 (0.46 to 23.08)	Low
IVPCA sufentanil vs. IVPCA hydromorphone	-	-	1.59 (0.60 to 4.25)	Moderate	1.59 (0.60 to 4.25)	Moderate
IVPCA tramadol vs. PCEA buprenorphine	-	-	0.40 (0.04 to 3.98)	Low	0.40 (0.04 to 3.98)	Low
IVPCA tramadol vs. PCEA fentanyl	-	-	5.80 (1.34 to 25.11)	Moderate	5.80 (1.34 to 25.11)	Moderate
IVPCA tramadol vs. PCEA meperidine	-	-	8.12 (1.31 to 50.41)	Moderate	8.12 (1.31 to 50.41)	Moderate
IVPCA tramadol vs. PCEA	-	-	4.11 (0.77 to 22.02)	Low	4.11 (0.77 to 22.02)	Low

sufentanil						
IVPCA tramadol vs. PCEA tramadol	-	-	1.39 (0.15 to 13.31)	Very low	1.39 (0.15 to 13.31)	Very low
IVPCA tramadol vs. PCEA hydromorphone	-	-	2.72 (0.46 to 16.12)	Low	2.72 (0.46 to 16.12)	Low
IVPCA tramadol vs. IVPCA hydromorphone	1.33 (0.79 to 2.27)	Moderate	-	-	1.34 (0.79 to 2.27)	Moderate
PCEA buprenorphine vs. PCEA fentanyl	14.29 (2.48 to 83.33)	Moderate	-	-	14.39 (2.48 to 83.41)	Moderate
PCEA buprenorphine vs. PCEA meperidine	-	-	20.12 (2.50 to 162.13)	Low	20.12 (2.50 to 162.13)	Low
PCEA buprenorphine vs. PCEA sufentanil	-	-	10.18 (1.46 to 70.82)	High	10.18 (1.46 to 70.82)	High
PCEA buprenorphine vs. PCEA tramadol	-	-	3.44 (0.29 to 40.30)	Low	3.44 (0.29 to 40.30)	Low
PCEA buprenorphine vs. PCEA hydromorphone	-	-	6.74 (0.37 to 122.29)	Very low	6.74 (0.37 to 122.29)	Very low
PCEA buprenorphine vs. IVPCA hydromorphone	-	-	3.31 (0.32 to 34.66)	Very low	3.31 (0.32 to 34.66)	Very low
PCEA fentanyl vs. PCEA meperidine	1.33 (0.41 to 4.39)	Low	2.1 (0.07 to 67.58)	Low	1.40 (0.45 to 4.31)	Low
PCEA fentanyl vs. PCEA sufentanil	0.71 (0.31 to 1.61)	Moderate	-	-	0.71 (0.31 to 1.61)	Moderate
PCEA fentanyl vs. PCEA tramadol	-	-	0.24 (0.04 to 1.34)	Low	0.24 (0.04 to 1.34)	Low
PCEA fentanyl vs. PCEA hydromorphone	-	-	0.47 (0.05 to 4.70)	Very low	0.47 (0.05 to 4.70)	Very low
PCEA fentanyl vs. IVPCA	-	-	0.23 (0.05 to 1.09)	Low	0.23 (0.05 to 1.09)	Low

hydromorphone						
PCEA meperidine vs. PCEA sufentanil	-	-	0.51 (0.13 to 2.04)	Low	0.51 (0.13 to 2.04)	Low
PCEA meperidine vs. PCEA tramadol	-	-	0.17 (0.02 to 1.34)	Low	0.17 (0.02 to 1.34)	Low
PCEA meperidine vs. PCEA hydromorphone	-	-	0.33 (0.03 to 4.29)	Very low	0.33 (0.03 to 4.29)	Very low
PCEA meperidine vs. IVPCA hydromorphone	-	-	0.16 (0.02 to 1.10)	Very low	0.16 (0.02 to 1.10)	Very low
PCEA sufentanil vs. PCEA tramadol	0.34 (0.07 to 1.54)	Low	-	-	0.34 (0.07 to 1.54)	Low
PCEA sufentanil vs. PCEA hydromorphone	-	-	0.66 (0.06 to 7.65)	Very low	0.66 (0.06 to 7.65)	Very low
PCEA sufentanil vs. IVPCA hydromorphone	-	-	0.33 (0.06 to 1.89)	Very low	0.33 (0.06 to 1.89)	Very low
PCEA tramadol vs. PCEA hydromorphone	-	-	1.96 (0.11 to 34.73)	Very low	1.96 (0.11 to 34.73)	Very low
PCEA tramadol vs. IVPCA hydromorphone	-	-	0.96 (0.09 to 9.79)	Very low	0.96 (0.09 to 9.79)	Very low
PCEA hydromorphone vs. IVPCA hydromorphone	0.49 (0.09 to 2.69)	Low	-	-	0.49 (0.09 to 2.69)	Low

IVPCA: intravenous patient-controlled analgesia; PCEA: patient-controlled epidural analgesia

F. Pruritus

Comparison	Direct evidence		Indirect evidence		Network evidence	
	Odds ratio (95% confidence interval)	Quality of evidence	Odds ratio (95% confidence interval)	Quality of evidence	Odds ratio (95% confidence interval)	Quality of evidence
IVPCA morphine vs. IVPCA	3.00 (0.11 to 76.92)	Moderate	1.62 (0.34 to 7.84)	Low	1.81 (0.45 to 7.28)	Moderate

fentanyl						
IVPCA morphine vs. IVPCA meperidine	3.68 (1.06 to 12.66)	Moderate	3.36 (0.29 to 38.39)	Low	3.58 (1.09 to 11.69)	Moderate
IVPCA morphine vs. IVPCA methadone	8.00 (1.04 to 62.50)	Moderate	-	-	8.03 (0.87 to 74.43)	Low
IVPCA morphine vs. IVPCA oxymorphone	2.56 (0.53 to 12.35)	Low	1.1 (0.005 to 259.34)	Low	2.42 (0.45 to 12.98)	Low
IVPCA morphine vs. PCEA buprenorphine	-	-	2.00 (0.43 to 9.39)	Moderate	2.00 (0.43 to 9.39)	Moderate
IVPCA morphine vs. PCEA fentanyl	0.96 (0.38 to 2.43)	Moderate	0.24 (0.06 to 0.97)	Low	0.56 (0.21 to 1.51)	Moderate
IVPCA morphine vs. PCEA meperidine	-	-	1.06 (0.26 to 4.23)	Low	1.06 (0.26 to 4.23)	Low
IVPCA morphine vs. PCEA sufentanil	0.11 (0.02 to 0.60)	Low	1.35 (0.43 to 4.23)	Moderate	0.47 (0.12 to 1.82)	Low
IVPCA morphine vs. PCEA tramadol	-	-	8.34 (0.29 to 241.11)	Low	8.34 (0.29 to 241.11)	Low
IVPCA fentanyl vs. IVPCA meperidine	-	-	1.98 (0.37 to 10.72)	Low	1.98 (0.37 to 10.72)	Low
IVPCA fentanyl vs. IVPCA methadone	-	-	4.44 (0.32 to 61.47)	Low	4.44 (0.32 to 61.47)	Low
IVPCA fentanyl vs. IVPCA oxymorphone	-	-	1.34 (0.16 to 11.62)	Low	1.34 (0.16 to 11.62)	Low
IVPCA fentanyl vs. PCEA buprenorphine	-	-	1.11 (0.22 to 5.54)	Low	1.11 (0.22 to 5.54)	Low
IVPCA fentanyl vs. PCEA fentanyl	0.32 (0.08 to 1.23)	Low	0.18 (0 to 6.23)	Moderate	0.31 (0.10 to 0.92)	High
IVPCA fentanyl vs. PCEA	-	-	0.59 (0.11 to 3.10)	Low	0.59 (0.11 to 3.10)	Low

meperidine						
IVPCA fentanyl vs. PCEA sufentanil	-	-	0.26 (0.05 to 1.28)	Low	0.26 (0.05 to 1.28)	Low
IVPCA fentanyl vs. PCEA tramadol	-	-	4.62 (0.14 to 147.73)	Low	4.62 (0.14 to 147.73)	Low
IVPCA meperidine vs. IVPCA methadone	-	-	2.24 (0.18 to 27.95)	Low	2.24 (0.18 to 27.95)	Low
IVPCA meperidine vs. IVPCA oxymorphone	0.50 (0.04 to 5.88)	Moderate	1.03 (0.04 to 24.64)	Low	0.68 (0.10 to 4.59)	Moderate
IVPCA meperidine vs. PCEA buprenorphine	-	-	0.56 (0.09 to 3.38)	Low	0.56 (0.09 to 3.38)	Low
IVPCA meperidine vs. PCEA fentanyl	-	-	0.16 (0.04 to 0.60)	Moderate	0.16 (0.04 to 0.60)	Moderate
IVPCA meperidine vs. PCEA meperidine	0.31 (0.09 to 1.13)	Low	0.26 (0.02 to 3.06)	Low	0.30 (0.08 to 1.04)	Low
IVPCA meperidine vs. PCEA sufentanil	-	-	0.13 (0.02 to 0.71)	Moderate	0.13 (0.02 to 0.71)	Moderate
IVPCA meperidine vs. PCEA tramadol	-	-	2.33 (0.07 to 77.90)	Low	2.33 (0.07 to 77.90)	Low
IVPCA methadone vs. IVPCA oxymorphone	-	-	0.30 (0.02 to 4.91)	Low	0.30 (0.02 to 4.91)	Low
IVPCA methadone vs. PCEA buprenorphine	-	-	0.25 (0.02 to 3.75)	Low	0.25 (0.02 to 3.75)	Low
IVPCA methadone vs. PCEA fentanyl	-	-	0.07 (0.01 to 0.79)	Moderate	0.07 (0.01 to 0.79)	Moderate
IVPCA methadone vs. PCEA meperidine	-	-	0.13 (0.01 to 1.82)	Low	0.13 (0.01 to 1.82)	Low
IVPCA methadone vs. PCEA	-	-	0.06 (0.00 to 0.79)	Moderate	0.06 (0.00 to 0.79)	Moderate

sufentanil						
IVPCA methadone vs. PCEA tramadol	-	-	1.04 (0.02 to 58.72)	Low	1.04 (0.02 to 58.72)	Low
IVPCA oxymorphone vs. PCEA buprenorphine	-	-	0.82 (0.09 to 7.89)	Low	0.82 (0.09 to 7.89)	Low
IVPCA oxymorphone vs. PCEA fentanyl	-	-	0.23 (0.03 to 1.57)	Low	0.23 (0.03 to 1.57)	Low
IVPCA oxymorphone vs. PCEA meperidine	-	-	0.44 (0.05 to 3.55)	Low	0.44 (0.05 to 3.55)	Low
IVPCA oxymorphone vs. PCEA sufentanil	-	-	0.19 (0.02 to 1.65)	Low	0.19 (0.02 to 1.65)	Low
IVPCA oxymorphone vs. PCEA tramadol	-	-	3.44 (0.08 to 146.09)	Low	3.44 (0.08 to 146.09)	Low
PCEA buprenorphine vs. PCEA fentanyl	0.27 (0.10 to 0.71)	High	-	-	0.28 (0.09 to 0.90)	High
PCEA buprenorphine vs. PCEA meperidine	-	-	0.53 (0.09 to 3.05)	Low	0.53 (0.09 to 3.05)	Low
PCEA buprenorphine vs. PCEA sufentanil	-	-	0.24 (0.05 to 1.18)	Moderate	0.24 (0.05 to 1.18)	Moderate
PCEA buprenorphine vs. PCEA tramadol	-	-	4.17 (0.13 to 134.80)	Low	4.17 (0.13 to 134.80)	Low
PCEA fentanyl vs. PCEA meperidine	1.8 (0.49 to 6.58)	Low	2.18 (0.2 to 24.12)	Low	1.90 (0.52 to 6.96)	Low
PCEA fentanyl vs. PCEA sufentanil	1.48 (0.68 to 3.25)	Moderate	0.12 (0.02 to 0.8)	Moderate	0.85 (0.27 to 2.61)	Low
PCEA fentanyl vs. PCEA tramadol	-	-	15.00 (0.57 to 397.74)	Low	15.00 (0.57 to 397.74)	Low
PCEA meperidine vs. PCEA	-	-	0.44 (0.08 to 2.37)	Low	0.44 (0.08 to 2.37)	Low

sufentanil						
PCEA meperidine vs. PCEA tramadol	-	-	7.88 (0.24 to 261.96)	Low	7.88 (0.24 to 261.96)	Low
PCEA sufentanil vs. PCEA tramadol	17.86 (0.93 to 333.33)	Low	-	-	17.73 (0.82 to 385.32)	Low

IVPCA: intravenous patient-controlled analgesia; PCEA: patient-controlled epidural analgesia

G. Sedation/drowsiness

	Direct evidence		Indirect evidence		Network evidence	
	Odds ratio (95% confidence interval)	Quality of evidence	Odds ratio (95% confidence interval)	Quality of evidence	Odds ratio (95% confidence interval)	Quality of evidence
IVPCA morphine vs. IVPCA fentanyl	-	-	0.59 (0.09 to 4.11)	Low	0.59 (0.09 to 4.11)	Low
IVPCA morphine vs. IVPCA meperidine	1.24 (0.58 to 2.67)	Low	1.18 (0.26 to 5.35)	Moderate	1.11 (0.56 to 2.24)	Moderate
IVPCA morphine vs. IVPCA meptazinol	5.68 (0.26 to 125)	Moderate	-	-	5.70 (0.26 to 125.36)	Moderate
IVPCA morphine vs. IVPCA methadone	0.82 (0.53 to 1.26)	Low	-	-	0.82 (0.53 to 1.26)	Low
IVPCA morphine vs. IVPCA oxymorphone	2.26 (1.02 to 5.03)	High	0.29 (0.02 to 5.4)	Low	2.26 (1.02 to 5.03)	High
IVPCA morphine vs. PCEA buprenorphine	-	-	3.82 (1.17 to 12.45)	Moderate	3.82 (1.17 to 12.45)	Moderate
IVPCA morphine vs. PCEA fentanyl	4.81 (1.80 to 12.82)	Moderate	4.22 (1.53 to 11.66)	High	4.51 (2.23 to 9.14)	High
IVPCA morphine vs. PCEA meperidine	-	-	6.66 (2.64 to 16.82)	Moderate	6.66 (2.64 to 16.82)	Moderate
IVPCA morphine vs. PCEA	2.11 (0.66 to 6.80)	Moderate	3.77 (1.41 to 10.07)	Moderate	2.96 (1.40 to 6.29)	High

sufentanil						
IVPCA morphine vs. PCEA tramadol	-	-	2.96 (0.16 to 55.78)	Low	2.96 (0.16 to 55.78)	Low
IVPCA fentanyl vs. IVPCA meperidine	-	-	1.88 (0.25 to 14.06)	Low	1.88 (0.25 to 14.06)	Low
IVPCA fentanyl vs. IVPCA meptazinol	-	-	9.64 (0.25 to 370.36)	Low	9.64 (0.25 to 370.36)	Low
IVPCA fentanyl vs. IVPCA methadone	-	-	1.38 (0.19 to 10.07)	Low	1.38 (0.19 to 10.07)	Low
IVPCA fentanyl vs. IVPCA oxymorphone	-	-	3.83 (0.47 to 31.16)	Low	3.83 (0.47 to 31.16)	Low
IVPCA fentanyl vs. PCEA buprenorphine	-	-	6.45 (0.84 to 49.66)	Moderate	6.45 (0.84 to 49.66)	Moderate
IVPCA fentanyl vs. PCEA fentanyl	7.63 (1.25 to 45.45)	High	-	-	7.63 (1.25 to 46.48)	High
IVPCA fentanyl vs. PCEA meperidine	-	-	11.27 (1.52 to 83.55)	High	11.27 (1.52 to 83.55)	High
IVPCA fentanyl vs. PCEA sufentanil	-	-	5.01 (0.77 to 32.43)	Moderate	5.01 (0.77 to 32.43)	Moderate
IVPCA fentanyl vs. PCEA tramadol	-	-	5.01 (0.17 to 149.61)	Low	5.01 (0.17 to 149.61)	Low
IVPCA meperidine vs. IVPCA meptazinol	-	-	5.12 (0.22 to 121.62)	Low	5.12 (0.22 to 121.62)	Low
IVPCA meperidine vs. IVPCA methadone	-	-	0.73 (0.32 to 1.67)	Low	0.73 (0.32 to 1.67)	Low
IVPCA meperidine vs. IVPCA oxymorphone	0.77 (0.18 to 3.23)	Moderate	6.55 (1.35 to 31.65)	Moderate	2.03 (0.70 to 5.87)	Low
IVPCA meperidine vs. PCEA	-	-	3.43 (0.94 to 12.51)	Low	3.43 (0.94 to 12.51)	Low

buprenorphine						
IVPCA meperidine vs. PCEA fentanyl	-	-	4.05 (1.68 to 9.78)	Moderate	4.05 (1.68 to 9.78)	Moderate
IVPCA meperidine vs. PCEA meperidine	7.35 (2.54 to 21.28)	Moderate	3.93 (0.86 to 17.87)	Low	5.98 (2.51 to 14.29)	Moderate
IVPCA meperidine vs. PCEA sufentanil	-	-	2.66 (1.04 to 6.79)	Moderate	2.66 (1.04 to 6.79)	Moderate
IVPCA meperidine vs. PCEA tramadol	-	-	2.66 (0.13 to 52.78)	Low	2.66 (0.13 to 52.78)	Low
IVPCA meptazinol vs. IVPCA methadone	-	-	0.14 (0.01 to 3.25)	Low	0.14 (0.01 to 3.25)	Low
IVPCA meptazinol vs. IVPCA oxymorphone	-	-	0.40 (0.02 to 9.67)	Low	0.40 (0.02 to 9.67)	Low
IVPCA meptazinol vs. PCEA buprenorphine	-	-	0.67 (0.02 to 18.33)	Low	0.67 (0.02 to 18.33)	Low
IVPCA meptazinol vs. PCEA fentanyl	-	-	0.79 (0.03 to 18.87)	Low	0.79 (0.03 to 18.87)	Low
IVPCA meptazinol vs. PCEA meperidine	-	-	1.17 (0.05 to 29.47)	Low	1.17 (0.05 to 29.47)	Low
IVPCA meptazinol vs. PCEA sufentanil	-	-	0.52 (0.02 to 12.52)	Moderate	0.52 (0.02 to 12.52)	Moderate
IVPCA meptazinol vs. PCEA tramadol	-	-	0.52 (0.01 to 36.92)	Low	0.52 (0.01 to 36.92)	Low
IVPCA methadone vs. IVPCA oxymorphone	-	-	2.77 (1.11 to 6.89)	Moderate	2.77 (1.11 to 6.89)	Moderate
IVPCA methadone vs. PCEA buprenorphine	-	-	4.68 (1.33 to 16.49)	Moderate	4.68 (1.33 to 16.49)	Moderate
IVPCA methadone vs. PCEA	-	-	5.53 (2.42 to 12.67)	Moderate	5.53 (2.42 to 12.67)	Moderate

fentanyl						
IVPCA methadone vs. PCEA meperidine	-	-	8.17 (2.94 to 22.72)	Moderate	8.17 (2.94 to 22.72)	Moderate
IVPCA methadone vs. PCEA sufentanil	-	-	3.63 (1.52 to 8.67)	Moderate	3.63 (1.52 to 8.67)	Moderate
IVPCA methadone vs. PCEA tramadol	-	-	3.63 (0.19 to 70.59)	Low	3.63 (0.19 to 70.59)	Low
IVPCA oxymorphone vs. PCEA buprenorphine	-	-	1.69 (0.40 to 7.03)	Low	1.69 (0.40 to 7.03)	Low
IVPCA oxymorphone vs. PCEA fentanyl	-	-	2.00 (0.69 to 5.80)	Low	2.00 (0.69 to 5.80)	Low
IVPCA oxymorphone vs. PCEA meperidine	-	-	2.95 (0.87 to 10.02)	Low	2.95 (0.87 to 10.02)	Low
IVPCA oxymorphone vs. PCEA sufentanil	-	-	1.31 (0.44 to 3.93)	Moderate	1.31 (0.44 to 3.93)	Moderate
IVPCA oxymorphone vs. PCEA tramadol	-	-	1.31 (0.06 to 27.45)	Low	1.31 (0.06 to 27.45)	Low
PCEA buprenorphine vs. PCEA fentanyl	1.18 (0.46 to 3.06)	Moderate	-	-	1.18 (0.46 to 3.06)	Moderate
PCEA buprenorphine vs. PCEA meperidine	-	-	1.75 (0.48 to 6.31)	Moderate	1.75 (0.48 to 6.31)	Moderate
PCEA buprenorphine vs. PCEA sufentanil	-	-	0.78 (0.27 to 2.24)	Moderate	0.78 (0.27 to 2.24)	Moderate
PCEA buprenorphine vs. PCEA tramadol	-	-	0.78 (0.04 to 16.05)	Low	0.78 (0.04 to 16.05)	Low
PCEA fentanyl vs. PCEA meperidine	1.20 (0.42 to 3.45)	Moderate	2.26 (0.49 to 10.35)	Low	1.48 (0.62 to 3.51)	Moderate
PCEA fentanyl vs. PCEA	0.70 (0.42 to 1.15)	Moderate	0.39 (0.09 to 1.66)	Low	0.66 (0.41 to 1.05)	Moderate

sufentanil						
PCEA fentanyl vs. PCEA tramadol	-	-	0.66 (0.04 to 11.65)	Low	0.66 (0.04 to 11.65)	Low
PCEA meperidine vs. PCEA sufentanil	-	-	0.44 (0.17 to 1.16)	Moderate	0.44 (0.17 to 1.16)	Moderate
PCEA meperidine vs. PCEA tramadol	-	-	0.44 (0.02 to 8.87)	Low	0.44 (0.02 to 8.87)	Low
PCEA sufentanil vs. PCEA tramadol	1.00 (0.06 to 16.95)	Low	-	-	1.00 (0.06 to 17.07)	Low

IVPCA: intravenous patient-controlled analgesia; PCEA: patient-controlled epidural analgesia

References

1. Wu Z, Zhao P, Peng J, Fang L, Ding J, Yan G, et al. A Patient-Controlled Intravenous Analgesia With Tramadol Ameliorates Postpartum Depression in High-Risk Woman After Cesarean Section: A Randomized Controlled Trial. *Front Med (Lausanne)* 2021; **8**: 679159.
2. Chi X, Li M, Mei W, Liao M. Comparison of patient-controlled intravenous analgesia with sufentanil versus tramadol in post-cesarean section pain management and lactation after general anesthesia - a prospective, randomized, double-blind, controlled study. *J Pain Res* 2017; **10**: 1521-1527.
3. Ebneshahidi A, Akbari M, Heshmati B. Patient-controlled versus nurse-controlled post-operative analgesia after caesarean section. *Adv Biomed Res* 2012; **1**: 6.
4. Saracoglu A, Saracoglu KT, Umuroglu T, But A. The effectivity of fentanyl versus tramadol as intravenous patient-controlled analgesia after cesarean section. *Adv Clin Exp Med* 2010; **19**: 739-743.
5. Cohen S, Pantuck CB, Amar D, Burley E, Pantuck EJ. The primary action of epidural fentanyl after cesarean delivery is via a spinal mechanism. *Anesth Analg* 2002; **94**: 674-679.
6. Cooper DW, Saleh U, Taylor M, Whyte S, Ryall D, Kokri MS, et al. Patient-controlled analgesia: epidural fentanyl and i.v. morphine compared after caesarean section. *Br J Anaesth* 1999; **82**: 366-370.
7. Kim CJ, Chea JS, Chung MY, Lee BH, Yoon JW. The Analgesic and Hemostatic Effects of Diclofenac as Adjuvant of Intravenous Opioid Using PCA after Cesarean Section. *Korean J Anesthesiol* 1999; **36**: 256-262.
8. Vercauteren MP, Mertens E, Schols G, Van Mol I, Adriaensen HA. Patient-controlled extradural analgesia after caesarean section: a comparison between tramadol, sufentanil and a mixture of both. *Eur J Pain* 1999; **3**: 205-210.
9. James KS, Davidson IT, McGrady E. Patient-controlled analgesia following caesarean section: a comparison of morphine and meptazinol. *Int J Obstet Anesth* 1997; **6**: 93-96.
10. Lee BH, Lim YG, Chea JS, Kim CJ, Chung MY, Jung JY. Morphine and Meperidine Analgesic Effect Using Intravenous PCA of Intramuscular Diclofenac after Cesarean Section. *Korean J Anesthesiol* 1997; **33**: 510-516.
11. Ngan Kee WD, Lam KK, Chen PP, Gin T. Comparison of patient-controlled epidural analgesia with patient-controlled intravenous analgesia using pethidine or fentanyl. *Anaesth Intensive Care* 1997; **25**: 126-132.
12. Goh JL, Evans SF, Pavy TJ. Patient-controlled epidural analgesia following caesarean delivery: a comparison of pethidine and fentanyl. *Anaesth Intensive Care* 1996; **24**: 45-50.
13. Cooper DW, Ryall DM, Desira WR. Extradural fentanyl for postoperative analgesia: predominant spinal or systemic action? *Br J Anaesth* 1995; **74**: 184-187.
14. Howell PR, Gambling DR, Pavy T, McMorland G, Douglas MJ. Patient-controlled analgesia following caesarean section under general anaesthesia: a comparison of fentanyl with morphine. *Can J Anaesth* 1995; **42**: 41-45.
15. Lee BH, Chea JS, Chung MY, Byun HJ. A Comparative Effect of Meperidine between Intravenous and Epidural Patient-Controlled Analgesia for the Postoperative Pain Relief after Cesarean Section. *Korean J Pain* 1995; **8**: 257-265.
16. Grass JA, Zuckerman RL, Sakima NT, Harris AP. Patient-controlled analgesia after cesarean delivery: epidural sufentanil versus intravenous morphine. *Reg Anesth Pain Med* 1994; **19**: 90-97.
17. Paech MJ, Moore JS, Evans SF. Meperidine for patient-controlled analgesia after cesarean section.

Intravenous versus epidural administration. *Anesthesiology* 1994; **80**: 1268-1276.

18. Cohen S, Amar D, Pantuck CB, Pantuck EJ, Goodman EJ, Widroff JS, et al. Postcesarean delivery epidural patient-controlled analgesia. Fentanyl or sufentanil? *Anesthesiology* 1993; **78**: 486-491.
19. Cohen S, Amar D, Pantuck CB, Pantuck EJ, Weissman AM, Landa S, et al. Epidural patient-controlled analgesia after cesarean section: buprenorphine-0.015% bupivacaine with epinephrine versus fentanyl-0.015% bupivacaine with and without epinephrine. *Anesth Analg* 1992; **74**: 226-230.
20. Cohen S, Amar D, Pantuck CB, Pantuck EJ, Weissman AB. Adverse effects of epidural 0.03% bupivacaine during analgesia after cesarean section. *Anesth Analg* 1992; **75**: 753-756.
21. Parker RK, White PF. Epidural patient-controlled analgesia: an alternative to intravenous patient-controlled analgesia for pain relief after cesarean delivery. *Anesthesia and analgesia* 1992; **75**: 245-251.
22. Sinatra RS, Lodge K, Sibert K, Chung KS, Chung JH, Parker A, et al. A comparison of morphine, meperidine, and oxymorphone as utilized in patient-controlled analgesia following cesarean delivery. *Anesthesiology* 1989; **70**: 585-590.
23. Sinatra R, Chung KS, Silverman DG, Brull SJ, Chung J, Harrison DM, et al. An evaluation of morphine and oxymorphone administered via patient-controlled analgesia (PCA) or PCA plus basal infusion in postcesarean-delivery patients. *Anesthesiology* 1989; **71**: 502-507.
